# Age, gender and UV-exposition related effects on gene expression in *in vivo* aged short term cultivated human dermal fibroblasts

**DOI:** 10.1371/journal.pone.0175657

**Published:** 2017-05-05

**Authors:** Wolfgang Kaisers, Petra Boukamp, Hans-Jürgen Stark, Holger Schwender, Julia Tigges, Jean Krutmann, Heiner Schaal

**Affiliations:** 1 Center for Bioinformatics and Biostatistics, BMFZ, Heinrich-Heine-Universität Düsseldorf, Düsseldorf, Germany; 2 Mathematical Institute, Heinrich-Heine-Universität Düsseldorf, Düsseldorf, Germany; 3 Institut für Virologie, Heinrich-Heine-Universität Düsseldorf, Düsseldorf, Germany; 4 IUF - Leibniz Research Institute for Environmental Medicine, Düsseldorf, Germany; 5 German Cancer Research Center (DKFZ), Heidelberg, Germany; 6 Medical Faculty, Heinrich-Heine-Universität Düsseldorf, Düsseldorf, Germany; University of Kansas Medical Center, UNITED STATES

## Abstract

Ageing, the progressive functional decline of virtually all tissues, affects numerous living organisms. Main phenotypic alterations of human skin during the ageing process include reduced skin thickness and elasticity which are related to extracellular matrix proteins. Dermal fibroblasts, the main source of extracellular fibrillar proteins, exhibit complex alterations during *in vivo* ageing and any of these are likely to be accompanied or caused by changes in gene expression. We investigated gene expression of short term cultivated *in vivo* aged human dermal fibroblasts using RNA-seq. Therefore, fibroblast samples derived from unaffected skin were obtained from 30 human donors. The donors were grouped by gender and age (Young: 19 to 25 years, Middle: 36 to 45 years, Old: 60 to 66 years). Two samples were taken from each donor, one from a sun-exposed and one from a sun-unexposed site. In our data, no consistently changed gene expression associated with donor age can be asserted. Instead, highly correlated expression of a small number of genes associated with transforming growth factor beta signalling was observed. Also, known gene expression alterations of *in vivo* aged dermal fibroblasts seem to be non-detectable in cultured fibroblasts.

## Introduction

Various biological effects have been related to ageing including accumulation of DNA damage and reactive oxygen species (ROS), metabolic alterations (especially energy metabolism) and cellular senescence [1]. Many of these ageing related effects, like accumulation of mutations in somatic and mitochondrial DNA and telomere attrition are not directly linked to altered mRNA expression. But still, transcriptomic profiling, by outlining many physiologic effects in parallel, provides sensible information on global uniformly age associated effects.

For whole transcriptome analysis, RNA-seq is an established platform [2]. For each step of analysis (alignment to genome, differential expression analysis, functional classification), a variety of standard technologies are available. Gene expression in normal cells [3] and age-related alterations of gene expression [4] have been found to be very tissue specific. Age-related gene expression changes are also species specific [4].

### Ageing skin

During the ageing process, human skin undergoes characteristic morphological and functional changes, for example, reduced epidermal thickness, flattening of dermo epidermal junction [5] and the reduction of fibrillar collagen content [6–9]. In young skin, thick collagen fiber bundles are present with little open space. Fibroblasts appear orientated along collagen bundles. In old skin, collagen fibers are more disorientated with present empty space and fibroblasts show little orientation along fibroblast bundles [7]. Additionally, aged skin contains increased amount of fragmented collagen [9]. Also, the *in vitro* growth capacity of aged fibroblasts is reduced [5, 7].

#### Homeostatis of dermal extracellular matrix

Fibroblasts produce the dermal collagen matrix consisting of 80–90% of Type I collagen and 10–15% Type III collagen. For both types of collagen, a linear age-related decrease of 29% over a 49-year period in cultured fibroblasts has been reported [10]. Gene expression of Type I procollagen has shown to be reduced by 75% in fibroblasts from direct dermis extracts [7, 8].

The content of matrix metalloproteinase 1 (MMP1) is elevated in aged upper dermis and in aged fibroblasts [11]. Additionally, an increased content of fragmented collagen, the product of collagen degradation by MMP1 can be found in aged skin [12].

#### Regulation of collagen production by TGF-*β*

Type I procollagen production is mainly regulated by the *transforming growth factor beta* (TGF-*β*) / SMAD signalling pathway [8]. Connective Tissue Growth Factor (CTFG) is an important downstream regulator of TGF-*β*/ R-SMAD mediated reduction of collagen and levels of mRNA TGF-*β*1 and CTGF decrease by 70% and 57% respectively in *in vivo* aged dermal fibroblasts [8]. Regulation of Type I collagen expression by TGF-*β* is mediated via Type II TGF-*β* receptor (T*β*RII) and SMAD3 in dermal fibroblasts [13] and expression of SMAD3 is reduced in aged skin [14]. A universal negative feedback regulation mechanism of TGF-*β* signalling is mediated via SMAD7 [15] which has also been shown to be active in dermal fibroblasts [8]. Age-related dermal reduction of collagen content therefore is a consequence of concomitant diminished expression of TGF-*β*, SMAD and CTGF in dermal fibroblasts.

#### In vivo interaction of dermal fibroblasts with extracellular matrix

Extracellular mechanical forces, mediated by Integrin receptors (e.g. Integrin *α*2*β*1) induce prominent alterations of cellular function (e.g. gene expression) in dermal fibroblasts [11, 16]. Presence of fragmented collagen increases Integrin *α*2*β*1 mRNA which in turn causes strong up-regulation of MMP-1 mRNA in dermal fibroblasts. Additionally, presence of reactive oxygen species (ROS) is markedly increased by contact with extracellular fragmented collagen. Based on these findings, a model of self-perpetuating age dependent collagen fragmentation has been proposed [11].

Increased ROS also down-regulate TGFBR2 (TGF-*β* receptor II) thereby impairing the TGF-*β*/ R-SMAD pathway [13]. In aged skin, dermal fibroblasts have lower cellular contact area to collagen fibers resulting in less mechanical stimulation [7]. Therefore, *in vivo* ageing effects of dermal fibroblasts depend on at least two different mechanisms:

- Cell intrinsic senescence
- Altered mechanical interaction with extracellular matrix

#### Genes associated with known cellular alterations

Known alterations associated with ageing skin as well as cellular senescence mechanisms draw attention to genes evidently related therewith. For skin, known gene groups with altered expression include collagens, metalloproteinases and TGF-*β* signalling pathway associated genes. With cellular senescence, functionally associated genes are TGF-*β* signalling pathway and cyclin dependent kinases [17]. Additionally, for senescent cells, commonly utilised reporter genes are p16^INK4a^(CDKN2A) and p21^Cip1^ (CDKN1A, WAF1) [18]. The indicator SA-*β*gal is mainly used for histochemical detection [19, 20]. Also, proteins secreted as part of Senescence-Associated Secretory Phenotype (SASP), for example, interleukins, proteinases or some chemokines are of interest [19, 21, 22].

#### Photoageing

Photoageing, the most important causative of extrinsic skin ageing [6], is mainly caused by UVA (320–400 nm) exposition [23, 24]. Characteristic for UV aged skin are deep wrinkles and reduced stiffness [6].

In UV radiated skin various molecular effects are detected, for example, reduced TGF-*β* signalling causing diminished collagen production [25].

#### Principle findings

We describe that consistent age-related alterations in gene expression are not detectable in short term cultured fibroblasts. Using an analysis approach based on monotone alignment depth ratios, we filtered out 42 genes with consistently increasing or deceasing alignment depth. In a subset of 9 TGF-*β* signalling related genes (ATOH8, SNAI1, ID3, SPHK1, ID1, PRRX2, SMAD7, FAM83G, SERTAD1) high pairwise correlated gene expression was found (correlation coefficients >0.8).

## Materials and methods

### Sample donors

Sixty 4 mm punch biopsies of healthy skin were taken from 30 human donors. From each donor one sample was derived from a sun-exposed site (neck/shoulder) and one sample from a sun-protected (buttock/gluteal) site. Fifteen donors were female and fifteen donors were male. Samples were analysed histologically to verify presence of characteristic age-related or UV-exposition related alterations. Sample donors were assigned to three groups according to their age: Young (18–25 years), Middle (35–49 years) and Old (60–67 years). Six samples (two samples from each age group) were excluded from further analysis due for serious disturbing effects identified by analysis of DNA 6-mer spectra using the Bioconductor package seqTools [26]. Samples from 27 subjects (13 female and 14 male) were included into the analysis (in total 54 samples, 18 samples per age-group). The study was approved by the Ethical Committee of the Medical Faculty of the University of Düsseldorf (# 3361) in 2011. Informed written consent was obtained from all donors before sample acquisition.

### Fibroblast cultures

From each sample a part was fixed in 3.4% formaldehyde and prepared for histology using standard procedures. Isolation and primary culture of the cells were performed as previously described [27]: 4-mm punch biopsies were washed in 70% ethanol, followed by sterile phosphate-buffered saline. In brief, skin pieces were incubated with dispase (10 mg/ml in phosphate-buffered saline, sterile filtered) at 37°C and 5% CO_2_ for 2 hours to remove the epidermis. Dermal pieces were dried for 10 min under the sterile bench, then culture medium was added. Cells were propagated further at 37°C, 5% CO_2_ and in high glucose and L-glutamine containing Dulbecco’s modified Eagle’s medium (DMEM; Biochrom) supplemented with 10% fetal calf serum (FCS Superior; Biochrom) and 1% antibiotics (PAN Biotech) with the medium changed every 2–3 days. The fibroblasts were sub-cultured by treatment with 0.05% trypsin for 5 min. Mycoplasma and virus contamination was excluded by the Multiplex Cell Contamination Test (Multiplexion). Fibroblasts started to migrate out of the dermal pieces after approximately 1–2 weeks. Fibroblasts for RNA-seq analysis were taken from passages 2–7.

### RNA-sequencing

For alignment and all subsequent analysis, Human genomic sequence (GRCh38) and annotation data (release 82) were downloaded from Ensembl [28] and BioMart [29, 30]. cDNA libraries were synthesised using TruSeq RNA SamplePrep kit (Illumina) according to the manufacturer’s protocol. One microgram of total RNA was used for poly(A) RNA enrichment. The samples were amplificated on 9 Illumina flow cells (v1.5) and sequenced on a Illumina HiSeq 2000 sequencer using TruSeq SBS kits v1. From each lane, the resulting 101-nt sequence reads were converted to Fastq by CASAVA (1.8.2). Subsequent alignments were calculated on unprocessed Fastq files. Alignments were calculated using bowtie2 (2.2.5) [31], TopHat (2.0.14) [32] and STAR (2.4.1d modified) [33]. The raw Fastq files are available under ArrayExpress accession E-MTAB-4652 (ENA study ERP015294).

### Analysis of alignment counts

Analysis of alignment counts were executed using R / Bioconductor software [34]. Read counts were calculated from BAM files using two different approaches: *summarizeOverlaps* (GenomicAlignments) [35] and *readExpSet*(rbamtools) [36]. For standard differential expression (DE) we used Quasi-likelihood F-Tests from the edgeR (3.12.0) framework [37] (EQLF). P-values were corrected for multiple testing using Benjamini Hochberg procedure. Adjusted p-values (FDR levels) below 0.1 were considered significant. Analysis of gene set enrichment (GSEA) for GO terms and KEGG pathways was done using functions *topGO* and *topKEGG* from package *limma* (Bioconductor) [38]. For GESA, p-values (*P.DE*), below 0.05 were considered significant.

#### Estimation of mRNA expression level

Gene expression levels were estimated with normalised read counts derived from *summarizeOverlaps* (GenomicAlignments). The read counts are presented as counts-per-million (CPM) values. We do not use model-based CPM estimates (e.g. obtained using *cpm* (edgeR)) because we want demonstrate the variability of CPM values within groups.

#### Alignment gap-sites

Analysis of read counts in this study utilises our R/Bioconductor analysis pipeline specialised on identification of splicing events consisting of the R packages *rbamtools* [39], *refGenome* [40] and *spliceSites* [41]. The central surrogate of splicing events in read alignment data are *gap-sites*: Inner borders of gapped read alignments, sharing coordinates with all other reads covering the same splicing event. In this analysis, read numbers on gap-sites are used for identification of splicing events present in alignment data and for estimation of gene expression abundance.

#### Gene filtering using monotone alignment depth (AD) ratios (MALDR approach)

For identification of age-related differential gene expression, a sensible approach should focus on small differences which monotonically increase with proceeding age surrounded by considerable inter-individual variation. Besides standard differential expression (DE) procedures, we therefore implemented a filter method based on monotone alignment depth relations (MALDR). The initial step in this procedure uses read counts on gap-sites. Gap-sites identified from TopHat alignment data using *readExpSet* (spliceSites) were first filtered for presence of at least one read in each sample and then filtered for annotated splice sites in Ensembl 82. The method consists of the following steps:

- Count aligns on annotated gap-sites using *readExpSet* (spliceSites).
- Perform edgeR differential expression analysis (Quasi likelihood F-test) on gap-site alignment counts and on two age groups: Young and Old.
- Filter out genes for which DE analysis resulted in FDR below 0.1 on at least one splice site.
- Count alignment depth on complete genomic regions for all filtered genes and all samples.
- First cut of genomic regions: Intronic regions (defined by alignment gap sites) are removed from alignment counts resulting in a restricted genetic region.
- Combine alignment depth data for each group using mean and smooth mean group alignment depth data using loess regression.
- Second cut of genomic regions: Regions with low read coverage (below 2% of maximal alignment depth within gene) are removed from alignment counts.
- Determine regions with monotone alignment depth relation (Young<Middle<Old or Old<Middle<Young) in loess smoothed alignment depth data.
- Determine *fraction of restricted genetic region* where relation between all adjacent groups is >1.2.
- Genes, for which *fraction of restricted genetic region* is >99% of restricted genetic region are considered significantly differential expressed.

We present MALDR results in increasing *fraction of restricted genetic region* order containing no information on effect size or baseline expression level, a property shared with ordering along p-values. We utilised MALDR approach for a more sensible detection of age-related alterations in gene expression level.

#### Testing for differences in different qualities

The samples analyzed in this study are grouped by three donor qualities: Age, gender and sample location. As we expected to find small group differences on a small set of differential expressed genes, we conducted three parallel test procedures (one for each quality) where each test ignores a heterogeneity with respect to other qualities. With this strategy, we maintained the full sample size for each quality. The hypothesis of small numbers of differentially expressed genes was confirmed by the results of this study where the number of significant different expressed genes is below 3 ‰ in all comparisons.

## Results

### Histological analysis

**Fig 1:**
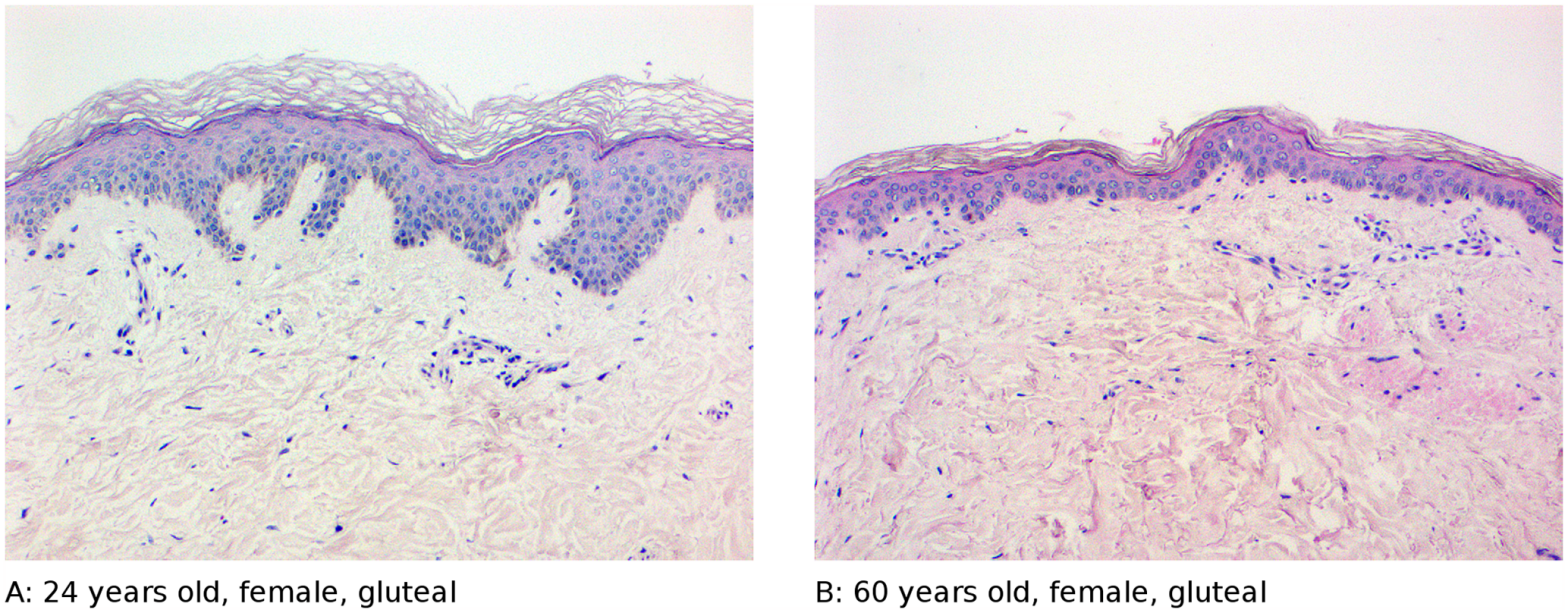
Representative histological sections from two female donors. **A**: Female donor, 24 years old (Young age group), gluteal (sun-protected) location. **B**: Female donor, 60 years old (Old age group), gluteal (sun-protected) location.

We show two representative histological images of biopsies from two female donors aged 24 and 60 years. Thereby we can show the characteristical age-dependent loss of rete ridges and thinning of the epidermis including a potentially reduced stratum corneum and a less dense dermal collagen structure (Fig 1). In addition, sun-exposed sites from the same donor (shoulder versus gluteal) are characterised by an increased number of melanocytes and in particular in the skin of the older donors an even more profound disturbance of the dermal collagen structure. More histological sections are shown in supplemental material (see S2 File).

### Analysis of age related differential gene expression

#### Standard differential expression analysis for age group

We performed differential expression analysis restricted to two age groups (Young and Old). Each group consists of 18 samples. Standard differential expression analysis of gene-wise read counts with EQLF (egdeR, Quasi-likelihood F-Test) resulted in *no* significant differential expressed gene (lowest FDR-value: 0.23).

#### Differential expression analysis for age group MALDR approach

We therefore re-evaluated the aligned reads using MALDR approach. From 54 samples, we identified 1,000,380 different unique gap-sites from which 100,040 reside on Ensembl 82 annotated splice sites. From testing number of alignments on each gap-site using edgeR QLF test we obtained 790 genes with at least one significant gap-site. Subsequent filtering using the beforehand described MALDR approach resulted in 42 genes with age dependent variation in gene expression. For these 42 genes, we examined alignment depth data:

- CPM values (as estimates for alteration of gene expression level) derived from *summarizeOverlaps* (shown in S3 File).
- Gene-wise maximum of *readExpSet* derived counts of gapped alignments crossing gap-sites (shown in S4 File).

Results from both methods have high similarity. They show that no consistent age dependent progress is present (in accordance with results from EQLF framework). Rather, the differences identified by MALDR base on different degrees of inter-individual variation in different age groups which is further illustrated in the following paragraph.

#### Exemplified results for gene ID1

**Fig 2:**
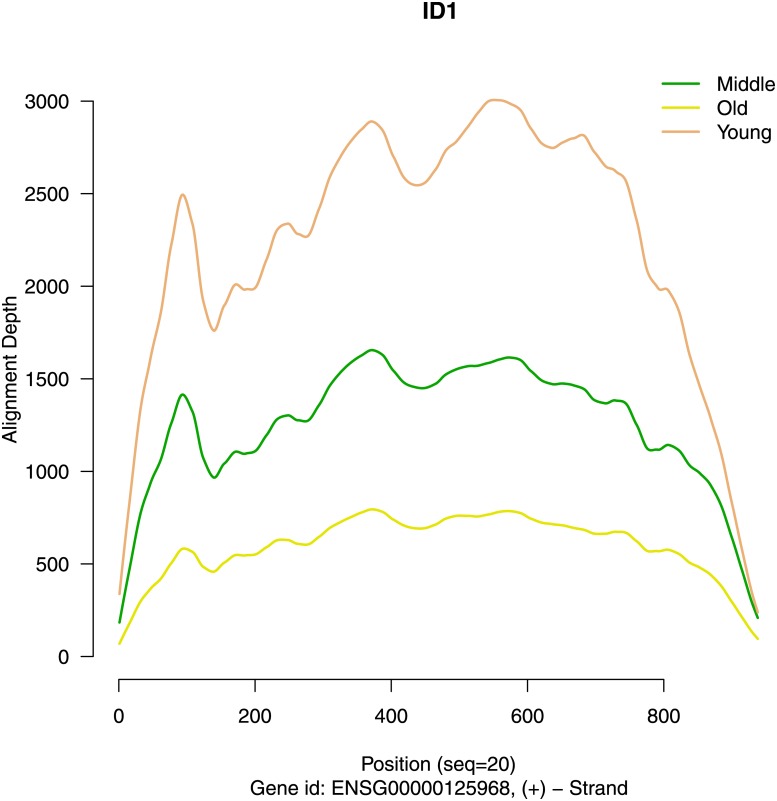
Align depth estimates for gene ID1. The figure displays alignment depth in absolute numbers. Three lines estimate mean alignment depth for each age group (y = Young, m = Middle, o = Old).

**Fig 3:**
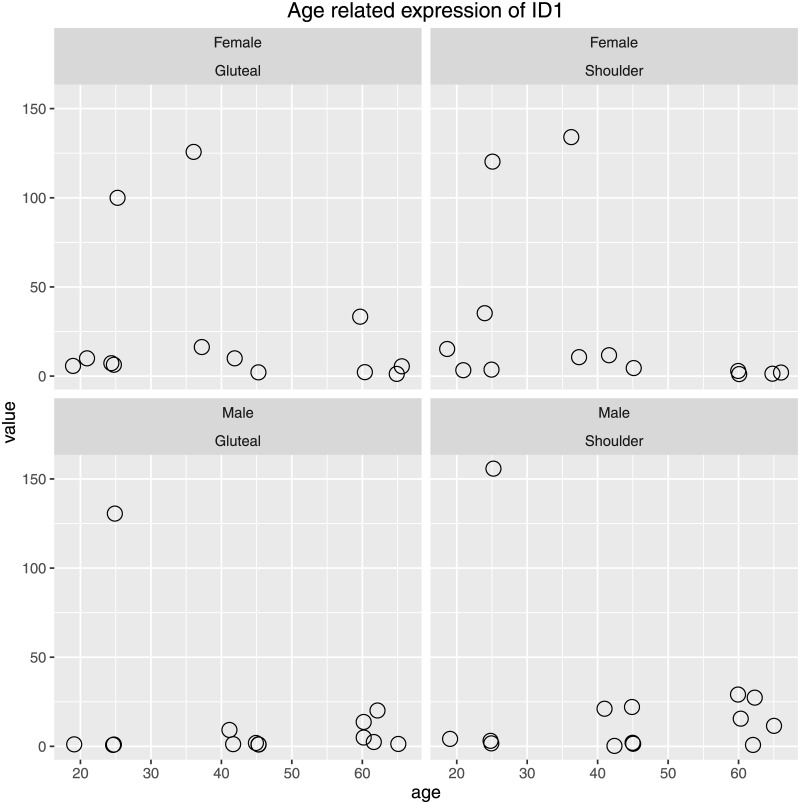
Gene-wise CPM values for ID1. Gene-wise *counts per million* (CPM) values derived from *summarizeOverlaps*.

We show the loess smoothed alignment depth data for gene ID1 (Inhibitor of DNA binding 1) after intronic and low coverage regions have been removed. A clear monotone ratio effect can be seen for age-group mean values of alignment depth (Fig 2). Plotting CPM values from individual samples (separated by gender and location) reveals that still for the majority of individuals, no systematic tendency is present (Fig 3). Obviously, the observed gene expression differences identified by MALDR are due to the presence of *three* out of 27 individuals (a male and a female at age of 25 and a female at age of 36) with markedly elevated transcript levels for ID1 (assigned to age groups Young and Middle). These three individuals generate a larger variation and increased mean values in the Young and Middle aged groups. We later on will explore, whether other genes with the same expression pattern can be identified in this data set and examine correlation coefficients for CPM values therefore.

#### MALDR derived result set

In order to differentiate the effect in MALDR filtered genes from a global, by differential expression analysis identified change we call these 42 genes *age MAR* (Monotone Alignment depth Ratio) genes. The complete table of *age MAR* genes is available in supplemental material S1 File. The number of *age MAR* genes is small compared to what has been reported for age related differential expression of genes in other tissues for example, in artery (3082 genes [4]) or blood (3287 genes [4] or 1497 genes [42]). But it is in the same order of magnitude as reported for skin (12 genes [4]) and dermal fibroblasts (104 genes [43]; In contrast, Glass et. al. describe 1672 differentially expressed genes in skin [44]).

Comparison *age MAR* genes from this study with age related differential expressed genes identified in the recently published GTEx study [4] resulted in *no* intersecting gene. Changing the FDR limit to 0.1 increased the number of *age MAR* genes in skin to 55 genes; we finally intersected our list herewith.

#### Functional characterisation of age MAR genes with GO and KEGG

Standard analysis for significantly enriched GO terms resulted in 609 terms (shown in S1 File). Thereof, only five GO terms were associated with fibroblast physiology (e.g. GO:0017134 fibroblast growth factor binding) or extracellular matrix (e.g. GO:0010715 regulation of extracellular matrix disassembly). Analysis for enriched KEGG pathways resulted in 12 enriched terms (shown in Table 1). Only for TGF-*β* signalling (path:hsa04350) and Rap1 signalling pathway (path:hsa04015) a relation to fibroblast physiology is plausible. The other associations are likely to be incidental.

**Table 1:** Enriched KEGG pathways in age MAR genes.

Pathway	N	Age MAR genes
TGF-beta signaling pathway	67	ID1, ID3, SMAD7
Signaling pathways regulating pluripotency of stem cells	96	ID1, ID3
Hippo signaling pathway	112	ID1, SMAD7
Rap1 signaling pathway	137	FGF13, ID1
Metabolic pathways	905	(4 genes)
Pentose phosphate pathway	23	PRPS1
Propanoate metabolism	28	ACSS1
Arginine and proline metabolism	33	CKB
Sphingolipid metabolism	36	SPHK1
VEGF signaling pathway	44	SPHK1
Arrhythmogenic right ventricular cardiomyopathy	47	GJA1
Melanoma	47	FGF13

Pathway: Name of KEGG pathway, N: Total number of genes in pathway, Age MAR genes: Number of selected genes in pathway

#### Functional characterisation of age MAR genes based on literature

We therefore explored putative physiologic roles of the 42 *age MAR* genes directly from literature by searching PubMed and online resources (HGNC, http://www.genenames.org/ and GeneCards, http://www.genecards.org/). Outlines of functionality with reference to literature is listed for each *age MAR* gene in the supplementary material (see S2 File). For every gene, we determined whether association with cellular senescence, cancer progression (or cell cycling), embryonal development (or tissue differentiaton), is described in the literature because of the close functional relationships between senescence, tissue differentiation, proliferation and cancer. The categories and associations of *age MAR* genes are shown in Table 2.

**Table 2:** Functional characterisation of age MAR genes.

Function	Associated genes
Cellular senescence (11 genes)	ATOH8, ID3, ID1, ERRFI1, MEG3, STC1, HSPB7, SMAD7, FAM83G, CKB, SERTAD1
Cancer progression / Cell cycle (20 genes)	PODXL, SNAI1, ID3, SPHK1, ID1, ERRFI1, SEPT5, MEG3, STC1, PRRX2, SMAD7, FAM83G, DDR1, EVA1A, FGFRL1, FILIP1L, ENC1, SERTAD1, ADGRL4, KCNC4
Differential expressed on cancer (11 genes	PODXL, SPHK1, ERRFI1, MEG3, PRRX2, DDR1, EVA1A, FILIP1L, ENC1, ROBO1, KCNC4
Development / Differentiation (10 genes)	ATOH8, PODXL, SNAI1, ID3, ID1, TRNP1, PRRX2, FAM83G, ENC1, SERTAD1
Cell migration / Adhesion (5 genes)	PODXL, ERRFI1, DDR1, FILIP1L, ROBO1
Cellular filaments / Skeleton (4 genes)	PODXL, SEPT5, HSPB7, ENC1
Vascularisation / Endothel (5 genes)	PODXL, FILIP1L, ADGRL4, ROBO1, KCNC4
Apoptosis / Autophagy (6 genes)	SPHK1, HSPB7, PRRX2, EVA1A, FILIP1L, GJA1

Functional associations of *age MAR* genes based on available literature. Genes possibly associate with multiple categories.

#### Correlation of gene expression between functional related genes

From our list of 42 *age MAR* genes, 8 genes (ATOH8, SNAI1, ID3, SPHK1, ID1, CNN1, PRRX2, ROBO1) are regulated by TGF-*β*. SMAD7 is part of TGF-*β* signalling and two genes (FAM83G, SERTAD1) interact with SMAD proteins. lncRNA MEG3 regulates TGF-*β* pathway genes. Altogether, TGF-*β* signal related genes comprise more than 25% of our *age MAR* genes. Analysis of correlation coefficients of *age MAR* gene CPM values revealed that for a set of 9 genes (ATOH8, SNAI1, ID3, SPHK1, ID1, PRRX2, SMAD7, FAM83G, SERTAD1), all pairwise correlation coefficients are greater than 0.8 (detailed analysis in supplemental material S2 File).

**Fig 4:**
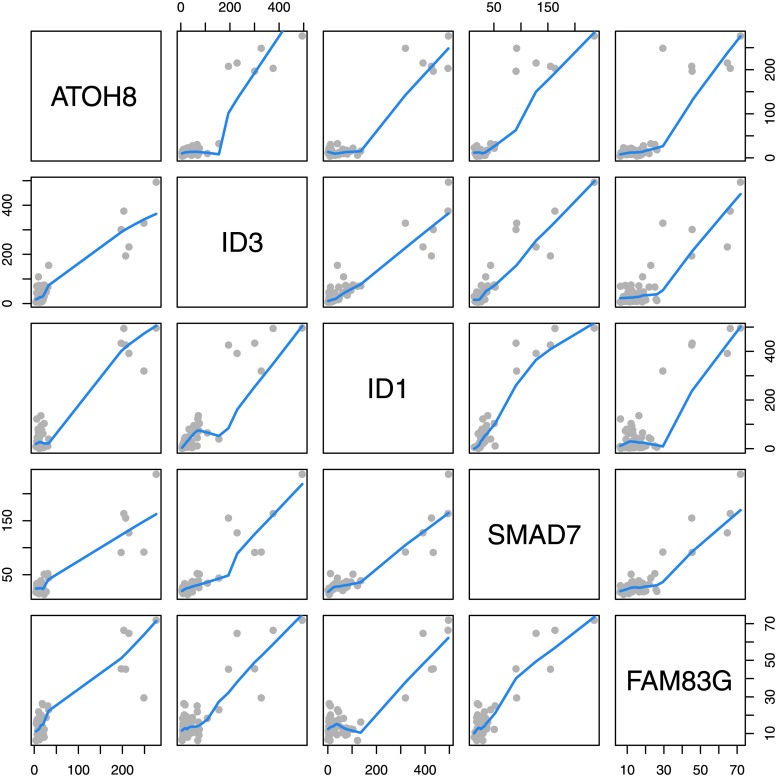
CPM values for five genes. CPM (counts per million) values derived from *summarizeOverlaps* for Genes ATOH8, ID3, ID1, SMAD7 and FAM83G for all 54 samples.

In five genes (ATOH8, ID3, ID1, SMAD7, FAM83G) gene expression in our samples show a similar expression pattern: CPM values (derived from *summarizeOverlaps*) for these genes are markedly elevated in the same three individuals (and they are therefore classified as *age MAR* genes). Fig 4 shows a panel of scatterplots of CPM values for genes ATOH8, ID3, ID1, SMAD7 and FAM83G revealing this relation. Highly correlated gene expression of SMAD7, ID1 and ATOH8 has indeed been described in mouse liver cells where the gene expression correlates with iron content of liver cells and is regulated by BMP6 [45].

### Analysis of gender and location related differential gene expression

Due to absence of age-related differential expressed genes in EQRF analysis, we regard the population as homogeneous with respect to donor age and age group and analysed differential expression of genes for gender and sample location (UV exposition) using EQLF.

#### Analysis of gender related DE genes

**Fig 5:**
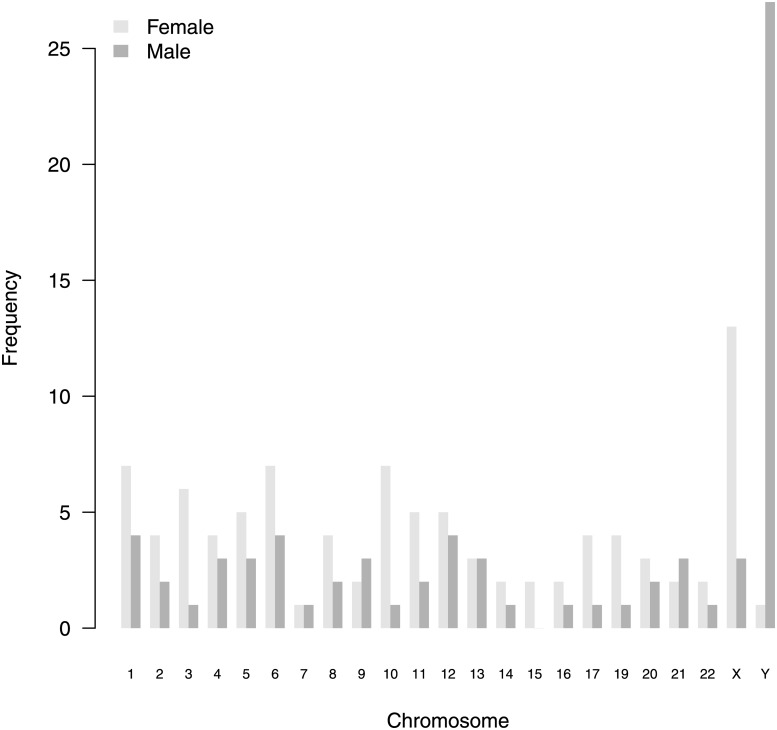
Chromosomal distribution of gender DE genes. Raw Number of significant gender DE genes per chromosome. On chromosome 18, no gene was differential expressed.

We analysed differential expression between samples derived from female and male sample donors (sex biased gene expression). Testing with EQLF revealed 168 gender related differential expressed genes (gender DE genes). The complete gene list is contained in S1 File (Gender_EQLF_DE_genes). Therefrom, 95 (57%) genes were higher expressed in male individuals (male-biased) and 73 (43%) genes were higher expressed in female individuals (female-biased). The chromosomal distribution of gender DE genes is shown in Fig 5. Twofold over-representation is restricted to Y chromosome for male-biased genes and is present on chromosomes 2, 10, 11, 15, 17, 19 and X for female-biased genes. Functional annotation with GO and KEGG reveals predominantly metabolic processes over-represented (e.g. GO:0006694 and path:hsa00100, both steroid biosynthesis).

#### Comparison of gender DE genes with other tissues

**Fig 6:**
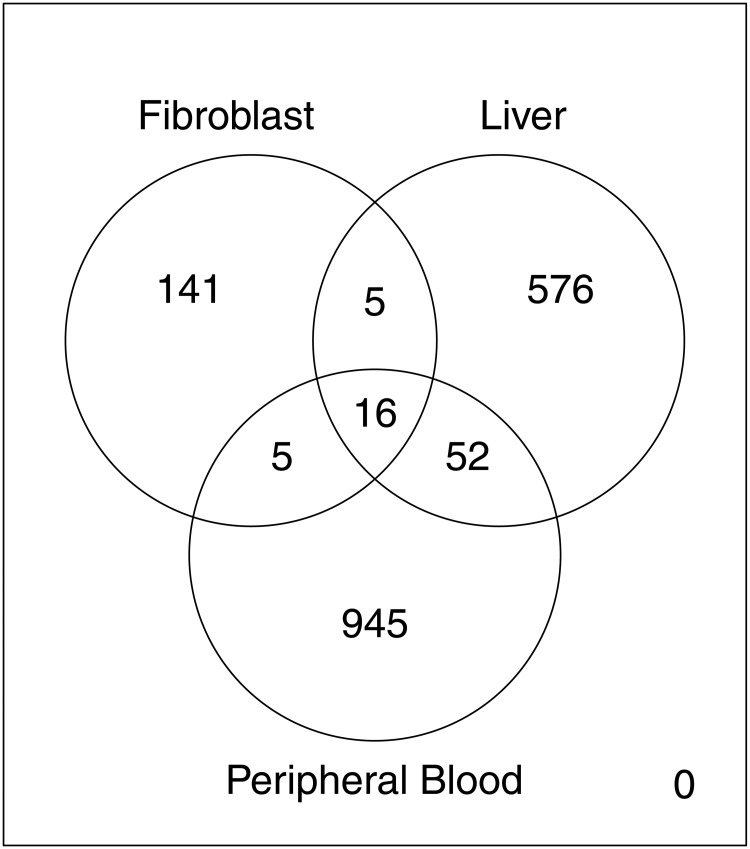
Number of gender related DE genes in different tissues. Comparison of gender related differential expressed genes. Gender DE genes in liver were described by [46]. Gender DE genes in peripheral blood were described by [47].

In order to compare the list of gender DE genes with results from other tissues, we downloaded lists of differential expressed genes from two mircroarray studies: A study on liver samples revealing 1,018 gender DE genes [46] and a study using peripheral blood samples revealing 649 gender DE genes. In Fig 6, a Venn diagram shows the number of shared genes between three tissues: fibroblasts, liver and peripheral blood. All study overlaps are below 10% of gender DE genes of each sample.

#### Analysis of location (UV exposition) related DE

From each donor, two samples were taken from different locations: One sample from the gluteal region (UV protected) and one sample from the shoulder (UV exposed). Differential expression analysis using EQLF results in 56 location related differential expressed (location DE) genes. The complete gene list is contained in S1 File (Location_EQLF_DE_genes).

### Analysis of gene expression with respect to fibroblast physiology

#### Collagen Type I gene expression

**Fig 7:**
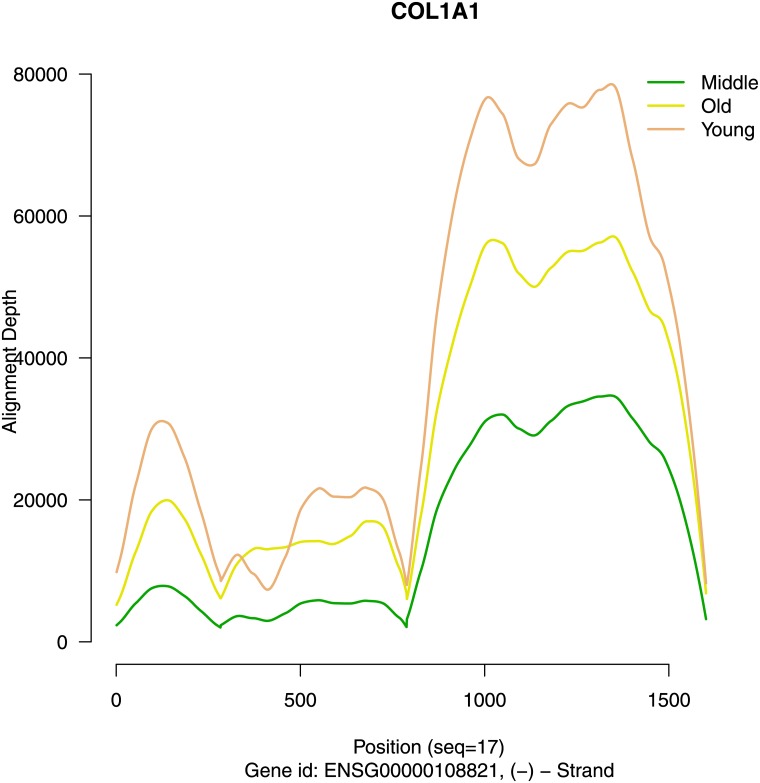
Alignment depth for gene COL1A1. Align depth in genetic region COL1A1 after cutting out intronic regions and regions with low alignment depth. Group-wise mean alignment depth values have been smoothed using loess regression.

**Fig 8:**
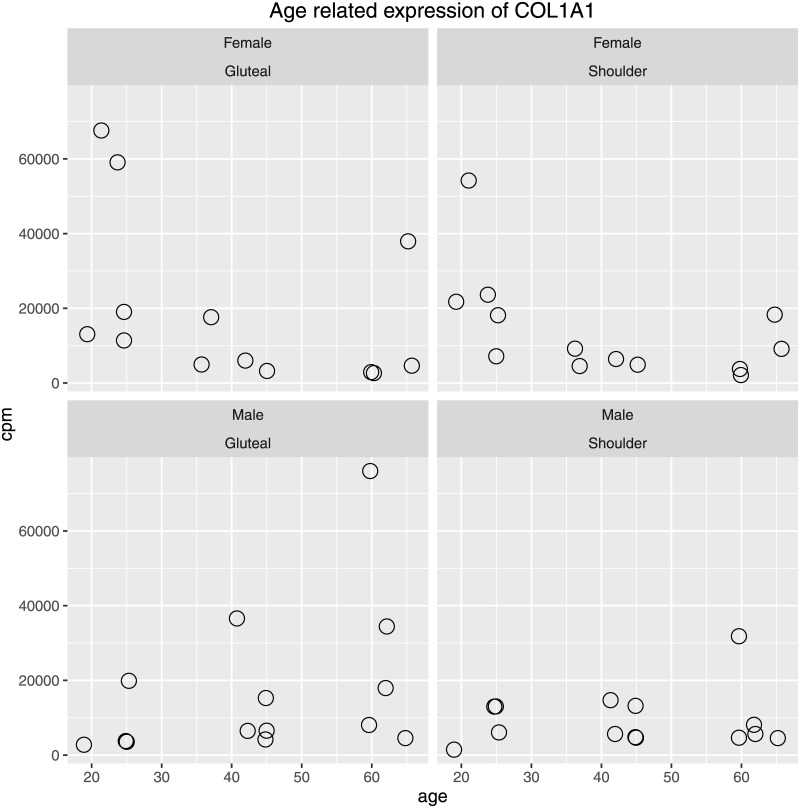
CPM values for gene COL1A1. Gene-wise counts per million (CPM) values directly derived from *summarizeOverlaps*.

From collagen Type I it is known, that *in vivo* gene expression of dermal fibroblasts progressively decreases with age. Therefore, the fact that in our samples, collagen Type I is not identified as differential expressed by EQLF and not filtered by MALDR approach probably requires further exploration. Age-group-wise alignment depth relations as shown in Fig 7 indicate, that collagen gene expression consistently is lowest in the Middle aged group explaining, that monotone alignment depth relations are absent for this gene. Alignment count values (CPM) show how this age group relation arises. Fig 8 shows that individuals expressing high amounts of COL1A1 (CPM >25,000) follow different patterns in female and male subjects. For male subjects, all COL1A1 high expressing individuals are >40 years old. For female subjects, one participant is 65 years old and all other are less than 30 years old.

#### Genes associated with cellular senescence

We tabulated gene expression level (logCPM) and changes in gene expression (logFC) for genes known to be associated with cellular senescence and SASP (see S1 File). Although, for some genes logFC values considerable different from 1 are present (-0.7 for TBX3 or 2.75 for CCL13) though changes are not significant (all FDR values are 1).

## Discussion

### Known effects of in vivo aged fibroblasts

In our data, consistent age-related changes gene expression for COL1A1, TGFB1, CTGF (CCN2) and MMP1 are absent. Therefore, the major known age-related physiological alterations in *in vivo* aged dermal fibroblasts are not present in our data. The apparently arising conflict is resolved by the fact, that changed gene expression for these genes have been shown to be undetectable in cultured dermal fibroblasts. For example, gene expression of procollagen Type I, TGF-*β* and CTGF does not differ between *in vivo* young and aged dermal fibroblasts [8]. Expression of MMP-1 is increased in aged dermal fibroblasts *in vivo*, but no difference has been observed in untreated cultured fibroblasts [11].

Fibroblasts interact very intensively with the extracellular fibrillar matrix (via integrin receptors) [7, 11] and physical forces regulate many important biological processes. Reduced mechanical forces down regulate TGFBR2 thereby impairing the TGF-*β*/ R-SMAD pathway. As consequence gene expression for Type I collagen, fibronectin and CTGF is reduced [48]. Removal of fibroblasts from tissue environment into cell culture is therefore likely to have profound impact on regulatory networks.

### Gene groups in differential expressed genes

We filtered out 42 age-related differential expressed genes using MALDR approach. Therefrom, a subset of 8 genes show highly correlated gene expression all of which are functionally related to TGF-*β* signalling pathway. The conclusions drawn from this observation are weakened by the fact that identified age-related alteration is mainly due to high expression of these genes in few individuals.

### Effects of photoageing

UV radiation induces a variety of changes in gene expression in the skin. Expression of Type I procollagen is reduces in human dermis and this reduction is mediated via TGF-*β* signalling pathway [25]. mRNA levels of JUN (c-jun) and FOS (c-Fos), members of transcription factor AP-1 family, are increased while the gene expression of SMAD 7 is up-regulated [12, 49]. Expression of matrix metalloproteinases (MMPs) is increased [50, 51]. These effects are not reflected in our location DE genes, implicating that chronic effects of UV radiation may be different from acute effects.

### Common age-related effects on gene expression

There are numerous studies on age-related physiological alterations and especially on gene expression. Age differences in gene expression show large variation between tissues as well as between species [4, 52].

#### Consistently age-related differential expressed genes

A central objective for high-throughput studies on samples from differently aged individuals is whether a global (i.e. not tissue specific) age related gene expression signature can be identified. Although there have been results suggesting that these gene sets may exist [53], already early studies reported that no such gene sets are present [54]. Results by Glass et. al. [44] suggest the same conclusion (by finding only one common *age MAR* gene in three tissues).

#### Functional enrichment analysis

Functional enrichment analysis of differential expressed genes commonly identifies mitochondrial pathways, RNA metabolism, and immune response pathways [4, 42, 53].

Enrichment of these pathways can not be concluded from our data. KEGG pathway analysis on our data is based on a very small set of genes (i.e. one gene for most pathways) and therefore statistical results are not reliable.

#### Experimental validation

The functional background of correlated gene expression in five genes (ATOH8, ID3, ID1, SMAD7, FAM83G) related to cellular senescence could further be explored using gene knockdown experiments, for example in the TGF-*β* pathway. As recently has been shown that reduction of mechanical forces leads to downregulation of TGF-*β* pathway [48], further cultivation likely reproduces the effect of cultivation and results could not directly be related to skin ageing.

A second question is whether the results from this transcriptomic study translate into protein levels especially when possibly insufficient correlations are considered. In a recent study, it was shown that predictability of protein levels from transcript level could be significantly enhanced when gene-specific RNA-to-protein (RTP) conversion factors are used leading to median pearson correlation coefficients of 0.93 [55]. As gene-specific RTP factors would not influence the results of gene-wise testing, it can be assumed that the major conclusions of this study also apply to protein levels.

### Concluding remarks

#### Ageing from evolutionary theory perspective

Evolutionary mechanisms select out morbidity and mortality before reproductive (and upbringing) ages but not beyond (selection shadow) [56]. Since ageing is not under evolutionary selection [57], single genes propagating (or preventing) ageing are unlikely to exist [56]. In contrast, since absence of selection evokes variation, a spectrum of ageing phenotypes with considerable inter- and intra-individual discrepancy can be expected. Human lifespan, a related property, is a complex trait where associated genomic loci have small effect size [58–60].

A component of evolutionary ageing models is *antagonistic pleiotropy*: Properties with beneficial effects in early life (which are under selection) and late detrimental effects (in the selection shadow) [56]. The tight connection between cellular differentiation, senescence, cancer and ageing now provides a model for antagonistic pleiotropy where cellular senescence is essential for embryonic tissue differentiation and stress response [61] but deleterious when organisms age or when cancer cells escape from senescence (a model which similarly has already been proposed [62, 63]).

## Conclusion

### Limitations of current study

#### Sample size

There is considerable variation in biological ageing processes, between individuals, tissues (and species). For capturing systematic effects as well as underlying variations, large sample sizes are needed [64]. Early size estimates were in the range of 16–63 samples per group [54, 64]. As tissue ageing appears gradually over time, experimental settings should be able to detect small fold changes (>2 [64]) which further increases sample size requirements [65]. Therefore, this study is under-powered, especially regarding two additional sources of variation (apart from age: Gender and UV exposition).

## Supporting information

S1 File Gene tables. Spreadsheet file containing the following tables:
Age_MALDR_DE_genes: Listed age-related differential expressed (*age MAR*) genes.
Age_MALDR_GO: Listed enriched GO terms for *age MAR* genes.
Age_MALDR_KEGG: Listed enriched KEGG pathways for *age MAR* genes.
Gender_EQLF_DE_genes: Listed gender related differential expressed (gender DE) genes.
Gender_EQLF_GO: Listed enriched GO terms for gender DE genes.
Gender_EQLF_KEGG: Listed enriched KEGG pathways for gender DE genes.
Location_EQLF_DE_genes: Listed location related differential expressed (gender DE) genes.
Location_EQLF_GO: Listed enriched GO terms for location DE genes.
Location_EQLF_KEGG: Listed enriched KEGG pathways for location DE genes.
Literature_selected_genes: Listed DE values for genes associated with senescence or fibroblast ageing.
(XLSX)

S2 File Supplemental material. Background information on methodology and additional results.
(pdf)

S3 File Age MAR CPM values. PDF file with gene expression data for the 42 age-related DE genes (derived from edgeR QLF test and ReadExpSet).
(pdf)

S4 File Age MAR CPM values. PDF file with *summarizeOverlaps* derived CPM data for the 42 age-related DE genes.
(pdf)

## References

[1] Lopez-Otin C, Blasco MA, Partridge L, Serrano M, Kroemer G. The hallmarks of aging. Cell. 2013;153(6):1194–1217. 10.1016/j.cell.2013.05.039 23746838 PMC3836174

[2] Mortazavi A, Williams BA, McCue K, Schaeffer L, Wold B. Mapping and quantifying mammalian transcriptomes by RNA-Seq. Nat Methods. 2008;5(7):621–628. 10.1038/nmeth.1226 18516045 PMC13303166

[3] Mele M, Ferreira PG, Reverter F, DeLuca DS, Monlong J, Sammeth M, et al Human genomics. The human transcriptome across tissues and individuals. Science. 2015;348(6235):660–665. 10.1126/science.aaa0355 25954002 PMC4547472

[4] Yang J, Huang T, Petralia F, Long Q, Zhang B, Argmann C, et al Synchronized age-related gene expression changes across multiple tissues in human and the link to complex diseases. Sci Rep. 2015;5:15145 10.1038/srep15145 26477495 PMC4609956

[5] Mine S, Fortunel NO, Pageon H, Asselineau D. Aging alters functionally human dermal papillary fibroblasts but not reticular fibroblasts: a new view of skin morphogenesis and aging. PLoS ONE. 2008;3(12):e4066 10.1371/journal.pone.0004066 19115004 PMC2605251

[6] Tobin DJ. Introduction to skin aging. J Tissue Viability. 2016;. 27020864 10.1016/j.jtv.2016.03.002

[7] Varani J, Dame MK, Rittie L, Fligiel SE, Kang S, Fisher GJ, et al Decreased collagen production in chronologically aged skin: roles of age-dependent alteration in fibroblast function and defective mechanical stimulation. Am J Pathol. 2006;168(6):1861–1868. 10.2353/ajpath.2006.051302 16723701 PMC1606623

[8] Quan T, Shao Y, He T, Voorhees JJ, Fisher GJ. Reduced expression of connective tissue growth factor (CTGF/CCN2) mediates collagen loss in chronologically aged human skin. J Invest Dermatol. 2010;130(2):415–424. 10.1038/jid.2009.224 19641518 PMC2877594

[9] Fligiel SE, Varani J, Datta SC, Kang S, Fisher GJ, Voorhees JJ. Collagen degradation in aged/photodamaged skin in vivo and after exposure to matrix metalloproteinase-1 in vitro. J Invest Dermatol. 2003;120(5):842–848. 10.1046/j.1523-1747.2003.12148.x 12713591

[10] Dumas M, Chaudagne C, Bonte F, Meybeck A. In vitro biosynthesis of type I and III collagens by human dermal fibroblasts from donors of increasing age. Mech Ageing Dev. 1994;73(3):179–187. 10.1016/0047-6374(94)90050-7 8057688

[11] Fisher GJ, Quan T, Purohit T, Shao Y, Cho MK, He T, et al Collagen fragmentation promotes oxidative stress and elevates matrix metalloproteinase-1 in fibroblasts in aged human skin. Am J Pathol. 2009;174(1):101–114. 10.2353/ajpath.2009.080599 19116368 PMC2631323

[12] Fisher GJ, Kang S, Varani J, Bata-Csorgo Z, Wan Y, Datta S, et al Mechanisms of photoaging and chronological skin aging. Arch Dermatol. 2002;138(11):1462–1470. 10.1001/archderm.138.11.1462 12437452

[13] He T, Quan T, Shao Y, Voorhees JJ, Fisher GJ. Oxidative exposure impairs TGF-beta pathway via reduction of type II receptor and SMAD3 in human skin fibroblasts. Age (Dordr). 2014;36(3):9623 10.1007/s11357-014-9623-6 24550076 PMC4082581

[14] Purohit T, He T, Qin Z, Li T, Fisher GJ, Yan Y, et al Smad3-dependent regulation of type I collagen in human dermal fibroblasts: Impact on human skin connective tissue aging. J Dermatol Sci. 2016;. 10.1016/j.jdermsci.2016.04.004 27132061

[15] Macias MJ, Martin-Malpartida P, Massague J. Structural determinants of Smad function in TGF-beta signaling. Trends Biochem Sci. 2015;40(6):296–308. 10.1016/j.tibs.2015.03.012 25935112 PMC4485443

[16] Eckes B, Zweers MC, Zhang ZG, Hallinger R, Mauch C, Aumailley M, et al Mechanical tension and integrin alpha 2 beta 1 regulate fibroblast functions. J Investig Dermatol Symp Proc. 2006;11(1):66–72. 10.1038/sj.jidsymp.5650003 17069012

[17] Brun C, Jean-Louis F, Oddos T, Bagot M, Bensussan A, Michel L. Phenotypic and functional changes in dermal primary fibroblasts isolated from intrinsically aged human skin. Exp Dermatol. 2016;25(2):113–119. 10.1111/exd.12874 26441147

[18] Childs BG, Durik M, Baker DJ, van Deursen JM. Cellular senescence in aging and age-related disease: from mechanisms to therapy. Nat Med. 2015;21(12):1424–1435. 10.1038/nm.4000 26646499 PMC4748967

[19] Munoz-Espin D, Serrano M. Cellular senescence: from physiology to pathology. Nat Rev Mol Cell Biol. 2014;15(7):482–496. 10.1038/nrm3823 24954210

[20] Dimri GP, Lee X, Basile G, Acosta M, Scott G, Roskelley C, et al A biomarker that identifies senescent human cells in culture and in aging skin in vivo. Proc Natl Acad Sci USA. 1995;92(20):9363–9367. 10.1073/pnas.92.20.9363 7568133 PMC40985

[21] Freund A, Orjalo AV, Desprez PY, Campisi J. Inflammatory networks during cellular senescence: causes and consequences. Trends Mol Med. 2010;16(5):238–246. 10.1016/j.molmed.2010.03.003 20444648 PMC2879478

[22] Coppe JP, Desprez PY, Krtolica A, Campisi J. The senescence-associated secretory phenotype: the dark side of tumor suppression. Annu Rev Pathol. 2010;5:99–118. 10.1146/annurev-pathol-121808-102144 20078217 PMC4166495

[23] Battie C, Jitsukawa S, Bernerd F, Del Bino S, Marionnet C, Verschoore M. New insights in photoaging, UVA induced damage and skin types. Exp Dermatol. 2014;23 Suppl 1:7–12. 10.1111/exd.12388 25234829

[24] Holick MF. Biological Effects of Sunlight, Ultraviolet Radiation, Visible Light, Infrared Radiation and Vitamin D for Health. Anticancer Res. 2016;36(3):1345–1356. 26977036

[25] Quan T, He T, Kang S, Voorhees JJ, Fisher GJ. Connective tissue growth factor: expression in human skin in vivo and inhibition by ultraviolet irradiation. J Invest Dermatol. 2002;118(3):402–408. 10.1046/j.0022-202x.2001.01678.x 11874477

[26] Kaisers W, Schwender H, Schaal H. Hierarchical clustering of DNA k-mer counts in RNA-seq fastq files reveals batch effects. arXiv. 2014;arXiv:1405.0114. 10.3390/ijms19113687 PMC6274891 30469355

[27] Tigges J, Weighardt H, Wolff S, Gotz C, Forster I, Kohne Z, et al Aryl hydrocarbon receptor repressor (AhRR) function revisited: repression of CYP1 activity in human skin fibroblasts is not related to AhRR expression. J Invest Dermatol. 2013;133(1):87–96. 10.1038/jid.2012.259 22951721

[28] Cunningham F, Amode MR, Barrell D, Beal K, Billis K, Brent S, et al Ensembl 2015. Nucleic Acids Res. 2015;43(Database issue):D662–669. 10.1093/nar/gku1010 25352552 PMC4383879

[29] Guberman JM, Ai J, Arnaiz O, Baran J, Blake A, Baldock R, et al BioMart Central Portal: an open database network for the biological community. Database (Oxford). 2011;2011:bar041 10.1093/database/bar041 21930507 PMC3263598

[30] Durinck S, Moreau Y, Kasprzyk A, Davis S, De Moor B, Brazma A, et al BioMart and Bioconductor: a powerful link between biological databases and microarray data analysis. Bioinformatics. 2005;21(16):3439–3440. 10.1093/bioinformatics/bti525 16082012

[31] Langmead B, Salzberg SL. Fast gapped-read alignment with Bowtie 2. Nat Methods. 2012;9(4):357–359. 10.1038/nmeth.1923 22388286 PMC3322381

[32] Trapnell C, Pachter L, Salzberg SL. TopHat: discovering splice junctions with RNA-Seq. Bioinformatics. 2009;25(9):1105–1111. 10.1093/bioinformatics/btp120 19289445 PMC2672628

[33] Dobin A, Davis CA, Schlesinger F, Drenkow J, Zaleski C, Jha S, et al STAR: ultrafast universal RNA-seq aligner. Bioinformatics. 2013;29(1):15–21. 10.1093/bioinformatics/bts635 23104886 PMC3530905

[34] Huber W, Carey VJ, Gentleman R, Anders S, Carlson M, Carvalho BS, et al Orchestrating high-throughput genomic analysis with Bioconductor. Nat Methods. 2015;12(2):115–121. 10.1038/nmeth.3252 25633503 PMC4509590

[35] Lawrence M, Huber W, Pages H, Aboyoun P, Carlson M, Gentleman R, et al Software for computing and annotating genomic ranges. PLoS Comput Biol. 2013;9(8):e1003118 10.1371/journal.pcbi.1003118 23950696 PMC3738458

[36] Kaisers W, Schaal H, Schwender H. rbamtools: an R interface to samtools enabling fast accumulative tabulation of splicing events over multiple RNA-seq samples. Bioinformatics. 2015;31(10):1663–1664. 10.1093/bioinformatics/btu846 25563331

[37] Robinson MD, McCarthy DJ, Smyth GK. edgeR: a Bioconductor package for differential expression analysis of digital gene expression data. Bioinformatics. 2010;26(1):139–140. 10.1093/bioinformatics/btp616 19910308 PMC2796818

[38] Ritchie ME, Phipson B, Wu D, Hu Y, Law CW, Shi W, et al limma powers differential expression analyses for RNA-sequencing and microarray studies. Nucleic Acids Res. 2015;43(7):e47 10.1093/nar/gkv007 25605792 PMC4402510

[39] Kaisers W. rbamtools: Read and Write BAM (Binary Alignment) Files; 2016. Available from: https://CRAN.R-project.org/package=rbamtools.

[40] Kaisers W. refGenome: Gene and Splice Site Annotation Using Annotation Data from Ensembl and UCSC Genome Browsers; 2016.

[41] Kaisers W. spliceSites: A bioconductor package for exploration of alignment gap positions from RNA-seq data; 2012.

[42] Peters MJ, Joehanes R, Pilling LC, Schurmann C, Conneely KN, Powell J, et al The transcriptional landscape of age in human peripheral blood. Nat Commun. 2015;6:8570 10.1038/ncomms9570 26490707 PMC4639797

[43] Dekker P, Gunn D, McBryan T, Dirks RW, van Heemst D, Lim FL, et al Microarray-based identification of age-dependent differences in gene expression of human dermal fibroblasts. Mech Ageing Dev. 2012;133(7):498–507. 10.1016/j.mad.2012.06.002 22721680

[44] Glass D, Vinuela A, Davies MN, Ramasamy A, Parts L, Knowles D, et al Gene expression changes with age in skin, adipose tissue, blood and brain. Genome Biol. 2013;14(7):R75 10.1186/gb-2013-14-7-r75 23889843 PMC4054017

[45] Kautz L, Meynard D, Monnier A, Darnaud V, Bouvet R, Wang RH, et al Iron regulates phosphorylation of Smad1/5/8 and gene expression of Bmp6, Smad7, Id1, and Atoh8 in the mouse liver. Blood. 2008;112(4):1503–1509. 10.1182/blood-2008-03-143354 18539898

[46] Zhang Y, Klein K, Sugathan A, Nassery N, Dombkowski A, Zanger UM, et al Transcriptional profiling of human liver identifies sex-biased genes associated with polygenic dyslipidemia and coronary artery disease. PLoS ONE. 2011;6(8):e23506 10.1371/journal.pone.0023506 21858147 PMC3155567

[47] Jansen R, Batista S, Brooks AI, Tischfield JA, Willemsen G, van Grootheest G, et al Sex differences in the human peripheral blood transcriptome. BMC Genomics. 2014;15:33 10.1186/1471-2164-15-33 24438232 PMC3904696

[48] Fisher GJ, Shao Y, He T, Qin Z, Perry D, Voorhees JJ, et al Reduction of fibroblast size/mechanical force down-regulates TGF-beta type II receptor: implications for human skin aging. Aging Cell. 2016;15(1):67–76. 10.1111/acel.12410 26780887 PMC4717276

[49] Quan T, He T, Voorhees JJ, Fisher GJ. Ultraviolet irradiation induces Smad7 via induction of transcription factor AP-1 in human skin fibroblasts. J Biol Chem. 2005;280(9):8079–8085. 10.1074/jbc.M409647200 15579469 PMC3738262

[50] Fisher GJ, Wang ZQ, Datta SC, Varani J, Kang S, Voorhees JJ. Pathophysiology of premature skin aging induced by ultraviolet light. N Engl J Med. 1997;337(20):1419–1428. 10.1056/NEJM199711133372003 9358139

[51] Pittayapruek P, Meephansan J, Prapapan O, Komine M, Ohtsuki M. Role of Matrix Metalloproteinases in Photoaging and Photocarcinogenesis. Int J Mol Sci. 2016;17(6). 10.3390/ijms17060868 27271600 PMC4926402

[52] Valdes AM, Glass D, Spector TD. Omics technologies and the study of human ageing. Nat Rev Genet. 2013;14(9):601–607. 23938363 10.1038/nrg3553

[53] de Magalhaes JP, Curado J, Church GM. Meta-analysis of age-related gene expression profiles identifies common signatures of aging. Bioinformatics. 2009;25(7):875–881. 10.1093/bioinformatics/btp073 19189975 PMC2732303

[54] Han E, Hilsenbeck SG. Array-based gene expression profiling to study aging. Mech Ageing Dev. 2001;122(10):999–1018. 10.1016/S0047-6374(01)00215-9 11389920

[55] Edfors F, Danielsson F, Hallstrom BM, Kall L, Lundberg E, Ponten F, et al Gene-specific correlation of RNA and protein levels in human cells and tissues. Mol Syst Biol. 2016;12(10):883 10.15252/msb.20167144 27951527 PMC5081484

[56] Kirkwood TB, Austad SN. Why do we age? Nature. 2000;408(6809):233–238. 10.1038/35041682 11089980

[57] Kirkwood TB, Melov S. On the programmed/non-programmed nature of ageing within the life history. Curr Biol. 2011;21(18):R701–707. 10.1016/j.cub.2011.07.020 21959160

[58] Zeng Y, Nie C, Min J, Liu X, Li M, Chen H, et al Novel loci and pathways significantly associated with longevity. Sci Rep. 2016;6:21243 10.1038/srep21243 26912274 PMC4766491

[59] Deelen J, Beekman M, Capri M, Franceschi C, Slagboom PE. Identifying the genomic determinants of aging and longevity in human population studies: progress and challenges. Bioessays. 2013;35(4):386–396. 10.1002/bies.201200148 23423909 PMC3633240

[60] Barzilai N, Guarente L, Kirkwood TB, Partridge L, Rando TA, Slagboom PE. The place of genetics in ageing research. Nat Rev Genet. 2012;13(8):589–594. 10.1038/nrg3290 22777128

[61] Childs BG, Baker DJ, Kirkland JL, Campisi J, van Deursen JM. Senescence and apoptosis: dueling or complementary cell fates? EMBO Rep. 2014;15(11):1139–1153. 10.15252/embr.201439245 25312810 PMC4253488

[62] Campisi J. Aging, tumor suppression and cancer: high wire-act! Mech Ageing Dev. 2005;126(1):51–58. 10.1016/j.mad.2004.09.024 15610762

[63] Hornsby PJ. Senescence and life span. Pflugers Arch. 2010;459(2):291–299. 10.1007/s00424-009-0723-6 19763609

[64] Passtoors WM, Beekman M, Gunn D, Boer JM, Heijmans BT, Westendorp RG, et al Genomic studies in ageing research: the need to integrate genetic and gene expression approaches. J Intern Med. 2008;263(2):153–166. 10.1111/j.1365-2796.2007.01904.x 18226093

[65] Schurch NJ, Schofield P, Gierlinski M, Cole C, Sherstnev A, Singh V, et al How many biological replicates are needed in an RNA-seq experiment and which differential expression tool should you use? RNA. 2016; p. 839–51. 10.1261/rna.053959.115 27022035 PMC4878611

