# Supplementary material for: Age, gender and UV-exposition related effects on gene expression in in vivo aged short term cultivated human dermal fibroblasts
Source: PLoS One. 2017 May 5;12(5):e0175657. doi: 10.1371/journal.pone.0175657 (PMC5419556; doi:10.1371/journal.pone.0175657)
Supplement: S2 File — Background information on methodology and additional results. (pdf) [file pone.0175657.s002.pdf]

Supplemental Material for: Age, Gender and UV-Exposition Related Effects on Gene Expression in Vivo Aged Short Term Cultivated Human Dermal Fibroblasts

Wolfgang Kaisers, CBiBs HHU Düsseldorf

December 7, 2016

Contents

1 Participants **2**

2 Histology **3**

3 Fastq reads and quality **4**
- 3.1 Global distribution of Phred scores 4
- 3.2 Nucleotide frequency in sequencing data 7

4 Reference Genome and annotation data **8**
- 4.1 Human DNA sequence 8
- 4.2 Annotation of genomic features 9

5 Analytic procedures **9**
- 5.1 Standard differential expression (DE) analysis: EQLF 9
- 5.2 Counting alignmnets using summarizeOverlaps 9
  - 5.2.1 Statistical testing for DE using edgeR 9
- 5.3 Differential expression analyis using MALDR approach 10
  - 5.3.1 Workflow 10
- 5.4 Estimation of gene expression abundance 12
  - 5.4.1 CPM values 13
  - 5.4.2 Gene filtering 13
  - 5.4.3 GO and KEGG pathway analysis 14

6 Additional results from DE analysis **15**
- 6.1 Analysis for age group 15
- 6.2 Results from DE analysis for age-group (Young vs. Middle vs. Old) 15
- 6.3 Analysis of correlation of CPM values 17
- 6.4 Correlation of CPM values between age related DE genes 21
- 6.5 Results from DE analysis for gender (Female vs. Male) 21
- 6.6 Gender related differential expressed genes 21
  - 6.6.1 Results from EQLF analysis 21
  - 6.6.2 Functional annotation with GO terms 21
  - 6.6.3 Functional annotation with KEGG pathways 22
- 6.7 Results from DE analysis for location (Gluteal vs. Shoulder) 23
  - 6.7.1 Gene list 23
  - 6.7.2 Functional annotation with GO terms 23
  - 6.7.3 Functional annotation with KEGG pathways 23

7 Fibroblast physiology **25**
7.1 Age related DE genes in literature 30
7.2 Results from expression analysis for high expressed genes 35

8 Comparison of DE test procedures **35**

9 Functional characterization of age VAR genes based on literature **36**

10 Further information on supplementary material **44**

1 Participants

In this study, we present RNA-seq data from human dermal fibroblasts derived from 54 samples. From each donor, two samples were obtained. Therefore, samples from 27 donors are included into the study. Table 1 displays assignment of donors to Age groups and Fig 1 shows distribution of donor age.

Age group	Frequency
Young	18
Middle	18
Old	18

Table 1: Group assignment of sample donors

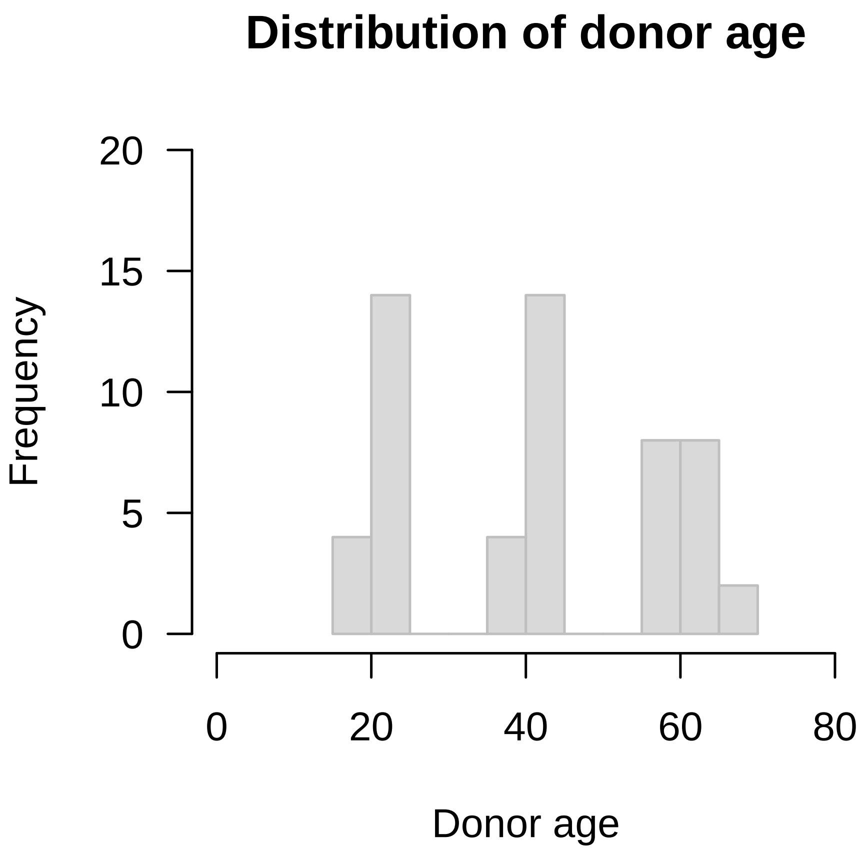

Fig 1: Distribution of sample donor age

2 Histology

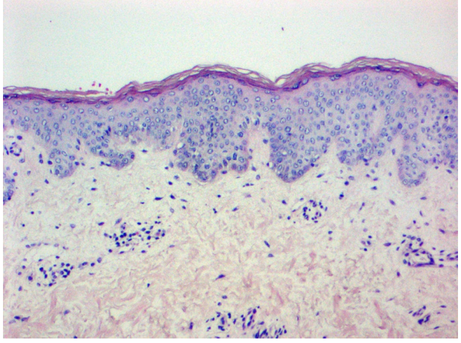

A: 25 years old, male, gluteal

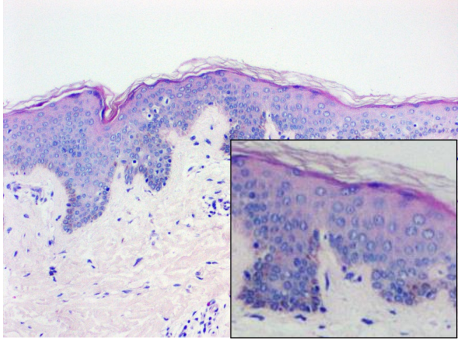

B: 25 years old, male, shoulder

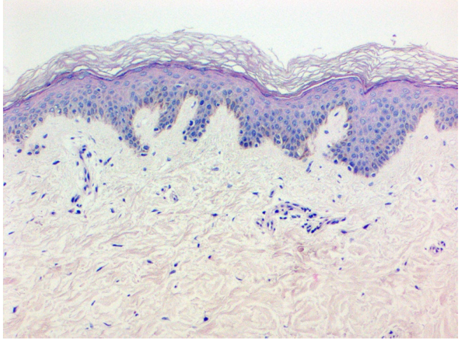

C: 24 years old, female, gluteal

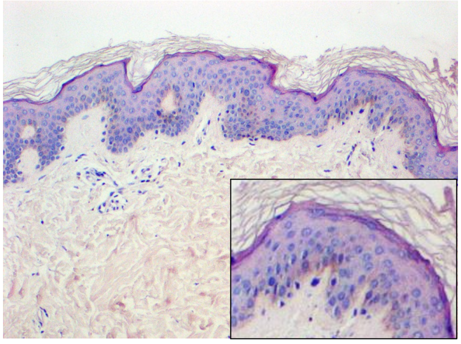

D: 24 years old, female, shoulder

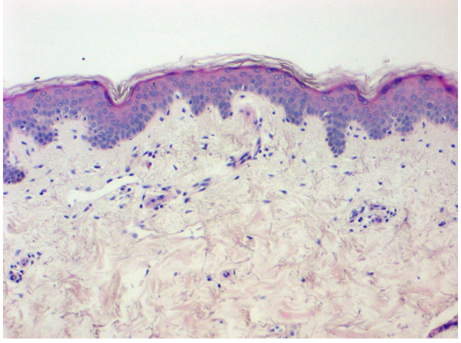

E: 63 years old, male, gluteal

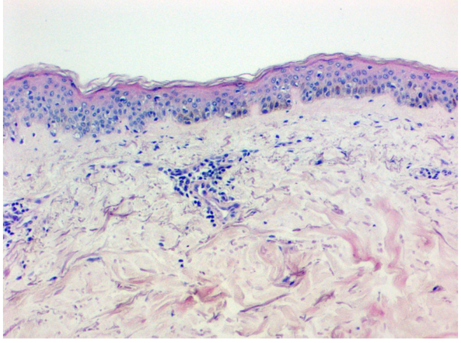

F: 63 years old, male, shoulder

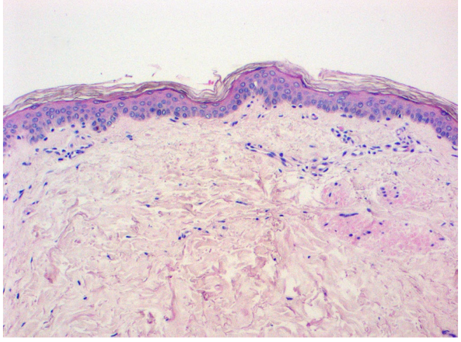

G: 60 years old, female, gluteal

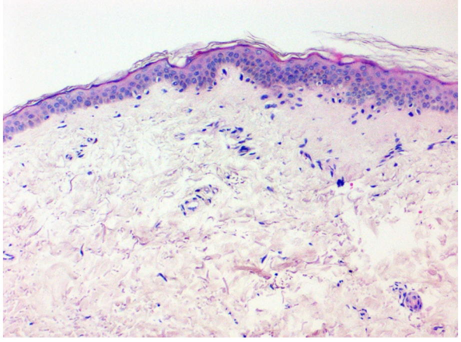

H: 60 years old, female, shoulder

Fig 2: Representative histological sections

Sections from eight samples derived from four donors. Two donors aged 24 and 25 years (Young age group) and two donors aged 60 and 63 years (Old age group). Two donors were male and two donors were female.

H&E staining of paraffin sections inf Fig 2 from skin of a young male (A) and female (C) demonstrate the characteristic rete-rich structure of the epidermis and the well-structured dense dermis of young skin.
Sun-exposed sites as demonstrated for sections of the neck of both male (B) and female young donors (D) show the same morphological characteristics. Only a tendency for an increased number of melanocytes (brown staining) in the basal layer of the epidermis (insert in B) may reflect increased UV exposure.
The histological appearance of H&E-stained sections from old male (E and F) and female (G and H) donors on the contrary, demonstrates the age-dependent reduction in rete ridges and epidermal thickness, potential reduction in thickness of the stratum corneum and destruction of the dermal collagen structure.
Interestingly, epidermal flattening (decrease of rete ridges) appears more pronounced in old female as compared to age-matched male skin than in male skin. At sun-exposed sites (photoaging), in both male (F) and female (H) skin the aging phenotype appeared even more pronounced.

3 Fastq reads and quality

3.1 Global distribution of Phred scores

We controlled the distribution of sequencing quality scores. Fig 3 shows distribution of phred scores in flowcells from Illumina sequencing. On each flowcell, 8 samples were sequenced.
Flowcell *b014* had inacceptable low phred scores. We excluded this flowcell from our analysis, because we found out, that batch effects associated with these samples could not be removed by filtering for adequate (e.g. >30) phred scores [81]. In the remaining samples, generally >80 % of phred scores were >30.
Fig 4 shows position wise distributions of phred scores for a representative sample.
Detailed information and links to Fastq files can be downloaded from ArrayExpress [1].

[1] `http://www.ebi.ac.uk/arrayexpress/files/E-MTAB-4652/E-MTAB-4652.sdrf.txt`

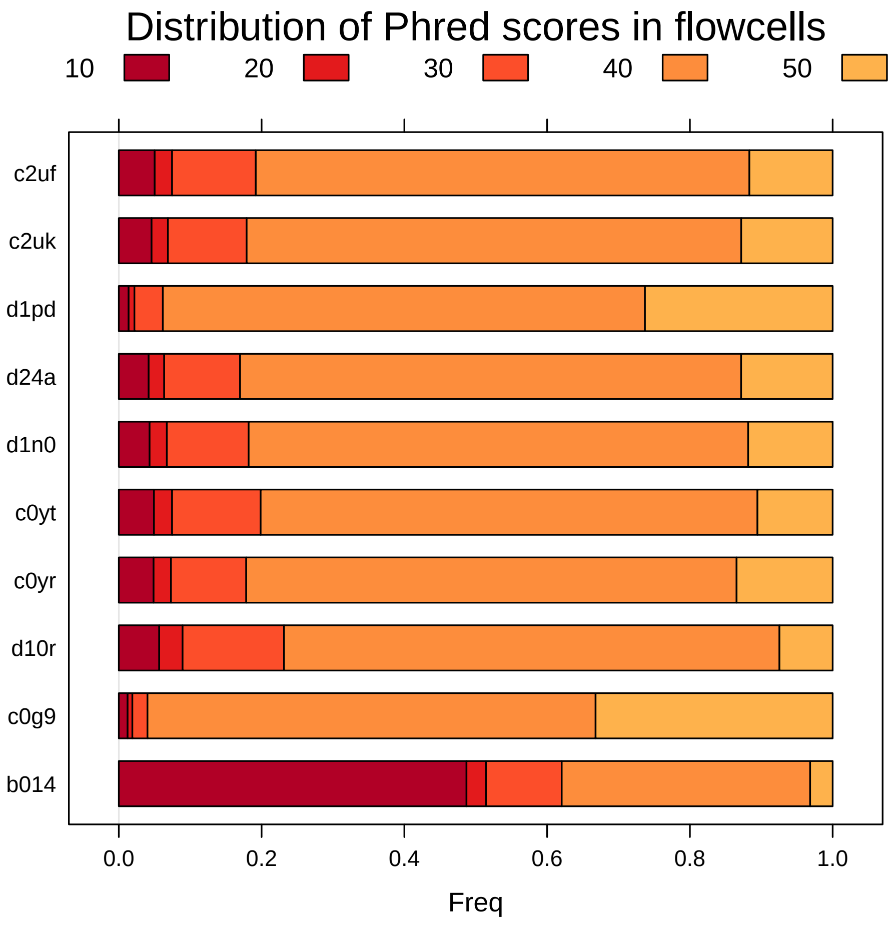

Fig 3: Distribution of Phred scores in Flowcells

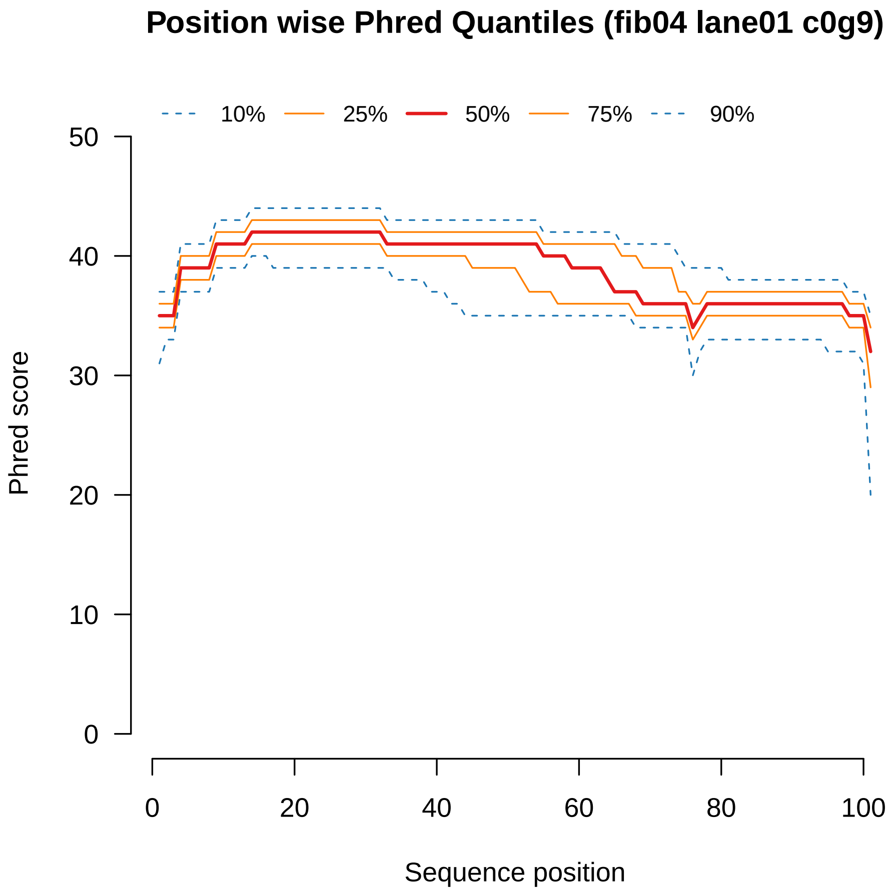

Fig 4: Distribution of Phred scores from sample grs_25_mal_bks_033

3.2 Nucleotide frequency in sequencing data

We counted nucleotide frequency in all FASTQ files. Fig 5 shows position wise nucleotide frequency in a representative sample (flowcell c0g9, sample grs_25_mal_bks_033) from a 25 year old male donor. The aberration from equal distribution visible in the first 20 positions is due to random hexamer priming.

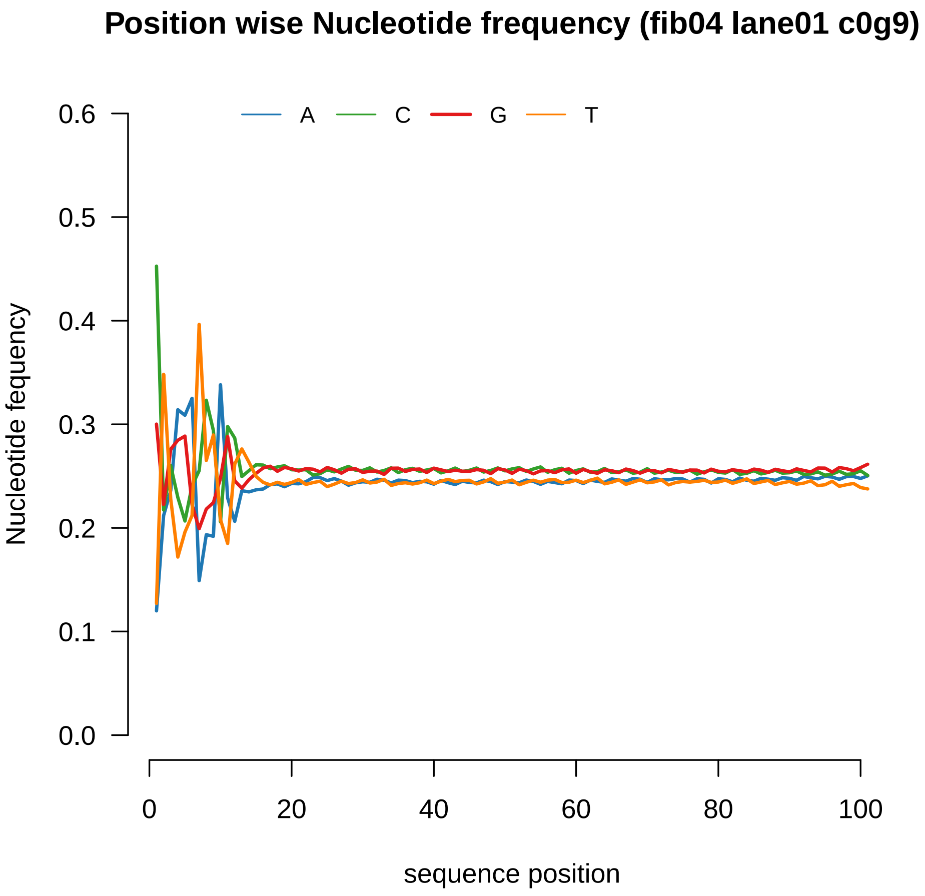

Fig 5: Distribution of nucleotide frequencies from sample grs_25_mal_bks_033

We analyzed GC content in our FASTQ files. Fig 6 shows distribution of GC content for all sequencing reads derived from flowcell *c0g9*. The relative GC content is distributed in a bell shaped manner with sufficient overlap between all samples.

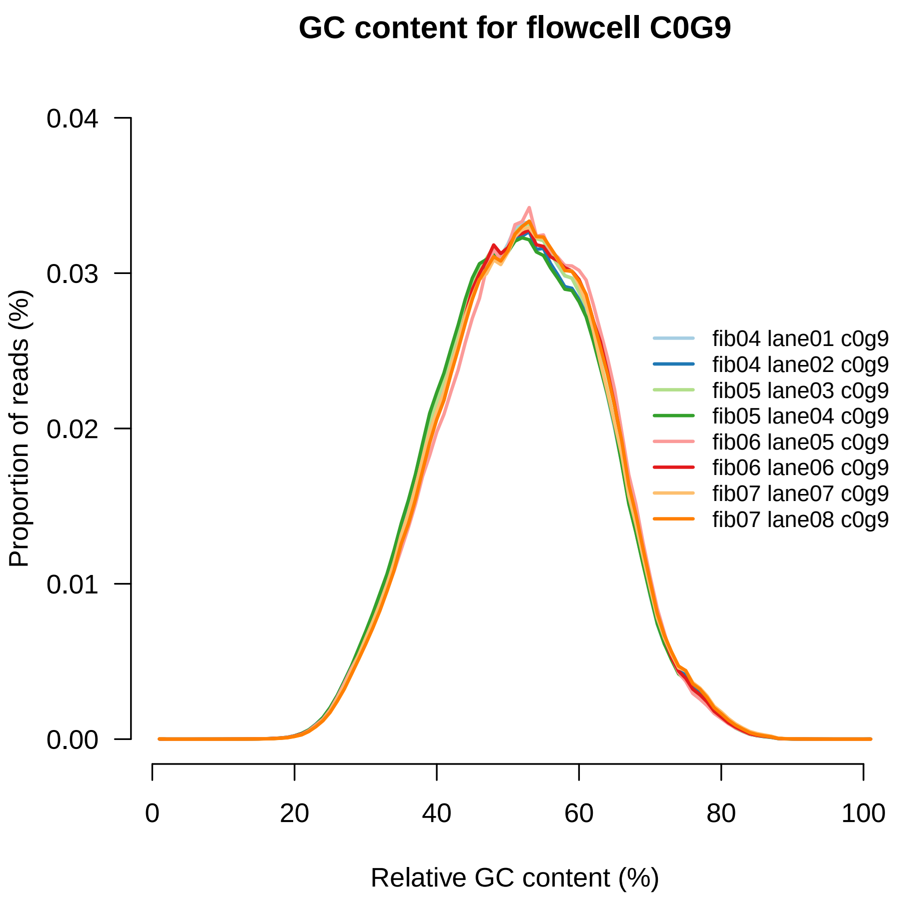

Fig 6: Distribution of GC content for flowcell C0G9

4 Reference Genome and annotation data

4.1 Human DNA sequence

For calculation of all genomic alignments, Human genomic reference sequence GRCh38, downloaded from Ensembl release 76 [2] on 06th March 2015 has been used.
Prior to alignment, primary assembly sequences (Chromosomes) had been extracted from Fasta files using *Biostrings* (Bioconductor) using the following R code.

```
> library(Biostrings)
> ## Read native DNA file
> dnaa <- readDNAStringSet("GRCh38.fa")
> ## Extract primary assembl sequences
> pa <- grep("^[1-9,M,X,Y]", names(dnaa))
> dna <- dnaa[pa]
> # - - - - - - - - - - - - - - - - - - - - - - - - - - - - #
> # Extract chomosome name from sequence name:
> # seq name: "10 dna_sm:chromosome chromosome:GRCh38: (...)
> # chr name: "10"
> # - - - - - - - - - - - - - - - - - - - - - - - - - - - - #
> c_name_len <- attr(regexpr("^\\w+", names(dna)),
+                       "match.length")
```

[2] http://ftp.ensembl.org/pub/release-76/fasta/homo_sapiens/cds/Homo_sapiens.GRCh38.cds.all.fa.gz

```
> names(dna) <- substr(names(dna), 1, c_name_len)
> # Save Fasta
> writeXStringSet(dna,
+         file="hs_grch38.fa",
+         format="fasta")
```

4.2 Annotation of genomic features

Human genomic annotation data was downloaded from Ensembl release 82 on 15th Dec 2015 [3]. For gene set enrichment analysis (GSEA), Ensembl gene ID need to be translated into Entrez identifier. We therefore downloaded a translation table from Ensembl using BiomaRt on 27th Jan 2016 using the following code.

```
> # Translation of ENSEMBL to ENTREZ
> library(biomaRt)
> hsen <- useMart("ensembl", dataset="hsapiens_gene_ensembl")
> la <- listAttributes(hsen)
> att <- la$name[c(1:4, 58,59)]
> bmEn82 <- getBM(attributes=att, mart=hsen)
> save(bmEn82, file="hsen82_entrez.RData")
```

5 Analytic procedures

5.1 Standard differential expression (DE) analysis: EQLF

Identification of significant differential expressed genes was done using a two sample approach. Therefore, from the three age groups present in the samples (Young, Middle, Old) the Middle age group was excluded (Young vs Old = yvo approach).
The standard differential expression (DE) analysis procedure consists of Read alignment using TopHat, Gene wise alignment counting using *summarizeOverlaps*, Quasi-likelihood F-Tests from edgeR framework and GSEA, GO and KEGG pathway analysis using limma.

5.2 Counting alignmnets using summarizeOverlaps

```
> library(Rsamtools)           # BamFileList
> library(GenomicAlignments)  # summarizeOverlaps
> bfl <- BamFileList(tophatBamFiles, yieldSize=5e4)
> tsovc <- summarizeOverlaps(gen82, bfl, mode="Union")
> tyosovc <- tsovc[ ,ageGroup!="middle"]
```

5.2.1 Statistical testing for DE using edgeR

```
> library(GenomicAlignments)
> # Assignment of samples to age group (from yvo samples table)
> yvomod <- model.matrix(~yvo$agg)
> # Loads gene-wise alignment count data
> # produced by summarizeOverlaps (tyvosovc)
> load(file.path(datdir, "g54t_en82_sov_counts.RData"))
> # Extract count matrix with gene_id's
> mtx <- assay(tyosovc)
> rownames(mtx) <- rownames(tyosovc)
> # DGEList
> tyvogel <- DGEList(mtx,  group=yvo$agg)
> tyvogel <- calcNormFactors(tyvogel)
```

[3] ftp://ftp.ensembl.org/pub/release-82/gtf/homo_sapiens/Homo_sapiens.GRCh38.82.gtf.gz

```
> tyvogel <- estimateDisp(tyvogel, yvomod)
> # Quasi-likelihood F-Tests:
> tyvoqlf <- glmQLFit(tyvogel, yvomod)
> tyvoqlf <- glmQLFTest(tyvoqlf, coef=2)
> topTags(tyvoqlf)
> # Likelihood ratio tests:
> tyvolrt <- glmFit(tyvogel, yvomod)
> tyvolrt <- glmLRT(tyvolrt, coef=2)
> topTags(tyvolrt)
```

5.3 Differential expression analyis using MALDR approach

We exemplify MALDR approach for age-groups. From age-related gene expression, it is known that observed effects are small and that considerable variation must be expected. Given a negative result of a standard approach (i.e. no significant age related difference using EQLF), we presumed that an alternative approach could be more sensible. The MALDR approach combines two criteria

- Preselection of genes using alignment counts on gap-sites only on two groups (Young and Old). This method estimates gene expression at multiple sites for each gene (for multi-exon genes) and provides high sensitivity
- Validation of genes using monotone alignment depth ratios along multiple sample groups and on nearly the whole alignment covered range of a gene. This is a quite strict criterion and should have a high specicificy for a continuosly proceeding effect as ageing.

The constituents of the MALDR approach are implemented in our splicing - events analysis toolchain consisting of tree R packages *rbamtools* (CRAN) [79], *refGenome* (CRAN) [80] and *spliceSites* (Bioconductor) [78].

5.3.1 Workflow

Read align count matrix on gap-sites using spliceSites. The *readExpSet* function reads alignment count data into an *ExpressionSet* object [4]

```
> library(spliceSites)
> pd <- new("AnnotatedDataFrame", data=prof, varMetadata=meta)
> tes <- readExpSet(bamFileNames, phenoData=pd)
```

Differential expression (DE) analysis using edgeR. For DE analysis, first, the count matrix (using *exprs*) and the data on gap-site positions (using *featureData*) are extracted resulting in 1,000,380 gap-sites.

```
> mtx <- exprs(tes)                         # 1.000.380
> rsm <- apply(mtx, 1, min, na.rm=TRUE)
> mtx <- mtx[rsm > 5, proj$agg != "m"]      #   101.771
```

Next, gap-sites below a minimal align-number (5 total alignments in 54 samples) are removed resulting in 101,771 gap-sites. The filtered alignment counts are analyzed for DE between two age groups (Young vs. Old) using EQLF.

```
> library(edgeR)
> fd <- featureData(tes)
> fdd <- fd@data
> yvomod <- model.matrix(~yvo$agg)
```

[4] ExpressionSet objects are designed for usage in Microarray Analysis. Meanwhile, *SummarizedExperiment* have emerged as standard container for this kind of data. But still, using this approach, the count data can straightly be transferred into edgeR analysis workflow.

```
> tyvogelxst <- DGEList(mtx)
> tyvogelxst <- calcNormFactors(tyvogelxst)
> tyvogelxst <- estimateDisp(tyvogelxst, yvomod)
> # Quasi - likelihood F-tests
> tyvoqlfxst <- glmQLFit(tyvogelxst, yvomod)
> tyvoqlfxst <- glmLRT(tyvoqlfxst, coef=2) #   101.771
```

After DE testing, all gap-sites are extracted together with p-values and FDR values.

```
> # Extract complete table from DGELRT
> tt <- topTags(tyvoqlfxst, n=nrow(tyvoqlfxst$table))$table
> tt$qid <- rownames(tt)
```

Annotation of gap-sites. From their genomic positions, the gap-sites are annotated. The *overlapJuncs* (refGenome) function performs annotation specialized for gap-sites and is implemented in *refGenome*. The *enjc82* object is of type *refJunctions* and can be created from GTF data (e.g. downloaded from Ensembl).

```
> # Position data from AnnotatedDataFrame
> fdd$id <- 1:nrow(fdd)
> # Do overlap
> an <- overlapJuncs(fdd, enjc82)
> # Transfer annotation to AnnotatedDataFrame
> fdd$gene_id[an$qid] <- an$gene_id
> fdd$gene_name[an$qid] <- an$gene_name
> fdd$transcript_id[an$qid] <- an$transcript_id
> fdd$strand[an$qid] <- an$strand
> fdd$sod[an$qid] <- an$sod
> fdd$qid <- rownames(fdd)
> # Merge ADF data with DGELRT data
> terex <- merge(tt, fdd, by="qid", all.x=TRUE)
```

After annotation, the remaining gap-sites table is restricted to annotated gap-sites (i.e. sod==0) meaning that only gapped alignments on annotated splice sites are further considered. The previous 5 alignment criterion already is a strict criterion since 98.3 % of the filtered gap-sites are annotated splice sites. After this step, 100,040 gap-sites remain in analysis.

```
> tersoz <- terex[!is.na(terex$sod),]
> tersoz <- tersoz[tersoz$sod==0, ]   # 100,040
```

Filter differential expressed annotated splice sites. From these gap-sites, the significant differential expressed gap-sites are identified using a FDR limit of 0.1. About 4.6 % of the gap-sites are significant age related expressed (4,626).

```
> tersig <- tersoz[tersoz$FDR < 0.1,] #   4,626
```

From significant regulated gap-sites, the unique present Ensembl gene_id is extracted and translated into Entrez id. In effect, every gene for which at least one splice site is differently expressed is extracted resulting in 790 different genes.

```
> genes <- unique(tersig$gene_id)      #      790
```

Collect alignement depth data on selected genes For these 790 genes alignment depth data is collected by counting alignments on all 54 BAM files for a genomic region associated with each gene. The locations of the BAM files and their assignment to sample group are managed in *sampleBamFiles* objects.

Table 2: **Number of gap-sites and gene in MALDR analysis**

Step	Type	Number of sites
Raw gap-sites	gap-sites	1,000,380
Filtered gap-sites	gap-sites	101,771
Annotated splice sites	gap-sites	100,040
FDR limit 0.1	gap-sites	4,626
Unique Ensemble Gene ID	gene	790
Monotone ALD ratio	gene	42

```
> bs <- sampleBamFiles(gsc$topBam)
> sampleLabels(bs) <- gsc$probe
> sampleGroups(bs) <- gsc$agg
> nAligns(bs) <- gsc$topBamAligns
> groupTable(bs) <- gsc
```

The genetic regions for all 790 genes are carried inside *geneList* objects.

```
> aldrat <- saveAldData(bs, gl, path, order=order, startId=startId)
```

The *saveAldData* function encapsulates several processing steps:

- Read alignment depth (AD) and gap-sites data data from BAM files for all genes.
- Cut out intronic regions (defined by gap-sites)
- Calculate mean AD per sample group (Young / Middle / Old)
- Calculate Loess model (ADL) for groupwise mean ALD values
- Cut out low ADL regions. The remaining regions are those, where a significant amount of gene expression takes place (translated region).
- Calculate ADL ratio between adjacent groups (Young vs. Middle and Middle vs. Old) for each position in the translated region.
- Calculate fraction of positions where ADL ratio is at least 1.2 in either direction (Young $<$ Middle $<$ Old or Young $>$ Middle $>$ Old).

```
> aldrat <- saveAldData(bs, gl, path, order=order, startId=startId)
```

Selection of differential expressed genes Finally significant differential expressed genes are extracted with the criterion that monotone ADL ratio of at least 1.2 is maintained over at least 99 % of the translated region.

Number of sites during MALDR analysis process Due to various filtering steps in the MALDR analysis, the number of gap-sites or genes decreases to the final 42 differential expressed genes. Table 2 shows the number of sites after each filtering step.

5.4 Estimation of gene expression abundance

Two measures were uses for calculation of gene expression:

- Counts per million (CPM) estimates derived from Quasi likelihood F-tests (edgeR)
- Maximal align depth on alignment gap sites for gene calculated (rbamtools).

The following code segment shows a procedure for extraction of CPM values from edgeR derived regression models.

```
> library(edgeR)
> library(reshape)
> # - - - - - - - - - - - - - - - - - - - - #
> # Calculation of edgeR model (TopHat)
> # - - - - - - - - - - - - - - - - - - - - #
> allmod <- model.matrix(~agg+gen+loc, data=proj)
> # Extract count matrix with gene_id's
> mtx <- assay(tsovc)
> rownames(mtx) <- rownames(tsovc)
> # FILTER OUT low abundant genes
> minc <- apply(mtx, 1, min)
> mtx <- mtx[minc > clim, ]
> # DGEList
> tallgel <- DGEList(mtx)
> tallgel <- calcNormFactors(tallgel)
> tallgel <- estimateDisp(tallgel, allmod)
> # Quasi-likelihood F-Tests:
> tallqlf <- glmQLFit(tallgel, allmod)
> tallqlf <- glmQLFTest(tallqlf, coef=2)
> # - - - - - - - - - - - - - - - - - - - - #
> # Extract CPM values
> # - - - - - - - - - - - - - - - - - - - - #
> tseygc <- cpm(tallqlf$fitted.values, lib.size=exp(getOffset(tallgel)))
> names(dimnames(tseygc)) <- c("gene_id", "probe")
> # Reshape matrix + add sample information
> tseygc <- melt(tseygc)
> mtc <- match(tseygc$gene_id, gt82$gene_id)
> tseygc$gene_name <- gt82$gene_name[mtc]
> mtc <- match(tseygc$probe, proj$project)
> tseygc$gen <- proj$gen[mtc]
> tseygc$age <- proj$age[mtc]
> tseygc$agg <- proj$agg[mtc]
> tseygc$loc <- proj$loc[mtc]
> tseygc$indiv <- proj$indiv[mtc]
> levels(tseygc$gen) <- c("Female", "Male")
> levels(tseygc$loc) <- c("Gluteal", "Shoulder")
```

5.4.1 CPM values

CPM estimates provided from edgeR regression model. Fig 7 shows the edgeR derived CPM estimates for each age (in years), gender and location. The estimates show a distinctive decrease of CPM estimates in a similar pattern for gender and sample location. The raw CPM values are shifted towards the group mean, so that the considerable variation present in the data is not present here. The shifted CPM values obscure the fact, that the monotone group differences arise from just three samples with high gene expression. We therefore think, that edgeR derived CPM values are not suitable vor visual inspectation in this setting.

5.4.2 Gene filtering

Following DE analysis, genes are filtered using a lower limit for count-per-million (CPM) which is provided by the *cpm* function in *edgeR*.
As pointed out in *edgeR User's Guide* [5], filtering shall ensure read counts of at least 5 - 10 for a gene. Using

```
> range(tyvogel$samples$lib.size)
```

[5] https://www.bioconductor.org/packages/3.3/bioc/vignettes/edgeR/inst/doc/edgeRUsersGuide.pdf

Fig 7: **edgeR CPM estimates for ID1**

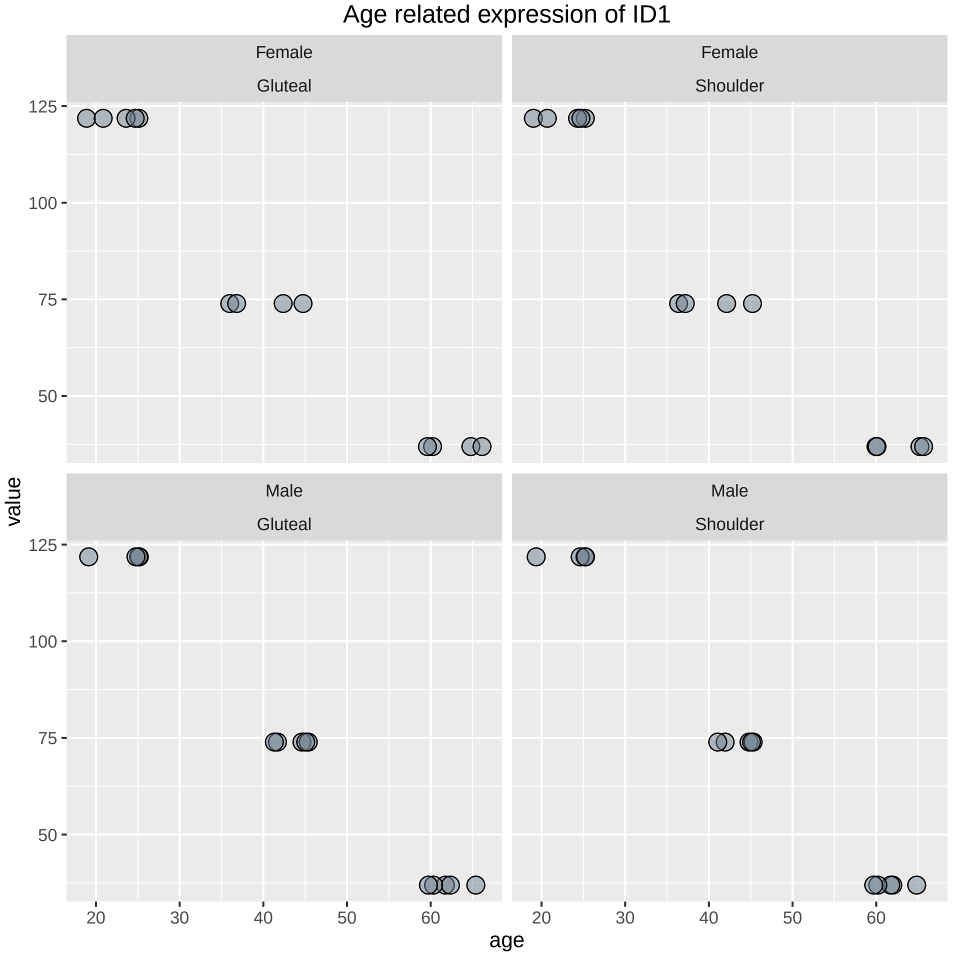

Counts per million (CPM) values for ID1 estimated by edgeR.

we get read counts between 21.8 10^6 and 107 10^6 reads per sample. We therefore set the lower limit for cpm to 0.23.

5.4.3 GO and KEGG pathway analysis

Translation of Ensembl Gene ID to Entrez id revealed that a few Entrez identiers occured multiple times (82 ID's twice, 10 ID's three times in age group testing). We unified Entrez identifier by extracting the first feature with the lowest P-value for each Entrez identifier. Gene set analyis requires definition of two sets of genes: The selected genes (i.e. significant differentially expressed genes) and a gene universe (defining the whole population). The two gene sets were selected according to the following scheme:

- Selected Genes: Genes where the *topTags* table (from edgeR QLF-Test) exhibited an FDR 0.1.
- Universe: All Entrez ID's in complete edgeR QLF-Test result table.

Testing for enriched GO terms and KEGG terms was performed using *goana* and *kegga* functions from *limma* (Bioconductor.)

```
> # Use limma goana for GO testing
> sig_genes <- as.character(eolre$entrez[eolre$FDR < fdrlim])
> universe <- eolre$entrez
> ego <- goana(sig_genes, universe)
> tgo <- topGO(ego, number=Inf)
```

```
> tgo <- tgo[tgo$P.DE < 0.05, ]
> # Use limma kegga
> keg <- kegga(sig_genes, universe)
> tkg <- topKEGG(keg, number=Inf)
> tkg <- tkg[tkg$P.DE < 0.05, ]
```

6 Additional results from DE analysis

6.1 Analysis for age group

6.2 Results from DE analysis for age-group (Young vs. Middle vs. Old)

From this procedure, we extracted 42 significant age regulated genes shown in Table 3. The table is ascending ordered according to QLF FDR values (i.e. ATOH8 has lowest QLF FDR value).

Functional annotation with GO terms

Functional annotation with GO terms revealed 609 enriched GO terms. The complete term lists are contained in 10. The words *mitochondrion* or *mitochondrial* are not contained in GO-term names. Table 4 shows filtered GO terms.

We analysed GO terms in order to identify relations to fibroblast physiology or ageing process. The GO-terms of the 609 significant enriched GO ID's contain 437 different words. Biologic terms the 20 most abundant words are (number of orrences in brackets) *development* (20), *morphogenesis* (38), *muscle*(38), *differentiation*(32), *growth* (31), *cardiac*(30) and *metabolic*(22).

Table 3: **Age related differentially expressed genes**

Gene name	Functional group	Regulation
ATOH8	bHLH transcripton factor	Down
PODXL	Membrane protein, cell adhesion	Down
SNAI1	transcription factor	Down
ID3	bHLH inhibitor	Down
SPHK1	Kinase	Down
ID1	bHLH inhibitor	Down
ERRFI1	Cytosolic adaptor/scaffold protein	Down
PENK	Endogene opioid precursor	Up
SEPT5	Cell skeleton / P-loop	Down
CPZ	Metallo carboxypeptidase	Up
PRPS1	Enzyme, nucleotide synthesis	Down
MEG3	lncRNA regulating p53	Down
CNN1	Actin regulating protein	Down
STC1	Ca/Ph regulating, Mitochondrial electron transport	Up
KIAA1324L	Estrogen induced protein	Up
TRNP1	Cerebral cortex expansion	Down
HSPB7	Heat shock protein	Down
PRRX2	Periredoxin (ROS inactivating enzyme)	Down
SMAD7	TGFB effector	Down
FAM83G	TGFB effector	Down
DDR1	Collagen sensing receptor kinase	Down
PPP1R3C	Protein phosphatase subunit	Down
EVA1A	ER membrane protein	Down
CRISPLD2	LPS binding protein	Down
RP11-309L24.6		Down
ZNF385D		Up
FGFRL1	FGF receptor	Down
CKB	Creatin kinase (brain type)	Down
FILIP1L		Down
GJA1	Gap junction constituting protein	Up
ENC1	Actin binding protein	Down
SH2D4A	Adaptor protein on T-cells	Down
ARHGAP23P1		Down
SERTAD1	Transciptional activator	Down
FGF13	Na^{+}-channel, intracellular singalling protein	Up
EHD1	Protein recycling receptor	Down
USP41	Deubiquitating enzyme	Up
ACSS3	AcylCoA synthetase	Down
BACE2	Secretase (Amyloid Aß proteolysis)	Up
ADGRL4	G-protein coupled receptor	Up
ROBO1	Transmembrane receptor	Up
KCNC4	Voltage gated Kalium channel	Down

Significant age related differential expressed genes. Up-regulation denotes Young<Middle<Old gene expression. Down-regulation denotes Young>Middle>Old gene expression. The table is ascending ordered according to QLF FDR values (i.e. ATOH8 has lowest QLF FDR value).

Table 4: **Selected significant enrichted GO terms**

GO Term	Ontology
Fibroblast growth factor-activated receptor activity	MF
Fibroblast growth factor binding	MF
Regulation of extracellular matrix disassembly	BP
Smooth muscle cell-matrix adhesion	BP
Regulation of extracellular matrix disassembly	BP

GSEA using topGO revealed 609 significant enrichted GO terms. The table contains the terms in which any of the words fibroblast, extracellular, matrix or mitochon* appears.

6.3 Analysis of correlation of CPM values

We identified a small subset of age VAR genes which are functionally related to TGF-β signalling pathway (ATOH8, ID3, ID1, SMAD7 and FAM83G) and for which gene expression (as measured in CPM) is highly positively correlate. In order to determine whether this correlation is usual in our gene expression data and eventually to identify further genes for which CPM closely correlates with genes ATOH8, ID3, ID1, SMAD7 and FAM83G we analysed correlation coefficients (CC) for CPM values for in all genes as well as in our 42 age VAR genes.

Correlation coefficients for all genes in gene ENSEMBL 82.

As calculation of CC for 60619 has high computational and memory demand, we repeatedly drew random samples of size 2000 in order to obtain distribution of pairwise CC. Figure **??** shows correlation coefficients (CC) for CPM values for one representative gene random sample. Gene expression data, positive correlation coefficients are roughly 15 % underrepresented (mean percentage of positive CC: 86 %, 95% confidence interval: 82 % - 89 % from 100 random samples).

Correlation coefficients for all 42 age related DE genes

CC for all 42 age related DE genes are shown in Fig **??**. For age related DE genes, 29.0 % of CC are positive and a substantial part of CC (26.8 %) are >0.5. Positive correlation occurs in these 42 age related genes much more frequently than expected from numbers of the whole measured transcriptome.

Age related DE with high correlation coefficients

Calculation of pairwise CC was done on 42 age related DE genes. We calculated all pairwise CC and extracted all genes for which at least one absolute CC value is >0.9 ($|CC| > 0.9$) knowing, that the functionally related gene group ATOH8, ID3, ID1, SMAD7 and FAM83G is contained therein. The procedure returned 9 genes. Gene- and sample-wise CPM values are plotted in Fig **??**. The panel verifies, that CPM values are positively correlated between all 9 genes, which is not self evident as negative regulation is not uncommon in many pathways.
The identified high correlating genes are: ATOH8, SNAI1, ID3, SPHK1, ID1, PRRX2, SMAD7, FAM83G, SERTAD1.

Fig 8: Correlation coefficients for a random sample of genes

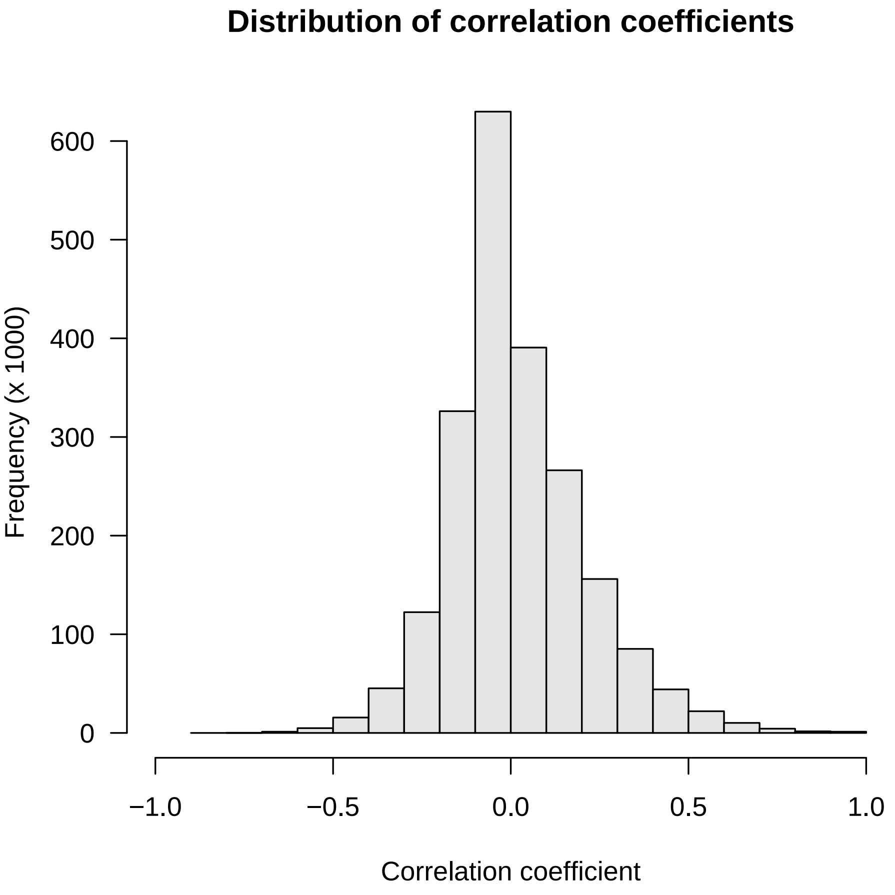

A sample of 2000 randomly selected genes was drawn from all present genes. The subset from CPM count matrix for these genes was drawn and correlation coefficients for these genes were calculated. Diagonal values (i.e. variances) were removed and counted numbers were divided by 2 because due to symmetry of covariance matrix, each correlation is present twice.

Fig 9: Correlation coefficients for 42 significant age related DE genes

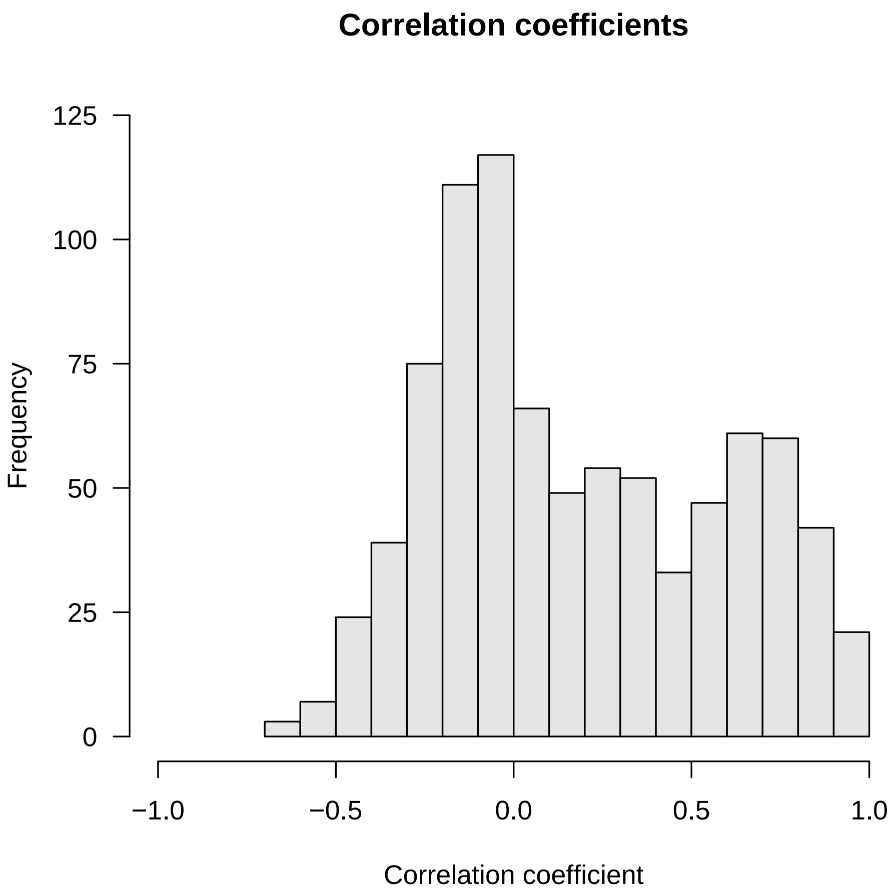

The subset from CPM count matrix for 42 significant age related DE genes was extracted and correlation coefficients for these genes were calculated. Diagonal values (i.e. variances) were removed and count numbers were divided by 2 because each correlation value is present twice in covariance matrix due to symmetry.

Fig 10: Pairs plot for CPM values genes with highly correlated CPM values

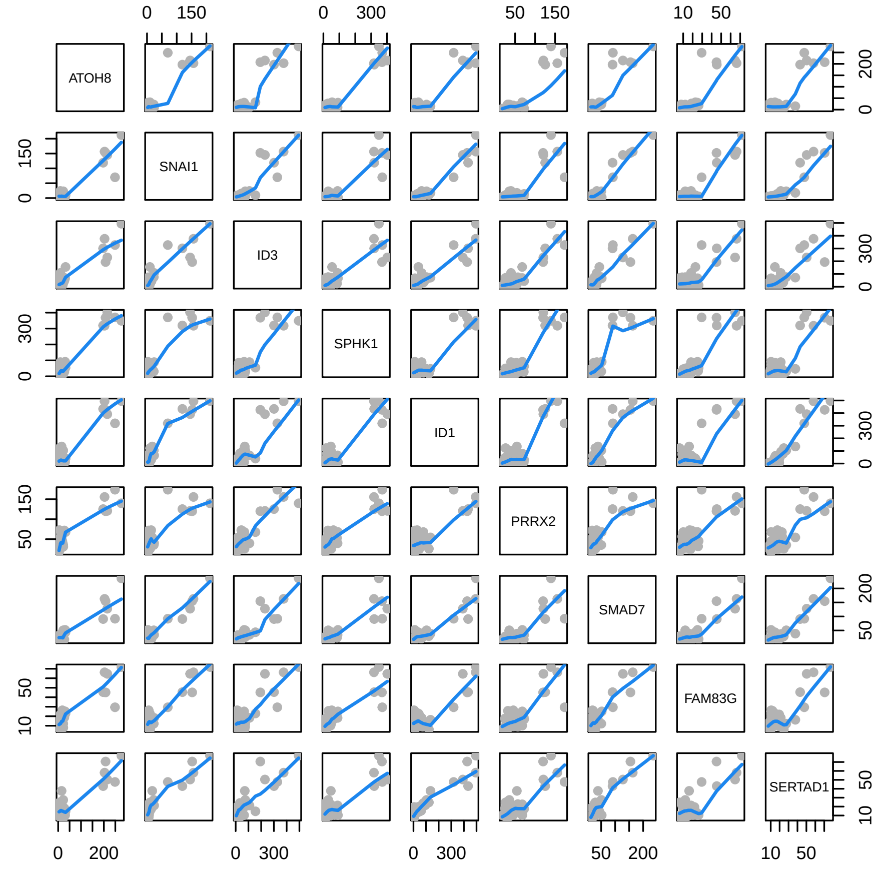

The subset from CPM count matrix for 42 significant age related DE genes was extracted and correlation coefficients for these genes were calculated. Genes for which at least one correlation coefficient is >0.9 were extracted, resulting in 9 genes.

6.4 Correlation of CPM values between age related DE genes

Fig 11: **Correlation coefficients for genewise CPM value**

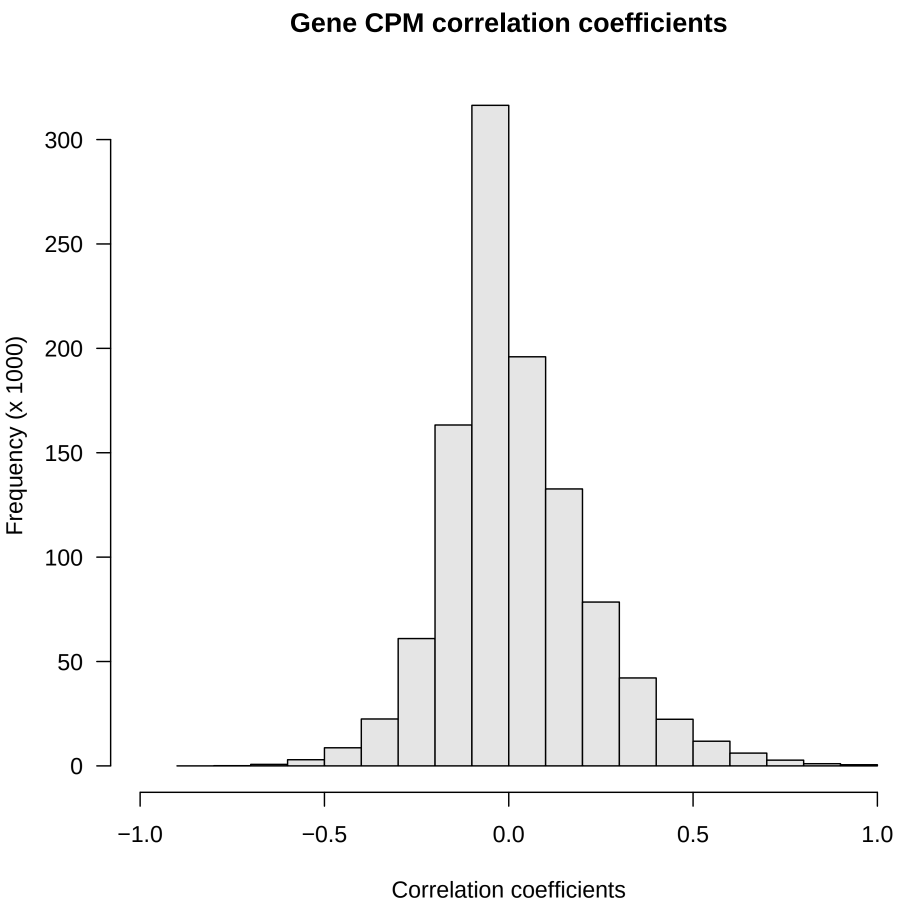

Counts per million (CPM) values derived from summarizeOverlaps. Correlation coefficient (CC) for genewise CPM values were calculated from a random sample of size 2000. For each pair of genes, one CC was counted.

6.5 Results from DE analysis for gender (Female vs. Male)

6.6 Gender related differential expressed genes

In this study, 30 female and 30 male participants were included. Due to impaired data quality, data from 6 samples was excluded from analysis. From the analysed samples, 26 derived from female participants and 28 derived from male participants. Due to the fact, that age related differences in gene expression tend to be small, we include a standard differential expression analysis for sample groups *female* versus *male.*

6.6.1 Results from EQLF analysis

Standard differential expression analysis using edgeR quasi likelihood F-test renders 168 significant DE genes. The complete table is contained in 10.

6.6.2 Functional annotation with GO terms

GSEA for GO terms resulted in 391 significantly enriched GO terms. The complete table is available in supplementary data 10. Table 5 shows the 20 most significant enriched GO terms. In the terms predominantly are associated with biosynthesis of steroids and histone demethylation.

Term	Ont	N	DE
cholesterol biosynthetic process	BP	49	5
secondary alcohol biosynthetic process	BP	49	5
sterol biosynthetic process	BP	54	5
steroid biosynthetic process	BP	151	6
alcohol biosynthetic process	BP	152	6
isoprenoid biosynthetic process	BP	26	3
histone H3-K4 demethylation	BP	6	2
histone demethylase activity (H3-K4 specific)	MF	6	2
cholesterol metabolic process	BP	121	5
secondary alcohol metabolic process	BP	124	5
histone demethylase activity	MF	33	3
sterol metabolic process	BP	132	5
histone lysine demethylation	BP	35	3
histone demethylation	BP	36	3
steroid metabolic process	BP	286	7
protein demethylation	BP	38	3
protein dealkylation	BP	38	3
multicellular organism growth	BP	147	5
purine ribonucleotide metabolic process	BP	480	9
demethylase activity	MF	41	3

Table 5: Top 20 enriched GO germs for gender DE genes from EQLF

6.6.3 Functional annotation with KEGG pathways

GSEA analysis for KEGG pathways returned 24 significant enriched pathways. Table 6 displays identity of enriched pathways.

Pathway	N	DE
Terpenoid backbone biosynthesis	22	3
Steroid biosynthesis	20	2
Neuroactive ligand-receptor interaction	276	4
Adrenergic signaling in cardiomyocytes	147	3
RNA transport	170	3
Metabolic pathways	1232	7
PPAR signaling pathway	72	2
Cytokine-cytokine receptor interaction	263	3
GABAergic synapse	88	2
Salivary secretion	88	2
Dilated cardiomyopathy	89	2
Thyroid hormone signaling pathway	118	2
Synthesis and degradation of ketone bodies	10	1
Ribosome	134	2
Tight junction	139	2
Cell adhesion molecules (CAMs)	141	2
cGMP-PKG signaling pathway	166	2
Axon guidance	175	2
Calcium signaling pathway	178	2
Chemokine signaling pathway	184	2
Glycosaminoglycan biosynthesis - heparan sulfate / heparin	24	1
cAMP signaling pathway	198	2
Butanoate metabolism	28	1
Other types of O-glycan biosynthesis	31	1

Table 6: Enrichted KEGG pathways for gender DE genes from EQLF

6.7 Results from DE analysis for location (Gluteal vs. Shoulder)

From each study participant, two fibroblast samples were obtained: One sample from gluteal region (UV protected) and one sample from shoulder (UV exposed). We show analysis results for differential gene expression analysis for Gluteal versus Shoulder.

6.7.1 Gene list

Standard differential expression analysis using EQLF returns 56 significant DE genes. The complete list is contained in supplementary material 10.

6.7.2 Functional annotation with GO terms

GSEA for GO terms resulted in 319 significantly enriched GO terms. The complete table is available in supplementary data 10. The enriched GO categories are not to be directly related to fibroblast physiology or senescence.

6.7.3 Functional annotation with KEGG pathways

GSEA analysis for KEGG pathways revealed 16 significant enriched pathways, shown in Table 8. The pathways and the number of affected pathways indicate no direct relationship with fibroblast physiology or senescence.

Term	Ont	N	DE
positive regulation of somatostatin secretion	BP	2	2
regulation of somatostatin secretion	BP	3	2
somatostatin secretion	BP	5	2
pancreatic juice secretion	BP	13	2
dopaminergic neuron differentiation	BP	14	2
gastric acid secretion	BP	16	2
signal transduction involved in regulation of gene expressio	BP	18	2
epithelial tube branching involved in lung morphogenesis	BP	28	2
thyroid hormone catabolic process	BP	1	1
negative regulation of transcription from RNA polymerase II	BP	1	1
carbon catabolite repression of transcription from RNA polym	BP	1	1
regulation of detection of glucose	BP	1	1
negative regulation of detection of glucose	BP	1	1
regulation of gastrin-induced gastric acid secretion	BP	1	1
negative regulation of gastrin-induced gastric acid secretio	BP	1	1
inner cell mass cellular morphogenesis	BP	1	1
cardiogenic plate morphogenesis	BP	1	1
regulation of transcription from RNA polymerase II promoter	BP	1	1
natural killer cell apoptotic process	BP	1	1
regulation of natural killer cell apoptotic process	BP	1	1

Table 7: Top 20 enriched GO germs for location DE genes (Gluteal vs. Shoulder) from EQLF (Term truncated to 60 characters)

Pathway	N	DE
Neuroactive ligand-receptor interaction	276	3
Oxytocin signaling pathway	155	2
Glycosaminoglycan biosynthesis - heparan sulfate / heparin	24	1
Maturity onset diabetes of the young	26	1
Apoptosis - multiple species	33	1
Cocaine addiction	49	1
Longevity regulating pathway - multiple species	64	1
Amphetamine addiction	66	1
Arrhythmogenic right ventricular cardiomyopathy (ARVC)	74	1
Cardiac muscle contraction	78	1
Hypertrophic cardiomyopathy (HCM)	83	1
Insulin secretion	85	1
Small cell lung cancer	86	1
Dilated cardiomyopathy	89	1
Pancreatic secretion	96	1
Phosphatidylinositol signaling system	97	1

Table 8: Enrichted KEGG pathways for location DE genes (Gluteal vs. Shoulder) from EQLF analysis

7 Fibroblast physiology

We relate known fibroblast physiology with our gene expression data focussing on signalling pathways and cellular senescence.

Transforming growth factor pathway(TGF-β)

TGF-β signalling pathway has been associated with ageing fibroblast phenotype [149] and induction of cellular senescence [91, 26]. TGF-β and *bone morphogenic protein* (BMP) transmit signals intracellularly by transmembrane serine/thyrosine kinase receptors. Their major intracellular effectors are SMAD proteins which are divided in three groups:

- Receptor-activated SMADs (R-SMADs: SMAD -1, -2, -3, -5) are phosphorylated by activated TGF-β receptor. R-SMADs -2 and -3 mainly transmit TGF-β signals, while R-SMADs -1, -5 and -8 mainly transmit BMP signals [47].

- Inhibitory SMADs (I-SMADs: SMAD -6 and -7) inactivate TGF-β receptor by blocking receptor kinase domain [154, 59].

- SMAD 4 binds to phosphorylated R-SMADs which is then translocated into the nucleus.

The actually TGF-β/ SMAD regulated genes are highly dependent on the cellular context [113, 149]. The TGF-β/ R-SMAD and the SMAD7 regulation are highly conserved.

Cellular senescence

Cellular senescence[6] (CS) is defined by irreversible cell cycle arrest in cells which are not quiescent or terminally differentiated [107, 26, 20, 176, 124]. Cellular senescence is divided in two major subclasses:

- *Acute senescence* is a response to diverse types of limited stressors and part of many physiological processes (e.g. embryonic developmment and tissue repair). Here secreted cytokines induce immunologic removal of senescent cells enabling tissue regeneration from progenitor cells.

- *Chronic senescence* is a consequence of prolonged damage for example due to ageing or chemotherapy where accumulation of senescent cells leads to chronic inflammation and fibrosis.

Senescent cells have been shown to accumulate with age in primates [74, 66] and the fraction of SA-βgalpositive stained dermal fibroblasts has been shown to increase with age in humans [16]. Known senescence causing triggers are DNA-damage response (DDR) or reactive oxygen species (ROS), oncogenes. The main pathways for cell cyle arrest are

- Activation of p53 which induces p21. p21 inhibits cyclin dependent kinase 2 (CDK2)

- $p16^{INK4a}$inhibits CDK4/6

Both pathways inhibit inactivation of retinoblastoma protein (RB) by keeping it hypophosphorylated. Downstream impediment of transition from G1 to S phase is mediated via E2F transcription factors [51, 17]. Age dependent accumulation of senescent fibroblasts (including secretory phenotype) has been suggested as major contributor to a cancer promoting activated stroma [19]. TGF-β-SMAD signals can participate in development of senescence via p21/CDK2 [26]. It has been reported, that TGF-β/SMAD signalling is reduced in dermal fibroblast from aged donors and that TGF-β signalling is blocked by SMAD7 overexpression [149].

[6] Reactome R-HSA-2559583

Replicative senescence due to telomer loss Replicative senescence initially had been described 1961 by Hayflick and Moorhead as limited proliferative capacity of cells derived from different human fetal tissues [60]. Shortened DNA replica as causative were proposed by Olovnikov 1973 [135, 136] and shortened telomeres were experimentally validated 1990 in ageing fibroblasts [58]. Because *in vivo* ageing dermal fibroblasts only rarely proliferate and only undergo insignificant telomer loss [92, 170], telomere shortening only marginally influences replicative capacity.

In vivo senescent fibroblasts CCN1, an important matricellular effector in wound healing [76] induces cellular senescence in fibroblasts seems to control fibrogenesis in dermal reconstitution [77]. The senescence marker p16 had been shown to be elevated in fibroblast cell lines from aged donors [30].

Metabolic alterations related to cellular senescence Cellular senescence is associated with alterations in metabolic pathways e.g. increased expression of glycolytic enzymes [203]. Alterations in mitochondrial homeostasis promote cellular senescence [202].

Identification of senescent cells Genes indicating cellular senescence are of interest in order to decide whether an age dependent increase of senescent fibroblasts is present. Unfortunately, there is no singular defining cellular marker of senescence. Also the most important marker, SA-βgal, must be detected histochemically [124, 33]. Therefore, cell cycle inhibitors $p16^{INK4a}$ (CDKN2A) and $p21^{Cip1}$ (CDKN1A, WAF1) are in use here [26]. From known mechanisms, a table of candidate genes for detection of senescent cells can be derived and is shown in Table 9.

HGNC	Gene ID	Reference	logCPM	logFC	FDR
CDKN2A	ENSG00000147889	[168]	3.49	0.29	1.00
CDKN1A	ENSG00000124762		8.89	-0.03	1.00
IL1A	ENSG00000115008	[126]			
IL6	ENSG00000136244	[40]	4.46	-0.55	1.00
CCL2	ENSG00000108691	[114]	5.50	0.15	1.00
CCL8	ENSG00000108700	[175]			
CCL3	ENSG00000277632				
CCL4	ENSG00000275302	[175]			
MMP1	ENSG00000196611		9.58	-0.68	1.00
MMP3	ENSG00000149968		7.83	-1.18	1.00
MMP10	ENSG00000166670				
TGFB1	ENSG00000105329	[179]	6.79	0.32	0.93
CSF2	ENSG00000164400	[21]			

Table 9: Cellular senescence indicating genes

For these genes, no increase in expression can be asserted in our data. To demonstrate this, we added logFC FDR values to Table 9 and show data for CDKN2A in Fig 12 as example. For Male individuums, samples from Gluteal region rather indicate a decreased expression of CDKN2a.

Senescence-Associated Secretory Phenotype (SASP) Proteins commonly secreted by senescent cells from different origings define the senescence-associated secretory phenotype (SASP) [7] [27]. SASP secreted proteines include Interleukins (IL1, IL6, IL7, IL8), Chemokines (CCL2 (MCP1), CCL8 (MCP2), CCL3 (MIP-1α) and CCL4 (MIP-1β)), Proteinases (MMP1, MMP3, MMP10), TGF-β and GM-CSF [124, 44, 27]. SASP induces *paracrine* senescence on adjacent cells (e.g. via TGF-β)[1] and *autocrine* reinforcement of senescence [2]. Autocrine TGF-β has

[7] Reactome R-HSA-2559582

Fig 12: CPM values for gene CDKN2a

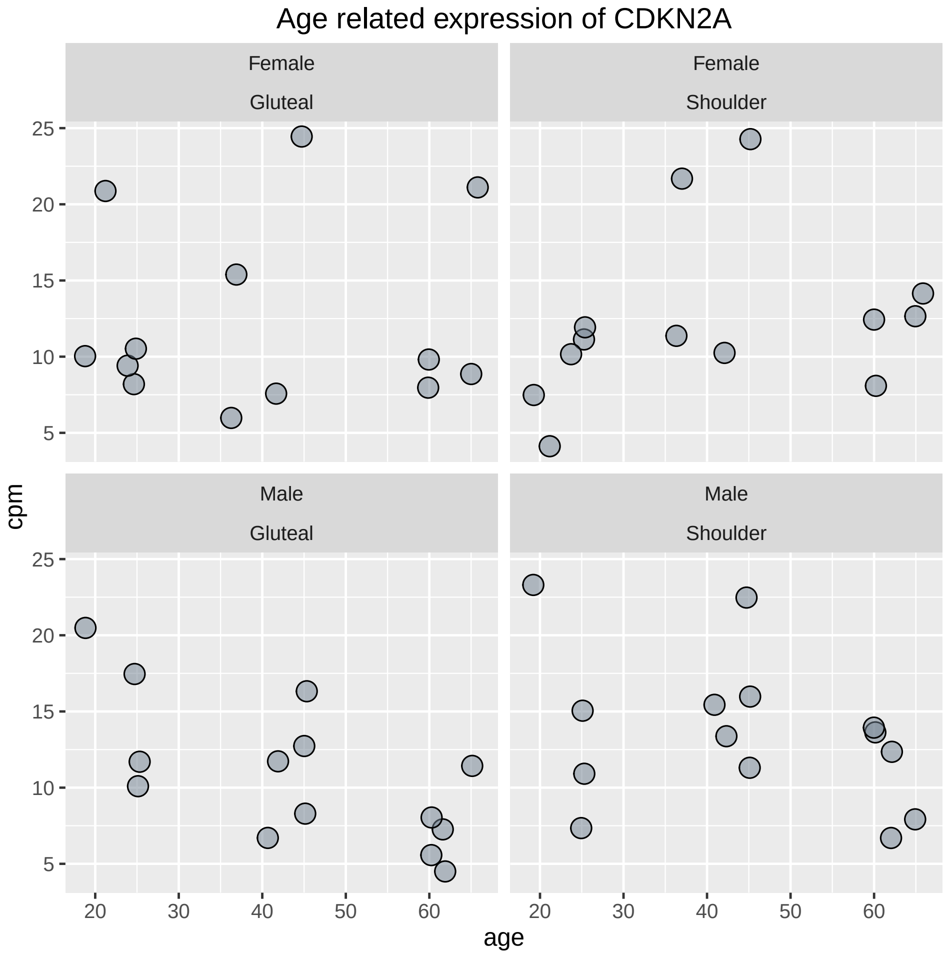

been shown to regulate collagen type I production via CTGF in dermal fibroblasts[149]. Pro-inflammatory and chemoattractant molecules create an inflammatory environment capable of removing damaged cells.

HGNC	Gene ID	Reference	logCPM	logFC	FDR
IL6	ENSG00000136244	[27]	4.46	-0.55	1.00
IL8	ENSG00000169429	[27]			
CSF2	ENSG00000164400	[27]			
CXCL-1	ENSG00000108702	[27]			
CXCL-2	ENSG00000108691	[27]	5.50	0.15	1.00
CXCL-3	ENSG00000163734	[27]			
ICAM-1	ENSG00000090339	[27]	5.48	-0.08	1.00
TNFRSF11B	ENSG00000164761	[27]	7.62	-0.36	1.00
CCL2	ENSG00000108691	[27]	5.50	0.15	1.00
CCL8	ENSG00000108700	[27]			
CCL13	ENSG00000181374	[27]			

Table 10: SASP indicating genes

For these genes, no increase in expression can be asserted in our data. To demonstrate this, we added logFC FDR values to Table 10 and show data for ICAM-1 in Fig 13 as example.

Fig 13: CPM values for gene ICAM-1

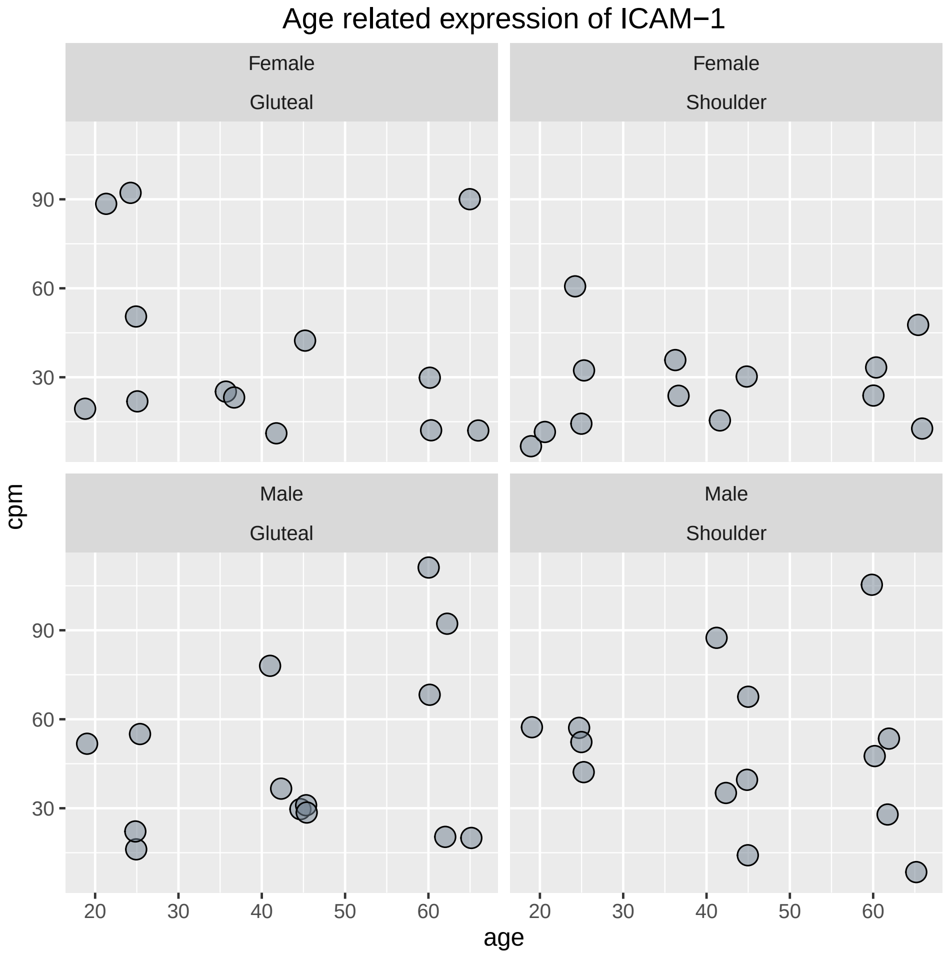

7.1 Age related DE genes in literature

A variety of published studies provide knowledge on age related physiological alterations in human dermal fibroblasts. In order to relate our findings with previous observations, we list genes for which age related differential expression in human dermal fibroglasts has been shown.

HGNC	Gene ID	Reference	logCPM	logFC	FDR
COL1A1	ENSG00000108821	[177, 149]	14.25	0.45	1.00
COL1A2	ENSG00000164692	[177, 149]	13.88	0.16	1.00
CDKN2A	ENSG00000147889	[16]	3.49	0.29	1.00
CDKN1A	ENSG00000124762	[16]	8.89	-0.03	1.00
E2F1	ENSG00000101412	[16]	3.69	-0.04	1.00
CCNA2	ENSG00000145386	[16]	5.06	0.23	1.00
CCNB1	ENSG00000134057	[16]	5.27	0.19	1.00
CCND1	ENSG00000110092	[16]	9.76	0.27	1.00
TERF2	ENSG00000132604	[16]	4.24	0.05	1.00
IGFBP7	ENSG00000163453	[16]	8.03	0.95	0.81
GADD45A	ENSG00000116717	[16]	5.28	0.39	0.72
CALR	ENSG00000179218	[16]	9.98	0.09	1.00
T-Box 3	ENSG00000135111	[16]	5.49	0.29	1.00
TGFB1	ENSG00000105329	[149]	6.79	0.32	0.93
CTGF	ENSG00000118523	[149]	10.05	1.33	0.79
MMP1	ENSG00000196611	[149]	9.58	-0.68	1.00
JUN	ENSG00000177606	[149, 42]	8.54	-0.03	1.00
ITGA2	ENSG00000164171	[149, 42]	3.58	0.10	1.00
ITGB1	ENSG00000150093	[149]	11.32	0.12	1.00
LIG4	ENSG00000174405	[82]	3.70	0.02	1.00
XRCC4	ENSG00000152422	[82]	2.26	0.09	1.00

Table 11: Genes with known age related differential expression in dermal fibroblasts

Collagens

A well kown property of *in vivo* aged dermal fibroblasts is the reduced expression of collagen.

Genes associated with collagen production

For TGF-β1 and CTGF, a reduced mRNA expression in *in vivo* aged dermal fibroblasts has been shown [149].
For MMP1, an increased expression in *in vivo* aged dermal fibroblasts has been shown. But also, no consistent difference in has been found in cultured fibroblasts from young or aged donors [42].
For all three genes, TGF-β1, CTGF(CCN2) and MMP1, no consistent change in gene expression (either age related or location related) is present in our gene expression data.

Fig 14: CPM values for gene COL1A1

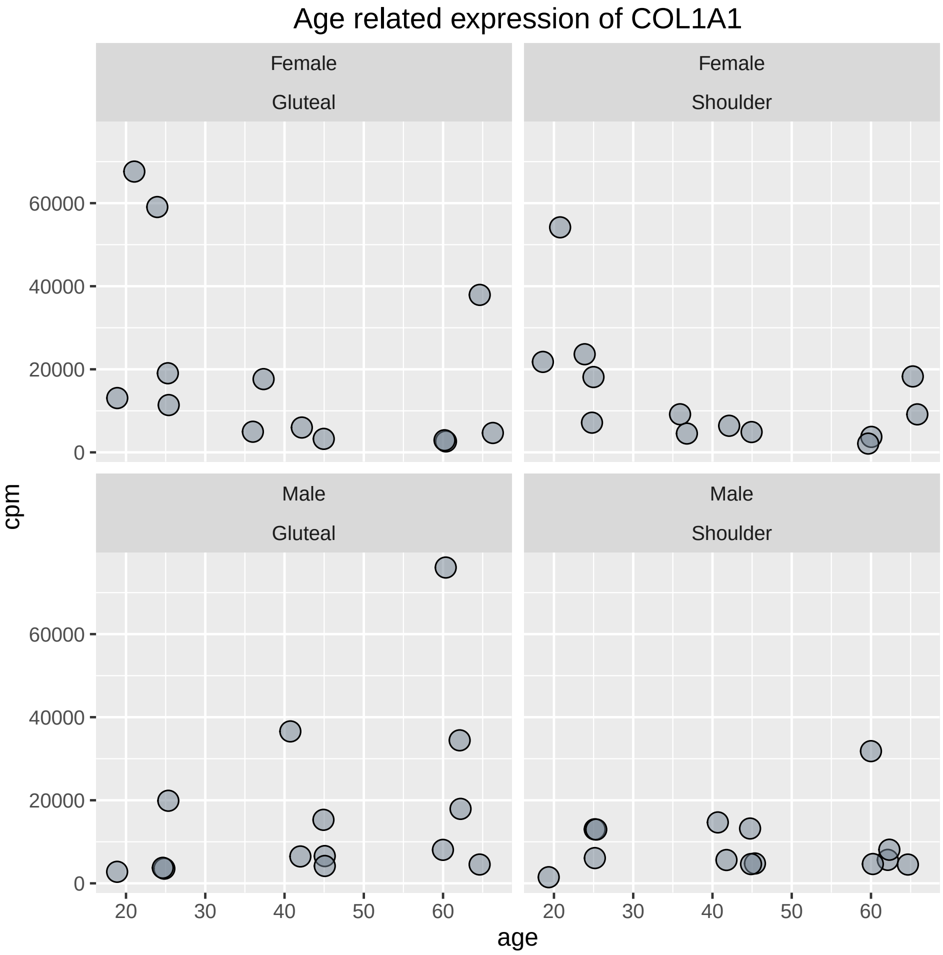

Fig 15: CPM values for gene TGF-β1

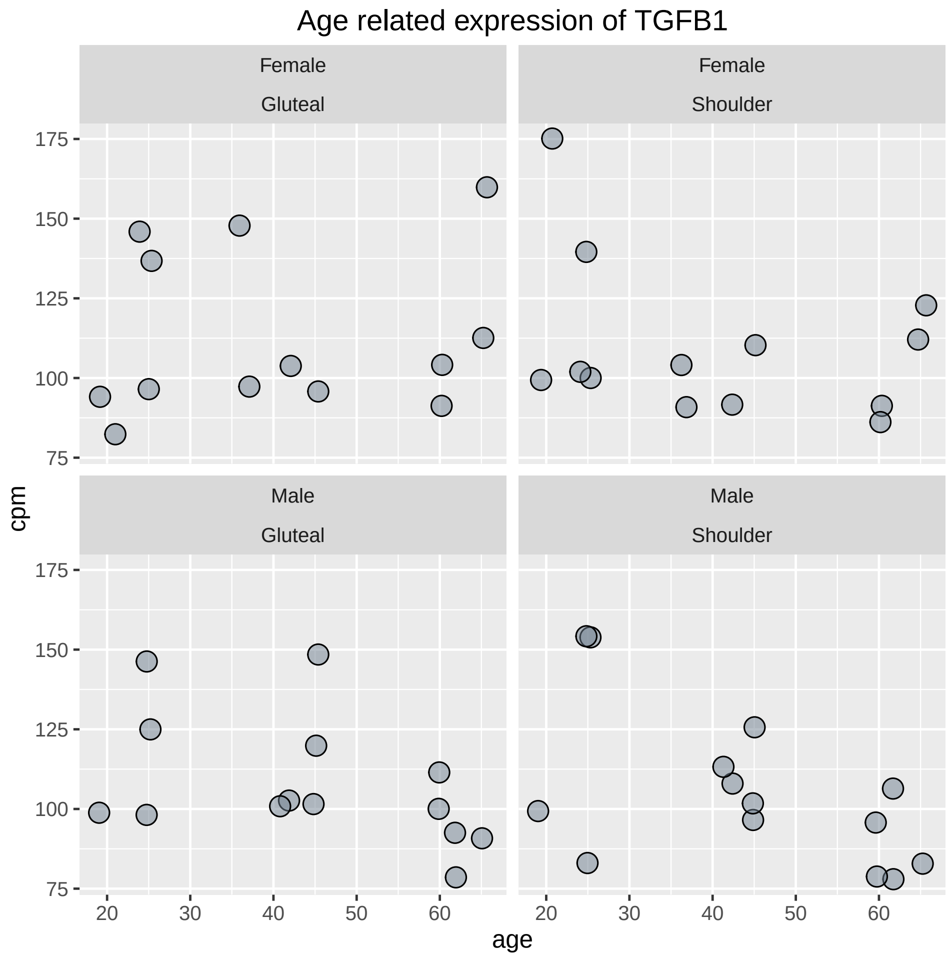

Fig 16: CPM values for gene CTGF (CCN2)

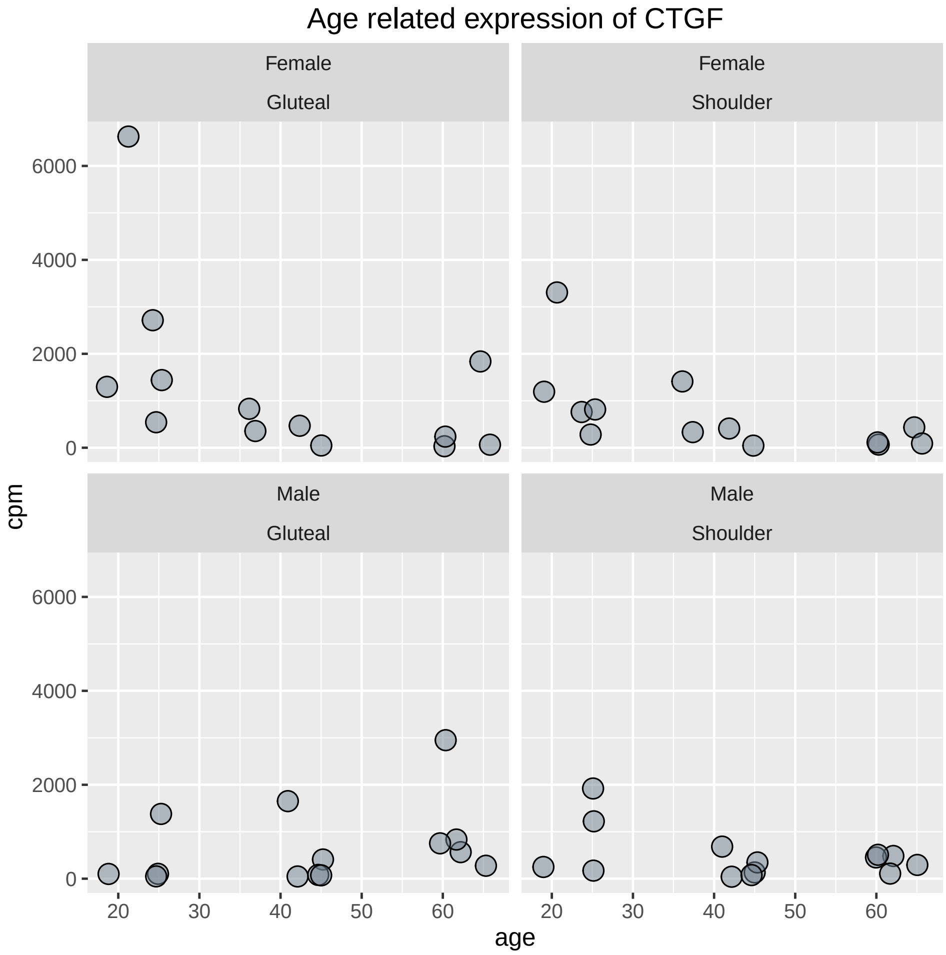

Fig 17: CPM values for gene MMP1

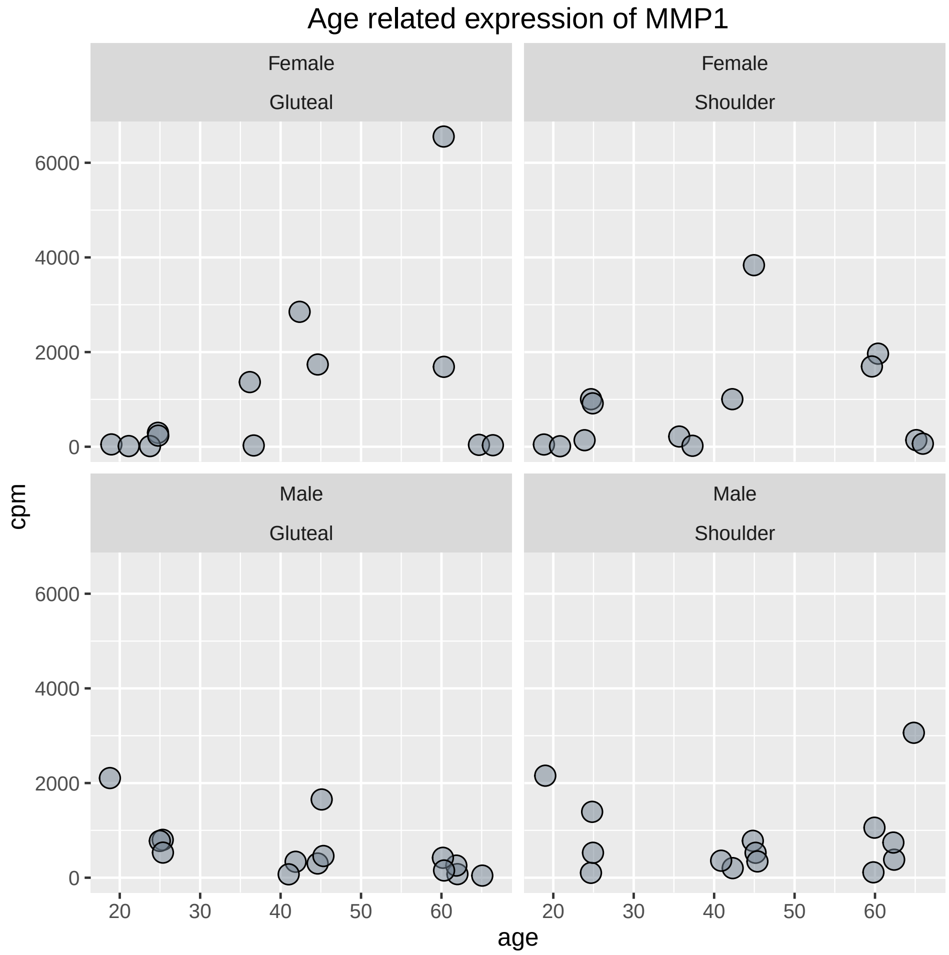

7.2 Results from expression analysis for high expressed genes

Gene expression levels reflect tissue specifity[7]. We therefore trace high expressed genes in our fibroblast samples. Genewise counts per million (CPM) estimates were obtained EQLF procedure on all age groups.
High expressed genes (CPM >1000) are listed in Table 12 (24 genes).

Gene Name	Description	CPM
FN1	fibronectin 1	33869
COL1A1	collagen, type I, alpha 1	16712
COL1A2	collagen, type I, alpha 2	14508
VIM	vimentin	10170
DCN	decorin	10017
THBS1	thrombospondin 1	7787
COL6A2	collagen, type VI, alpha 2	7626
MT-CO1	mitochondrially encoded cytoch	6315
EEF1A1	eukaryotic translation elongat	5954
COL3A1	collagen, type III, alpha 1	5617
COL6A1	collagen, type VI, alpha 1	5232
SERPINE2	serpin peptidase inhibitor, cl	4819
COL6A3	collagen, type VI, alpha 3	4181
MMP2	matrix metallopeptidase 2	3519
CTSB	cathepsin B	3329
SPARC	secreted protein, acidic, cyst	3108
FBLN1	fibulin 1	2772
FLNA	filamin A, alpha	2725
ACTB	actin, beta	2667
VIM-AS1	VIM antisense RNA 1	2665
FSTL1	follistatin-like 1	2650
CTSK	cathepsin K	2596
ACTG1	actin gamma 1	2546
TIMP2	TIMP metallopeptidase inhibito	2507

Table 12: High expressed genes (CPM > 2500)

As can already be assumed from gene list 12, enriched GO terms inclued *extracellular matrix organization* (BP) *wound healing* (BP) and *cell adhesion* (BP) as well as*hemostasis* (BP). Significantly enrichted KEGG pathways include *ECM-receptor interaction*, *Platelet activation* and *TGF-beta signaling pathway* as well as many disease associations.

8 Comparison of DE test procedures

We compare p-values for testing differential gene expression between age groups Young and Old using edgeR Quasi-likelihood F-Tests. Event counting matrices were obtained from TopHat aligned BAM files using two different approaches: summarizeOverlaps (SOV, GenomicAlignments) and readExpSet (RES, spliceSites).

All count matrices were filtered for at least 5 events for each sample. Gene-wise values were unified in RES derived matrix by extracting the minimal p-value for each gene.

Figure 18 shows a direct comparison of p-values. For most genes p-values obtained using RES usually are less or approximately equal to p-values derived using SOV.
In order to compare sensitivity and specificity of RES and SOV basing procedures we compare the number of p-values < 0.01.

```
> table(Sov=ptop$eqlfo < plim, Res=ptop$eqlfr < plim)
```

Fig 18: Comparison of P-values for testing DE (Young vs. Old age group) using edgeR QLF

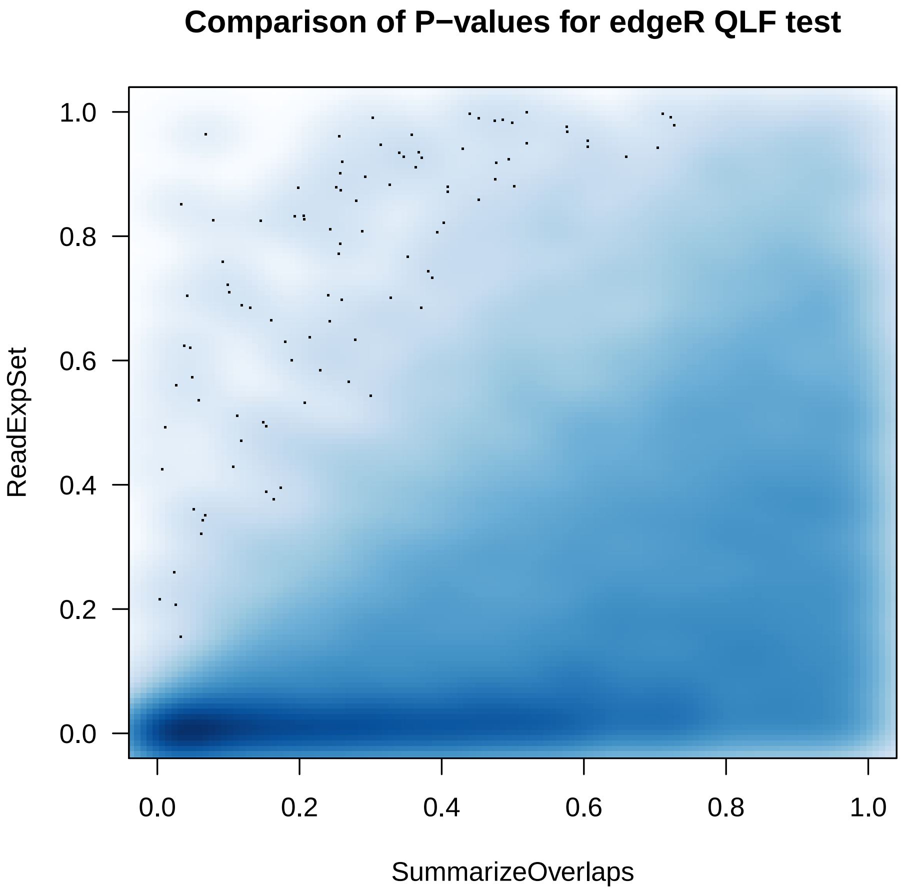


```
        Res
Sov      FALSE TRUE
  FALSE  7960 1929
  TRUE      4  114
```

9 Functional characterization of age VAR genes based on literature

ATOH8

ATOH8 is a basic helix-loop-helix (bHLH) transcription factor (group A) [25]. ATOH8 is evolutionary conserved and widely expressed during embryonic development (in brain, retina, pancreas, kidney and muscle) [36, 35]. ATOH8 gene expression has been reported to be upregulated by BMP2 on mouse embryonic fibroblasts[84]. BMP6, SMAD7, ID1 and ATOH8 are upregulated in mice under iron-enriched diet as confirmed with qPCR revieling high correlation coefficents between each other and liver iron content[88].

PODXL

Podocalyxin, a trans membrane adhesion protein constitutes together with endoglycan the CD34 family of transmembrane proteins[46]. PODXL regulates cell apical morphology [127]. CD34 mainly expressed on early hematopoietic stem cells (and progressively downregulated during maturation[90]) and on vascular endothelial cells[128]. CD34 cells promote lymphocyte adhesion to vascular endothel in lymph tissue but also blocks cell adhesion [128]. PODXL expression

on tumor cells has been associated with impaired prognosis in many tumor types, for example gastric cancer [97] or pancreatic cancer [158] High PODXL expression is associated with poor differentiation in colon cancer[85]. Its anti-adhesive functions have been associated with increased tumor expansion property and elevated MAPK activation[161]. Podocalyxin has been identified as major sialoprotein of renal glomerular epithelial cell [89]. Increased MMP levels are induced by PODXL (which is often present in increased cellular invasion) [161]. PODXL is a downstream target of TP53 [161]. PODXL may have a role in development of Focal segmental glomerulosclerosis [48].

SNAI1

SNAI family of transcription factors are involved in developmental processes and cancer genesis [29]. Control of cell motility is an important conserved function of Snail genes [64]. Expression of SNAI1 is mediated via TGF-β/SMAD signalling and plays a central role in epithelial-mesenchymal transition [119] SNAI1 transcription factor induces cancer cell invasion [28]. SNAI1 is a zinc finger protein important during formation of mesoderm [111] during gastrulation [140] and CNS development [64]. SNAI1 represses E-cadherin. Downregulated E-cadherin is associated with acquisition of metastatic cancer potential [9]. SNAI1 induced E-cadherin expression correlates with cancer progression [64]. SNAI1 is expressed in fibroblasts[9].

ID3

ID3 is a member of the inhibition of differentiation (ID) family. Physiology of ID genes is described for gene ID1 9. ID3 and ID1 have similar expression patterns in cardiovascular tissues [105].

SPHK1

Sphingosine kinase 1 katalyses production of intracellular sphingosine-1-phospate (S1P) from sphingosine [139]. Expression of SPHK1 is upregulated by TGF-βin dermal fibroblasts [193]. S1P influence cell growth and survival of cancer cells [139]. Inhibition of SPHK1 inhibits cell growth and induced apoptosis in human histiocytic leukemia cells [139]. Increased SPHK1 activity promotes cell growth in fibroblasts[134]. SPHK is upregulated in cancer [139].

ID1

ID (inhibitor of differentiation) are evolutionary conserved helix-loop-helix (HLH) proteins [105]. Id assemble heterodimers with basic HLH (bHLH) monomers. Due to missing DNA binding region, bHLH are inactivated[171]. ID proteins generally are ubiquitously expressed during embryonic development and expression decreases with cellular differentiation and is associated with exit from cell cycle [?]. Four ID proteins (ID1 to ID4) are identified which mostly have overlapping patterns of expression [105]. Through functional inhibition of bHLH transcription factors, ID regulate genes associated with cellular differentiation[171].
Expression of ID is down regulated during cellular differentiation and senescence in different cell types (including fibroblasts)[171]. ID1 knockdown results in preterm cellular senescense [200] and ID1 expression promotes fibroblast growth [105, 146]. ID1, ID2 and ID3 expression prolong lifespan of cultured keratinocytes [4].

Functional association of ID1 with cellular differenziation / senescence ID proteins potentially suppress induction of senescent phenotype via inhibition of cyclin dependent kinase inhibitors p16 and p21. ID1 silences E47 (TCF3 transcription factor) and thereby via interaction with E47, ID1 expression down-regulates p16$^{\mathrm{INK4a}}$expression level[200]. p16$^{\mathrm{INK4a}}$expression increases activation of pRB via inhibition of CDK4/6 (cyclin dependent kinase). [26]. Inhibition of CDK2 and CDK4/6 promotes conversion of pRB into phosphorylated (= inactive) form[17]. Keeping pRB in hypo-phosphorylated form is a crucial step for induction of senescence[124]. In

effect, down-regulation of ID1, promotes cellular senescence.

Cyclin dependend kinase inhibitor p21 is inhibited by ID1 via bHLH transcription factor E2A[146]. E2A induced gene expression leads to cell differentiation in multiple cell types[146]. ID1 inversly correlates with p16^{INK4a}expression [200]. In senescent cells, upregulated p16^{INK4a}depends on increased Ets1 expression (and therefore on the absence of ID1)[132]. Ets1 and Ets2 transcription factors activate p16^{INK4a}promoter [132] and can be inactivated by ID1[197].

Regulation of ID by BMP/SMAD BMP has been shown to induce expression of ID1 in various tissues [105, 87] and is a direct target of BMP/SMAD signals [87]. ID1 expression is repressed by TGF-β[159]. Involvement of ID1 in TGF-β signalling pathways indicates close functional relation intracellular signalling by SMAD 9.

ERRFI1 (MIG-6)

ERRFI1 is a cytosolic adaptor/scaffold protein [5]. MIG-6 expression positively correlates with apoptotic cell death [5]. Knockdown of ERRFI1 increases cellular proliferation and invasion [117]. ERRFI1 has been found to be down-regulated in diverse cancer types [117]. Human fibroblast senescence is associated with up-regulation of ERRFI1 [117]. MIG-6 provides negative feedback for EGFR[117] MIG-6 is expressed in fibroblasts [70]

PENK

PENK (preproenkephalin) is a precursor of the endogenous opioids Met- and Leu-enkephalin [12]. Additionally to analgetic effects, opioids effect cell survival and proliferation[172]. Hypermethylated CpG sites located in PENK have been found to be associated with advanced age in middle aged individuals [83].

SEPT5

Septins belong to the phosphate-binding loop (P-loop) proteins and polymerize into oligomer protein complexes potentially forming filaments in the cell cortex and as such are regarded as part of the cytoskeleton [121, 13]. Septins comprise the major component of bud neck filaments during cell separation [121]. SEPT5 is ubiquitously expressed is functionally associated with axon growth, secretion processes on platelets, vesicle locomotion, apoptosis, cell cycle regulation and neoplasia [121, 192, 55]. Septin disturbances have been associated with neurologic disorders and cancer[13]. A proteomic study showed decreased protein amount of septin 2 homolog on cultured human dermal fibroblasts [11].

CPZ

CPZ is a metallo carboxypeptidase identified in human salivary gland [164] [8]. Metallo carboxypeptidases remove hydrolytical C-terminal amino acids from proteins [151, 130]. Pancreatic enzymes belong to metallo carboxypeptidases [151]. CPZ is part of the extracellular matrix [131]

PRPS1

PRPS1 encodes PRS-I enzyme catalyzing steps in purin and pyrimidine nucleotides [118]. PRPS1 mutations may induce chemotherapy resistance in pediatric ALL [102] resulting from reduced feedback inhibition and increasesd purine biosynthesis [123].

[8] Peptidases are divided into endopeptidases (e.g. pancreatic trypsin) and exopepdidases (e.g. carboxypeptidases and aminopeptidases)

MEG3

MEG3 is a long non-coding RNA (lncRNA) regulating gene expression and protein accumulation of p53 [201]. MEG3 regulates TGF-βpathway genes in breast carcinoma cells [120]. MEG3 is expressed in many normal tissues (especially brain, adrenal gland and placenta) and expression is lost in many tumors and tumor cell lines [201]. MEG3 expression inhibits proliferation of tumor cells partially due to MEG3 induced apoptosis [201, 148, 109]. MEG3 expression is reduced in gastric cancer [141]. MEG3 and other lncRNAs have been shown to be up-regulated in ageing mouse liver [188].

CNN1

CNN1 is a calponin isoform, an actin regulatory protein [106]. CNN2 and CNN3 are expressed in fibroblasts [106]. CNN1 expression is highly specific to smooth muscle cells but antibody reactivity has been demonstrated in other cell types [106]. CNN1 expression is regulated by TGF-β[93].

STC1

STC1 is a secreted homodimeric glycoprotein hormone [195, 169]. STC seems to be active in calcium/phosphat homeostatis [195] STC1 has been identified while searching for genes contolling proliferation in senescent fibroblats[23] High STC1 expression is associated with markedly shortened survial in glioma patients [169]. STC1 binds to receptors on outer and inner mitochondrial membranes where it has been shown to increase respiration rate [37]. STC-1 stimulates electron and calcium transport in mitochondria [37]

KIAA1324L

KIAA1434L (EIG121L) is a close homolog of EIG121 [6]. EIG121 has been as estrogene induced gene in humans endometrial hyperplasia and adenocarcinoma [31]. EIG121 was identified from microarray analysis as estrogen induced gene in human endometrium (KIAA1324) and was found to be evolutionary conserved[31].

TRNP1

TRNP1 is a DNA associated protein regulating human cerebral cortex expansion by promotion of neural stem cell self-renewal [166]. TRNP1 protein expression is restriced to neurogenesis [166].

HSPB7

HSPB7 is a member of heat shock 27kDa protein family (member 7) [104] [9]. HSPB7 has been shown to be down-regulated in Renal cell carcinoma (RCC) [104]. Some HSPB7 (HSP27) mutations are cause Charcot-Marie-Tooth disease [39]. HSPB7 inhibits apoptosis by inhibition of caspase activation in cytosol [15]. HSP27 is associated with glial fibrillary acidic protein and viementin in astrocytoma [144]. HSP27 protein content of human skeletal muscle inceases with age [86]

PRRX2

PRRX2 (Prx2, EC 1.11.1.15), peroxiredoxin reduces peroxides[150]. Six Prx isoforms exist [152]. Expression of Prx2 is inversely correlated with metastatic potential of melanoma [100]. Expression of PRRX2 gene is induced by TGF-β[75]. Damage accumulation from ROS plays a major role in ageing and development of age relate genes. ROS inactivation by antioxidant enzymes (e.g. catalase, glutathione peroxidase and peroxiredoxins) is the most important protective mechanism [189]. Peroxiredoxins have been identified in organisms of all kindoms [189].

[9] Heat shock proteins protect unfolded proteins from aggregation

PRRX2 has been shown to interact with p66Shc keeping p66Shc inactive. Knockdown of p66Shc has been shown to decrease ROS levels and 30 % extended lifespan in rodents[50]. Periredoxins have effect on cellular proliferation and differentiation, apoptosis and resistance to chemotherapy and radiotherapy[189] Most human tumors overexpress PRXs compared to normal tissues [189]. PRX2 is often present in colorectal carcinoma and expression correlates with pathological features [189].

SMAD7

SMAD proteins are the major intracellular effectors for the TGF-β superfamily [154]. TGF-β type I phosphorylates members of the receptor-regulated Smads (R-Smads: SMAD -1, -2, -3, -5 and -8). [154]. A separate class of SMAD molecules (I-SMADS: SMAD -6 and -7) inactivate TGF-β receptor by blocking receptor kinase domain [154, 59]. SMAD genes were identified when genes mediating tumor growth inhibitory effects of TGF-β were searched[153]. SMAD7 has been found to be lower expressed in aged epidermis and dermis [56]. SMAD7 inhibits stimulation of type I procollagen expression by CTGF (connective tissue growth factor) [149]. Decreased CTGF expression induces collagen loss in aged human skin [149]. Therefore, SMAD7 down regulation should lead to increase collagen type I expression. SMAD7 mRNA is significant increased in aged and photoaged skin [56]

.

FAM83G

FAM83G (PAWS1=protein interacting with SMAD1) has been identified as protein forming complexes with SMAD1 while searching for regulators in BMP pathway [183]. FAM83G is phosphorylated by BMP but does not change SMAD1 phosphorylation by BMP [183]. FAM83G regulates expression of some BMP dependent genes as well as BMP independend genes [183]. BMP-1 is a metalloproteinase and BMP 2-4 are members of TGF-β family transducing signals via type I and II serine/threonine kinase receptors [87]. FAM83G is a substrate of BMP (TGF-β) type I *receptor* kinase [183]. FAM83G interacts with SMAD1. [183]. BMP2 stimulation increases ID1 (mRNA) expression which is mediated by SMAD1 and SMAD4 [108].
BMP2-SMAD1 is involved in DNA damage response Atm-p53 [24] which is part of senescense signalling pathway [26] and where loss of function induces tumorigenesis [24]. This could be a mechanism by wich FAM83 is involved in induction of cellular senescence but clear data therefor seems to be absent.

MAD genes are involved in TGF-β signaling [69] Common in human cancers are abrogated growth inhibitory effects of TGF-β[153]. BMP2 activation results in nuclear FAM83G (SMAD1) accumulation [69]. BMP-Smad1 signalling pathway is important in tissue development and homeostasis, DNA damage response and tumor suppression[24]. DNA damage response of BMP-SMAD1 occurs via Atm-p53 kinases, central actors in DNA damage response [24]. Therein, SMAD1 directly interacts with p53[24]. A proposed mechanism for BMP induce p53 stabilation under genotoxic stress is shown in [24] (Fig. 7g). Oncogene Ras potentially induces cellular senescence [32] for which Bmps2-Smad1 signal is essential [84].

DDR1

Discoidin receptor thyrosine kinases DDR1 (CD167, RTK6) and DDR2 are collagen receptors which have been found on fibroblasts [156, 191, 181]. DDR (discoidin domain receptors) are evolutionary conserved [182] and involved in regulation of cellular adhesion and proliferation and in matrix remodeling [182]. DDR seem mainly to sense extracellular collagen and are involved in proliferation, collagen degradation (via MMPs) and cellular migration [63, 43]. DDR interacts directly with collagen type I [181]. Collagen interaction induces delayed kinase activation [181]. Collagen I induces expression of DDR1 on fibroblasts[155]. Soluble extracellular domains of DDR1 and DDR2 inhibit collagen production in mouse osteoblasts[43]. Distinct groups of receptor tyrosin kinases (RTK, e.g. fibronectin, immunoglogulin, cadherin, epidermal

growth factor, kringle-repeats) regulate cellular processes like cell differentiation, growth and survival as well as migration and metabolism [181]. Discoidin Dromain form a subgroup of RTK [182]. Characteristic for DDR is homology to discoidin [181]. DDR1 is expressed in brain, on keratinocytes and kidney epithelia colonic epithel, lung epithel and in thyroid follicels and langerhans islets [180]. DDR1 is overexpressed in many human tumors [180]. p53 overexpression and γ irradiation can induce DDR1 expression [180]. DDR1 is expressed in five (?) isoforms [182].

PPP1R3C

Gene PPP1R3C codes for a regulatory subunit of protein phosphatase-1 (PP1) [10]. PPP1R3C functions as glycogen-targeting subunit [11]. [34] [157]

EVA1A

EVA1A is a lysosome and ER (endoplasmic reticulum) associated membrane protein [184]. Over-expression of EVA1A (TMEM166) results in high levels of autophagosome formation [61, 184]. Cellular death induced by EVA1A shows autophagic and apoptotic characteristics[184]. EVA1A is down-regulated in cancer tissues and EV1A inhibits tumor proliferation by autophagy[103].

CRISPLD2

CRISPLD2 (cysteine-rich secretory protein containing LCCL domain 2) binds LPS protein. CRISPLD2 serum levels are elevated in patients with septic shock and upregulation protects mice against LPS related mortality [185]. Inflammation blocking effects are mediated via MiR155 [198]. CRISPLD2 is released from Granulocytes, mononuclear cells, NK cells and T cells [186].

RP11-309L24.6

According to Ensembl: Known unprocessed pseudogene (no coding exon).
Transcript: RP11-309L24.6-001 ENST00000469533

ZNF385D

Gene ZNF385D related SNPs have been associated with Schizophrenia-Negative symptoms [190].

FGFRL1

Gene FGFRL1 is a transmembrane receptor for Fibroblast Growth factor (FGF) [173]. FGFR1 increases cancer cell proliferation (via prevention of cell cycle arrest in G_1/G_0) [174]. FGFR1 is target of tumor suppressive miR-210[174]. Overexpression of FGFRL1 repressed cell proliferation [173]. Intronic FGFR2 SNPs are associated with Breast cancer risk. Variants of osther FGF recptors (especially FGFRL1) is not associated with breast cancer risk [3]. FGFR1 is target of miR-210 [174].

CKB

Gene CKB, brain type creatine kinase, is a metallocarboxypepdidase [12]. Inactivation of CKB in bronchial epithelial cells induces cellular senescence [57]. Preterm cellular senescence is an important effector in pathogenesis of COPD [57]

[10] `http://www.ncbi.nlm.nih.gov/gene/5507`
[11] `http://www.uniprot.org/uniprot/Q9UQK1`
[12] IUBMB Enzyme Nomenclature: EC 3.4.17.2

FILIP1L

Gene FILIP1L (Filamin A interacting protein 1-like) has been shown to be upregulated in anti-angiogenesis therapy of cancer [94]. FILIP1L overexpression inhibits endothelial cell proliferation and increases apoptosis [94]. FILIP1L promotes cellular proliferation in normal and tumor cells[18]. FILIP1L may be a tumor suppressor gene in ovarian cancer [129]. FILIP1L (DOC-1 = downregulated in ovarian cancer 1) expression is inversly correlated with invasive potential of ovarian cancer [18]. Overexpression of FILIP1L results in inhibion of cell division and increased apoptosis in ovarian cancer[18]. High expression of FILIP1L in tumor vessels diminishes tumor growth [94] It has been proposed that FILIP1L has an important role in early endothelial response to angiogenesis inhibitors [94].

GJA1

Hexameric GJA1 (gap junction protein connexin43, Cx43) form intercellular channels constituting Gap Junctions [116]. Gap junctions allow direct exchange of small molecules between adjacent cells [53]. GJA1 is expressed in dermal keratinocytes and fibroblasts [96]. Mutations of GJA1 have been related to oculodentodigital dysplasia (ODDD) [96]. Connexins play an important role in autophagosome formation [10].

ENC1

ENC1 (Ectoderm and neural cortex 1) was identified in murine early development of nervous system [68] and is expressed in human CNS [67]. ENC1(NRP/B) binds actin cytoskeleton and is intracellularly located in adjacency of actin [68]. ENC1 expression is up-regulated in 66% of colorectal carcinomas [45]. Expression of ENC1 has been associated wich suppression of differentiation and increased proliferation in colon cancer cells [45]. ENC1 is increased in adipocyte differentiation is proposed to participate in cellular reshaping [199] Gene ENC1 (PIG10) is member of p53 induced genes [145].

SH2D4A

Gene SH2D4A (SH2 domain containing 4A) belongs to the SH2 domain containing gene family [13]. SH2D4A is ubiquitously expressed [14]. Gene SH2D4A is an adapter protein expressed on T-cells [98]. Knockdown of SH2D4A in Mice and on T-cells had no functional impact [98]. Adapter proteins are a group of signalling proteins performing transmission of activation signal after stimulation of TCR, BCR or Fc-receptors[101]

ARHGAP23P1

Gene ARHGAP23P1 (Rho GTPase activating protein 23 pseudogene 1) has two transcripts. Transcript 002 (ENST00000567025) is processed.

SERTAD1

Gene SERTAD1 (SEI1, TRIP-Br1, $p34^{SEI\text{-}1}$) is member of a group of proteins active in regulation of transcription and cell cycle [95]. SERTAD1 may promote cell cycling via antagonizing $p16^{INK4a}$[95]. SERTAD1 interacts with SMAD1 and augments BMP signalling in mouse embryonic hearts[142]. TRIP-Br1 is predominantly located in cell nucleus and interacts with bromodomain conaining proteins[95]. TRIP-Br (Transcriptional Regulator Interacting with the PHD-Bro- modomain) interacts with CDK4 which makes cyclin-D/CDK4 complexes inert to inhibition by $p16^{INK4a}$[95, 41]. Overexpression of SERTAD1 in mouse fibroblasts causes tumor formation in nude mice. suppression of SERTAD1 expression inhibits cellular proliferation (S-phase entry) [196]. Low serum level of SERTAD1 promote cell growth by inhibiting

[13] `http://www.genenames.org/cgi-bin/genefamilies/set/741`
[14] `http://www.uniprot.org/uniprot/Q9H788`

p16^{INK4a}function[95]. Bromodomain/acetyl-lysine binding is an important mechanism in gene transcription[122]. TRIP-Br1 and TRIP-Br2 contain bromodomain binding domains and act as transcriptional activators[95].

FGF13

Fibroblast growth factors (FGFs) are associated with cell division and survival [162]. Fibroblast growth factor homologous factors (FHFs) are a subfamily of fibroblast growth factor (FGF) family [162]. FHFs (FGF11–14) modulate voltage-gated Na^+ channels and are expressed in cardiomyocytes [65] and neuronal cells [162] (excitable cells). FHF are expressed in neuronal cells [162] FGF13 belongs to iFgf subfamily[72]. FHFs are intracellular signaling proteins [160]. Upregulated FGF12 has been shown to mediate resistence to platinum in cervical cancer [133]. Four fibroblast growth factor receptors (FGFRs) exist[38]. FGFG are tyrosine kinases consisting of an extracellular, a transmembrane and a cytoplasmatic domain. The latter contains the catalytic centre [38]. FHFs are functionally not related to FGFs [52]. Ligands for FHFs are intracellular domains of voltage-gated sodium channels (VGSCs) (and to IB2)[52]. FHF are mainly expressed in post-mitotic neurons[52].

EHD1

EHD (Eps15 homology domain) proteins regulate endocytic recycling of membrane receptors[125, 49]. EDH1 acts as gatekeeper in promoting ERC-to-plasma-membrane recycling [125]. EHD1 is involved for example in recycling transferrin receptor, MHC class I and II and β 1 integrins. EHD1 also regulates LDL-receptor internalization and modulates insuline like growth factor receptor (IGF1R) signalling [125].
[49].

USP41

USP41 is a ubiquitin-specific protease [15] [147] belonging to Deubiquitating enzymes (DUBs) [165]. Post-translational attached ubiquitin (a small protein) modifies proteins as regulative for cellular proteolysis, DNA repair and cell communcation [62]. Addition of ubiquitin is mediated via ubiquitin-activating enzymes E1/E2/E3 and ubiquitin is removed by DUBs [165, 62]. For DUBs groups exist: USP, OTU, MJD and UCH. USP41 is member of USP group. DUBs have been associated wich many disease associated pathways like Immunity, growth factors, cell cycle, apoptosis and DNA repair [62].

ACSS3

Acyl-coenzyme A synthetases (ACSs) catalyze the fundamental, initial reaction in fatty acid metabolism. ACSS3[16], a Acetyl-CoA short chain synthetase activates fatty acids by building thioesters with CoA [22], an important metabolic entry point [187]. ACSS3 is a short-chain acyl-coenzyme A synthetase wich activate acetate, propionate and butyrate (C2-4 fatty acids) [143]. ACSS3 is mainly expressed in lung and located in mitochondria[143].

BACE2

Generation of amyloid β-peptide (Aβ) depends on proteolysis of amyloid precursor protein(APP)[178] by two proteases: β-secretase (BACE) and γ-secretase[178]. BACE (β-site APP-cleaving enzyme) [167] are involved in Down Syndrome and Alzheimer disease. BACE2 has been identified during search for genes involved in Down Syndrome [163]. Aβ (amolyoid β-peptide) originates by sequential cleavage by β-secretase (BACE=β-site APP-cleaving enzyme 1) and γ-secretase [167]. APP mutations cause autosomal dominant Alzheimer disease[194]. Brain Aβ is mainly produced by BACE1[167].

[15] Putative ubiquitin carboxyl-terminal hydrolase 41 (EC:3.4.19.12) http://www.uniprot.org/uniprot/Q3LFD5
[16] http://www.uniprot.org/uniprot/Q9H6R3

ADGRL4

ADGRL4 (ELTD1) is G-protein-coupled receptor[112]. ADGRL4 regulates vascular sprouting via tip cell specification/activity [112]. Silencing of ADGRL4 results in markedly reduced tumor growth and dramatic increase in survival in mouse ovarian carcinoma [112].

ROBO1

Roundabout (Robo) has been identified as molecule guiding axon path, axon branching and neuron migration [14, 71]. ROBO are transmembrane receptors belonging to immunoglobin superfamily, usually consisting of an extracellular domain, five Immunoglobulin-like motifs and three fibronectin type 3 domains [71]. Slit (Slit1, Slit2, Slit3) are extracellular matrix proteins and ROBO it the corresponding cell surface receptor[8]. Roles for Slit-ROBO signalling have been shown for angiogenesis (endothel), development of kidney and heart, leukocyte migration as well as cell cycle, apoptosis, cell adhesion [71, 8]. ROBO1 expression is induced by TGF-β1 [110]. Significant up- and downreulation of Slit/ROBO has been shown for diverse tumors where mostly cell invation and migration is inhibited, but controversial results exist[71]

KCNC4

KCNC4 (Kv3.4) is a voltage-gated Kalium channel[54] and is expressed in cardiomyocytes [137]. KCNC4 has important impact on cell cycle and growth of vascular smooth muscle cells [99, 73]. KCNC4 influences radioresistance of myeloid leukemia cells [138] and is frequently overexpressed during malignant transformation of head and neck squamous cell carcinomas [115].

10 Further information on supplementary material

Tables in S1_File s1_table.xlsx Spreadsheet file containing tables:
Age_MALDR_DE_genes: Listed age related differential expressed genes.
Age_MALDR_GO: Listed enriched GO terms for age VAR genes.
Age_MALDR_KEGG: Listed enriched KEGG pathways for age VAR genes.
Gender_EQLF_DE_genes: Listed gender related DE genes.
Gender_EQLF_DE_GO: Enriched GO terms for gender related DE genes.
Gender_EQLF_DE_KEGG: Enriched KEGG pathways for gender related DE genes.
Location_EQLF_DE_genes: Listed location (UV exposition) related DE genes.
Location_EQLF_DE_GO: Enriched GO terms for location (UV exposition) related DE genes.
Location_EQLF_DE_KEGG: Enriched KEGG pathways for location (UV exposition) related DE genes.

S2 Document. s2_document.pdf PDF file with gene expression data for the 42 age related DE genes.

S3 Document. s3_document.pdf PDF file with 406 tabulated gender related DE genes. Gene expression data for the 25 genes on X and Y chromosome are shown.

S4 Document. s4_document.pdf PDF file with 173 tabulated locaction (UV-exposition) related DE genes. Gene expression data for the 50 genes are shown.

S5 Document. s5_document.pdf PDF file with gene expression data for genes selected based on literture search.

S6 Document. grs_man_sup.pdf PDF file background information on methodology.

References

[1] J. C. Acosta, A. Banito, T. Wuestefeld, A. Georgilis, P. Janich, J. P. Morton, D. Athineos, T. W. Kang, F. Lasitschka, M. Andrulis, G. Pascual, K. J. Morris, S. Khan, H. Jin, G. Dharmalingam, A. P. Snijders, T. Carroll, D. Capper, C. Pritchard, G. J. Inman, T. Longerich, O. J. Sansom, S. A. Benitah, L. Zender, and J. Gil. A complex secretory program orchestrated by the inflammasome controls paracrine senescence. *Nat. Cell Biol.*, 15(8):978–990, Aug 2013.

[2] J. C. Acosta, A. O'Loghlen, A. Banito, M. V. Guijarro, A. Augert, S. Raguz, M. Fumagalli, M. Da Costa, C. Brown, N. Popov, Y. Takatsu, J. Melamed, F. d'Adda di Fagagna, D. Bernard, E. Hernando, and J. Gil. Chemokine signaling via the CXCR2 receptor reinforces senescence. *Cell*, 133(6):1006–1018, Jun 2008.

[3] D. Agarwal, S. Pineda, K. Michailidou, J. Herranz, G. Pita, L. T. Moreno, M. R. Alonso, J. Dennis, Q. Wang, M. K. Bolla, K. B. Meyer, P. Menendez-Rodriguez, D. Hardisson, M. Mendiola, A. Gonzalez-Neira, A. Lindblom, S. Margolin, A. Swerdlow, A. Ashworth, N. Orr, M. Jones, K. Matsuo, H. Ito, H. Iwata, N. Kondo, M. Hartman, M. Hui, W. Y. Lim, P. T. Iau, E. Sawyer, I. Tomlinson, M. Kerin, N. Miller, D. Kang, J. Choi, S. K. Park, D. Noh, J. L. Hopper, D. F. Schmidt, E. Makalic, M. C. Southey, S. H. Teo, C. H. Yip, K. Sivanandan, W. Tay, H. Brauch, T. Bruning, U. Hamann, A. M. Dunning, M. Shah, I. L. Andrulis, J. A. Knight, G. Glendon, S. Tchatchou, M. K. Schmidt, A. Broeks, E. H. Rosenberg, L. J. van't Veer, P. A. Fasching, S. P. Renner, A. B. Ekici, M. W. Beckmann, C. Shen, C. Hsiung, J. Yu, M. Hou, W. Blot, Q. Cai, A. H. Wu, C. Tseng, D. Van Den Berg, D. O. Stram, A. Cox, I. W. Brock, M. W. Reed, K. Muir, A. Lophatananon, S. Stewart-Brown, P. Siriwanarangsan, W. Zheng, S. Deming-Halverson, M. J. Shrubsole, J. Long, X. Shu, W. Lu, Y. Gao, B. Zhang, P. Radice, P. Peterlongo, S. Manoukian, F. Mariette, S. Sangrajrang, J. McKay, F. J. Couch, A. E. Toland, D. Yannoukakos, O. Fletcher, N. Johnson, I. dos Santos Silva, J. Peto, F. Marme, B. Burwinkel, P. Guenel, T. Truong, M. Sanchez, C. Mulot, S. E. Bojesen, B. G. Nordestgaard, H. Flyer, H. Brenner, A. K. Dieffenbach, V. Arndt, C. Stegmaier, A. Mannermaa, V. Kataja, V. Kosma, J. M. Hartikainen, D. Lambrechts, B. T. Yesilyurt, G. Floris, K. Leunen, J. Chang-Claude, A. Rudolph, P. Seibold, D. Flesch-Janys, X. Wang, J. E. Olson, C. Vachon, K. Purrington, G. G. Giles, G. Severi, L. Baglietto, C. A. Haiman, B. E. Henderson, F. Schumacher, L. L. Marchand, J. Simard, M. Dumont, M. S. Goldberg, F. Labreche, R. Winqvist, K. Pylkas, A. Jukkola-Vuorinen, M. Grip, P. Devilee, R. A. Tollenaar, C. Seynaeve, M. Garcia-Closas, S. J. Chanock, J. Lissowska, J. D. Figueroa, K. Czene, M. Eriksson, K. Humphreys, H. Darabi, M. J. Hooning, M. Kriege, J. M. Collee, M. Tilanus-Linthorst, J. Li, A. Jakubowska, J. Lubinski, K. Jaworska-Bieniek, K. Durda, H. Nevanlinna, T. A. Muranen, K. Aittomaki, C. Blomqvist, N. Bogdanova, T. Dork, P. Hall, G. Chenevix-Trench, D. F. Easton, P. D. Pharroah, J. I. Arias-Perez, P. Zamora, J. Benitez, R. L. Milne, A. Berchuck, R. A. Eeles, A. Amin Al Olama, Z. Kote-Jarai, S. Benlloch, A. Antoniou, L. McGuffog, K. Offit, A. Lee, E. Dicks, C. Luccarini, D. C. Tessier, F. Bacot, D. Vincent, S. LaBoissiere, F. Robidoux, S. F. Nielsen, J. M. Cunningham, S. A. Windebank, C. A. Hilker, J. Meyer, M. Angelakos, J. Maskiell, S. Cornelissen, E. van der Schoot, E. Rutgers, S. Verhoef, F. Hogervorst, P. Boonyawongviroj, P. Siriwanarungsan, A. Hein, M. Schrauder, M. Rubner, S. Oeser, S. Landrith, E. Williams, E. Ryder-Mills, K. Sargus, N. McInerney, G. Colleran, A. Rowan, A. Jones, C. of Sohn, A. Schneeweiss, P. Bugert, A. Osorio, A. Barroso, V. Fernandez, M. Gonzalez-Niera, N. Malats, F. Real, B. Herraez, N. Alvarez, E. Diaz, M. Miguel-Martin, L. Bernstein, J. Lacey, S. Wang, H. Ma, Y. Lu, J. Clague DeHart, D. Deapen, R. Pinder, E. Lee, F. Schumacher, P. Horn-Ross, P. Reynolds, D. Nelson, H. Park, H. Ziegler, S. Wolf, V. Hermann, M. Fischer-Bosch, W. Y. Lo, C. Justenhoven, K. Yon-Dschun, C. Baisch, H. P. Fischer, B. Pesch, S. Rabstein, A. Lotz, V. Harth, T. Heikkinen, I. Erkkila, K. Aaltonene, K. von Smitten, N. Antonenkova, P. Hillemanns, H. Christiansen, E. Myohanen, H. Kemilainen, H. Thorne, E. Niedermayr, D. Botwell, G. Chenevix-Trench, A. deFazio, D. Gertig, A. Green, P. Webb, P. Parsons, N. Hayward, P. Webb, D. Whiteman, A. Fung,

J. Yashiki, G. Peuteman, D. Smeets, T. Van Brussel, K. Corthouts, N. Obi, J. Heinz, S. Behrens, U. Eilber, M. Celik, T. Olchers, B. Peissel, G. Scuvera, D. Zaffaroni, B. Bonanni, M. Barile, I. Feroce, M. Tranchant, M. F. Valois, A. Turgeon, L. Heguy, P. S. Yee, P. Kang, K. I. Nee, S. Mariapun, Y. Sook-Ye, D. Lee, T. Y. Ching, N. A. Mohd Taib, M. Otsukka, K. Mononen, T. Selander, N. Weerasooriya, E. Krol-Warmerdam, J. Molenaar, J. Blom, L. Brinton, N. Szeszenia-Dabrowska, B. Peplonska, W. Zatonski, P. Chao, M. Stagner, P. Bos, J. Blom, E. Crepin, A. Nieuwlaat, A. Heemskerk, S. Higham, S. Cross, H. Cramp, D. Conley, C. Luccarini, D. Conroy, C. Baynes, K. Chua, and R. Pilarski. FGF receptor genes and breast cancer susceptibility: results from the Breast Cancer Association Consortium. *Br. J. Cancer*, 110(4):1088–1100, Feb 2014.

[4] R. M. Alani, J. Hasskarl, M. Grace, M. C. Hernandez, M. A. Israel, and K. Munger. Immortalization of primary human keratinocytes by the helix-loop-helix protein, Id-1. *Proc. Natl. Acad. Sci. U.S.A.*, 96(17):9637–9641, Aug 1999.

[5] S. Anastasi, L. Castellani, S. Alema, and O. Segatto. A pervasive role for MIG6 in restraining cell proliferation. *Cell Death Differ.*, 21(3):345–347, Mar 2014.

[6] T. Araki, M. Kusakabe, and E. Nishida. Expression of estrogen induced gene 121-like (EIG121L) during early Xenopus development. *Gene Expr. Patterns*, 7(6):666–671, Jun 2007.

[7] K. G. Ardlie, D. S. Deluca, A. V. Segre, T. J. Sullivan, T. R. Young, E. T. Gelfand, C. A. Trowbridge, J. B. Maller, T. Tukiainen, M. Lek, L. D. Ward, P. Kheradpour, B. Iriarte, Y. Meng, C. D. Palmer, T. Esko, W. Winckler, J. N. Hirschhorn, M. Kellis, D. G. MacArthur, G. Getz, A. A. Shabalin, G. Li, Y. H. Zhou, A. B. Nobel, I. Rusyn, F. A. Wright, T. Lappalainen, P. G. Ferreira, H. Ongen, M. A. Rivas, A. Battle, S. Mostafavi, J. Monlong, M. Sammeth, M. Mele, F. Reverter, J. M. Goldmann, D. Koller, R. Guigo, M. I. McCarthy, E. T. Dermitzakis, E. R. Gamazon, H. K. Im, A. Konkashbaev, D. L. Nicolae, N. J. Cox, T. Flutre, X. Wen, M. Stephens, J. K. Pritchard, Z. Tu, B. Zhang, T. Huang, Q. Long, L. Lin, J. Yang, J. Zhu, J. Liu, A. Brown, B. Mestichelli, D. Tidwell, E. Lo, M. Salvatore, S. Shad, J. A. Thomas, J. T. Lonsdale, M. T. Moser, B. M. Gillard, E. Karasik, K. Ramsey, C. Choi, B. A. Foster, J. Syron, J. Fleming, H. Magazine, R. Hasz, G. D. Walters, J. P. Bridge, M. Miklos, S. Sullivan, L. K. Barker, H. M. Traino, M. Mosavel, L. A. Siminoff, D. R. Valley, D. C. Rohrer, S. D. Jewell, P. A. Branton, L. H. Sobin, M. Barcus, L. Qi, J. McLean, P. Hariharan, K. S. Um, S. Wu, D. Tabor, C. Shive, A. M. Smith, S. A. Buia, A. H. Undale, K. L. Robinson, N. Roche, K. M. Valentino, A. Britton, R. Burges, D. Bradbury, K. W. Hambright, J. Seleski, G. E. Korzeniewski, K. Erickson, Y. Marcus, J. Tejada, M. Taherian, C. Lu, M. Basile, D. C. Mash, S. Volpi, J. P. Struewing, G. F. Temple, J. Boyer, D. Colantuoni, R. Little, S. Koester, L. J. Carithers, H. M. Moore, P. Guan, C. Compton, S. J. Sawyer, J. P. Demchok, J. B. Vaught, C. A. Rabiner, N. C. Lockhart, K. G. Ardlie, G. Getz, F. A. Wright, M. Kellis, S. Volpi, and E. T. Dermitzakis. Human genomics. The Genotype-Tissue Expression (GTEx) pilot analysis: multitissue gene regulation in humans. *Science*, 348(6235):648–660, May 2015.

[8] M. S. Ballard and L. Hinck. A roundabout way to cancer. *Adv. Cancer Res.*, 114:187–235, 2012.

[9] E. Batlle, E. Sancho, C. Franci, D. Dominguez, M. Monfar, J. Baulida, and A. Garcia De Herreros. The transcription factor snail is a repressor of E-cadherin gene expression in epithelial tumour cells. *Nat. Cell Biol.*, 2(2):84–89, Feb 2000.

[10] E. Bejarano, A. Yuste, B. Patel, R. F. Stout, D. C. Spray, and A. M. Cuervo. Connexins modulate autophagosome biogenesis. *Nat. Cell Biol.*, 16(5):401–414, May 2014.

[11] F. Boraldi, L. Bini, S. Liberatori, A. Armini, V. Pallini, R. Tiozzo, I. Pasquali-Ronchetti, and D. Quaglino. Proteome analysis of dermal fibroblasts cultured in vitro from human healthy subjects of different ages. *Proteomics*, 3(6):917–929, Jun 2003.

[12] A. Bottger and B. A. Spruce. Proenkephalin is a nuclear protein responsive to growth arrest and differentiation signals. *J. Cell Biol.*, 130(6):1251–1262, Sep 1995.

[13] A. A. Bridges and A. S. Gladfelter. Septin Form and Function at the Cell Cortex. *J. Biol. Chem.*, 290(28):17173–17180, Jul 2015.

[14] K. Brose, K. S. Bland, K. H. Wang, D. Arnott, W. Henzel, C. S. Goodman, M. Tessier-Lavigne, and T. Kidd. Slit proteins bind Robo receptors and have an evolutionarily conserved role in repulsive axon guidance. *Cell*, 96(6):795–806, Mar 1999.

[15] J. M. Bruey, C. Ducasse, P. Bonniaud, L. Ravagnan, S. A. Susin, C. Diaz-Latoud, S. Gurbuxani, A. P. Arrigo, G. Kroemer, E. Solary, and C. Garrido. Hsp27 negatively regulates cell death by interacting with cytochrome c. *Nat. Cell Biol.*, 2(9):645–652, Sep 2000.

[16] C. Brun, F. Jean-Louis, T. Oddos, M. Bagot, A. Bensussan, and L. Michel. Phenotypic and functional changes in dermal primary fibroblasts isolated from intrinsically aged human skin. *Exp. Dermatol.*, 25(2):113–119, Feb 2016.

[17] J. R. Burke, G. L. Hura, and S. M. Rubin. Structures of inactive retinoblastoma protein reveal multiple mechanisms for cell cycle control. *Genes Dev.*, 26(11):1156–1166, Jun 2012.

[18] E. R. Burton, A. Gaffar, S. J. Lee, F. Adeshuko, K. D. Whitney, J. Y. Chung, S. M. Hewitt, G. S. Huang, G. L. Goldberg, S. K. Libutti, and M. Kwon. Downregulation of Filamin A interacting protein 1-like is associated with promoter methylation and induces an invasive phenotype in ovarian cancer. *Mol. Cancer Res.*, 9(8):1126–1138, Aug 2011.

[19] J. Campisi. Aging, tumor suppression and cancer: high wire-act! *Mech. Ageing Dev.*, 126(1):51–58, Jan 2005.

[20] J. Campisi and F. d'Adda di Fagagna. Cellular senescence: when bad things happen to good cells. *Nat. Rev. Mol. Cell Biol.*, 8(9):729–740, Sep 2007.

[21] M. A. Cantrell, D. Anderson, D. P. Cerretti, V. Price, K. McKereghan, R. J. Tushinski, D. Y. Mochizuki, A. Larsen, K. Grabstein, and S. Gillis. Cloning, sequence, and expression of a human granulocyte/macrophage colony-stimulating factor. *Proc. Natl. Acad. Sci. U.S.A.*, 82(18):6250–6254, Sep 1985.

[22] L. F. Castro, M. Lopes-Marques, J. M. Wilson, E. Rocha, M. A. Reis-Henriques, M. M. Santos, and I. Cunha. A novel Acetyl-CoA synthetase short-chain subfamily member 1 (Acss1) gene indicates a dynamic history of paralogue retention and loss in vertebrates. *Gene*, 497(2):249–255, Apr 2012.

[23] A. C. Chang, J. Janosi, M. Hulsbeek, D. de Jong, K. J. Jeffrey, J. R. Noble, and R. R. Reddel. A novel human cDNA highly homologous to the fish hormone stanniocalcin. *Mol. Cell. Endocrinol.*, 112(2):241–247, Aug 1995.

[24] J. F. Chau, D. Jia, Z. Wang, Z. Liu, Y. Hu, X. Zhang, H. Jia, K. P. Lai, W. F. Leong, B. J. Au, Y. Mishina, Y. G. Chen, C. Biondi, E. Robertson, D. Xie, H. Liu, L. He, X. Wang, Q. Yu, and B. Li. A crucial role for bone morphogenetic protein-Smad1 signalling in the DNA damage response. *Nat Commun*, 3:836, 2012.

[25] J. Chen, F. Dai, A. Balakrishnan-Renuka, F. Leese, W. Schempp, F. Schaller, M. M. Hoffmann, G. Morosan-Puopolo, F. Yusuf, I. J. Bisschoff, V. Chankiewitz, J. Xue, J. Chen, K. Ying, and B. Brand-Saberi. Diversification and molecular evolution of ATOH8, a gene encoding a bHLH transcription factor. *PLoS ONE*, 6(8):e23005, 2011.

[26] B. G. Childs, M. Durik, D. J. Baker, and J. M. van Deursen. Cellular senescence in aging and age-related disease: from mechanisms to therapy. *Nat. Med.*, 21(12):1424–1435, Dec 2015.

[27] J. P. Coppe, P. Y. Desprez, A. Krtolica, and J. Campisi. The senescence-associated secretory phenotype: the dark side of tumor suppression. *Annu Rev Pathol*, 5:99–118, 2010.

[28] B. De Craene, B. Gilbert, C. Stove, E. Bruyneel, F. van Roy, and G. Berx. The transcription factor snail induces tumor cell invasion through modulation of the epithelial cell differentiation program. *Cancer Res.*, 65(14):6237–6244, Jul 2005.

[29] B. De Craene, F. van Roy, and G. Berx. Unraveling signalling cascades for the Snail family of transcription factors. *Cell. Signal.*, 17(5):535–547, May 2005.

[30] P. Dekker, D. Gunn, T. McBryan, R. W. Dirks, D. van Heemst, F. L. Lim, A. G. Jochemsen, M. Verlaan-de Vries, J. Nagel, P. D. Adams, H. J. Tanke, R. G. Westendorp, and A. B. Maier. Microarray-based identification of age-dependent differences in gene expression of human dermal fibroblasts. *Mech. Ageing Dev.*, 133(7):498–507, Jul 2012.

[31] L. Deng, R. R. Broaddus, A. McCampbell, G. L. Shipley, D. S. Loose, G. M. Stancel, J. H. Pickar, and P. J. Davies. Identification of a novel estrogen-regulated gene, EIG121, induced by hormone replacement therapy and differentially expressed in type I and type II endometrial cancer. *Clin. Cancer Res.*, 11(23):8258–8264, Dec 2005.

[32] T. Dimauro and G. David. Ras-induced senescence and its physiological relevance in cancer. *Curr Cancer Drug Targets*, 10(8):869–876, Dec 2010.

[33] G. P. Dimri, X. Lee, G. Basile, M. Acosta, G. Scott, C. Roskelley, E. E. Medrano, M. Linskens, I. Rubelj, and O. Pereira-Smith. A biomarker that identifies senescent human cells in culture and in aging skin in vivo. *Proc. Natl. Acad. Sci. U.S.A.*, 92(20):9363–9367, Sep 1995.

[34] M. J. Doherty, P. R. Young, and P. T. Cohen. Amino acid sequence of a novel protein phosphatase 1 binding protein (R5) which is related to the liver- and muscle-specific glycogen binding subunits of protein phosphatase 1. *FEBS Lett.*, 399(3):339–343, Dec 1996.

[35] M. Ejarque, J. Altirriba, R. Gomis, and R. Gasa. Characterization of the transcriptional activity of the basic helix-loop-helix (bHLH) transcription factor Atoh8. *Biochim. Biophys. Acta*, 1829(11):1175–1183, Nov 2013.

[36] M. Ejarque, J. Mir-Coll, R. Gomis, M. S. German, F. C. Lynn, and R. Gasa. Generation of a Conditional Allele of the Transcription Factor Atonal Homolog 8 (Atoh8). *PLoS ONE*, 11(1):e0146273, 2016.

[37] J. P. Ellard, C. R. McCudden, C. Tanega, K. A. James, S. Ratkovic, J. F. Staples, and G. F. Wagner. The respiratory effects of stanniocalcin-1 (STC-1) on intact mitochondria and cells: STC-1 uncouples oxidative phosphorylation and its actions are modulated by nucleotide triphosphates. *Mol. Cell. Endocrinol.*, 264(1-2):90–101, Jan 2007.

[38] V. P. Eswarakumar, I. Lax, and J. Schlessinger. Cellular signaling by fibroblast growth factor receptors. *Cytokine Growth Factor Rev.*, 16(2):139–149, Apr 2005.

[39] O. V. Evgrafov, I. Mersiyanova, J. Irobi, L. Van Den Bosch, I. Dierick, C. L. Leung, O. Schagina, N. Verpoorten, K. Van Impe, V. Fedotov, E. Dadali, M. Auer-Grumbach, C. Windpassinger, K. Wagner, Z. Mitrovic, D. Hilton-Jones, K. Talbot, J. J. Martin, N. Vasserman, S. Tverskaya, A. Polyakov, R. K. Liem, J. Gettemans, W. Robberecht, P. De Jonghe, and V. Timmerman. Mutant small heat-shock protein 27 causes axonal Charcot-Marie-Tooth disease and distal hereditary motor neuropathy. *Nat. Genet.*, 36(6):602–606, Jun 2004.

[40] A. C. Ferguson-Smith, Y. F. Chen, M. S. Newman, L. T. May, P. B. Sehgal, and F. H. Ruddle. Regional localization of the interferon-beta 2/B-cell stimulatory factor 2/hepatocyte stimulating factor gene to human chromosome 7p15-p21. *Genomics*, 2(3):203–208, Apr 1988.

[41] P. J. Fernandez-Marcos, C. Pantoja, A. Gonzalez-Rodriguez, N. Martin, J. M. Flores, A. M. Valverde, E. Hara, and M. Serrano. Normal proliferation and tumorigenesis but impaired pancreatic function in mice lacking the cell cycle regulator sei1. *PLoS ONE*, 5(1):e8744, 2010.

[42] G. J. Fisher, T. Quan, T. Purohit, Y. Shao, M. K. Cho, T. He, J. Varani, S. Kang, and J. J. Voorhees. Collagen fragmentation promotes oxidative stress and elevates matrix metalloproteinase-1 in fibroblasts in aged human skin. *Am. J. Pathol.*, 174(1):101–114, Jan 2009.

[43] L. A. Flynn, A. R. Blissett, E. P. Calomeni, and G. Agarwal. Inhibition of collagen fibrillogenesis by cells expressing soluble extracellular domains of DDR1 and DDR2. *J. Mol. Biol.*, 395(3):533–543, Jan 2010.

[44] A. Freund, A. V. Orjalo, P. Y. Desprez, and J. Campisi. Inflammatory networks during cellular senescence: causes and consequences. *Trends Mol Med*, 16(5):238–246, May 2010.

[45] M. Fujita, Y. Furukawa, T. Tsunoda, T. Tanaka, M. Ogawa, and Y. Nakamura. Up-regulation of the ectodermal-neural cortex 1 (ENC1) gene, a downstream target of the beta-catenin/T-cell factor complex, in colorectal carcinomas. *Cancer Res.*, 61(21):7722–7726, Nov 2001.

[46] S. G. Furness and K. McNagny. Beyond mere markers: functions for CD34 family of sialomucins in hematopoiesis. *Immunol. Res.*, 34(1):13–32, 2006.

[47] T. Gaarenstroom and C. S. Hill. TGF-Î² signaling to chromatin: how Smads regulate transcription during self-renewal and differentiation. *Semin. Cell Dev. Biol.*, 32:107–118, Aug 2014.

[48] C. A. Gebeshuber, C. Kornauth, L. Dong, R. Sierig, J. Seibler, M. Reiss, S. Tauber, M. Bilban, S. Wang, R. Kain, G. A. Bohmig, M. J. Moeller, H. J. Grone, C. Englert, J. Martinez, and D. Kerjaschki. Focal segmental glomerulosclerosis is induced by microRNA-193a and its downregulation of WT1. *Nat. Med.*, 19(4):481–487, Apr 2013.

[49] M. George, G. Ying, M. A. Rainey, A. Solomon, P. T. Parikh, Q. Gao, V. Band, and H. Band. Shared as well as distinct roles of EHD proteins revealed by biochemical and functional comparisons in mammalian cells and C. elegans. *BMC Cell Biol.*, 8:3, 2007.

[50] M. Gertz, F. Fischer, M. Leipelt, D. Wolters, and C. Steegborn. Identification of Peroxiredoxin 1 as a novel interaction partner for the lifespan regulator protein p66Shc. *Aging (Albany NY)*, 1(2):254–265, Feb 2009.

[51] C. Giacinti and A. Giordano. RB and cell cycle progression. *Oncogene*, 25(38):5220–5227, Aug 2006.

[52] M. Goldfarb. Fibroblast growth factor homologous factors: evolution, structure, and function. *Cytokine Growth Factor Rev.*, 16(2):215–220, Apr 2005.

[53] D. A. Goodenough and D. L. Paul. Gap junctions. *Cold Spring Harb Perspect Biol*, 1(1):a002576, Jul 2009.

[54] G. A. Gutman, K. G. Chandy, S. Grissmer, M. Lazdunski, D. McKinnon, L. A. Pardo, G. A. Robertson, B. Rudy, M. C. Sanguinetti, W. Stuhmer, and X. Wang. International Union of Pharmacology. LIII. Nomenclature and molecular relationships of voltage-gated potassium channels. *Pharmacol. Rev.*, 57(4):473–508, Dec 2005.

[55] P. A. Hall, K. Jung, K. J. Hillan, and S. E. Russell. Expression profiling the human septin gene family. *J. Pathol.*, 206(3):269–278, Jul 2005.

[56] K. H. Han, H. R. Choi, C. H. Won, J. H. Chung, K. H. Cho, H. C. Eun, and K. H. Kim. Alteration of the TGF-beta/SMAD pathway in intrinsically and UV-induced skin aging. *Mech. Ageing Dev.*, 126(5):560–567, May 2005.

[57] H. Hara, J. Araya, N. Takasaka, S. Fujii, J. Kojima, Y. Yumino, K. Shimizu, T. Ishikawa, T. Numata, M. Kawaishi, K. Saito, J. Hirano, M. Odaka, T. Morikawa, H. Hano, K. Nakayama, and K. Kuwano. Involvement of creatine kinase B in cigarette smoke-induced bronchial epithelial cell senescence. *Am. J. Respir. Cell Mol. Biol.*, 46(3):306–312, Mar 2012.

[58] C. B. Harley, A. B. Futcher, and C. W. Greider. Telomeres shorten during ageing of human fibroblasts. *Nature*, 345(6274):458–460, May 1990.

[59] H. Hayashi, S. Abdollah, Y. Qiu, J. Cai, Y. Y. Xu, B. W. Grinnell, M. A. Richardson, J. N. Topper, M. A. Gimbrone, J. L. Wrana, and D. Falb. The MAD-related protein Smad7 associates with the TGFbeta receptor and functions as an antagonist of TGFbeta signaling. *Cell*, 89(7):1165–1173, Jun 1997.

[60] L. HAYFLICK and P. S. MOORHEAD. The serial cultivation of human diploid cell strains. *Exp. Cell Res.*, 25:585–621, Dec 1961.

[61] P. He, Z. Peng, Y. Luo, L. Wang, P. Yu, W. Deng, Y. An, T. Shi, and D. Ma. High-throughput functional screening for autophagy-related genes and identification of TM9SF1 as an autophagosome-inducing gene. *Autophagy*, 5(1):52–60, Jan 2009.

[62] J. Heideker and I. E. Wertz. DUBs, the regulation of cell identity and disease. *Biochem. J.*, 465(1):1–26, Jan 2015.

[63] J. Heino. The collagen family members as cell adhesion proteins. *Bioessays*, 29(10):1001–1010, Oct 2007.

[64] K. Hemavathy, S. I. Ashraf, and Y. T. Ip. Snail/slug family of repressors: slowly going into the fast lane of development and cancer. *Gene*, 257(1):1–12, Oct 2000.

[65] J. A. Hennessey, E. Q. Wei, and G. S. Pitt. Fibroblast growth factor homologous factors modulate cardiac calcium channels. *Circ. Res.*, 113(4):381–388, Aug 2013.

[66] U. Herbig, M. Ferreira, L. Condel, D. Carey, and J. M. Sedivy. Cellular senescence in aging primates. *Science*, 311(5765):1257, Mar 2006.

[67] M. C. Hernandez, P. J. Andres-Barquin, I. Holt, and M. A. Israel. Cloning of human ENC-1 and evaluation of its expression and regulation in nervous system tumors. *Exp. Cell Res.*, 242(2):470–477, Aug 1998.

[68] M. C. Hernandez, P. J. Andres-Barquin, S. Martinez, A. Bulfone, J. L. Rubenstein, and M. A. Israel. ENC-1: a novel mammalian kelch-related gene specifically expressed in the nervous system encodes an actin-binding protein. *J. Neurosci.*, 17(9):3038–3051, May 1997.

[69] P. A. Hoodless, T. Haerry, S. Abdollah, M. Stapleton, M. B. O'Connor, L. Attisano, and J. L. Wrana. MADR1, a MAD-related protein that functions in BMP2 signaling pathways. *Cell*, 85(4):489–500, May 1996.

[70] S. Hopkins, E. Linderoth, O. Hantschel, P. Suarez-Henriques, G. Pilia, H. Kendrick, M. J. Smalley, G. Superti-Furga, and I. Ferby. Mig6 is a sensor of EGF receptor inactivation that directly activates c-Abl to induce apoptosis during epithelial homeostasis. *Dev. Cell*, 23(3):547–559, Sep 2012.

[71] T. Huang, W. Kang, A. S. Cheng, J. Yu, and K. F. To. The emerging role of Slit-Robo pathway in gastric and other gastro intestinal cancers. *BMC Cancer*, 15:950, 2015.

[72] N. Itoh and D. M. Ornitz. Functional evolutionary history of the mouse Fgf gene family. *Dev. Dyn.*, 237(1):18–27, Jan 2008.

[73] W. F. Jackson. Potassium channels and proliferation of vascular smooth muscle cells. *Circ. Res.*, 97(12):1211–1212, Dec 2005.

[74] J. C. Jeyapalan, M. Ferreira, J. M. Sedivy, and U. Herbig. Accumulation of senescent cells in mitotic tissue of aging primates. *Mech. Ageing Dev.*, 128(1):36–44, Jan 2007.

[75] Y. L. Juang, Y. M. Jeng, C. L. Chen, and H. C. Lien. PRRX2 as a novel TGF-beta-induced factor enhances invasion and migration in mammary epithelial cell and correlates with poor prognosis in breast cancer. *Mol. Carcinog.*, Jan 2016.

[76] J. I. Jun, K. H. Kim, and L. F. Lau. The matricellular protein CCN1 mediates neutrophil efferocytosis in cutaneous wound healing. *Nat Commun*, 6:7386, 2015.

[77] J. I. Jun and L. F. Lau. The matricellular protein CCN1 induces fibroblast senescence and restricts fibrosis in cutaneous wound healing. *Nat. Cell Biol.*, 12(7):676–685, Jul 2010.

[78] Wolfgang Kaisers. *spliceSites: A bioconductor package for exploration of alignment gap positions from RNA-seq data*, 2012. R package version 1.8.3.

[79] Wolfgang Kaisers. *rbamtools: Read and Write BAM (Binary Alignment) Files*, 2016. R package version 2.16.0.

[80] Wolfgang Kaisers. *refGenome: Gene and Splice Site Annotation Using Annotation Data from Ensembl and UCSC Genome Browsers*, 2016. R package version 1.6.14.

[81] Wolfgang Kaisers, Holger Schwender, and Heiner Schaal. Hierarchical clustering of dna k-mer counts in rna-seq fastq files reveals batch effects. *arXiv*, arXiv:1405.0114, 2014.

[82] F. Kalfalah, S. Seggewiss, R. Walter, J. Tigges, M. Moreno-Villanueva, A. Burkle, S. Ohse, H. Busch, M. Boerries, B. Hildebrandt, B. Royer-Pokora, and F. Boege. Structural chromosome abnormalities, increased DNA strand breaks and DNA strand break repair deficiency in dermal fibroblasts from old female human donors. *Aging (Albany NY)*, 7(2):110–122, Feb 2015.

[83] L. Kananen, S. Marttila, T. Nevalainen, J. Jylhava, N. Mononen, M. Kahonen, O. T. Raitakari, T. Lehtimaki, and M. Hurme. Aging-associated DNA methylation changes in middle-aged individuals: the Young Finns study. *BMC Genomics*, 17:103, 2016.

[84] A. Kaneda, T. Fujita, M. Anai, S. Yamamoto, G. Nagae, M. Morikawa, S. Tsuji, M. Oshima, K. Miyazono, and H. Aburatani. Activation of Bmp2-Smad1 signal and its regulation by coordinated alteration of H3K27 trimethylation in Ras-induced senescence. *PLoS Genet.*, 7(11):e1002359, Nov 2011.

[85] T. Kaprio, C. Fermer, J. Hagstrom, H. Mustonen, C. Bockelman, O. Nilsson, and C. Haglund. Podocalyxin is a marker of poor prognosis in colorectal cancer. *BMC Cancer*, 14:493, 2014.

[86] S. Karvinen, M. Silvennoinen, P. Vainio, L. Sistonen, L. G. Koch, S. L. Britton, and H. Kainulainen. Effects of intrinsic aerobic capacity, aging and voluntary running on skeletal muscle sirtuins and heat shock proteins. *Exp. Gerontol.*, 79:46–54, Jun 2016.

[87] T. Katagiri and T. Watabe. Bone Morphogenetic Proteins. *Cold Spring Harb Perspect Biol*, 8(6), 2016.

[88] L. Kautz, D. Meynard, A. Monnier, V. Darnaud, R. Bouvet, R. H. Wang, C. Deng, S. Vaulont, J. Mosser, H. Coppin, and M. P. Roth. Iron regulates phosphorylation of Smad1/5/8 and gene expression of Bmp6, Smad7, Id1, and Atoh8 in the mouse liver. *Blood*, 112(4):1503–1509, Aug 2008.

[89] D. Kerjaschki, D. J. Sharkey, and M. G. Farquhar. Identification and characterization of podocalyxin–the major sialoprotein of the renal glomerular epithelial cell. *J. Cell Biol.*, 98(4):1591–1596, Apr 1984.

[90] L. Kerosuo, E. Juvonen, R. Alitalo, M. Gylling, D. Kerjaschki, and A. Miettinen. Podocalyxin in human haematopoietic cells. *Br. J. Haematol.*, 124(6):809–818, Mar 2004.

[91] K. H. Kim, G. T. Park, Y. B. Lim, S. W. Rue, J. C. Jung, J. K. Sonn, Y. S. Bae, J. W. Park, and Y. S. Lee. Expression of connective tissue growth factor, a biomarker in senescence of human diploid fibroblasts, is up-regulated by a transforming growth factor-beta-mediated signaling pathway. *Biochem. Biophys. Res. Commun.*, 318(4):819–825, Jun 2004.

[92] D. Krunic, S. Moshir, K. M. Greulich-Bode, R. Figueroa, A. Cerezo, H. Stammer, H. J. Stark, S. G. Gray, K. V. Nielsen, W. Hartschuh, and P. Boukamp. Tissue context-activated telomerase in human epidermis correlates with little age-dependent telomere loss. *Biochim. Biophys. Acta*, 1792(4):297–308, Apr 2009.

[93] K. Kurpinski, H. Lam, J. Chu, A. Wang, A. Kim, E. Tsay, S. Agrawal, D. V. Schaffer, and S. Li. Transforming growth factor-beta and notch signaling mediate stem cell differentiation into smooth muscle cells. *Stem Cells*, 28(4):734–742, Apr 2010.

[94] M. Kwon, E. Hanna, D. Lorang, M. He, J. S. Quick, A. Adem, C. Stevenson, J. Y. Chung, S. M. Hewitt, E. Zudaire, D. Esposito, F. Cuttitta, and S. K. Libutti. Functional characterization of filamin a interacting protein 1-like, a novel candidate for antivascular cancer therapy. *Cancer Res.*, 68(18):7332–7341, Sep 2008.

[95] I. L. Lai, S. Y. Wang, Y. L. Yao, and W. M. Yang. Transcriptional and subcellular regulation of the TRIP-Br family. *Gene*, 388(1-2):102–109, Feb 2007.

[96] D. W. Laird. Life cycle of connexins in health and disease. *Biochem. J.*, 394(Pt 3):527–543, Mar 2006.

[97] A. Laitinen, C. Bockelman, J. Hagstrom, A. Kokkola, C. Fermer, O. Nilsson, and C. Haglund. Podocalyxin as a Prognostic Marker in Gastric Cancer. *PLoS ONE*, 10(12):e0145079, 2015.

[98] P. E. Lapinski, J. A. Oliver, L. A. Kamen, E. D. Hughes, T. L. Saunders, and P. D. King. Genetic analysis of SH2D4A, a novel adapter protein related to T cell-specific adapter and adapter protein in lymphocytes of unknown function, reveals a redundant function in T cells. *J. Immunol.*, 181(3):2019–2027, Aug 2008.

[99] N. Leblanc. Kv3.4, a key signalling molecule controlling the cell cycle and proliferation of human arterial smooth muscle cells. *Cardiovasc. Res.*, 86(3):351–352, Jun 2010.

[100] D. J. Lee, D. H. Kang, M. Choi, Y. J. Choi, J. Y. Lee, J. H. Park, Y. J. Park, K. W. Lee, and S. W. Kang. Peroxiredoxin-2 represses melanoma metastasis by increasing E-Cadherin/beta-Catenin complexes in adherens junctions. *Cancer Res.*, 73(15):4744–4757, Aug 2013.

[101] A. Leo, J. Wienands, G. Baier, V. Horejsi, and B. Schraven. Adapters in lymphocyte signaling. *J. Clin. Invest.*, 109(3):301–309, Feb 2002.

[102] B. Li, H. Li, Y. Bai, R. Kirschner-Schwabe, J. J. Yang, Y. Chen, G. Lu, G. Tzoneva, X. Ma, T. Wu, W. Li, H. Lu, L. Ding, H. Liang, X. Huang, M. Yang, L. Jin, H. Kang, S. Chen, A. Du, S. Shen, J. Ding, H. Chen, J. Chen, A. von Stackelberg, L. Gu, J. Zhang, A. Ferrando, J. Tang, S. Wang, and B. B. Zhou. Negative feedback-defective PRPS1 mutants drive thiopurine resistance in relapsed childhood ALL. *Nat. Med.*, 21(6):563–571, Jun 2015.

[103] M. Li, G. Lu, J. Hu, X. Shen, J. Ju, Y. Gao, L. Qu, Y. Xia, Y. Chen, and Y. Bai. EVA1A/TMEM166 Regulates Embryonic Neurogenesis by Autophagy. *Stem Cell Reports*, 6(3):396–410, Mar 2016.

[104] J. Lin, Z. Deng, C. Tanikawa, T. Shuin, T. Miki, K. Matsuda, and Y. Nakamura. Down-regulation of the tumor suppressor HSPB7, involved in the p53 pathway, in renal cell carcinoma by hypermethylation. *Int. J. Oncol.*, 44(5):1490–1498, May 2014.

[105] F. Ling, B. Kang, and X. H. Sun. Id proteins: small molecules, mighty regulators. *Curr. Top. Dev. Biol.*, 110:189–216, 2014.

[106] R. Liu and J. P. Jin. Calponin isoforms CNN1, CNN2 and CNN3: Regulators for actin cytoskeleton functions in smooth muscle and non-muscle cells. *Gene*, 585(1):143–153, Jul 2016.

[107] C. Lopez-Otin, M. A. Blasco, L. Partridge, M. Serrano, and G. Kroemer. The hallmarks of aging. *Cell*, 153(6):1194–1217, Jun 2013.

[108] T. Lopez-Rovira, E. Chalaux, J. Massague, J. L. Rosa, and F. Ventura. Direct binding of Smad1 and Smad4 to two distinct motifs mediates bone morphogenetic protein-specific transcriptional activation of Id1 gene. *J. Biol. Chem.*, 277(5):3176–3185, Feb 2002.

[109] K. H. Lu, W. Li, X. H. Liu, M. Sun, M. L. Zhang, W. Q. Wu, W. P. Xie, and Y. Y. Hou. Long non-coding RNA MEG3 inhibits NSCLC cells proliferation and induces apoptosis by affecting p53 expression. *BMC Cancer*, 13:461, 2013.

[110] H. Macias, A. Moran, Y. Samara, M. Moreno, J. E. Compton, G. Harburg, P. Strickland, and L. Hinck. SLIT/ROBO1 signaling suppresses mammary branching morphogenesis by limiting basal cell number. *Dev. Cell*, 20(6):827–840, Jun 2011.

[111] O. M. Martinez-Estrada, A. Culleres, F. X. Soriano, H. Peinado, V. Bolos, F. O. Martinez, M. Reina, A. Cano, M. Fabre, and S. Vilaro. The transcription factors Slug and Snail act as repressors of Claudin-1 expression in epithelial cells. *Biochem. J.*, 394(Pt 2):449–457, Mar 2006.

[112] M. Masiero, F. C. Simoes, H. D. Han, C. Snell, T. Peterkin, E. Bridges, L. S. Mangala, S. Y. Wu, S. Pradeep, D. Li, C. Han, H. Dalton, G. Lopez-Berestein, J. B. Tuynman, N. Mortensen, J. L. Li, R. Patient, A. K. Sood, A. H. Banham, A. L. Harris, and F. M. Buffa. A core human primary tumor angiogenesis signature identifies the endothelial orphan receptor ELTD1 as a key regulator of angiogenesis. *Cancer Cell*, 24(2):229–241, Aug 2013.

[113] J. Massague. How cells read TGF-beta signals. *Nat. Rev. Mol. Cell Biol.*, 1(3):169–178, Dec 2000.

[114] M. Mehrabian, R. S. Sparkes, T. Mohandas, A. M. Fogelman, and A. J. Lusis. Localization of monocyte chemotactic protein-1 gene (SCYA2) to human chromosome 17q11.2-q21.1. *Genomics*, 9(1):200–203, Jan 1991.

[115] S. T. Menendez, J. P. Rodrigo, E. Allonca, D. Garcia-Carracedo, G. Alvarez-Alija, S. Casado-Zapico, M. F. Fresno, C. Rodriguez, C. Suarez, and J. M. Garcia-Pedrero. Expression and clinical significance of the Kv3.4 potassium channel subunit in the development and progression of head and neck squamous cell carcinomas. *J. Pathol.*, 221(4):402–410, Aug 2010.

[116] G. Mese, G. Richard, and T. W. White. Gap junctions: basic structure and function. *J. Invest. Dermatol.*, 127(11):2516–2524, Nov 2007.

[117] M. Milewska and W. Kolch. Mig-6 participates in the regulation of cell senescence and retinoblastoma protein phosphorylation. *Cell. Signal.*, 26(9):1870–1877, Sep 2014.

[118] R. Mittal, K. Patel, J. Mittal, B. Chan, D. Yan, M. Grati, and X. Z. Liu. Association of PRPS1 Mutations with Disease Phenotypes. *Dis. Markers*, 2015:127013, 2015.

[119] K. Miyazono. Transforming growth factor-beta signaling in epithelial-mesenchymal transition and progression of cancer. *Proc. Jpn. Acad., Ser. B, Phys. Biol. Sci.*, 85(8):314–323, 2009.

[120] T. Mondal, S. Subhash, R. Vaid, S. Enroth, S. Uday, B. Reinius, S. Mitra, A. Mohammed, A. R. James, E. Hoberg, A. Moustakas, U. Gyllensten, S. J. Jones, C. M. Gustafsson, A. H. Sims, F. Westerlund, E. Gorab, and C. Kanduri. MEG3 long noncoding RNA regulates the TGF-beta pathway genes through formation of RNA-DNA triplex structures. *Nat Commun*, 6:7743, 2015.

[121] S. Mostowy and P. Cossart. Septins: the fourth component of the cytoskeleton. *Nat. Rev. Mol. Cell Biol.*, 13(3):183–194, Mar 2012.

[122] S. Mujtaba, L. Zeng, and M. M. Zhou. Structure and acetyl-lysine recognition of the bromodomain. *Oncogene*, 26(37):5521–5527, Aug 2007.

[123] C. G. Mullighan. Mutant PRPS1: a new therapeutic target in relapsed acute lymphoblastic leukemia. *Nat. Med.*, 21(6):553–554, Jun 2015.

[124] D. Munoz-Espin and M. Serrano. Cellular senescence: from physiology to pathology. *Nat. Rev. Mol. Cell Biol.*, 15(7):482–496, Jul 2014.

[125] N. Naslavsky and S. Caplan. EHD proteins: key conductors of endocytic transport. *Trends Cell Biol.*, 21(2):122–131, Feb 2011.

[126] M. J. Nicklin, A. Weith, and G. W. Duff. A physical map of the region encompassing the human interleukin-1 alpha, interleukin-1 beta, and interleukin-1 receptor antagonist genes. *Genomics*, 19(2):382–384, Jan 1994.

[127] J. S. Nielsen, M. L. Graves, S. Chelliah, A. W. Vogl, C. D. Roskelley, and K. M. McNagny. The CD34-related molecule podocalyxin is a potent inducer of microvillus formation. *PLoS ONE*, 2(2):e237, 2007.

[128] J. S. Nielsen and K. M. McNagny. Novel functions of the CD34 family. *J. Cell. Sci.*, 121(Pt 22):3683–3692, Nov 2008.

[129] M. Notaridou, L. Quaye, D. Dafou, C. Jones, H. Song, E. H?gdall, S. K. Kjaer, L. Christensen, C. H?gdall, J. Blaakaer, V. McGuire, A. H. Wu, D. J. Van Den Berg, M. C. Pike, A. Gentry-Maharaj, E. Wozniak, T. Sher, I. J. Jacobs, J. Tyrer, J. M. Schildkraut, P. G. Moorman, E. S. Iversen, A. Jakubowska, K. M?drek, J. Lubi?ski, R. B. Ness, K. B. Moysich, G. Lurie, L. R. Wilkens, M. E. Carney, S. Wang-Gohrke, J. A. Doherty, M. A. Rossing, M. W. Beckmann, F. C. Thiel, A. B. Ekici, X. Chen, J. Beesley, J. Gronwald, P. A. Fasching, J. Chang-Claude, M. T. Goodman, G. Chenevix-Trench, A. Berchuck, C. L. Pearce, A. S. Whittemore, U. Menon, P. D. Pharoah, S. A. Gayther, and S. J. Ramus. Common alleles in candidate susceptibility genes associated with risk and development of epithelial ovarian cancer. *Int. J. Cancer*, 128(9):2063–2074, May 2011.

[130] E. G. Novikova and L. D. Fricker. Purification and characterization of human metallocarboxypeptidase Z. *Biochem. Biophys. Res. Commun.*, 256(3):564–568, Mar 1999.

[131] E. G. Novikova, S. E. Reznik, O. Varlamov, and L. D. Fricker. Carboxypeptidase Z is present in the regulated secretory pathway and extracellular matrix in cultured cells and in human tissues. *J. Biol. Chem.*, 275(7):4865–4870, Feb 2000.

[132] N. Ohtani, Z. Zebedee, T. J. Huot, J. A. Stinson, M. Sugimoto, Y. Ohashi, A. D. Sharrocks, G. Peters, and E. Hara. Opposing effects of Ets and Id proteins on p16INK4a expression during cellular senescence. *Nature*, 409(6823):1067–1070, Feb 2001.

[133] T. Okada, K. Murata, R. Hirose, C. Matsuda, T. Komatsu, M. Ikekita, M. Nakawatari, F. Nakayama, M. Wakatsuki, T. Ohno, S. Kato, T. Imai, and T. Imamura. Upregulated expression of FGF13/FHF2 mediates resistance to platinum drugs in cervical cancer cells. *Sci Rep*, 3:2899, 2013.

[134] A. Olivera, T. Kohama, L. Edsall, V. Nava, O. Cuvillier, S. Poulton, and S. Spiegel. Sphingosine kinase expression increases intracellular sphingosine-1-phosphate and promotes cell growth and survival. *J. Cell Biol.*, 147(3):545–558, Nov 1999.

[135] A. M. Olovnikov. A theory of marginotomy. The incomplete copying of template margin in enzymic synthesis of polynucleotides and biological significance of the phenomenon. *J. Theor. Biol.*, 41(1):181–190, Sep 1973.

[136] A. M. Olovnikov. Telomeres, telomerase, and aging: origin of the theory. *Exp. Gerontol.*, 31(4):443–448, 1996.

[137] B. Ordog, E. Brutyo, L. G. Puskas, J. G. Papp, A. Varro, J. Szabad, and Z. Boldogkoi. Gene expression profiling of human cardiac potassium and sodium channels. *Int. J. Cardiol.*, 111(3):386–393, Aug 2006.

[138] D. Palme, M. Misovic, E. Schmid, D. Klumpp, H. R. Salih, J. Rudner, and S. M. Huber. Kv3.4 potassium channel-mediated electrosignaling controls cell cycle and survival of irradiated leukemia cells. *Pflugers Arch.*, 465(8):1209–1221, Aug 2013.

[139] S. W. Paugh, B. S. Paugh, M. Rahmani, D. Kapitonov, J. A. Almenara, T. Kordula, S. Milstien, J. K. Adams, R. E. Zipkin, S. Grant, and S. Spiegel. A selective sphingosine kinase 1 inhibitor integrates multiple molecular therapeutic targets in human leukemia. *Blood*, 112(4):1382–1391, Aug 2008.

[140] W. A. Paznekas, K. Okajima, M. Schertzer, S. Wood, and E. W. Jabs. Genomic organization, expression, and chromosome location of the human SNAIL gene (SNAI1) and a related processed pseudogene (SNAI1P). *Genomics*, 62(1):42–49, Nov 1999.

[141] W. Peng, S. Si, Q. Zhang, C. Li, F. Zhao, F. Wang, J. Yu, and R. Ma. Long non-coding RNA MEG3 functions as a competing endogenous RNA to regulate gastric cancer progression. *J. Exp. Clin. Cancer Res.*, 34:79, 2015.

[142] Y. Peng, S. Zhao, L. Song, M. Wang, and K. Jiao. Sertad1 encodes a novel transcriptional co-activator of SMAD1 in mouse embryonic hearts. *Biochem. Biophys. Res. Commun.*, 441(4):751–756, Nov 2013.

[143] G. Perez-Chacon, A. M. Astudillo, D. Balgoma, M. A. Balboa, and J. Balsinde. Control of free arachidonic acid levels by phospholipases A2 and lysophospholipid acyltransferases. *Biochim. Biophys. Acta*, 1791(12):1103–1113, Dec 2009.

[144] M. D. Perng, L. Cairns, P. van den IJssel, A. Prescott, A. M. Hutcheson, and R. A. Quinlan. Intermediate filament interactions can be altered by HSP27 and alphaB-crystallin. *J. Cell. Sci.*, 112 (Pt 13):2099–2112, Jul 1999.

[145] K. Polyak, Y. Xia, J. L. Zweier, K. W. Kinzler, and B. Vogelstein. A model for p53-induced apoptosis. *Nature*, 389(6648):300–305, Sep 1997.

[146] S. Prabhu, A. Ignatova, S. T. Park, and X. H. Sun. Regulation of the expression of cyclin-dependent kinase inhibitor p21 by E2A and Id proteins. *Mol. Cell. Biol.*, 17(10):5888–5896, Oct 1997.

[147] X. S. Puente, L. M. Sanchez, C. M. Overall, and C. Lopez-Otin. Human and mouse proteases: a comparative genomic approach. *Nat. Rev. Genet.*, 4(7):544–558, Jul 2003.

[148] R. Qin, Z. Chen, Y. Ding, J. Hao, J. Hu, and F. Guo. Long non-coding RNA MEG3 inhibits the proliferation of cervical carcinoma cells through the induction of cell cycle arrest and apoptosis. *Neoplasma*, 60(5):486–492, 2013.

[149] T. Quan, Y. Shao, T. He, J. J. Voorhees, and G. J. Fisher. Reduced expression of connective tissue growth factor (CTGF/CCN2) mediates collagen loss in chronologically aged human skin. *J. Invest. Dermatol.*, 130(2):415–424, Feb 2010.

[150] L. M. Randall, B. Manta, M. Hugo, M. Gil, C. Batthyany, M. Trujillo, L. B. Poole, and A. Denicola. Nitration transforms a sensitive peroxiredoxin 2 into a more active and robust peroxidase. *J. Biol. Chem.*, 289(22):15536–15543, May 2014.

[151] S. E. Reznik and L. D. Fricker. Carboxypeptidases from A to z: implications in embryonic development and Wnt binding. *Cell. Mol. Life Sci.*, 58(12-13):1790–1804, Nov 2001.

[152] S. G. Rhee, H. Z. Chae, and K. Kim. Peroxiredoxins: a historical overview and speculative preview of novel mechanisms and emerging concepts in cell signaling. *Free Radic. Biol. Med.*, 38(12):1543–1552, Jun 2005.

[153] G. J. Riggins, S. Thiagalingam, E. Rozenblum, C. L. Weinstein, S. E. Kern, S. R. Hamilton, J. K. Willson, S. D. Markowitz, K. W. Kinzler, and B. Vogelstein. Mad-related genes in the human. *Nat. Genet.*, 13(3):347–349, Jul 1996.

[154] S. Ross and C. S. Hill. How the Smads regulate transcription. *Int. J. Biochem. Cell Biol.*, 40(3):383–408, 2008.

[155] P. A. Ruiz and G. Jarai. Collagen I induces discoidin domain receptor (DDR) 1 expression through DDR2 and a JAK2-ERK1/2-mediated mechanism in primary human lung fibroblasts. *J. Biol. Chem.*, 286(15):12912–12923, Apr 2011.

[156] P. A. Ruiz and G. Jarai. Discoidin domain receptors regulate the migration of primary human lung fibroblasts through collagen matrices. *Fibrogenesis Tissue Repair*, 5:3, 2012.

[157] A. Salminen, K. Kaarniranta, and A. Kauppinen. Age-related changes in AMPK activation: Role for AMPK phosphatases and inhibitory phosphorylation by upstream signaling pathways. *Ageing Res. Rev.*, 28:15–26, Apr 2016.

[158] K. Saukkonen, J. Hagstrom, H. Mustonen, A. Juuti, S. Nordling, C. Fermer, O. Nilsson, H. Seppanen, and C. Haglund. Podocalyxin Is a Marker of Poor Prognosis in Pancreatic Ductal Adenocarcinoma. *PLoS ONE*, 10(6):e0129012, 2015.

[159] B. Schmierer and C. S. Hill. TGFbeta-SMAD signal transduction: molecular specificity and functional flexibility. *Nat. Rev. Mol. Cell Biol.*, 8(12):970–982, Dec 2007.

[160] J. Schoorlemmer and M. Goldfarb. Fibroblast growth factor homologous factors are intracellular signaling proteins. *Curr. Biol.*, 11(10):793–797, May 2001.

[161] S. Sizemore, M. Cicek, N. Sizemore, K. P. Ng, and G. Casey. Podocalyxin increases the aggressive phenotype of breast and prostate cancer cells in vitro through its interaction with ezrin. *Cancer Res.*, 67(13):6183–6191, Jul 2007.

[162] P. M. Smallwood, I. Munoz-Sanjuan, P. Tong, J. P. Macke, S. H. Hendry, D. J. Gilbert, N. G. Copeland, N. A. Jenkins, and J. Nathans. Fibroblast growth factor (FGF) homologous factors: new members of the FGF family implicated in nervous system development. *Proc. Natl. Acad. Sci. U.S.A.*, 93(18):9850–9857, Sep 1996.

[163] A. Solans, X. Estivill, and S. de La Luna. A new aspartyl protease on 21q22.3, BACE2, is highly similar to Alzheimer's amyloid precursor protein beta-secretase. *Cytogenet. Cell Genet.*, 89(3-4):177–184, 2000.

[164] L. Song and L. D. Fricker. Cloning and expression of human carboxypeptidase Z, a novel metallocarboxypeptidase. *J. Biol. Chem.*, 272(16):10543–10550, Apr 1997.

[165] M. E. Sowa, E. J. Bennett, S. P. Gygi, and J. W. Harper. Defining the human deubiquitinating enzyme interaction landscape. *Cell*, 138(2):389–403, Jul 2009.

[166] R. Stahl, T. Walcher, C. De Juan Romero, G. A. Pilz, S. Cappello, M. Irmler, J. M. Sanz-Aquela, J. Beckers, R. Blum, V. Borrell, and M. Gotz. Trnp1 regulates expansion and folding of the mammalian cerebral cortex by control of radial glial fate. *Cell*, 153(3):535–549, Apr 2013.

[167] J. H. Stockley and C. O'Neill. The proteins BACE1 and BACE2 and beta-secretase activity in normal and Alzheimer's disease brain. *Biochem. Soc. Trans.*, 35(Pt 3):574–576, Jun 2007.

[168] S. Stone, P. Jiang, P. Dayananth, S. V. Tavtigian, H. Katcher, D. Parry, G. Peters, and A. Kamb. Complex structure and regulation of the P16 (MTS1) locus. *Cancer Res.*, 55(14):2988–2994, Jul 1995.

[169] J. Su, B. Guo, T. Zhang, K. Wang, X. Li, and G. Liang. Stanniocalcin-1, a new biomarker of glioma progression, is associated with prognosis of patients. *Tumour Biol.*, 36(8):6333–6339, Aug 2015.

[170] M. Sugimoto, R. Yamashita, and M. Ueda. Telomere length of the skin in association with chronological aging and photoaging. *J. Dermatol. Sci.*, 43(1):43–47, Jul 2006.

[171] J. Tang, G. M. Gordon, B. J. Nickoloff, and K. E. Foreman. The helix-loop-helix protein id-1 delays onset of replicative senescence in human endothelial cells. *Lab. Invest.*, 82(8):1073–1079, Aug 2002.

[172] I. Tegeder and G. Geisslinger. Opioids as modulators of cell death and survival–unraveling mechanisms and revealing new indications. *Pharmacol. Rev.*, 56(3):351–369, Sep 2004.

[173] B. Trueb, L. Zhuang, S. Taeschler, and M. Wiedemann. Characterization of FGFRL1, a novel fibroblast growth factor (FGF) receptor preferentially expressed in skeletal tissues. *J. Biol. Chem.*, 278(36):33857–33865, Sep 2003.

[174] S. Tsuchiya, T. Fujiwara, F. Sato, Y. Shimada, E. Tanaka, Y. Sakai, K. Shimizu, and G. Tsujimoto. MicroRNA-210 regulates cancer cell proliferation through targeting fibroblast growth factor receptor-like 1 (FGFRL1). *J. Biol. Chem.*, 286(1):420–428, Jan 2011.

[175] E. Van Coillie, P. Fiten, H. Nomiyama, Y. Sakaki, R. Miura, O. Yoshie, J. Van Damme, and G. Opdenakker. The human MCP-2 gene (SCYA8): cloning, sequence analysis, tissue expression, and assignment to the CC chemokine gene contig on chromosome 17q11.2. *Genomics*, 40(2):323–331, Mar 1997.

[176] J. M. van Deursen. The role of senescent cells in ageing. *Nature*, 509(7501):439–446, May 2014.

[177] J. Varani, M. K. Dame, L. Rittie, S. E. Fligiel, S. Kang, G. J. Fisher, and J. J. Voorhees. Decreased collagen production in chronologically aged skin: roles of age-dependent alteration in fibroblast function and defective mechanical stimulation. *Am. J. Pathol.*, 168(6):1861–1868, Jun 2006.

[178] R. Vassar, B. D. Bennett, S. Babu-Khan, S. Kahn, E. A. Mendiaz, P. Denis, D. B. Teplow, S. Ross, P. Amarante, R. Loeloff, Y. Luo, S. Fisher, J. Fuller, S. Edenson, J. Lile, M. A. Jarosinski, A. L. Biere, E. Curran, T. Burgess, J. C. Louis, F. Collins, J. Treanor, G. Rogers, and M. Citron. Beta-secretase cleavage of Alzheimer's amyloid precursor protein by the transmembrane aspartic protease BACE. *Science*, 286(5440):735–741, Oct 1999.

[179] S. P. Vaughn, S. Broussard, C. R. Hall, A. Scott, S. H. Blanton, J. M. Milunsky, and J. T. Hecht. Confirmation of the mapping of the Camurati-Englemann locus to 19q13. 2 and refinement to a 3.2-cM region. *Genomics*, 66(1):119–121, May 2000.

[180] W. Vogel. Discoidin domain receptors: structural relations and functional implications. *FASEB J.*, 13 Suppl:77–82, 1999.

[181] W. Vogel, G. D. Gish, F. Alves, and T. Pawson. The discoidin domain receptor tyrosine kinases are activated by collagen. *Mol. Cell*, 1(1):13–23, Dec 1997.

[182] W. F. Vogel, R. Abdulhussein, and C. E. Ford. Sensing extracellular matrix: an update on discoidin domain receptor function. *Cell. Signal.*, 18(8):1108–1116, Aug 2006.

[183] J. Vogt, K. S. Dingwell, L. Herhaus, R. Gourlay, T. Macartney, D. Campbell, J. C. Smith, and G. P. Sapkota. Protein associated with SMAD1 (PAWS1/FAM83G) is a substrate for type I bone morphogenetic protein receptors and modulates bone morphogenetic protein signalling. *Open Biol*, 4:130210, 2014.

[184] L. Wang, C. Yu, Y. Lu, P. He, J. Guo, C. Zhang, Q. Song, D. Ma, T. Shi, and Y. Chen. TMEM166, a novel transmembrane protein, regulates cell autophagy and apoptosis. *Apoptosis*, 12(8):1489–1502, Aug 2007.

[185] T. Wang, Z. Q. Wang, L. Wang, L. Yan, J. Wan, S. Zhang, H. Q. Jiang, W. F. Li, and Z. F. Lin. CRISPLD2 is expressed at low levels during septic shock and is associated with procalcitonin. *PLoS ONE*, 8(6):e65743, 2013.

[186] Z. Q. Wang, W. M. Xing, H. H. Fan, K. S. Wang, H. K. Zhang, Q. W. Wang, J. Qi, H. M. Yang, J. Yang, Y. N. Ren, S. J. Cui, X. Zhang, F. Liu, D. H. Lin, W. H. Wang, M. K. Hoffmann, and Z. G. Han. The novel lipopolysaccharide-binding protein CRISPLD2 is a critical serum protein to regulate endotoxin function. *J. Immunol.*, 183(10):6646–6656, Nov 2009.

[187] P. A. Watkins, D. Maiguel, Z. Jia, and J. Pevsner. Evidence for 26 distinct acyl-coenzyme A synthetase genes in the human genome. *J. Lipid Res.*, 48(12):2736–2750, Dec 2007.

[188] R. R. White, B. Milholland, S. L. MacRae, M. Lin, D. Zheng, and J. Vijg. Comprehensive transcriptional landscape of aging mouse liver. *BMC Genomics*, 16:899, 2015.

[189] X. Y. Wu, Z. X. Fu, and X. H. Wang. Peroxiredoxins in colorectal neoplasms. *Histol. Histopathol.*, 25(10):1297–1303, Oct 2010.

[190] C. Xu, N. Aragam, X. Li, E. C. Villla, L. Wang, D. Briones, L. Petty, Y. Posada, T. B. Arana, G. Cruz, C. Mao, C. Camarillo, B. B. Su, M. A. Escamilla, and K. Wang. BCL9 and C9orf5 are associated with negative symptoms in schizophrenia: meta-analysis of two genome-wide association studies. *PLoS ONE*, 8(1):e51674, 2013.

[191] H. Xu, N. Raynal, S. Stathopoulos, J. Myllyharju, R. W. Farndale, and B. Leitinger. Collagen binding specificity of the discoidin domain receptors: binding sites on collagens II and III and molecular determinants for collagen IV recognition by DDR1. *Matrix Biol.*, 30(1):16–26, Jan 2011.

[192] M. Yagi, B. Zieger, G. J. Roth, and J. Ware. Structure and expression of the human septin gene HCDCREL-1. *Gene*, 212(2):229–236, Jun 1998.

[193] M. Yamanaka, D. Shegogue, H. Pei, S. Bu, A. Bielawska, J. Bielawski, B. Pettus, Y. A. Hannun, L. Obeid, and M. Trojanowska. Sphingosine kinase 1 (SPHK1) is induced by transforming growth factor-beta and mediates TIMP-1 up-regulation. *J. Biol. Chem.*, 279(52):53994–54001, Dec 2004.

[194] R. Yan, M. J. Bienkowski, M. E. Shuck, H. Miao, M. C. Tory, A. M. Pauley, J. R. Brashier, N. C. Stratman, W. R. Mathews, A. E. Buhl, D. B. Carter, A. G. Tomasselli, L. A. Parodi, R. L. Heinrikson, and M. E. Gurney. Membrane-anchored aspartyl protease with Alzheimer's disease beta-secretase activity. *Nature*, 402(6761):533–537, Dec 1999.

[195] Y. Yoshiko and J. E. Aubin. Stanniocalcin 1 as a pleiotropic factor in mammals. *Peptides*, 25(10):1663–1669, Oct 2004.

[196] Z. J. Zang, L. Gunaratnam, J. K. Cheong, L. Y. Lai, L. L. Hsiao, E. O'Leary, X. Sun, M. Salto-Tellez, J. V. Bonventre, and S. I. Hsu. Identification of PP2A as a novel interactor and regulator of TRIP-Br1. *Cell. Signal.*, 21(1):34–42, Jan 2009.

[197] Z. Zebedee and E. Hara. Id proteins in cell cycle control and cellular senescence. *Oncogene*, 20(58):8317–8325, Dec 2001.

[198] H. Zhang, Z. Liu, and S. Liu. HMGB1 induced inflammatory effect is blocked by CRISPLD2 via MiR155 in hepatic fibrogenesis. *Mol. Immunol.*, 69:1–6, Jan 2016.

[199] L. Zhao, F. Gregoire, and H. S. Sul. Transient induction of ENC-1, a Kelch-related actin-binding protein, is required for adipocyte differentiation. *J. Biol. Chem.*, 275(22):16845–16850, Jun 2000.

[200] W. Zheng, H. Wang, L. Xue, Z. Zhang, and T. Tong. Regulation of cellular senescence and p16(INK4a) expression by Id1 and E47 proteins in human diploid fibroblast. *J. Biol. Chem.*, 279(30):31524–31532, Jul 2004.

[201] Y. Zhou, X. Zhang, and A. Klibanski. MEG3 noncoding RNA: a tumor suppressor. *J. Mol. Endocrinol.*, 48(3):45–53, Jun 2012.

[202] D. V. Ziegler, C. D. Wiley, and M. C. Velarde. Mitochondrial effectors of cellular senescence: beyond the free radical theory of aging. *Aging Cell*, 14(1):1–7, Feb 2015.

[203] W. Zwerschke, S. Mazurek, P. Stockl, E. Hutter, E. Eigenbrodt, and P. Jansen-Durr. Metabolic analysis of senescent human fibroblasts reveals a role for AMP in cellular senescence. *Biochem. J.*, 376(Pt 2):403–411, Dec 2003.
