# Supplementary material for: Age, gender and UV-exposition related effects on gene expression in in vivo aged short term cultivated human dermal fibroblasts
Source: PLoS One. 2017 May 5;12(5):e0175657. doi: 10.1371/journal.pone.0175657 (PMC5419556; doi:10.1371/journal.pone.0175657)

Age related differential expression. Supplemental Material for: Age related Gene Expression Differences on Human Dermal Fibroblasts

Wolfgang Kaisers, CBiBs HHU Düsseldorf

June 9, 2016

Contents

1 Differential expression analysis **1**
1.1 Sample groups . . . 1

2 Complete gene table **1**

3 Gene list **2**
3.1 ATOH8 . . . 2
3.2 PODXL . . . 9
3.3 SNAI1 . . . 16
3.4 ID3 . . . 23
3.5 SPHK1 . . . 30
3.6 ID1 . . . 37
3.7 ERRFI1 . . . 44
3.8 PENK . . . 51
3.9 SEPT5 . . . 58
3.10 CPZ . . . 65
3.11 PRPS1 . . . 72
3.12 MEG3 . . . 79
3.13 CNN1 . . . 86
3.14 STC1 . . . 93
3.15 KIAA1324L . . . 100
3.16 TRNP1 . . . 107
3.17 HSPB7 . . . 114
3.18 PRRX2 . . . 121
3.19 SMAD7 . . . 128
3.20 FAM83G . . . 135
3.21 DDR1 . . . 142
3.22 PPP1R3C . . . 149

3.23 EVA1A . . . 156
3.24 CRISPLD2 . . . 163
3.25 RP11-309L24.6 . . . 170
3.26 ZNF385D . . . 177
3.27 FGFRL1 . . . 184
3.28 CKB . . . 191
3.29 FILIP1L . . . 198
3.30 GJA1 . . . 205
3.31 ENC1 . . . 212
3.32 SH2D4A . . . 219
3.33 ARHGAP23P1 . . . 226
3.34 SERTAD1 . . . 233
3.35 FGF13 . . . 240
3.36 EHD1 . . . 247
3.37 USP41 . . . 254
3.38 ACSS3 . . . 261
3.39 BACE2 . . . 268
3.40 ADGRL4 . . . 275
3.41 ROBO1 . . . 282
3.42 KCNC4 . . . 289

1 Differential expression analysis

1.1 Sample groups

Sample groups for differential expression analysis varied by donor age. The three disinguished groups are:

- Young (18-25 years)
- Middle (35-49 years)
- Old (60-67 years)

2 Complete gene table

gene_name	gene_id	maxald	old	seqid	strand	ID
ATOH8	ENSG00000168874	4308	down	2	+	1
PODXL	ENSG00000128567	3927	down	7	-	2
SNAI1	ENSG00000124216	4575	down	20	+	3
ID3	ENSG00000117318	19530	down	1	-	4
SPHK1	ENSG00000176170	6585	down	17	+	5
ID1	ENSG00000125968	14913	down	20	+	6

Continued on next page

gene_name	gene_id	maxald	old	seqid	strand	ID
ERRFI1	ENSG00000116285	1776	down	1	-	7
PENK	ENSG00000181195	2554	up	8	-	8
SEPT5	ENSG00000184702	1468	down	22	+	9
CPZ	ENSG00000109625	2585	up	4	+	10
PRPS1	ENSG00000147224	5550	down	X	+	11
MEG3	ENSG00000214548	3031	down	14	+	12
CNN1	ENSG00000130176	16224	down	19	+	13
STC1	ENSG00000159167	1877	up	8	-	14
KIAA1324L	ENSG00000164659	1751	up	7	-	15
TRNP1	ENSG00000253368	3613	down	1	+	16
HSPB7	ENSG00000173641	14954	down	1	-	17
PRRX2	ENSG00000167157	6616	down	9	+	18
SMAD7	ENSG00000101665	3768	down	18	-	19
FAM83G	ENSG00000188522	523	down	17	-	20
DDR1	ENSG00000204580	606	down	6	+	21
PPP1R3C	ENSG00000119938	1889	down	10	-	22
EVA1A	ENSG00000115363	862	down	2	-	23
CRISPLD2	ENSG00000103196	2516	down	16	+	24
RP11-309L24.6	ENSG00000224163	6288	down	7	-	25
ZNF385D	ENSG00000151789	529	up	3	-	26
FGFRL1	ENSG00000127418	1174	down	4	+	27
CKB	ENSG00000166165	2224	down	14	-	28
FILIP1L	ENSG00000168386	2028	down	3	-	29
GJA1	ENSG00000152661	12333	up	6	+	30
ENC1	ENSG00000171617	1200	down	5	-	31
SH2D4A	ENSG00000104611	5544	down	8	+	32
ARHGAP23P1	ENSG00000260781	763	down	16	-	33
SERTAD1	ENSG00000197019	2018	down	19	-	34
FGF13	ENSG00000129682	345	up	X	-	35
EHD1	ENSG00000110047	2871	down	11	-	36
USP41	ENSG00000161133	155	up	22	-	37
ACSS3	ENSG00000111058	201	down	12	+	38
BACE2	ENSG00000182240	2384	up	21	+	39
ADGRL4	ENSG00000162618	524	up	1	-	40
ROBO1	ENSG00000169855	826	up	3	-	41
KCNC4	ENSG00000116396	187	down	1	+	42

Table 1: Age differential expressed genes

3 Gene list

3.1 ATOH8

Parameter	Value
gene_name	ATOH8
gene_id	ENSG00000168874
maxAld	4308
old	down
seqid	2
strand	+
start	85751344
end	85788066
descr	atonal bHLH transcription factor 8

Table 2: Gene identifcation

Gene expression estimates for Age

Influence of UV exposition on gene expression

Figure 1: Gene expression estimates based on CPM (SummarizeOveraps)

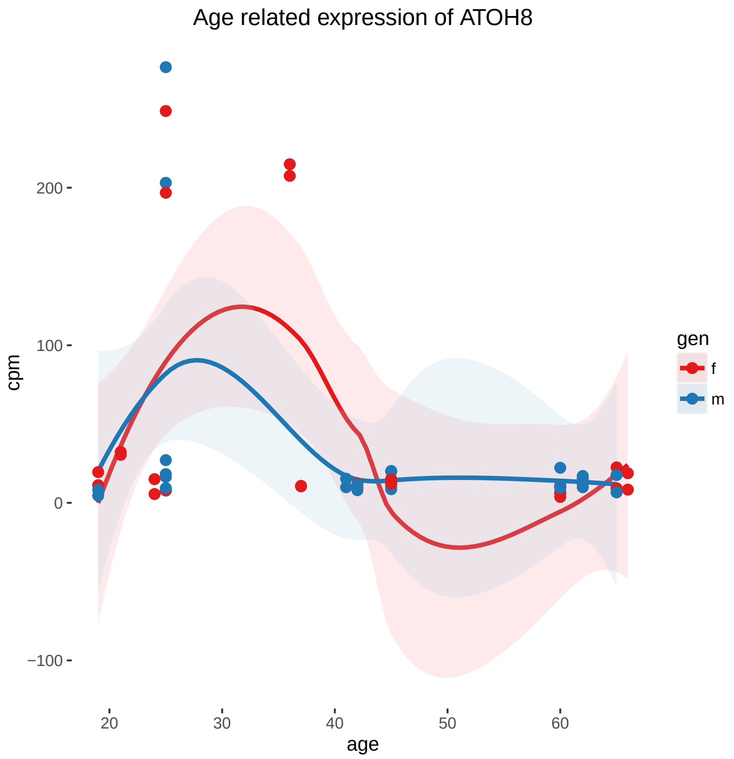

Figure 2: edgeR QLF test based CPM estimates

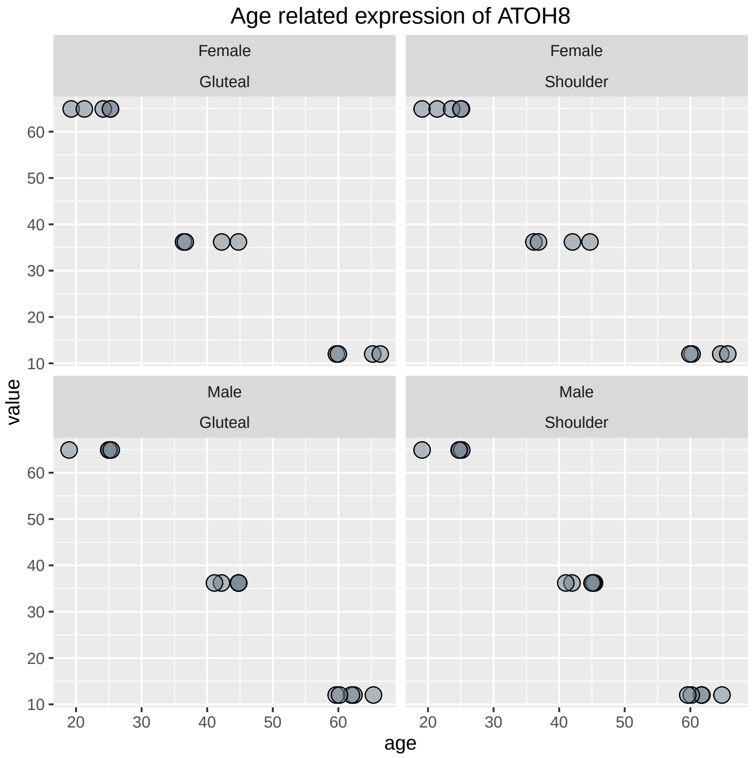

Figure 3: ReadExpSet based genewise CPM estimates

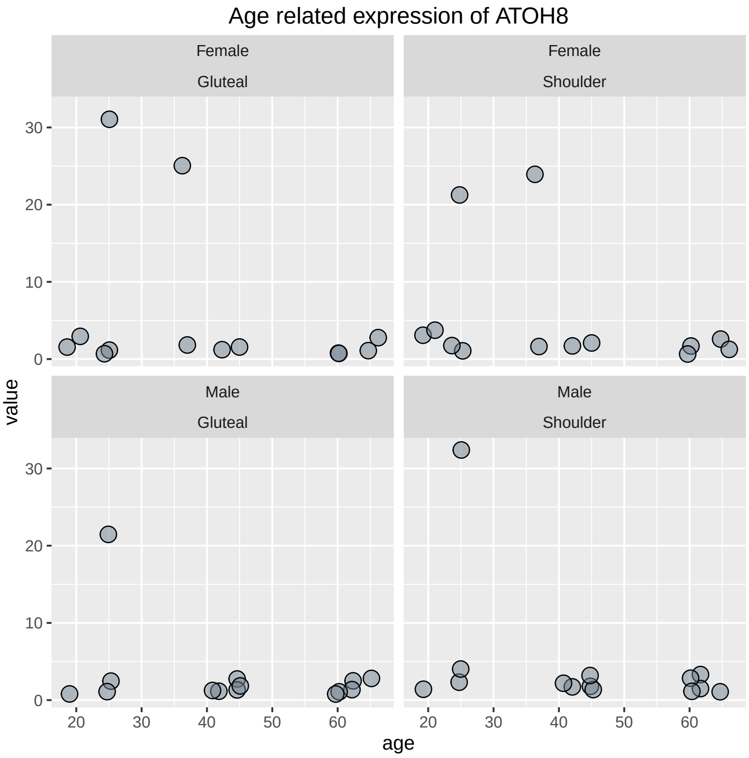

Figure 4: Loess regression for exon align depth

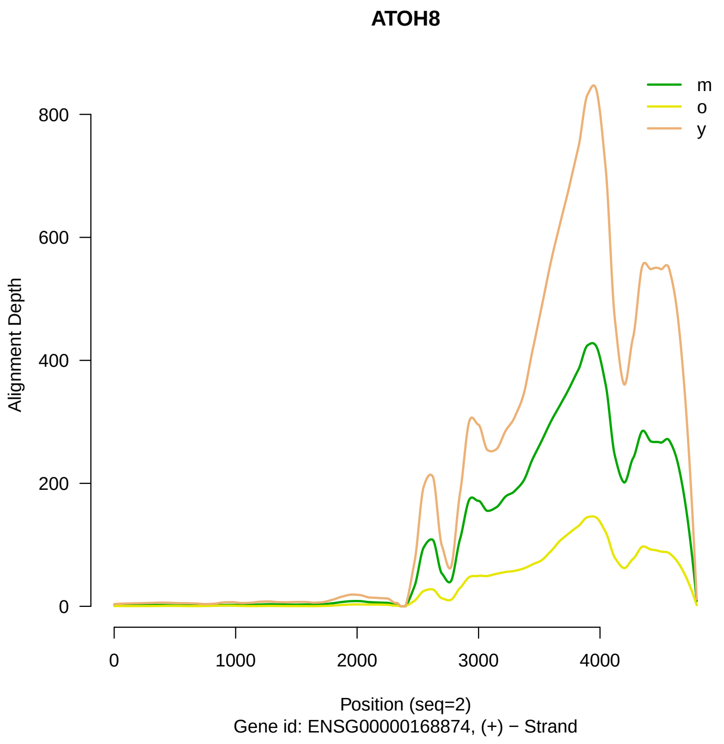

Figure 5: edgeR QLF test based CPM estimates

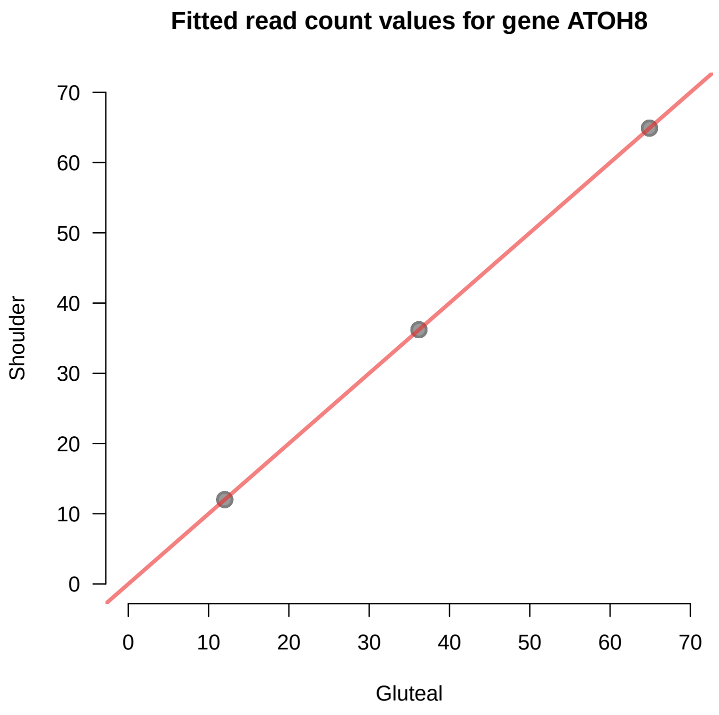

Figure 6: ReadExpSet based genewise CPM estimates

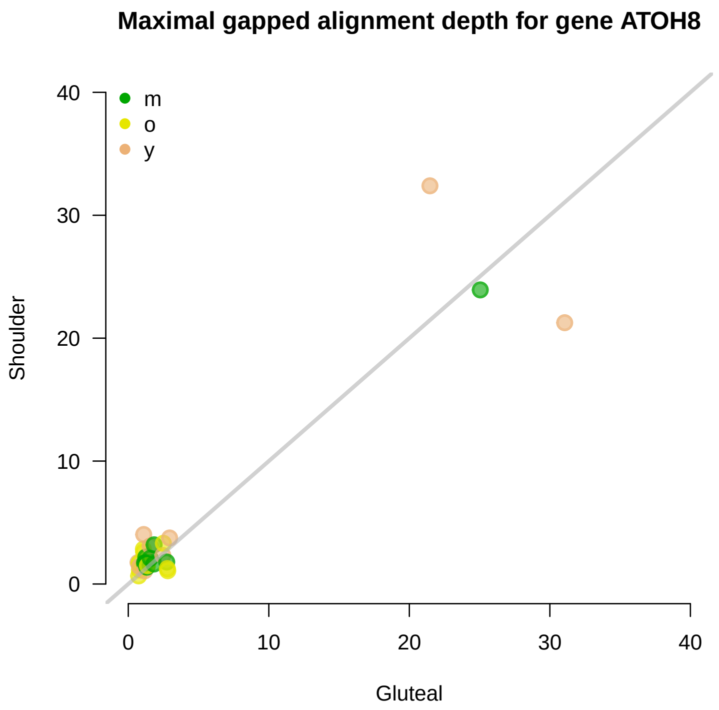

3.2 PODXL

Parameter	Value
gene_name	PODXL
gene_id	ENSG00000128567
maxAd	3927
old	down
seqid	7
strand	-
start	131500262
end	131558217
descr	podocalyxin-like

Table 3: Gene identifcation

Gene expression estimates for Age

Influence of UV exposition on gene expression

Figure 7: Gene expression estimates based on CPM (SummarizeOveraps)

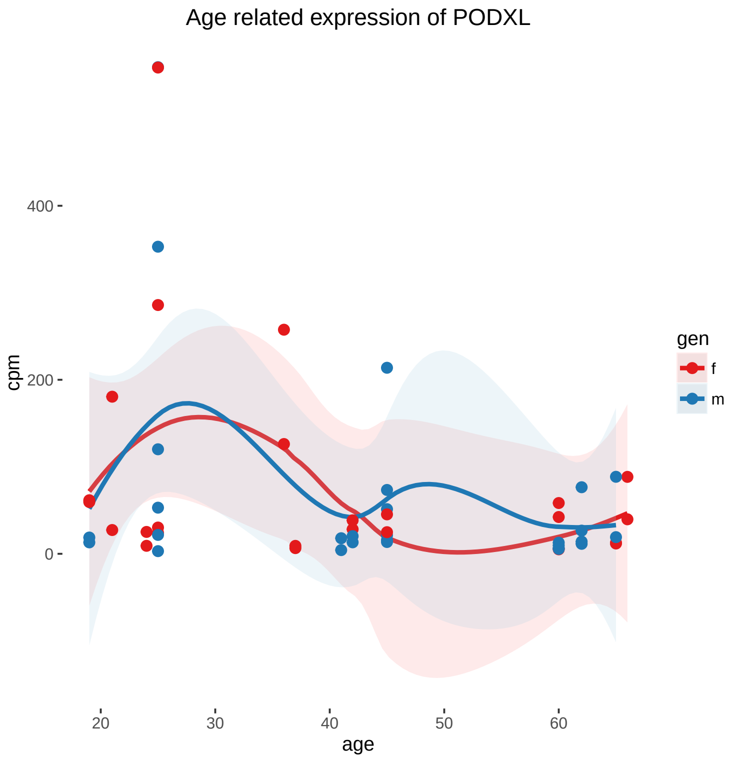

Figure 8: edgeR QLF test based CPM estimates

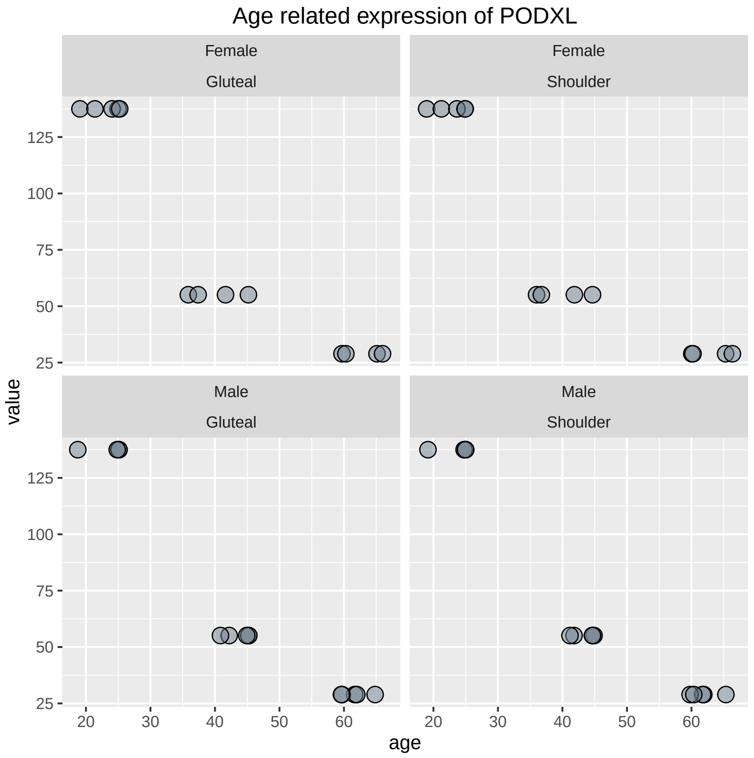

Figure 9: ReadExpSet based genewise CPM estimates

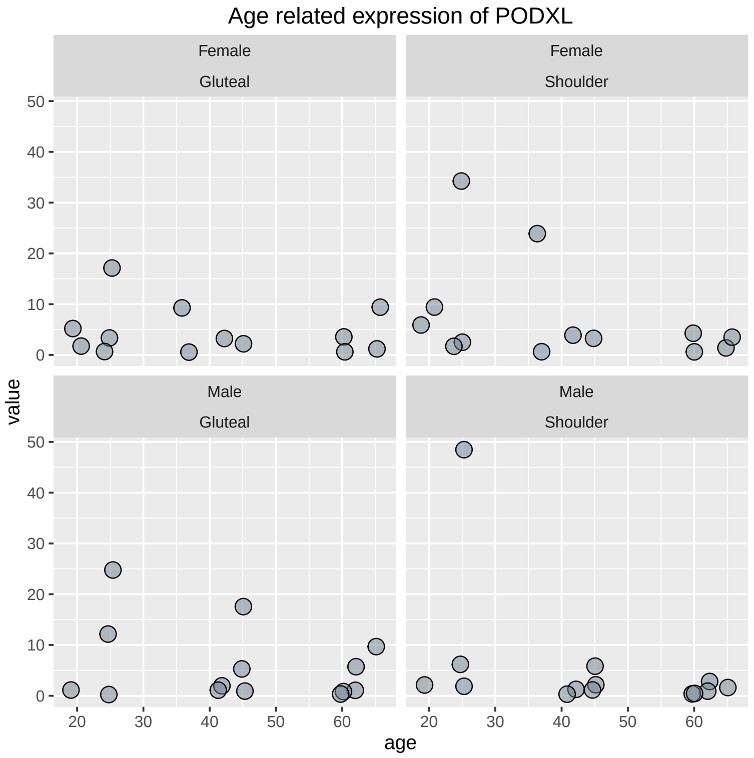

Figure 10: Loess regression for exon align depth

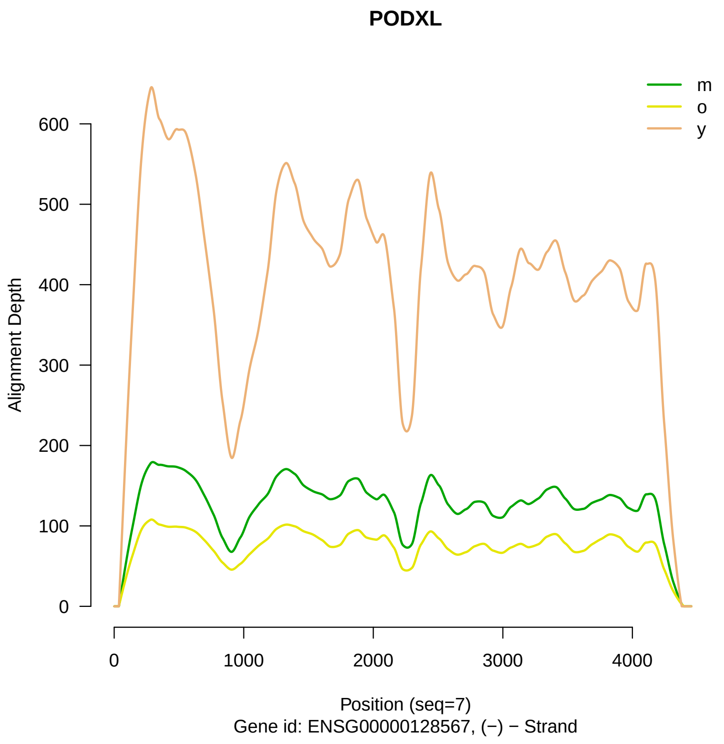

Figure 11: edgeR QLF test based CPM estimates

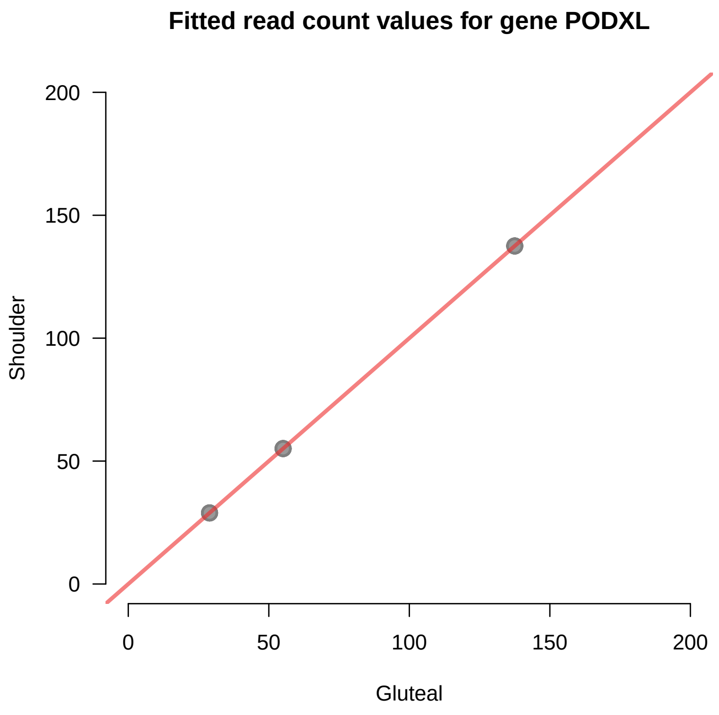

Figure 12: ReadExpSet based genewise CPM estimates

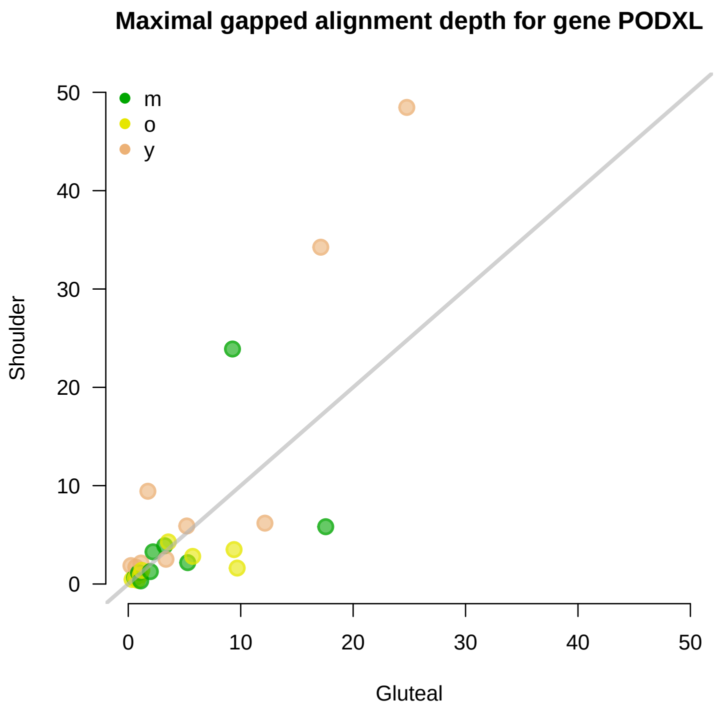

3.3 SNAI1

Parameter	Value
gene_name	SNAI1
gene_id	ENSG00000124216
maxald	4575
old	down
seqid	20
strand	+
start	49982999
end	49988886
descr	snail family zinc finger 1

Table 4: Gene identifcation

Gene expression estimates for Age

Influence of UV exposition on gene expression

Figure 13: Gene expression estimates based on CPM (SummarizeOveraps)

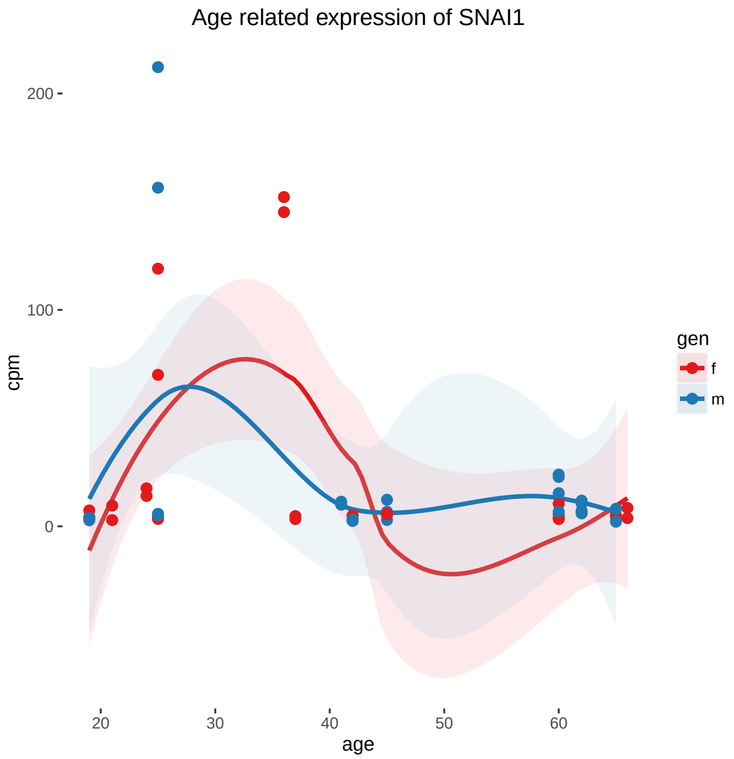

Figure 14: edgeR QLF test based CPM estimates

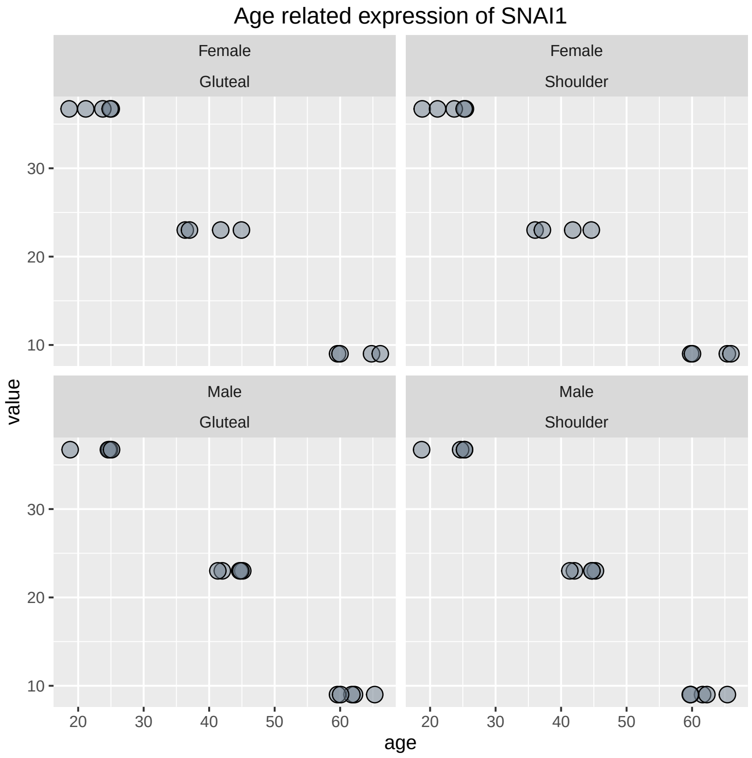

Figure 15: ReadExpSet based genewise CPM estimates

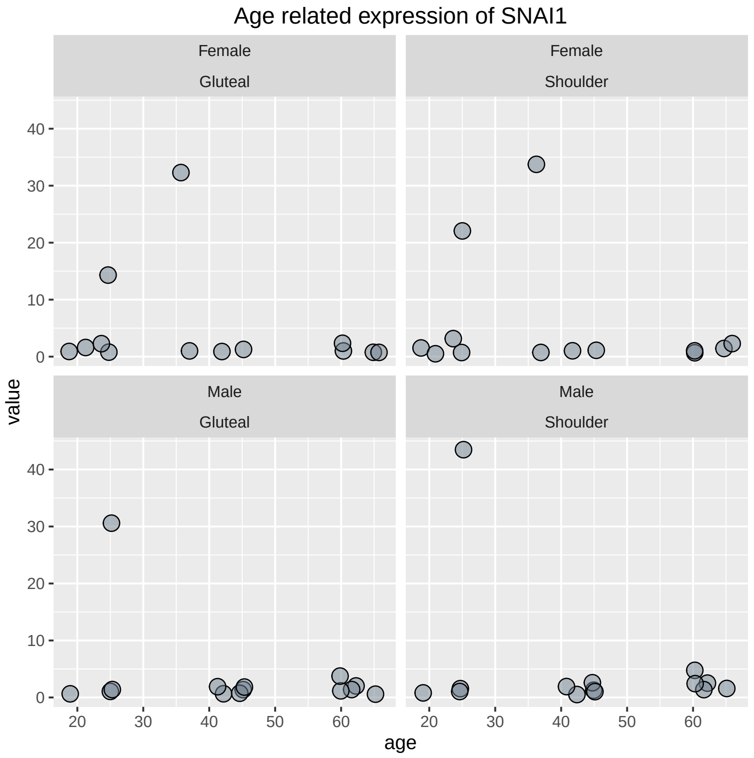

Figure 16: Loess regression for exon align depth

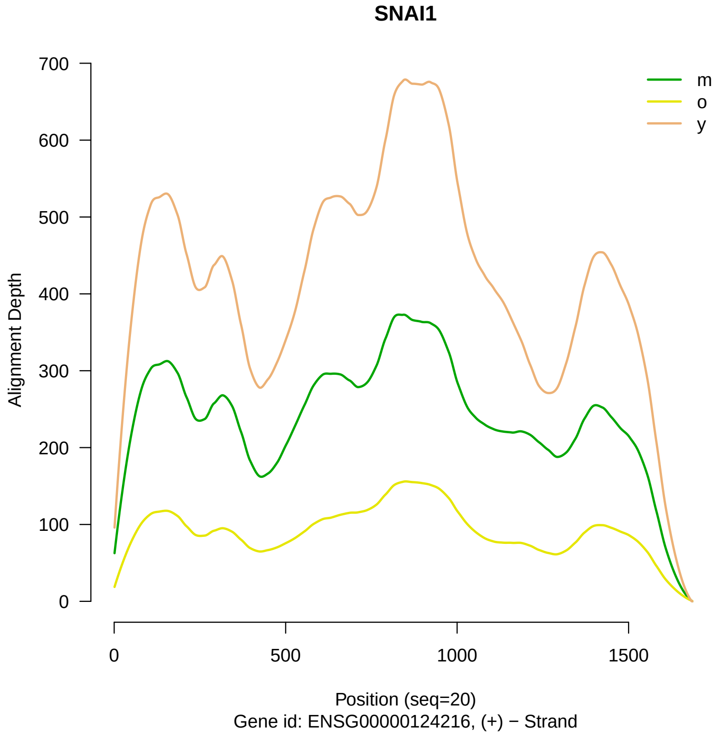

Figure 17: edgeR QLF test based CPM estimates

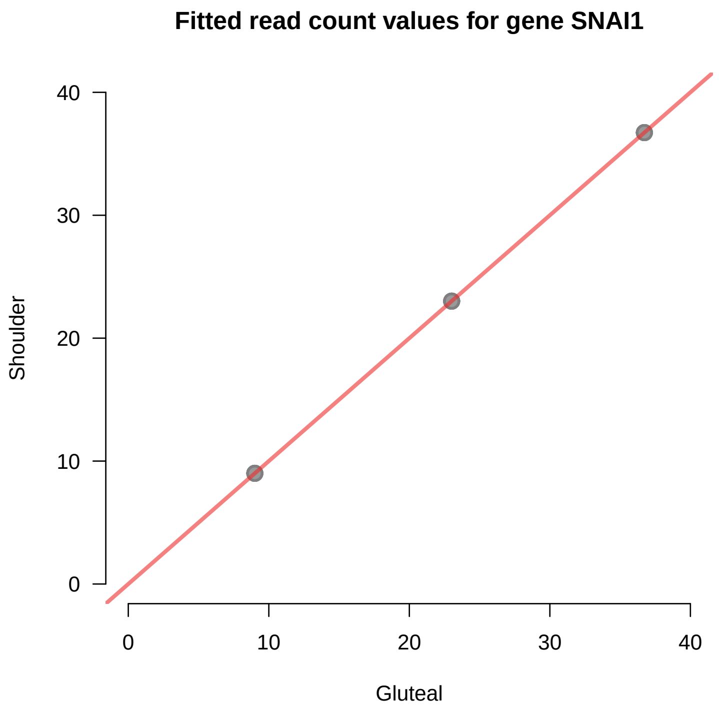

Figure 18: ReadExpSet based genewise CPM estimates

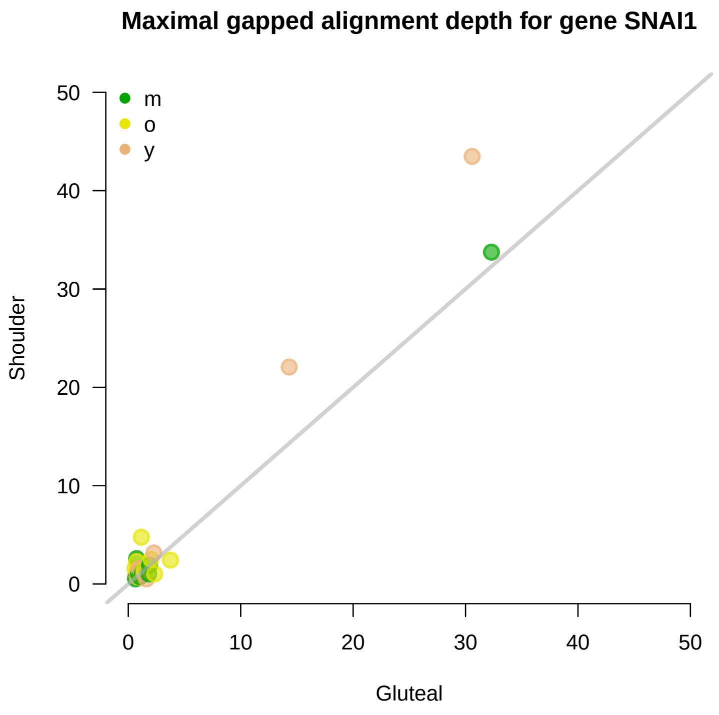

3.4 ID3

Parameter	Value
gene_name	ID3
gene_id	ENSG00000117318
maxAId	19530
oId	down
seqid	1
strand	-
start	23557918
end	23559794
descr	inhibitor of DNA binding 3, dominant negative helix-loop-helix protein

Table 5: Gene identifcation

Gene expression estimates for Age

Influence of UV exposition on gene expression

Figure 19: Gene expression estimates based on CPM (SummarizeOveraps)

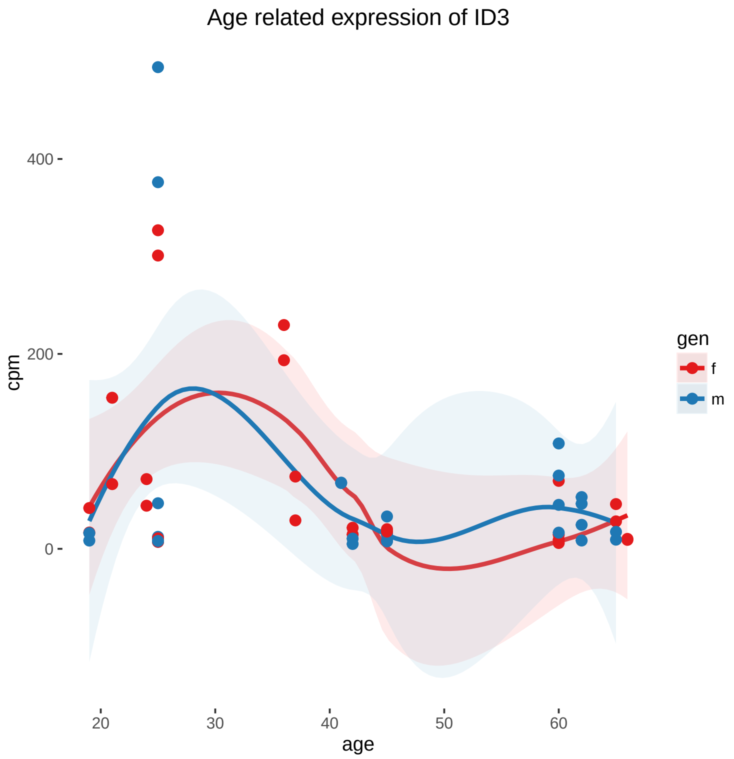

Figure 20: edgeR QLF test based CPM estimates

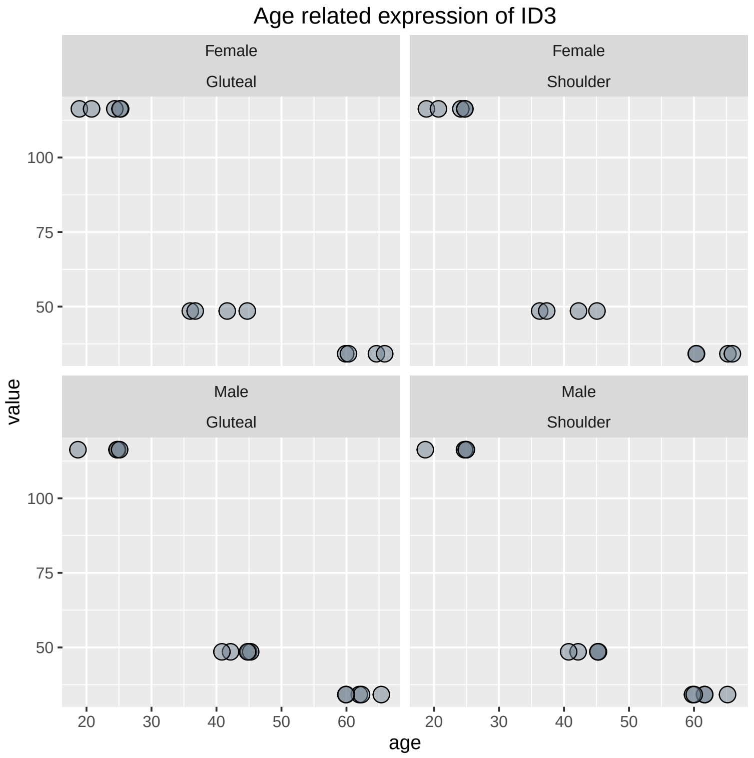

Figure 21: ReadExpSet based genewise CPM estimates

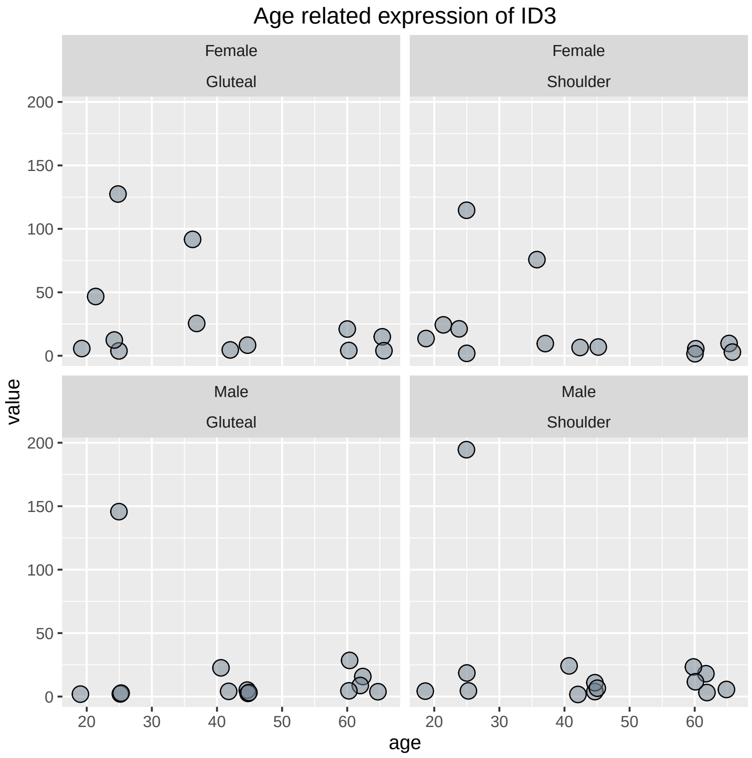

Figure 22: Loess regression for exon align depth

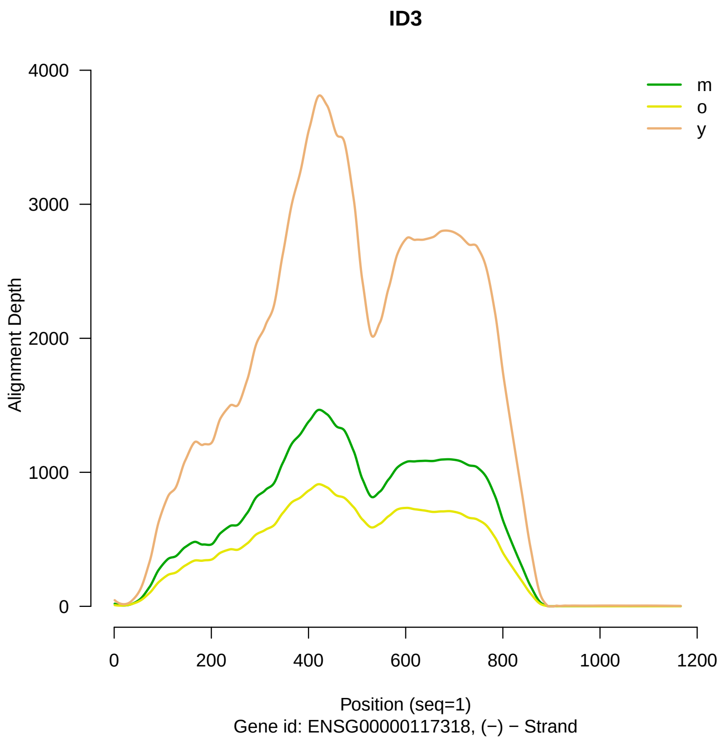

Figure 23: edgeR QLF test based CPM estimates

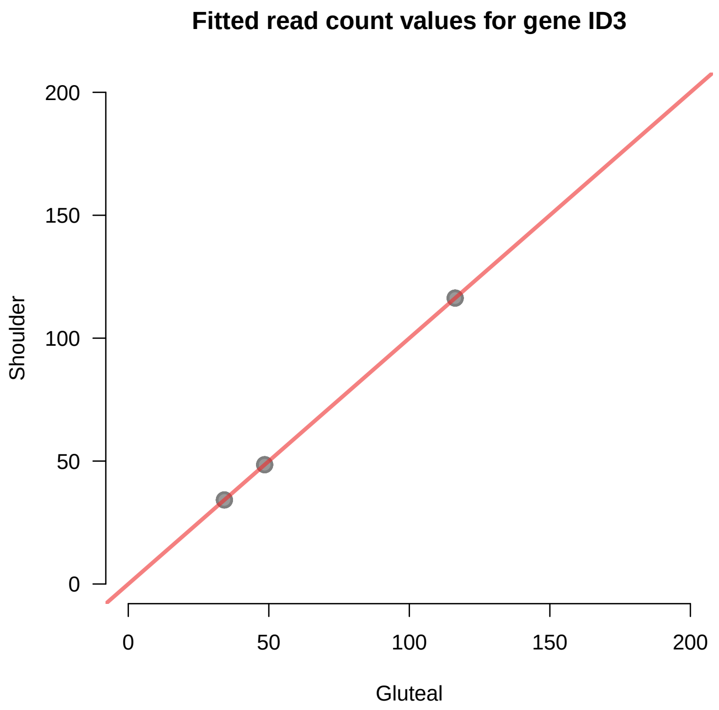

Figure 24: ReadExpSet based genewise CPM estimates

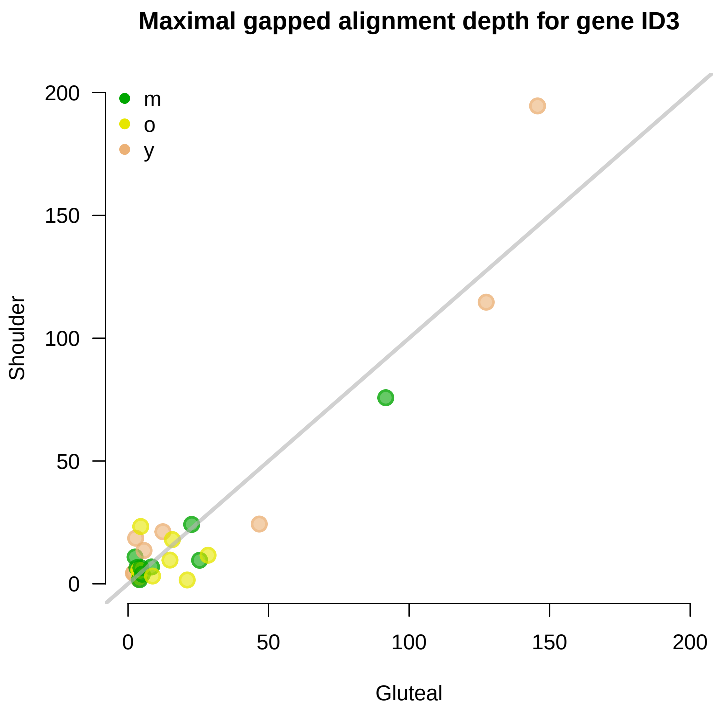

3.5 SPHK1

Parameter	Value
gene_name	SPHK1
gene_id	ENSG00000176170
maxald	6585
old	down
seqid	17
strand	+
start	76376584
end	76387860
descr	sphingosine kinase 1

Table 6: Gene identifcation

Gene expression estimates for Age

Influence of UV exposition on gene expression

Figure 25: Gene expression estimates based on CPM (SummarizeOveraps)

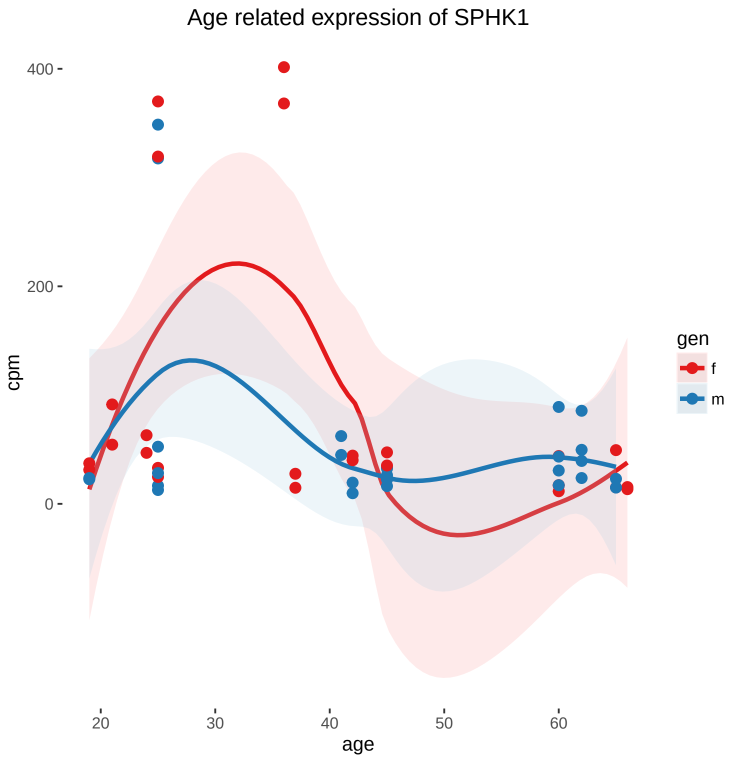

Figure 26: edgeR QLF test based CPM estimates

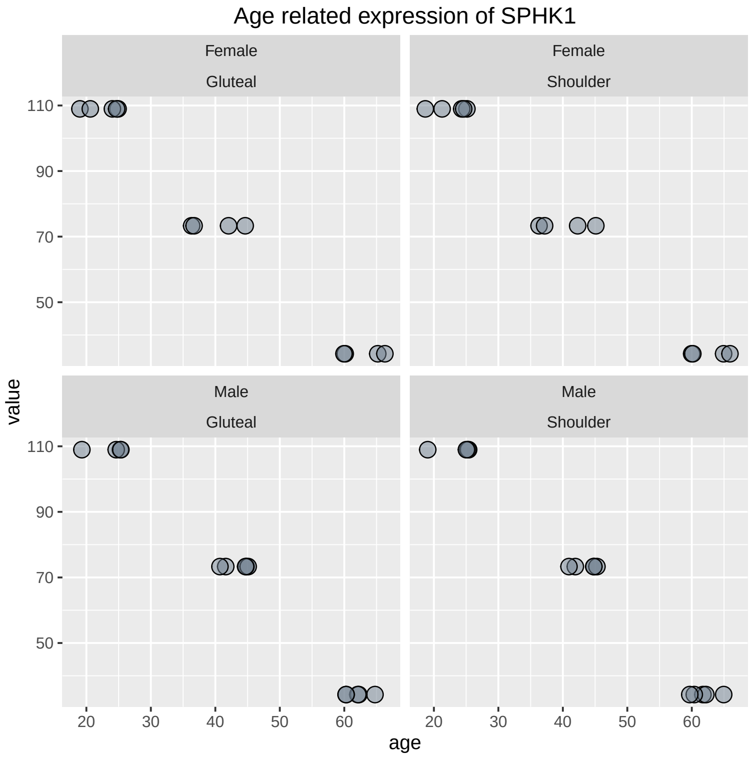

Figure 27: ReadExpSet based genewise CPM estimates

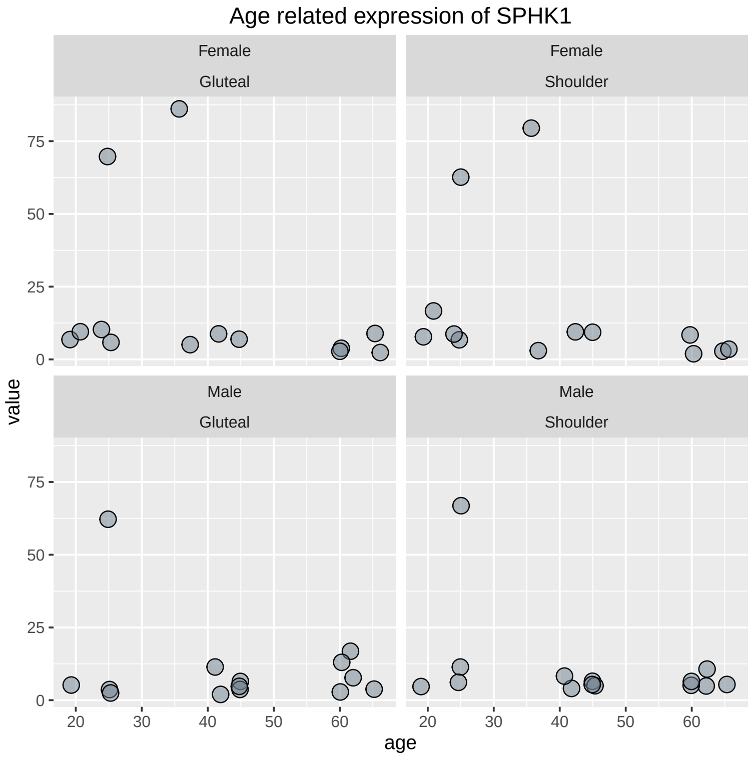

Figure 28: Loess regression for exon align depth

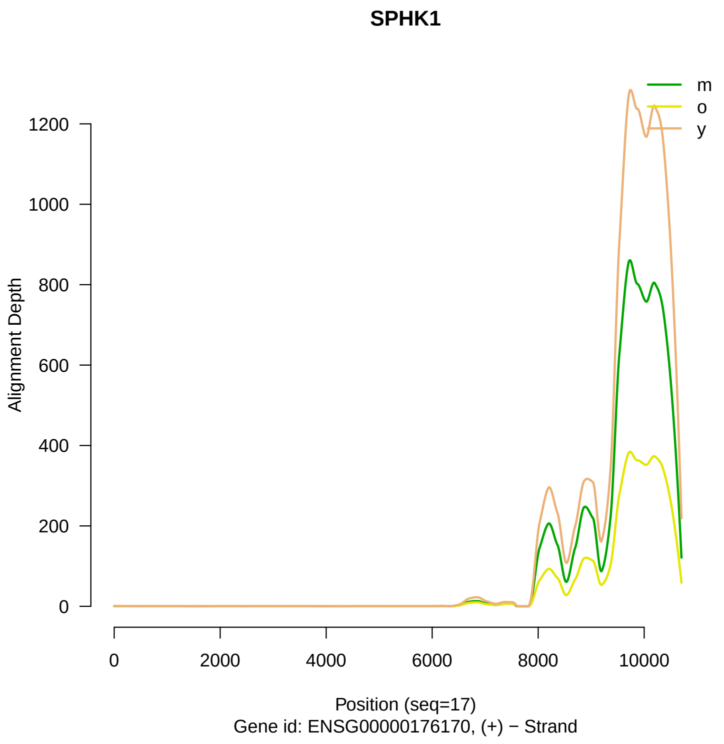

Figure 29: edgeR QLF test based CPM estimates

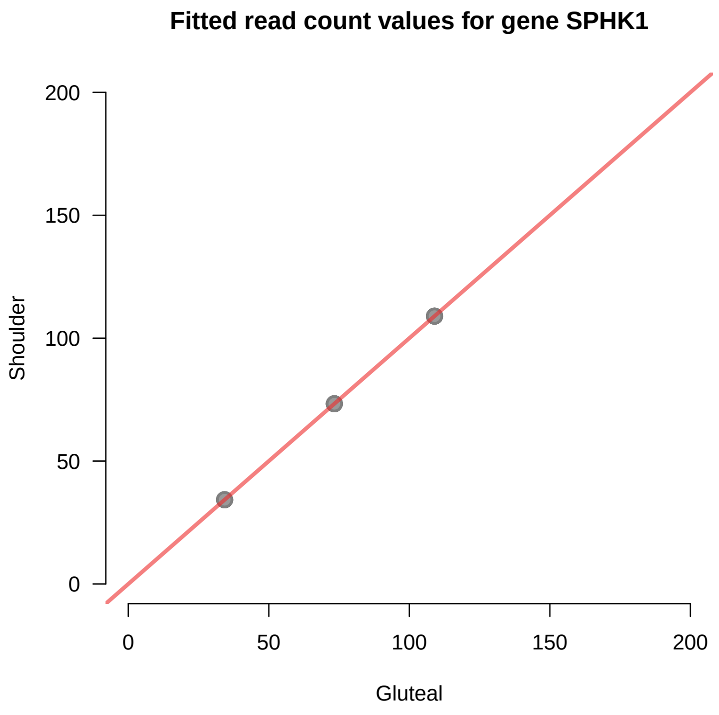

Figure 30: ReadExpSet based genewise CPM estimates

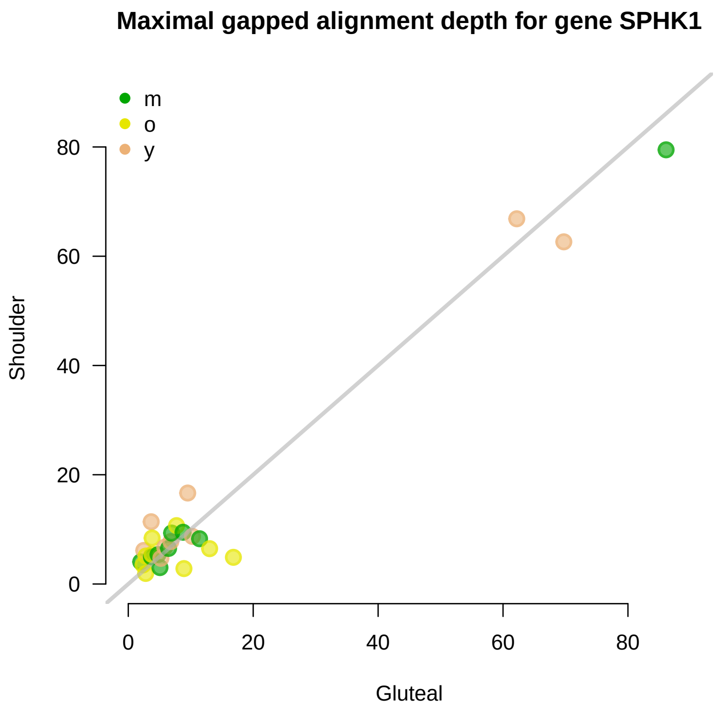

3.6 ID1

Parameter	Value
gene_name	ID1
gene_id	ENSG00000125968
maxald	14913
old	down
seqid	20
strand	+
start	31605283
end	31606515
descr	inhibitor of DNA binding 1, dominant negative helix-loop-helix protein

Table 7: Gene identifcation

Gene expression estimates for Age

Influence of UV exposition on gene expression

Figure 31: Gene expression estimates based on CPM (SummarizeOveraps)

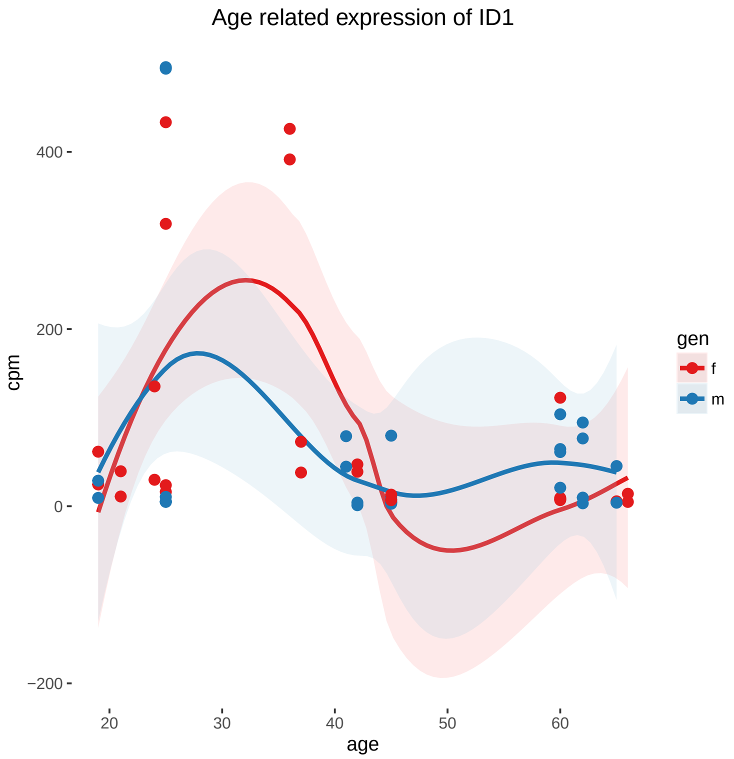

Figure 32: edgeR QLF test based CPM estimates

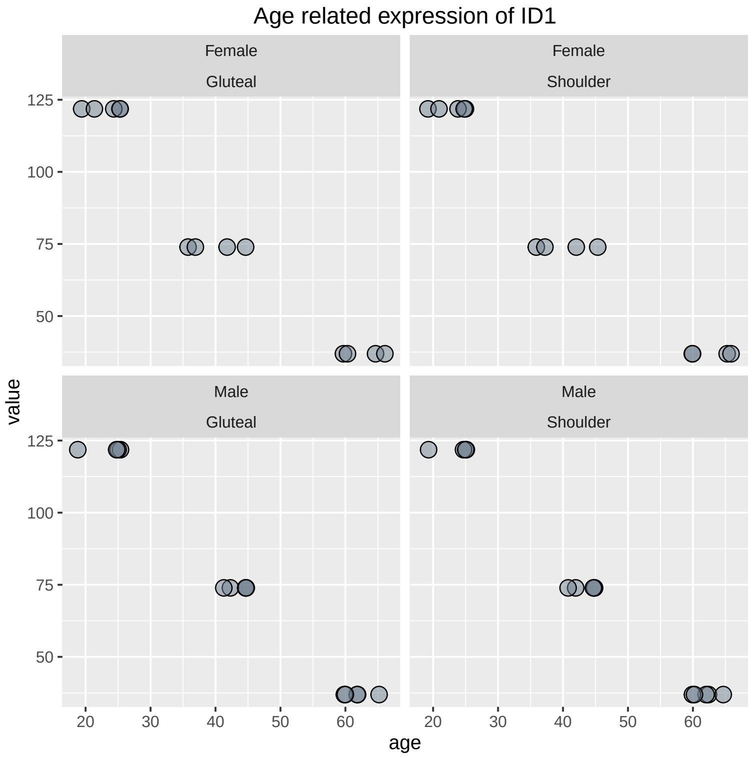

Figure 33: ReadExpSet based genewise CPM estimates

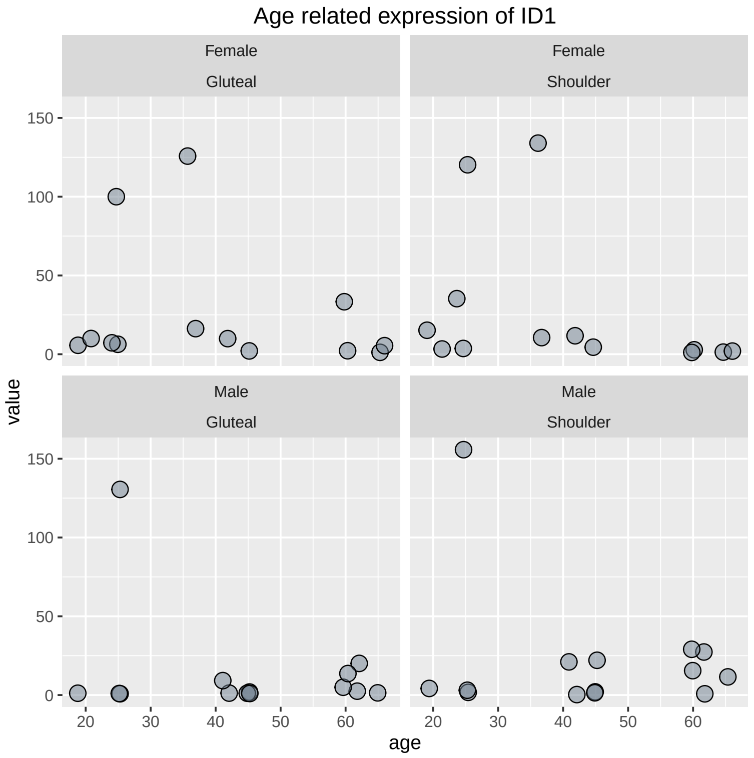

Figure 34: Loess regression for exon align depth

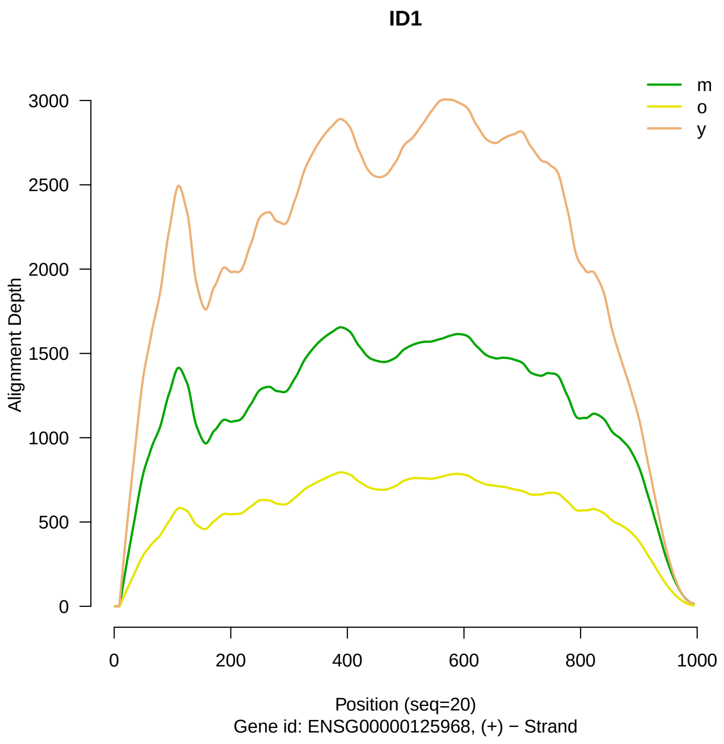

Figure 35: edgeR QLF test based CPM estimates

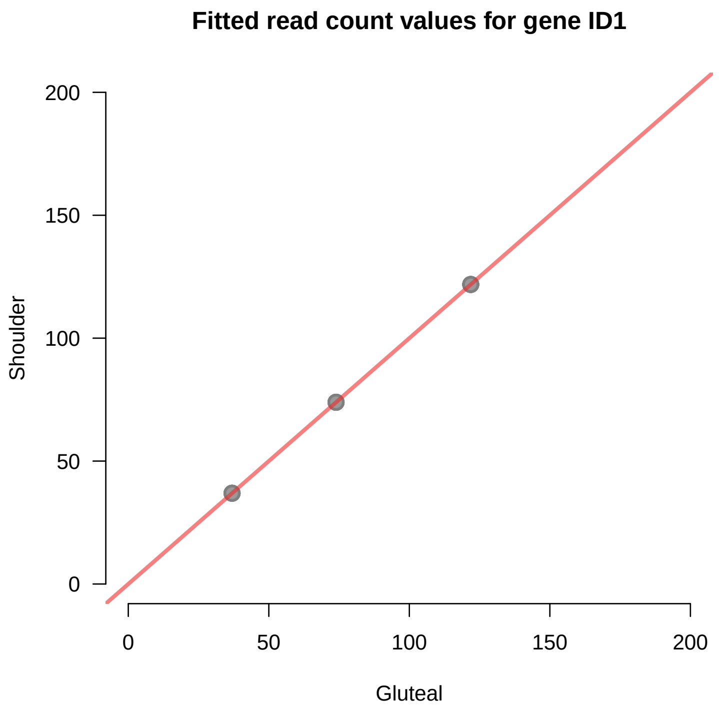

Figure 36: ReadExpSet based genewise CPM estimates

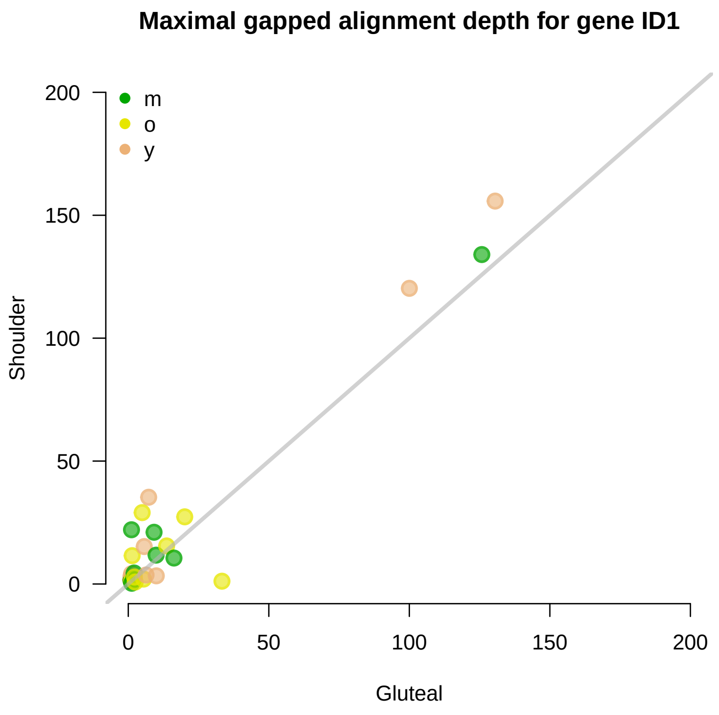

3.7 ERRFI1

Parameter	Value
gene_name	ERRFI1
gene_id	ENSG00000116285
maxald	1776
old	down
seqid	1
strand	-
start	8004404
end	8026308
descr	ERBB receptor feedback inhibitor 1

Table 8: Gene identifcation

Gene expression estimates for Age

Influence of UV exposition on gene expression

Figure 37: Gene expression estimates based on CPM (SummarizeOveraps)

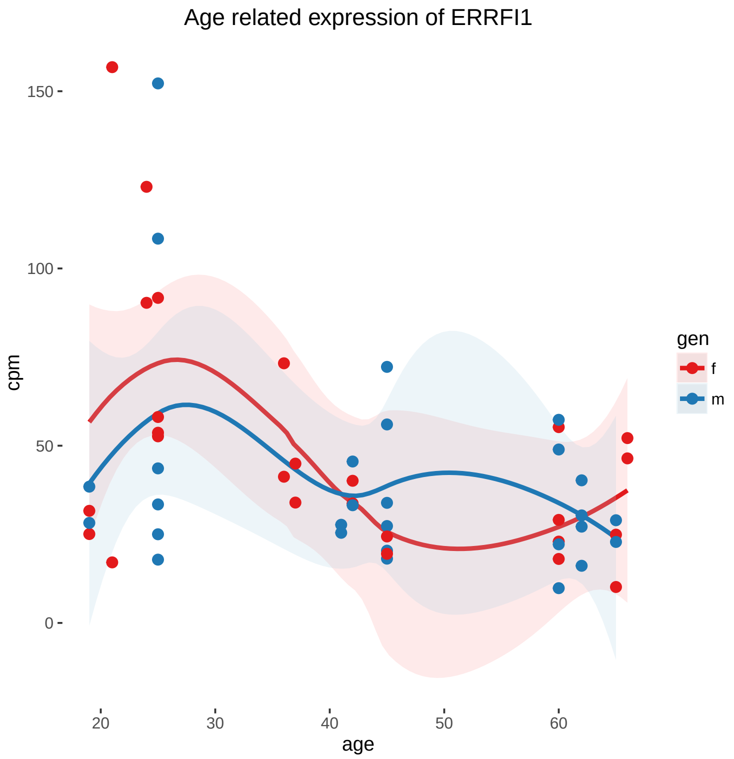

Figure 38: edgeR QLF test based CPM estimates

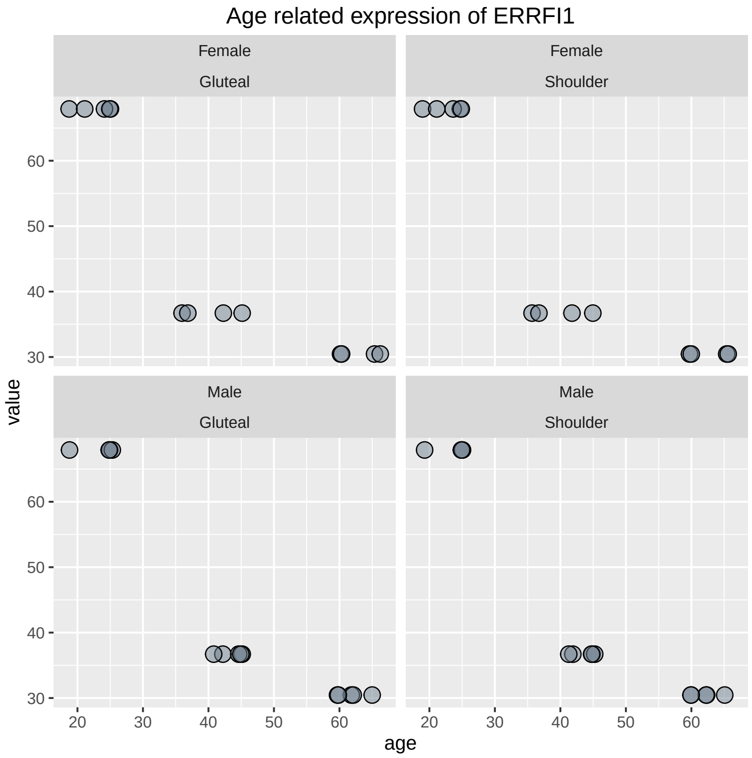

Figure 39: ReadExpSet based genewise CPM estimates

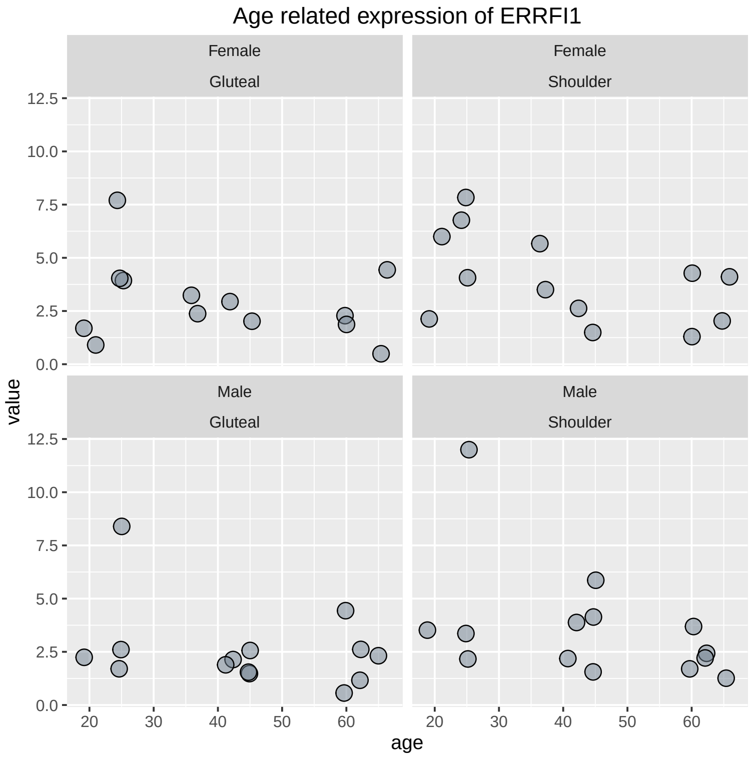

Figure 40: Loess regression for exon align depth

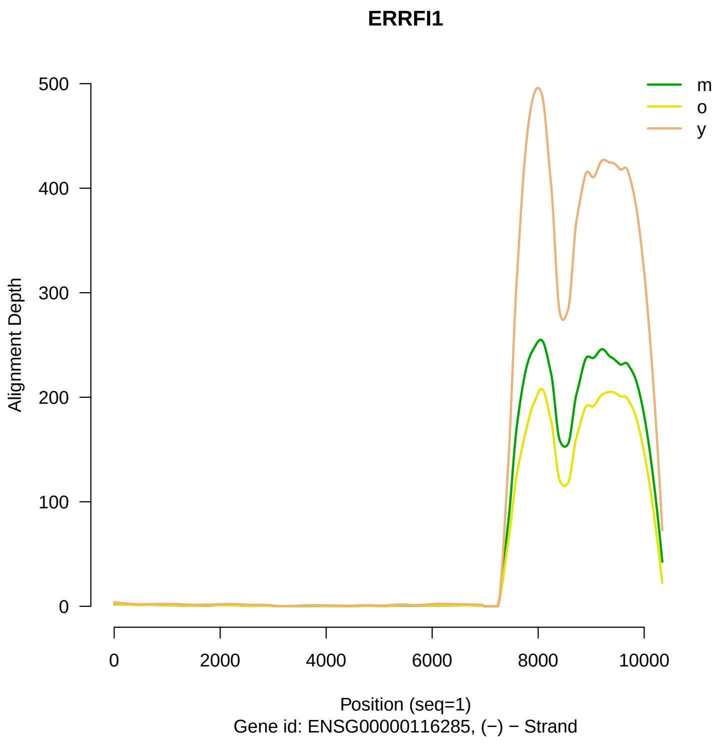

Figure 41: edgeR QLF test based CPM estimates

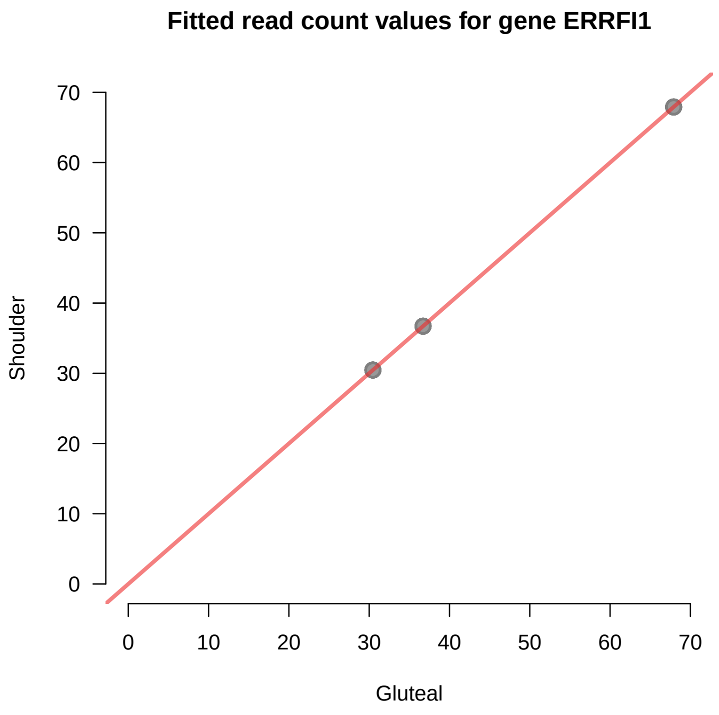

Figure 42: ReadExpSet based genewise CPM estimates

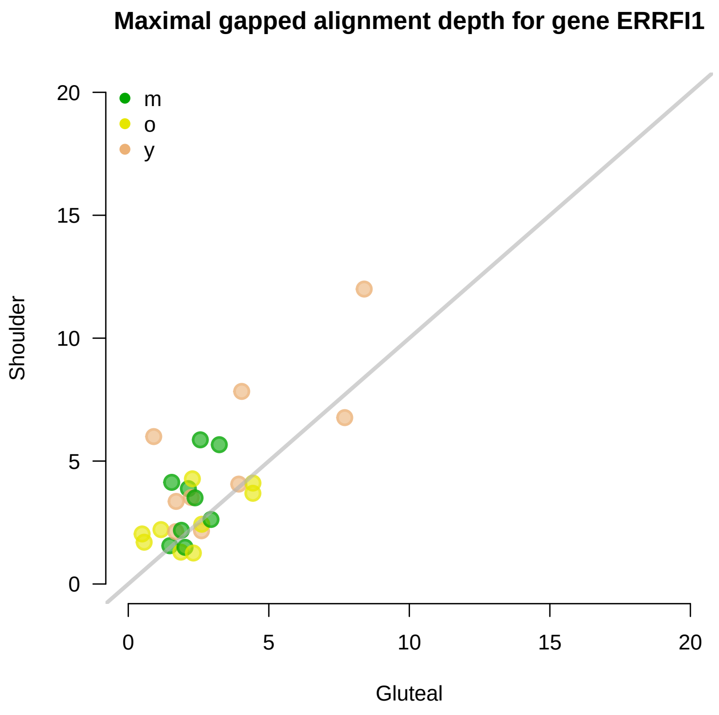

3.8 PENK

Parameter	Value
gene_name	PENK
gene_id	ENSG00000181195
maxAld	2554
old	up
seqid	8
strand	-
start	56436674
end	56446734
descr	proenkephalin

Table 9: Gene identifcation

Gene expression estimates for Age

Influence of UV exposition on gene expression

Figure 43: Gene expression estimates based on CPM (SummarizeOveraps)

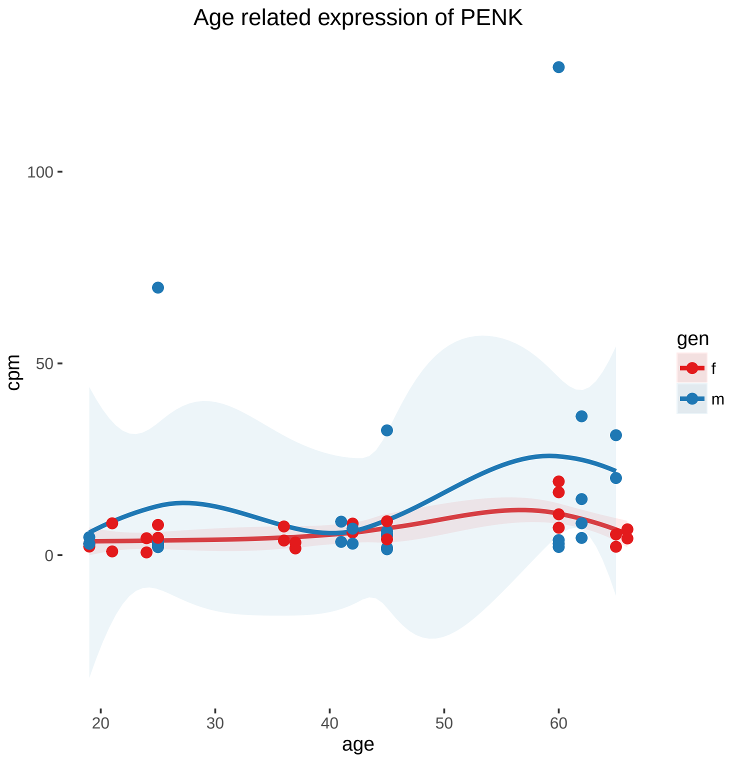

Figure 44: edgeR QLF test based CPM estimates

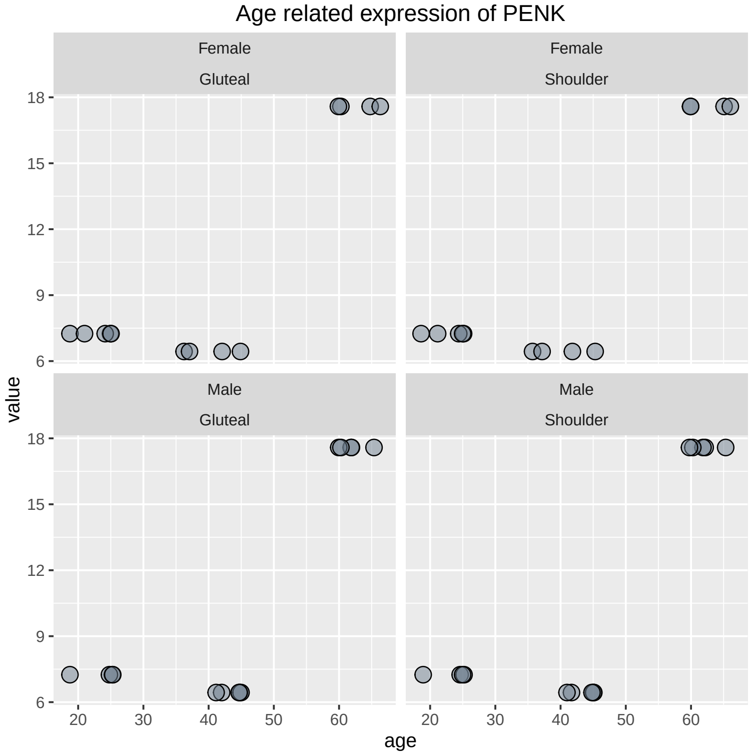

Figure 45: ReadExpSet based genewise CPM estimates

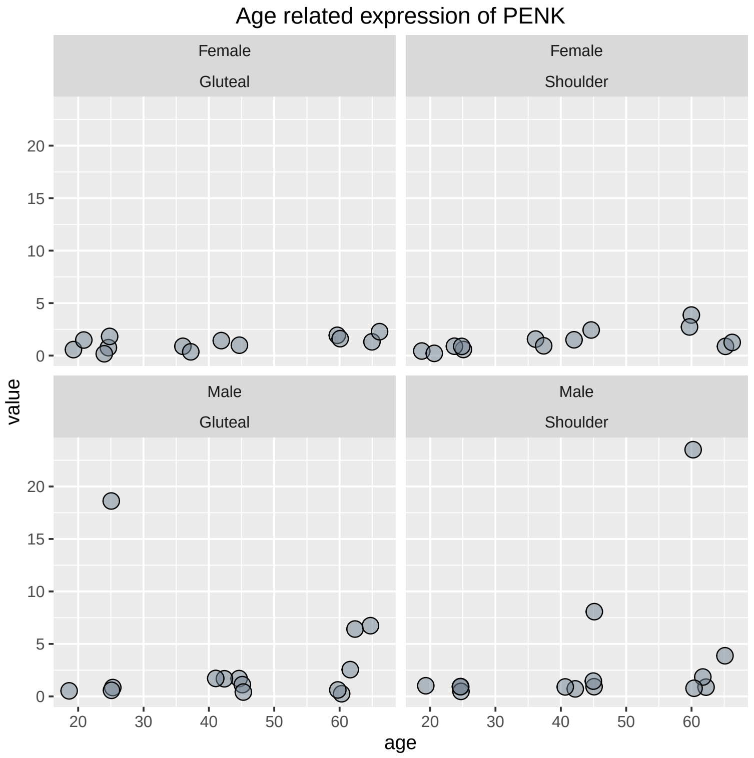

Figure 46: Loess regression for exon align depth

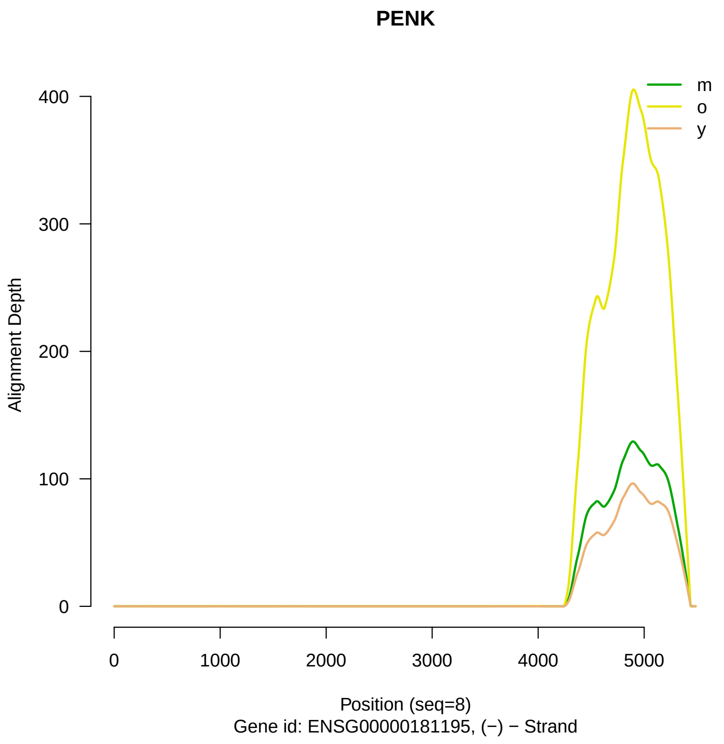

Figure 47: edgeR QLF test based CPM estimates

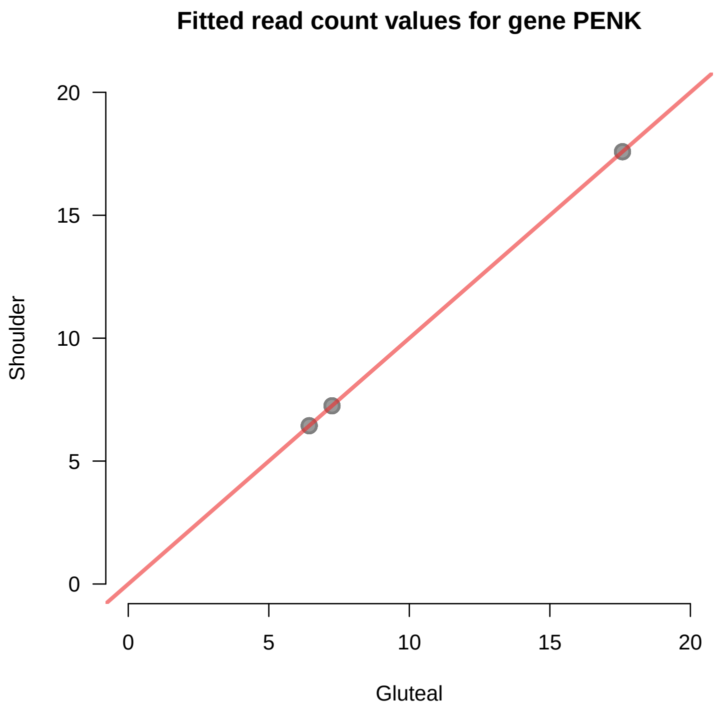

Figure 48: ReadExpSet based genewise CPM estimates

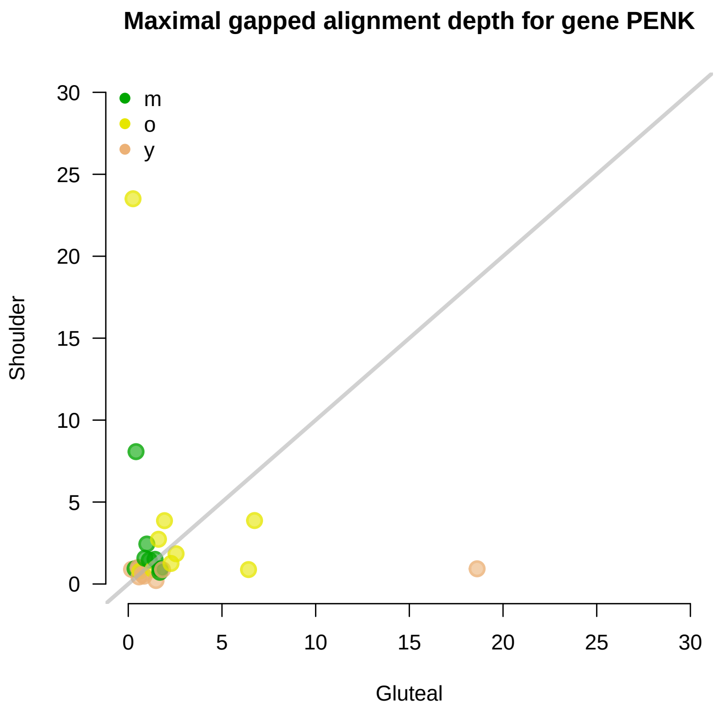

3.9 SEPT5

Parameter	Value
gene_name	SEPT5
gene_id	ENSG00000184702
maxald	1468
old	down
seqid	22
strand	+
start	19714464
end	19724772
descr	septin 5

Table 10: Gene identifcation

Gene expression estimates for Age

Influence of UV exposition on gene expression

Figure 49: Gene expression estimates based on CPM (SummarizeOveraps)

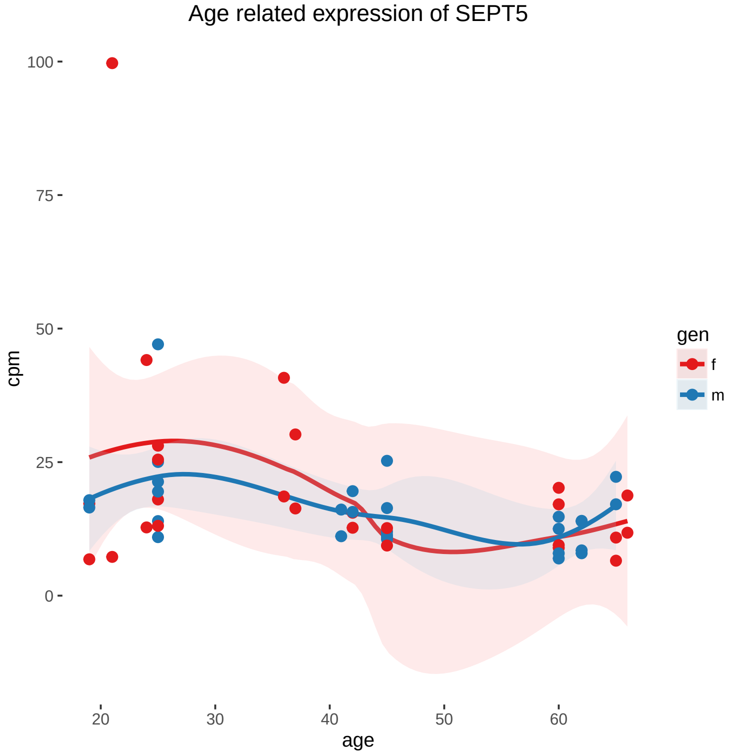

Figure 50: edgeR QLF test based CPM estimates

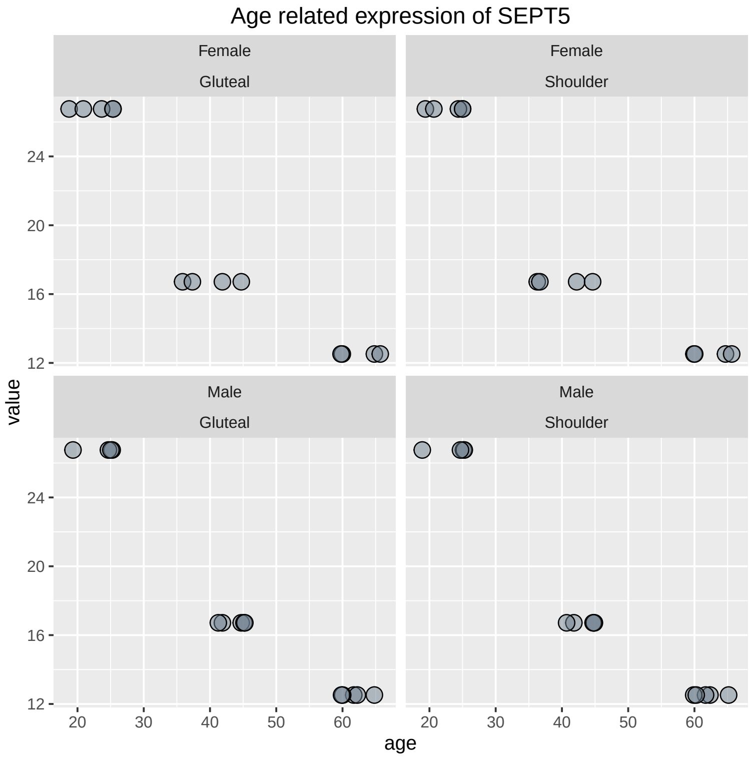

Figure 51: ReadExpSet based genewise CPM estimates

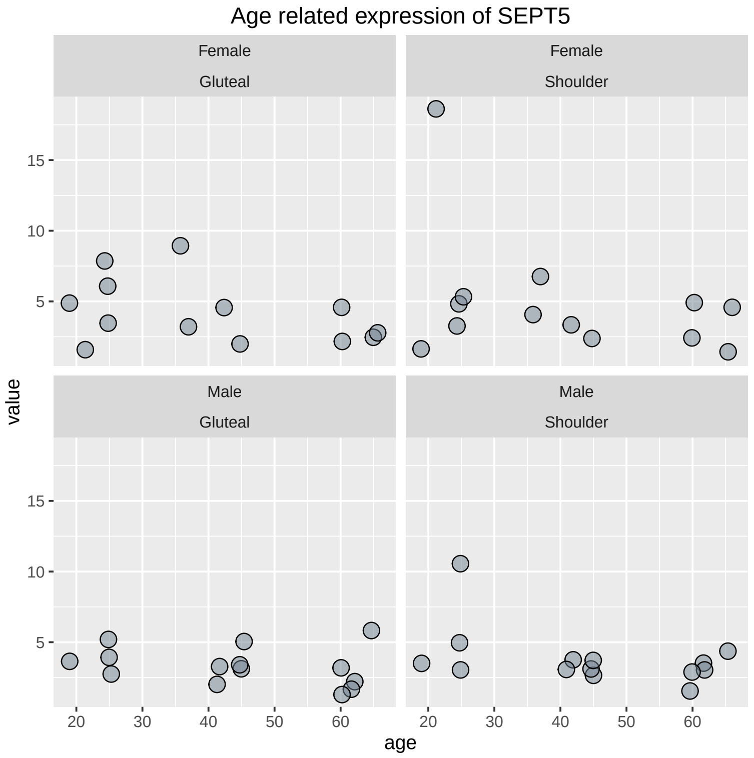

Figure 52: Loess regression for exon align depth

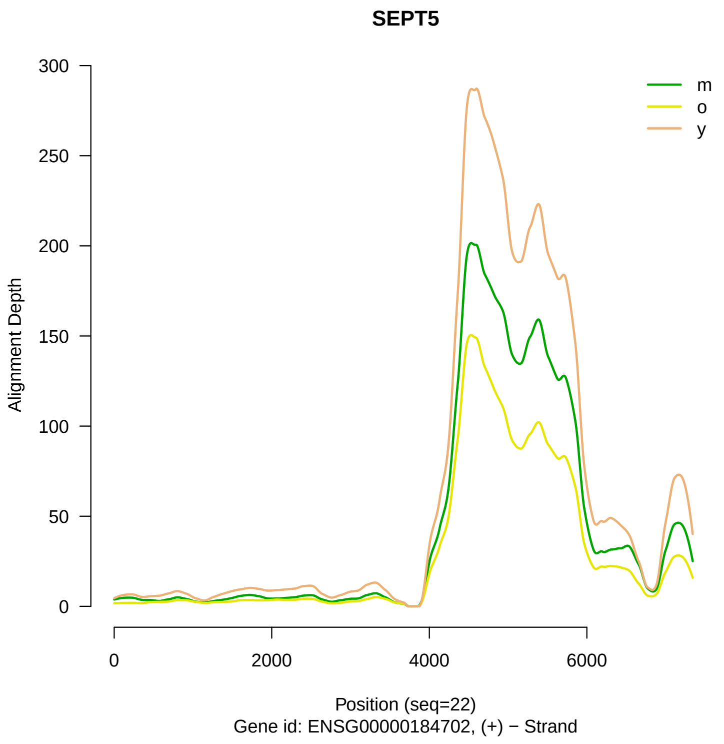

Figure 53: edgeR QLF test based CPM estimates

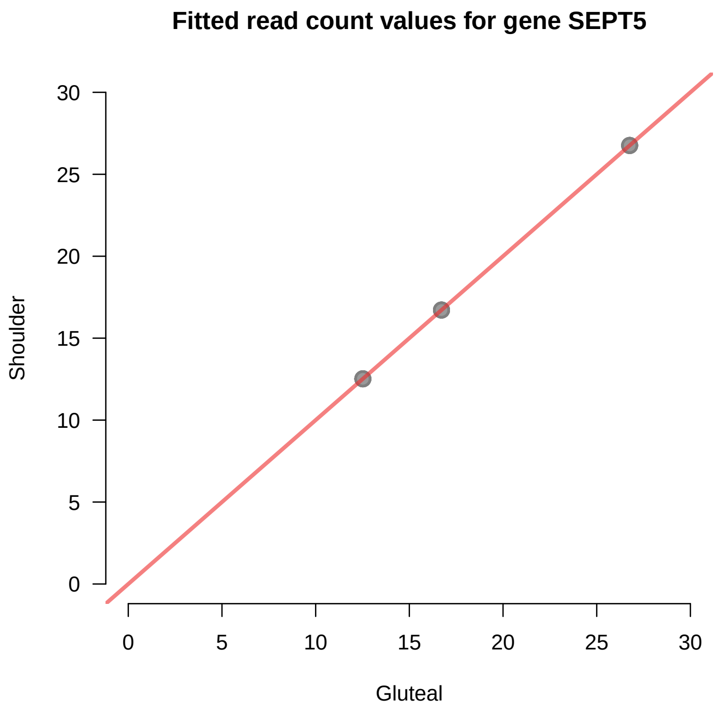

Figure 54: ReadExpSet based genewise CPM estimates

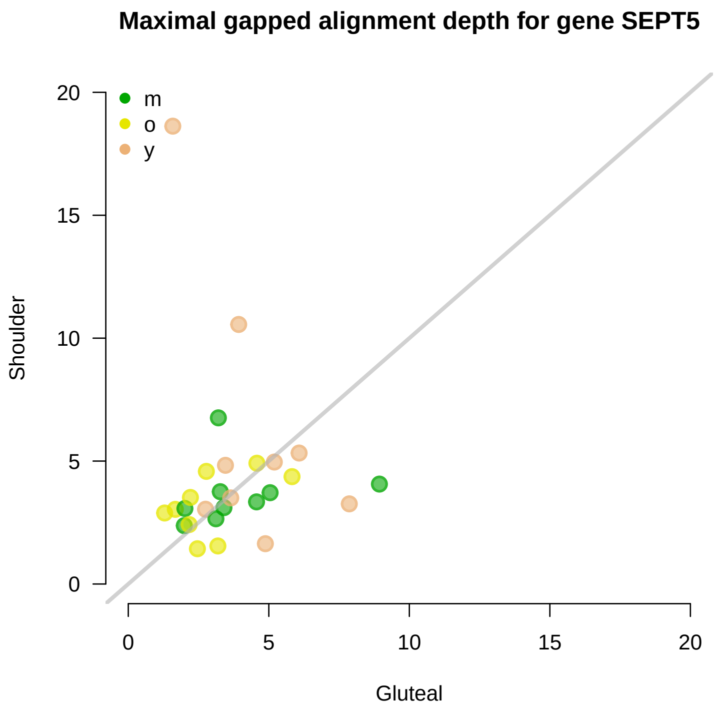

3.10 CPZ

Parameter	Value
gene_name	CPZ
gene_id	ENSG00000109625
maxald	2585
old	up
seqid	4
strand	+
start	8592660
end	8619759
descr	carboxypeptidase Z

Table 11: Gene identifcation

Gene expression estimates for Age

Influence of UV exposition on gene expression

Figure 55: Gene expression estimates based on CPM (SummarizeOveraps)

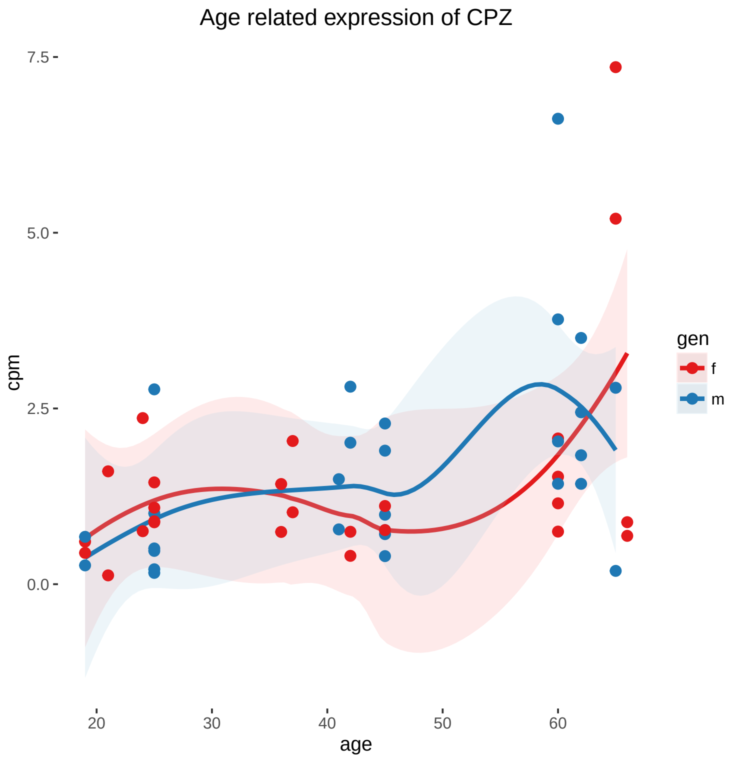

Figure 56: edgeR QLF test based CPM estimates

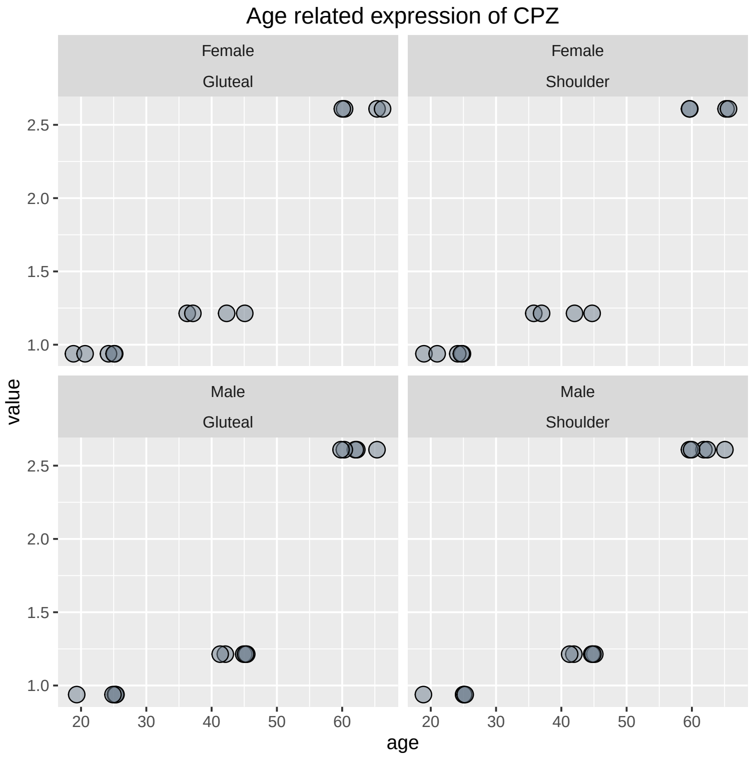

Figure 57: ReadExpSet based genewise CPM estimates

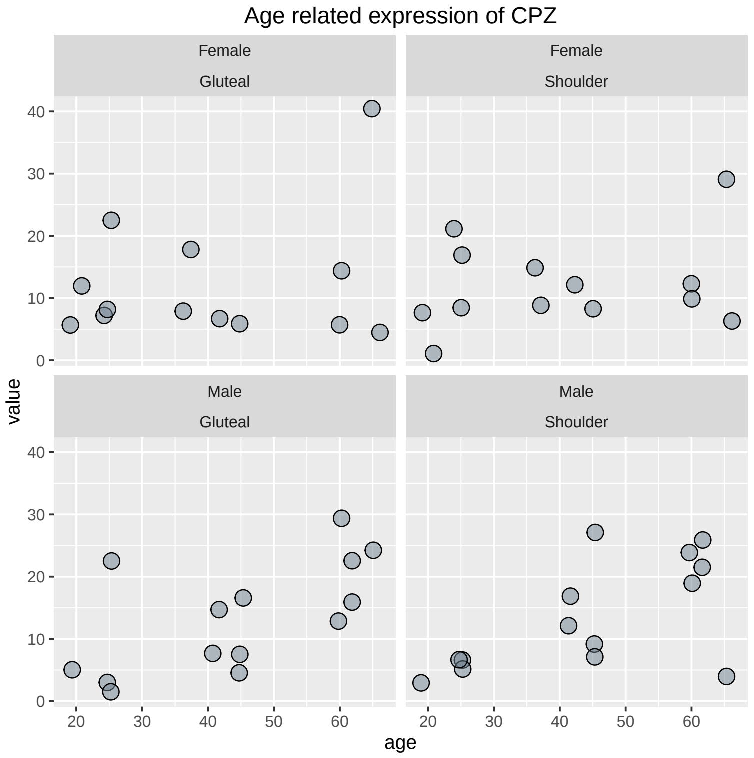

Figure 58: Loess regression for exon align depth

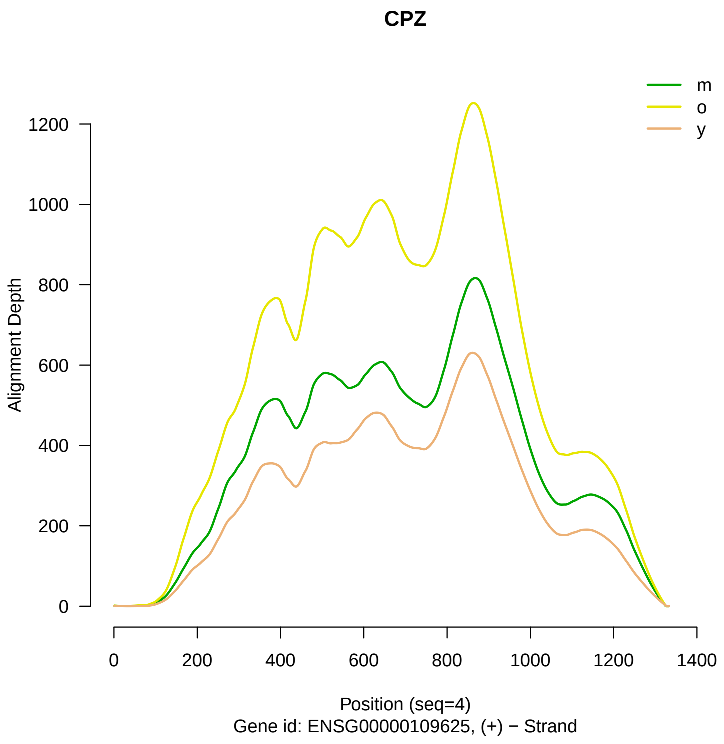

Figure 59: edgeR QLF test based CPM estimates

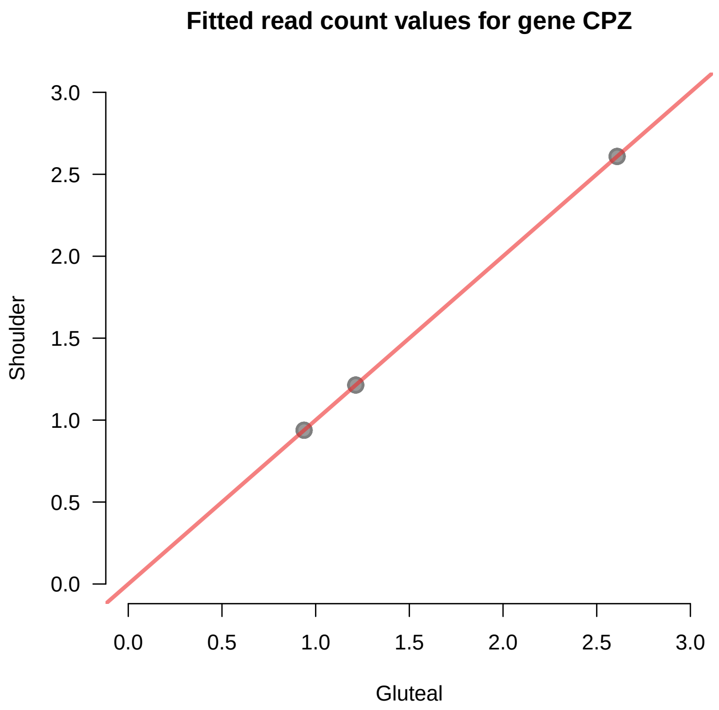

Figure 60: ReadExpSet based genewise CPM estimates

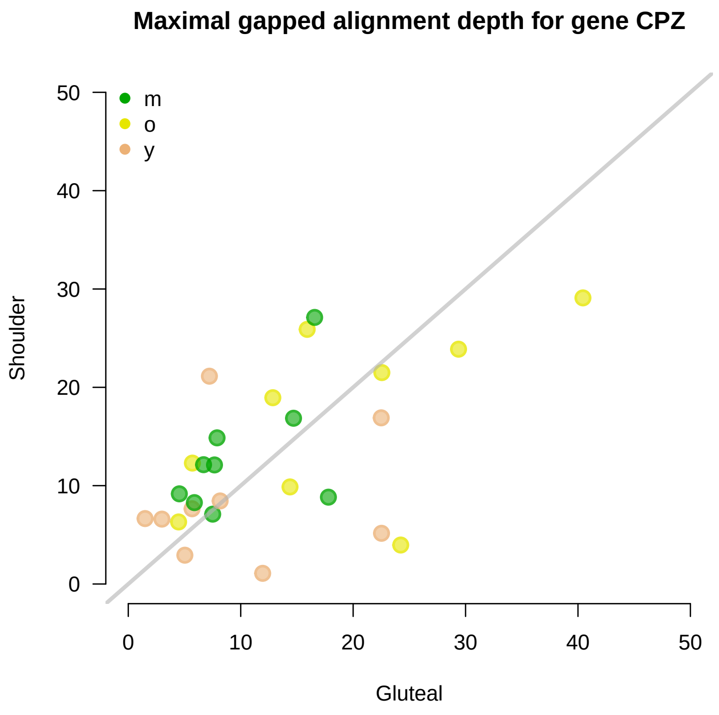

3.11 PRPS1

Parameter	Value
gene_name	PRPS1
gene_id	ENSG00000147224
maxald	5550
old	down
seqid	X
strand	+
start	107628424
end	107651026
descr	phosphoribosyl pyrophosphate synthetase 1

Table 12: Gene identifcation

Gene expression estimates for Age

Influence of UV exposition on gene expression

Figure 61: Gene expression estimates based on CPM (SummarizeOveraps)

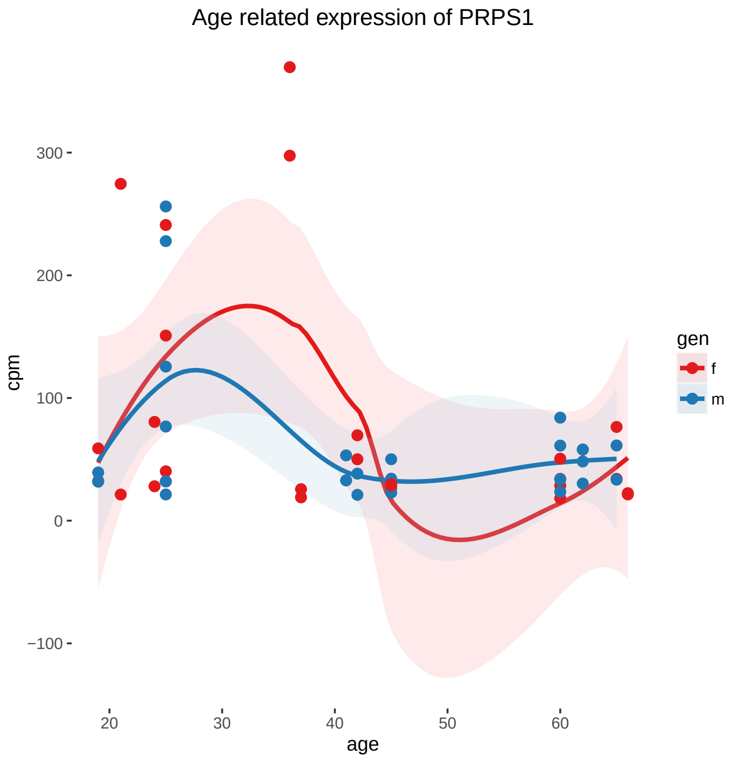

Figure 62: edgeR QLF test based CPM estimates

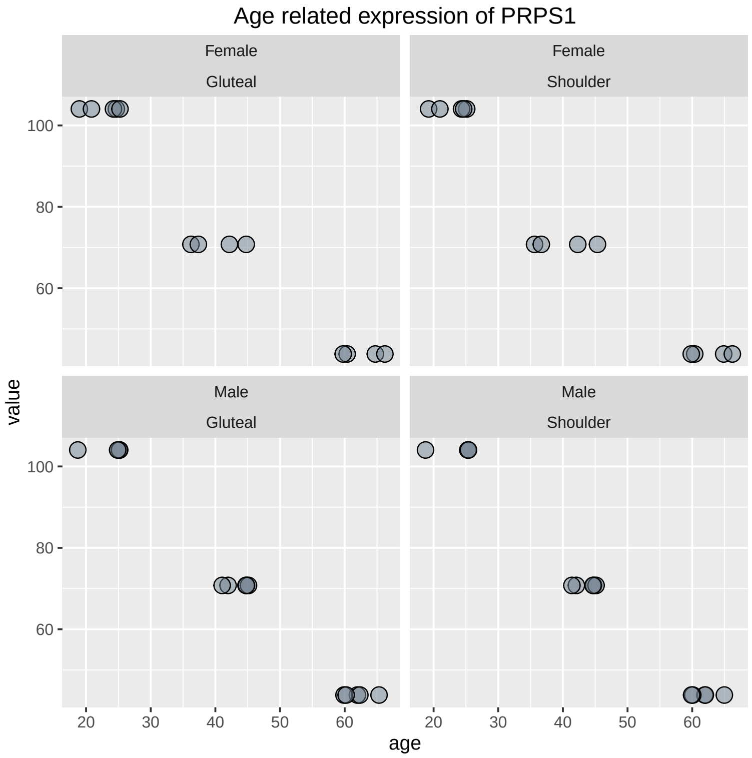

Figure 63: ReadExpSet based genewise CPM estimates

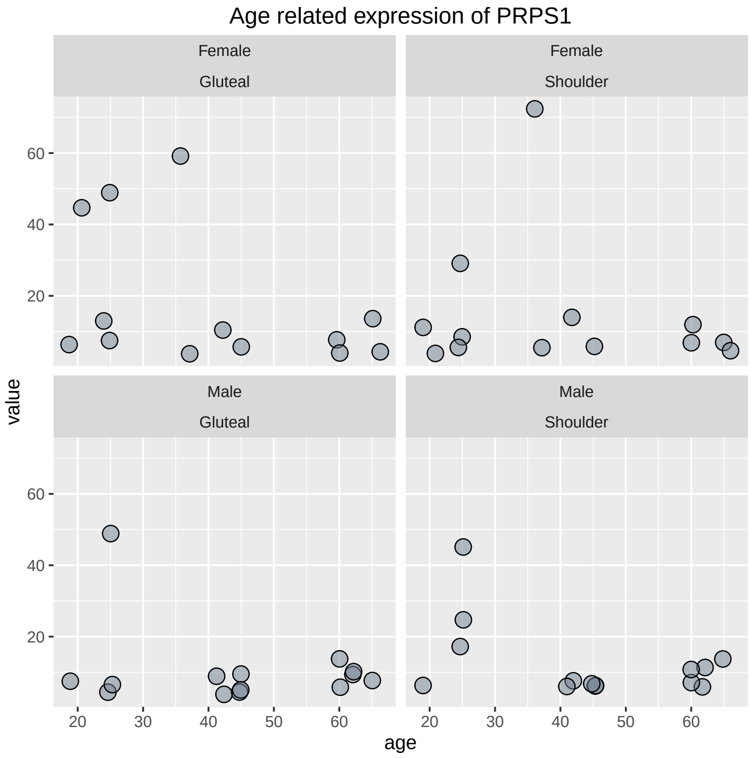

Figure 64: Loess regression for exon align depth

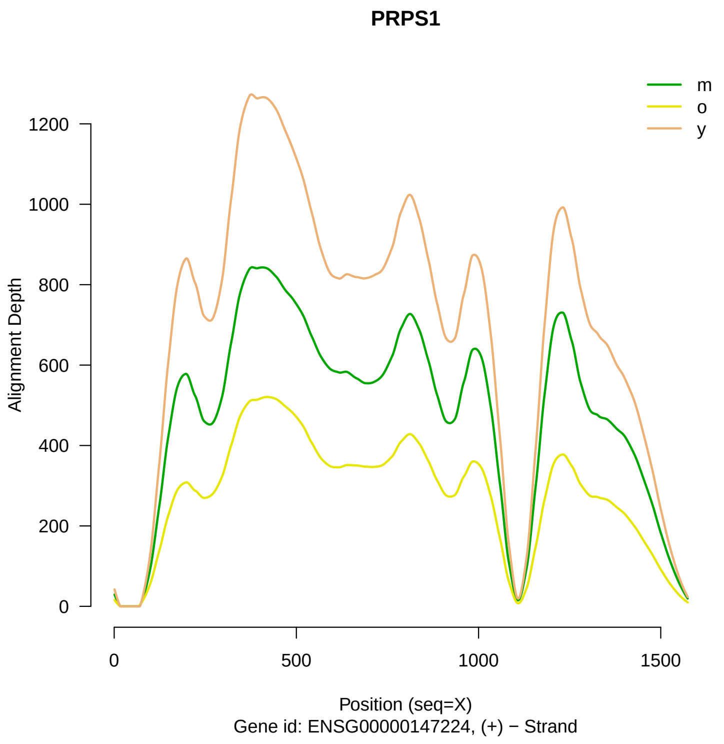

Figure 65: edgeR QLF test based CPM estimates

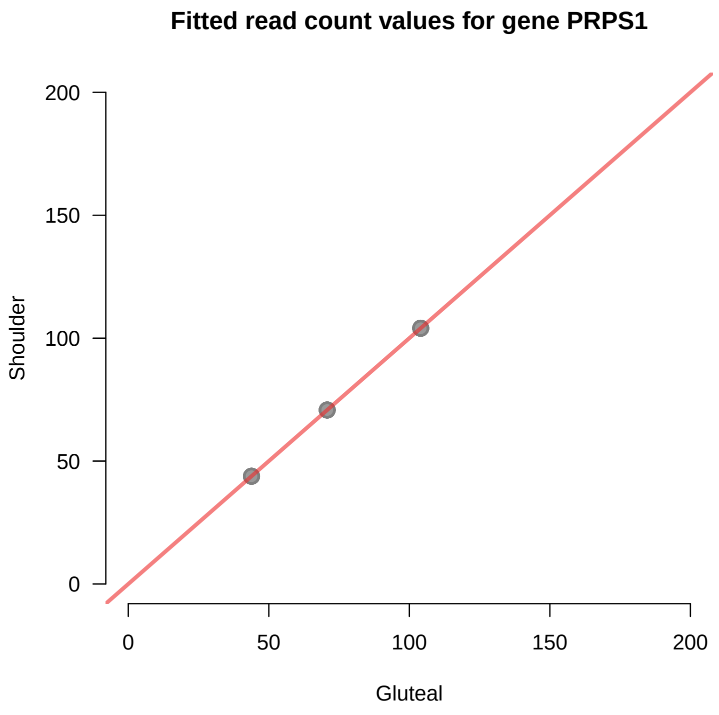

Figure 66: ReadExpSet based genewise CPM estimates

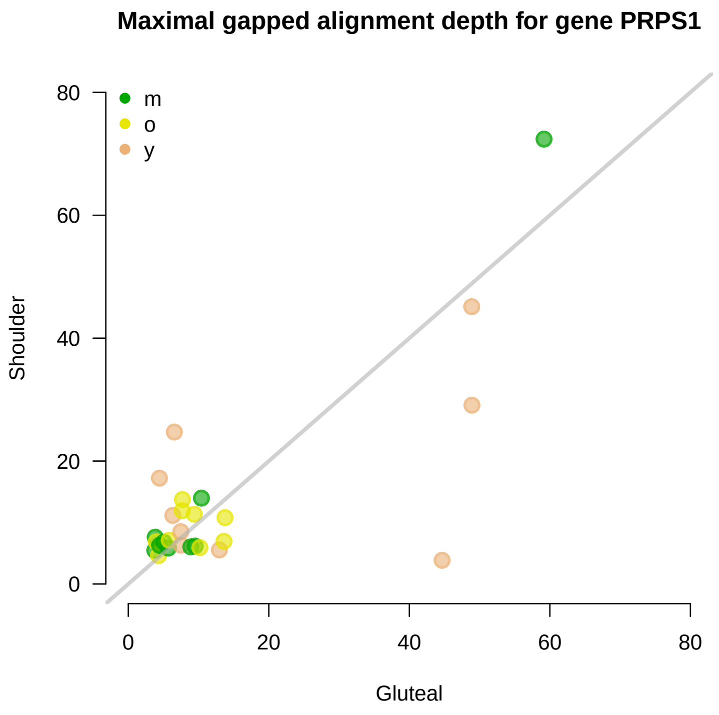

3.12 MEG3

Parameter	Value
gene_name	MEG3
gene_id	ENSG00000214548
maxald	3031
old	down
seqid	14
strand	+
start	100779410
end	100861031
descr	maternally expressed 3 (non-protein coding)

Table 13: Gene identifcation

Gene expression estimates for Age

Influence of UV exposition on gene expression

Figure 67: Gene expression estimates based on CPM (SummarizeOveraps)

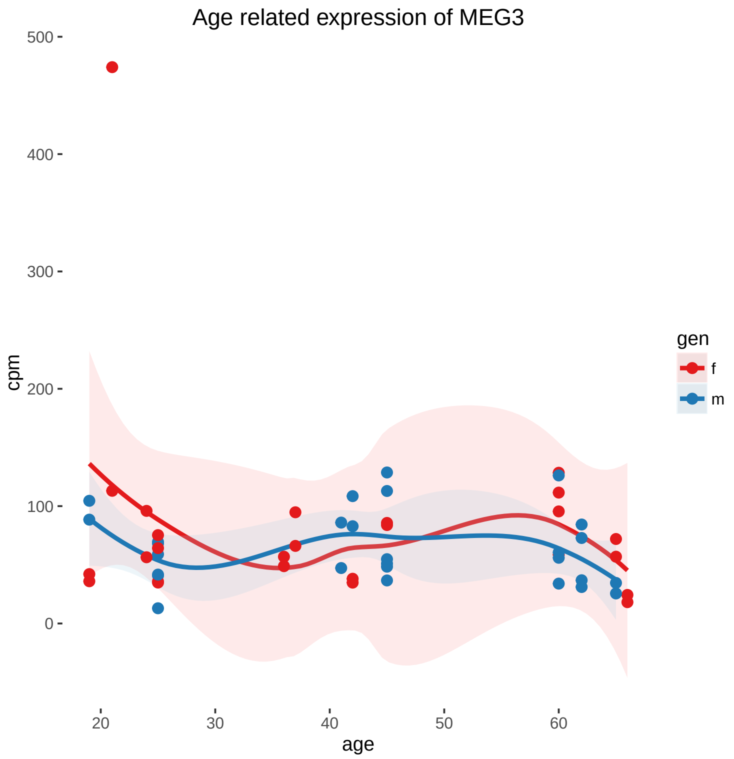

Figure 68: edgeR QLF test based CPM estimates

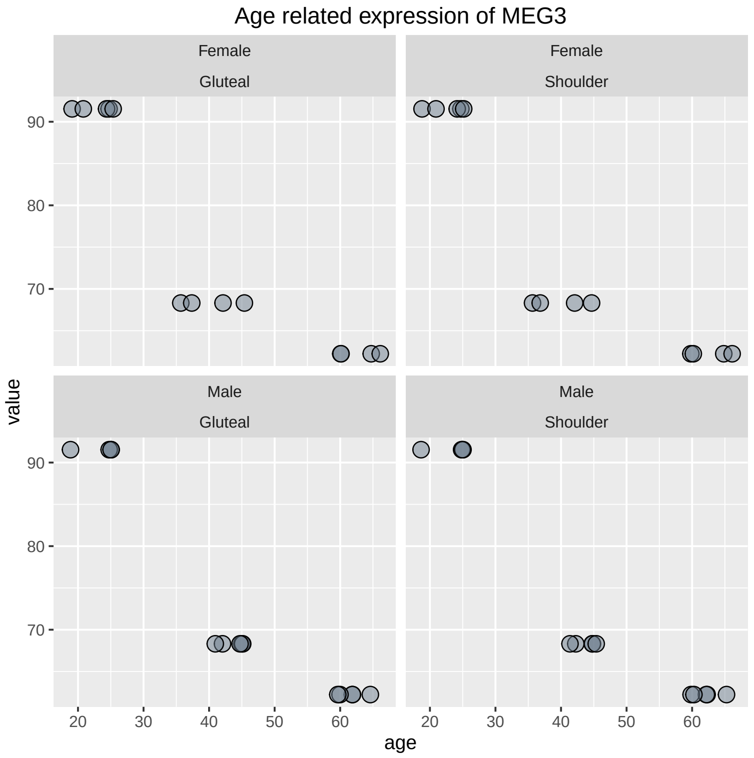

Figure 69: ReadExpSet based genewise CPM estimates

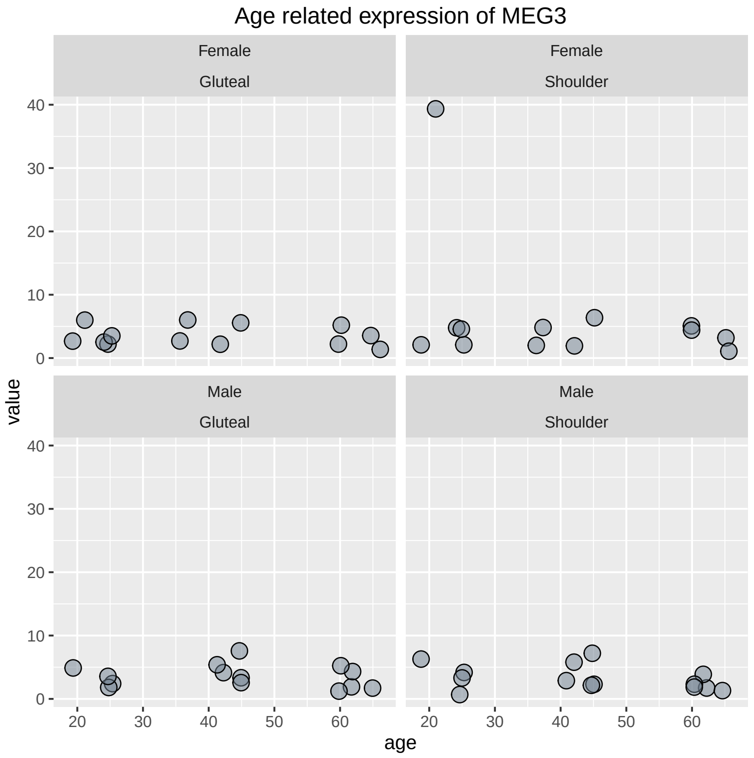

Figure 70: Loess regression for exon align depth

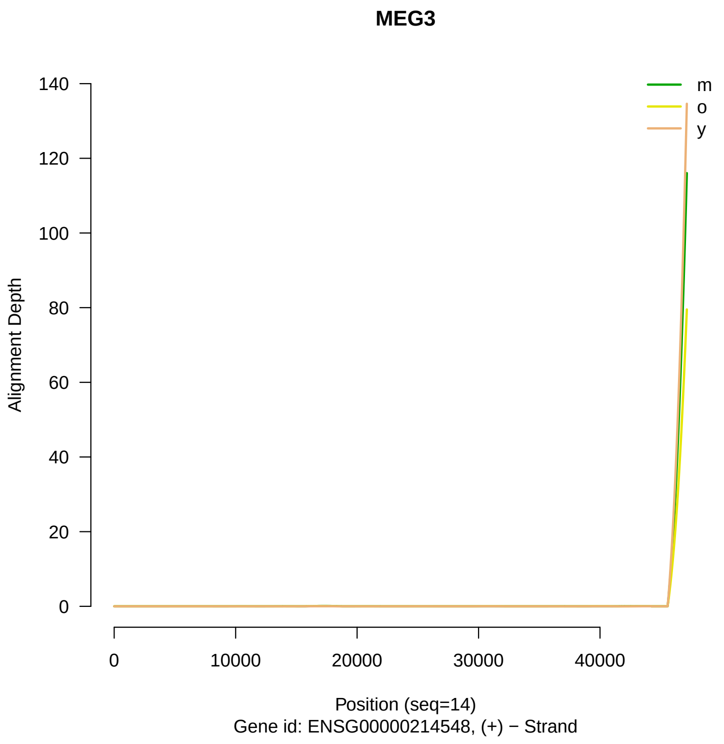

Figure 71: edgeR QLF test based CPM estimates

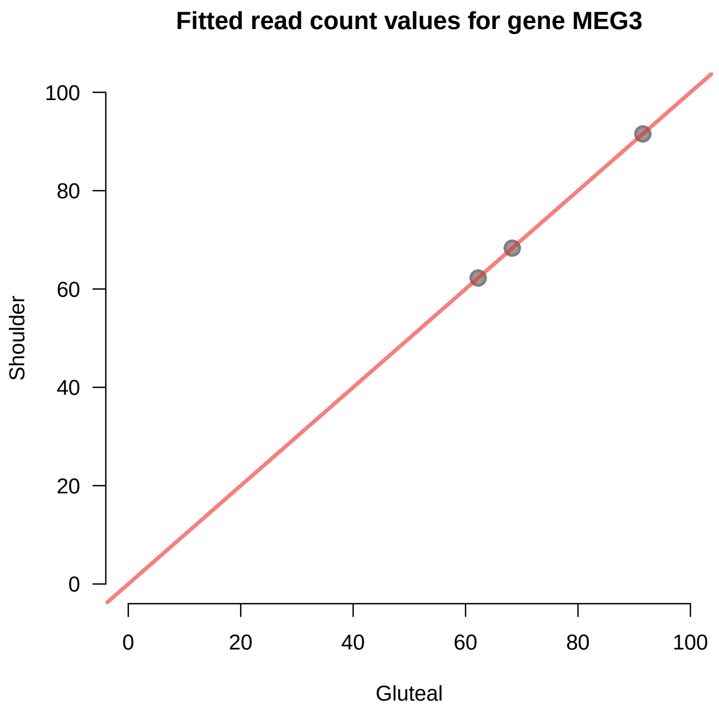

Figure 72: ReadExpSet based genewise CPM estimates

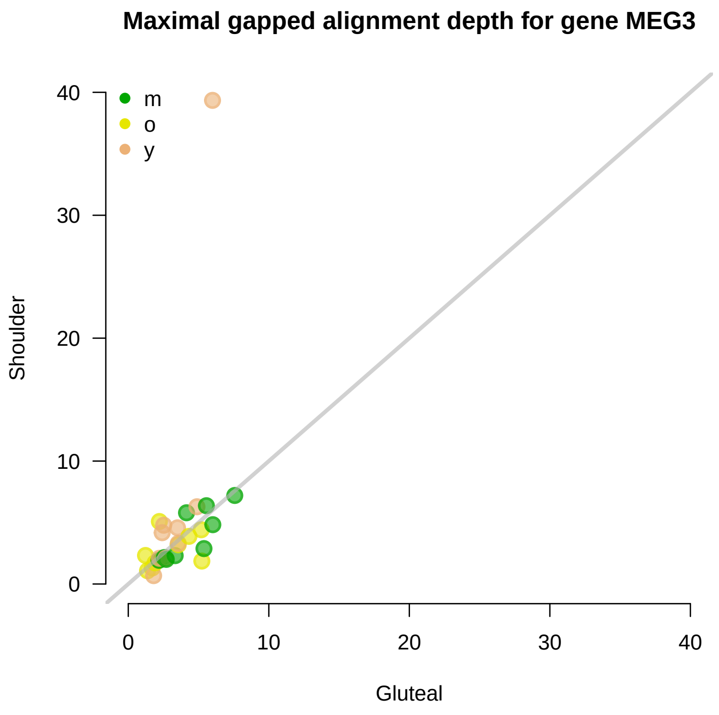

3.13 CNN1

Parameter	Value
gene_name	CNN1
gene_id	ENSG00000130176
maxald	16224
old	down
seqid	19
strand	+
start	11538717
end	11550323
descr	calponin 1, basic, smooth muscle

Table 14: Gene identifcation

Gene expression estimates for Age

Influence of UV exposition on gene expression

Figure 73: Gene expression estimates based on CPM (SummarizeOveraps)

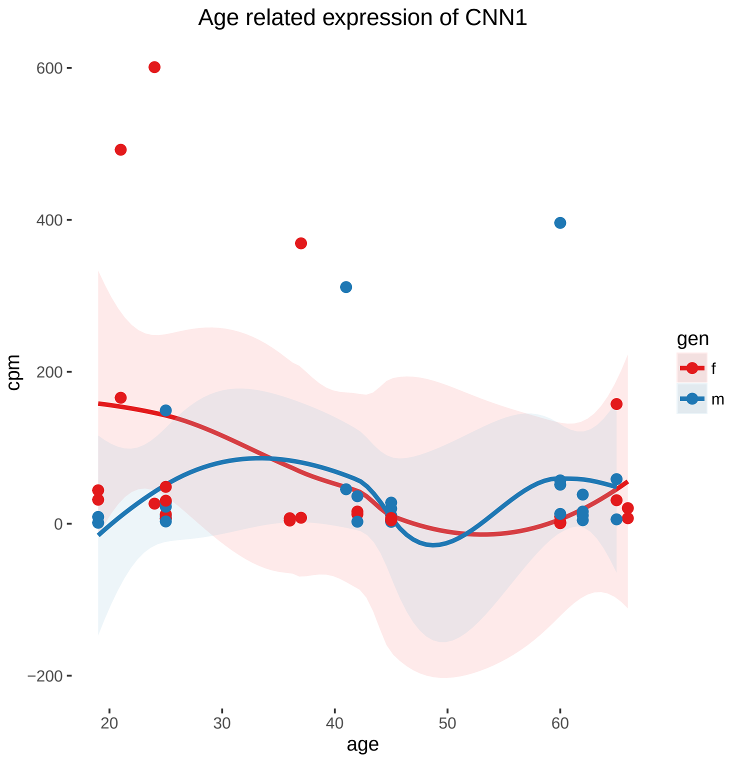

Figure 74: edgeR QLF test based CPM estimates

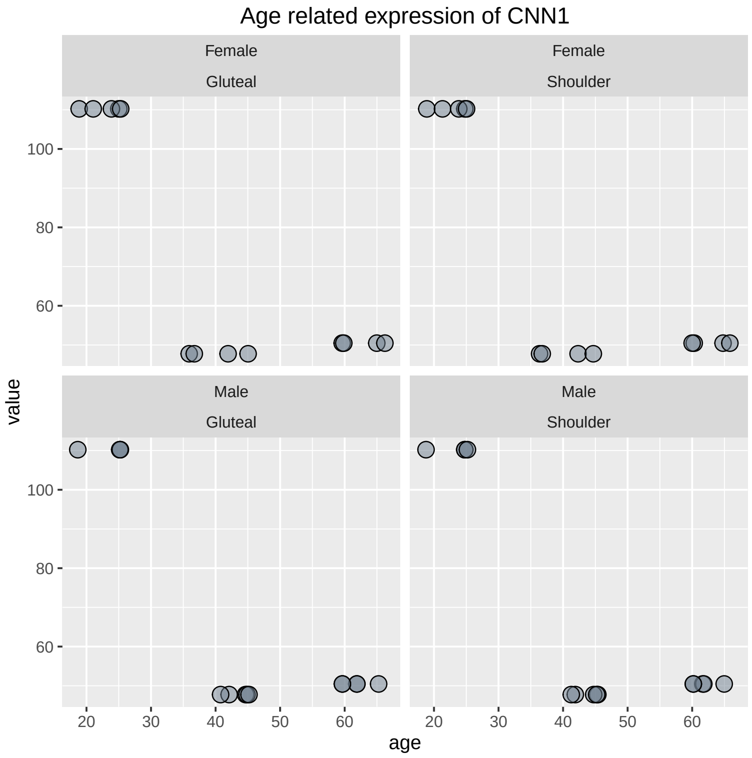

Figure 75: ReadExpSet based genewise CPM estimates

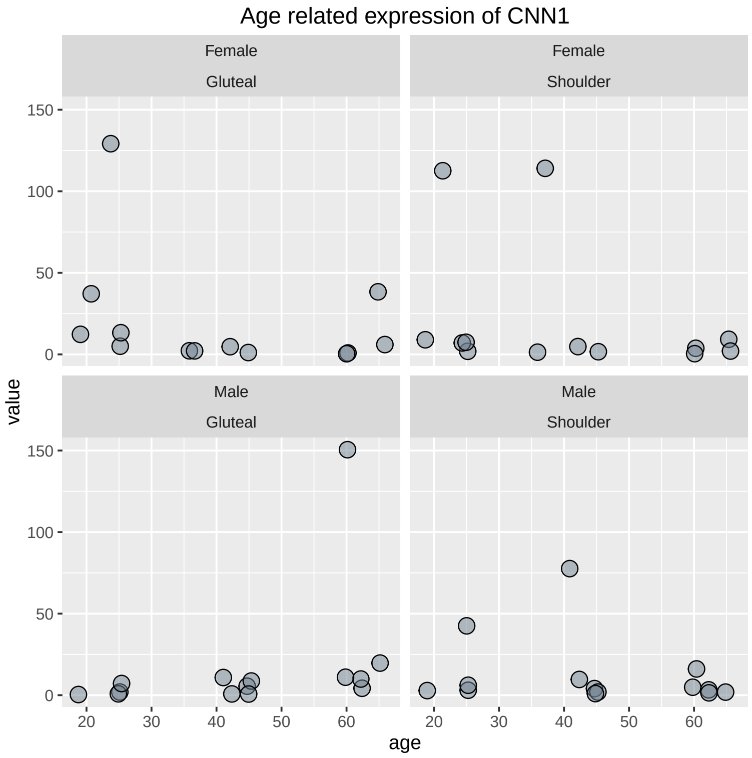

Figure 76: Loess regression for exon align depth

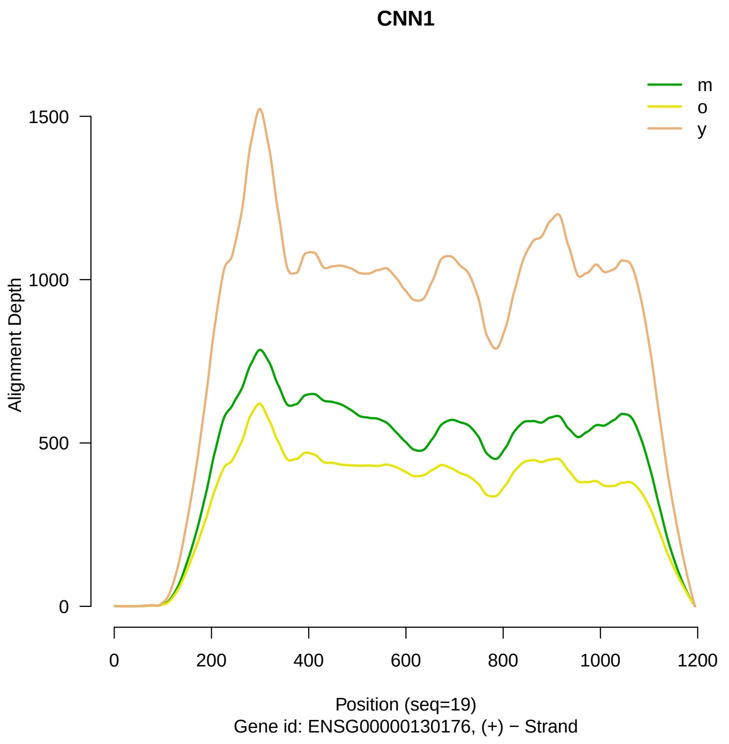

Figure 77: edgeR QLF test based CPM estimates

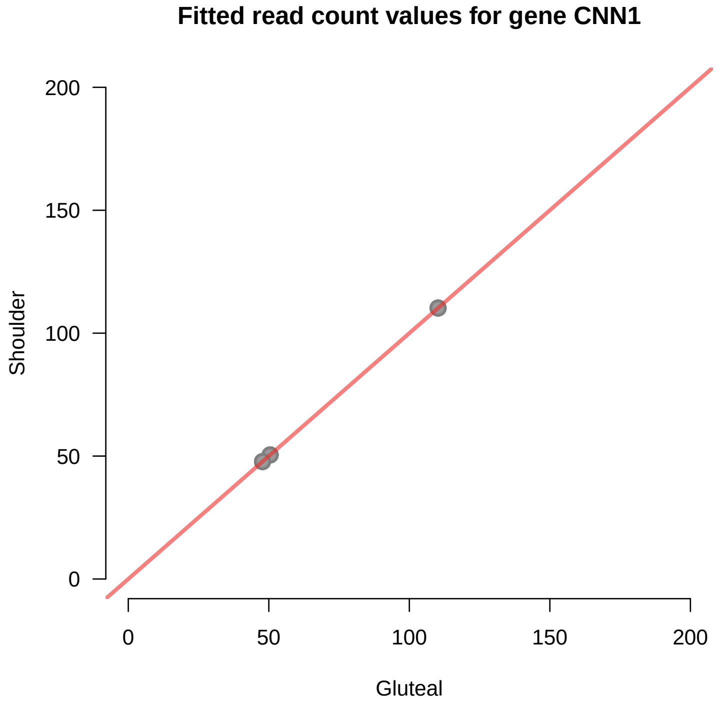

Figure 78: ReadExpSet based genewise CPM estimates

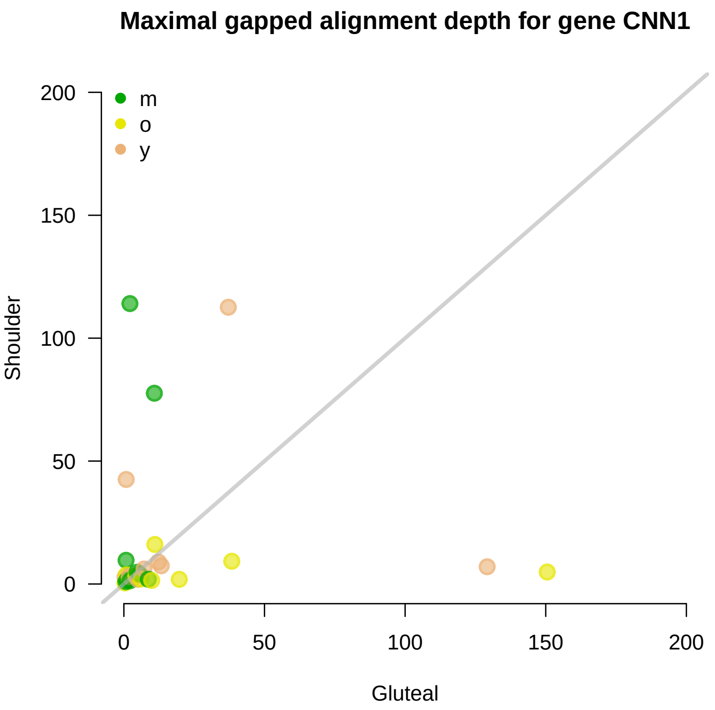

3.14 STC1

Parameter	Value
gene_name	STC1
gene_id	ENSG00000159167
maxald	1877
old	up
seqid	8
strand	-
start	23841915
end	23854807
descr	stanniocalcin 1

Table 15: Gene identifcation

Gene expression estimates for Age

Influence of UV exposition on gene expression

Figure 79: Gene expression estimates based on CPM (SummarizeOveraps)

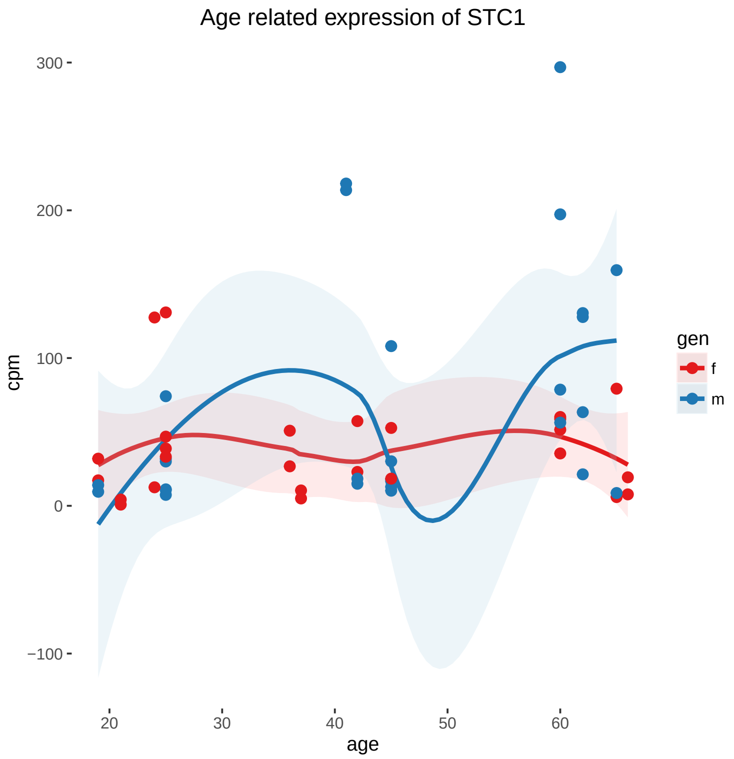

Figure 80: edgeR QLF test based CPM estimates

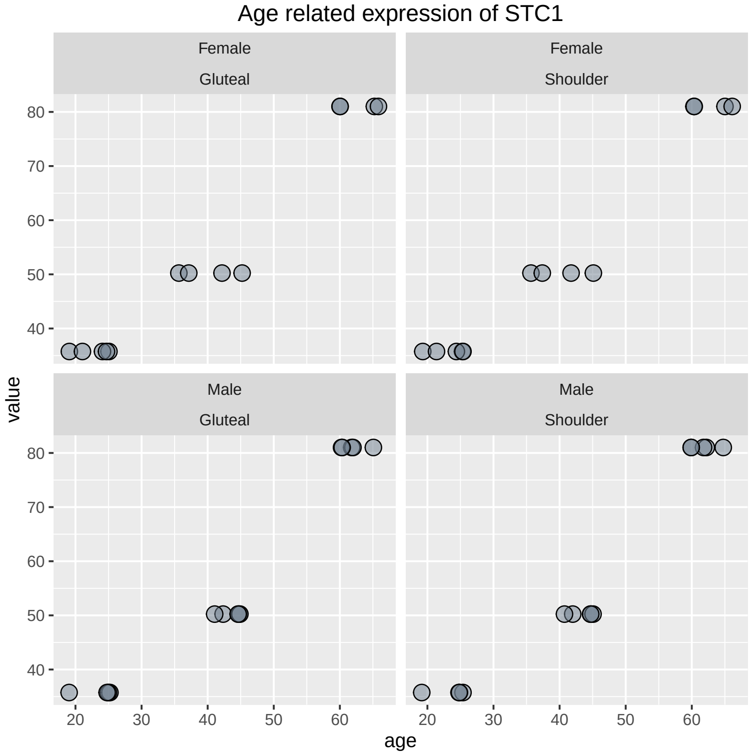

Figure 81: ReadExpSet based genewise CPM estimates

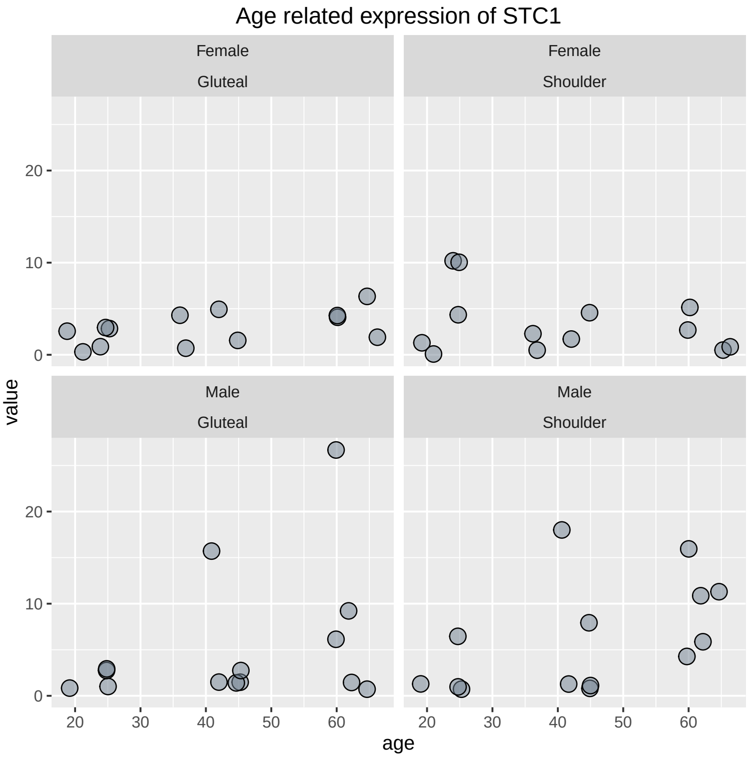

Figure 82: Loess regression for exon align depth

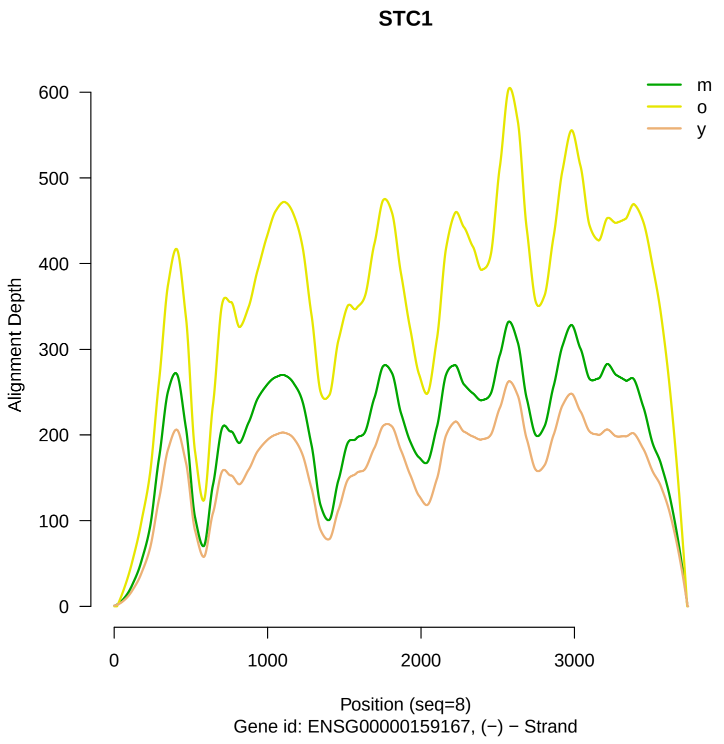

Figure 83: edgeR QLF test based CPM estimates

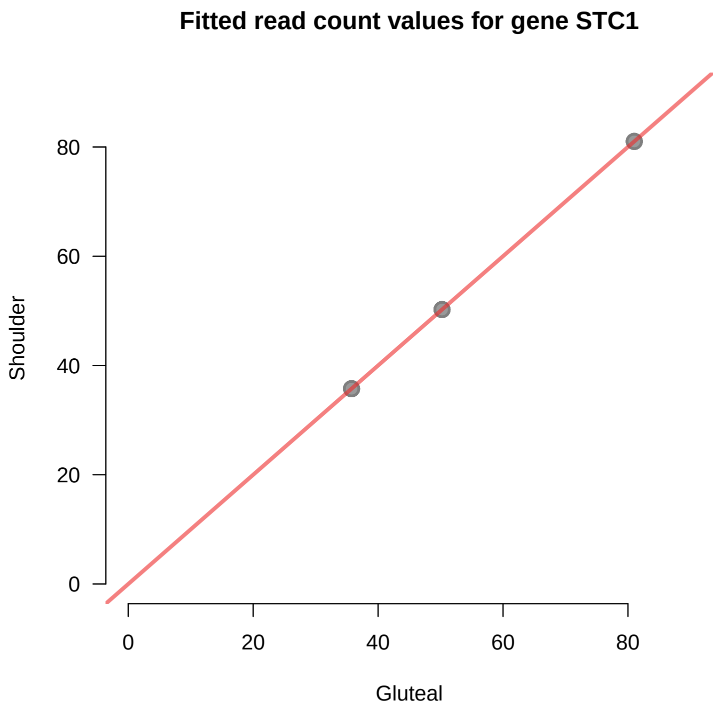

Figure 84: ReadExpSet based genewise CPM estimates

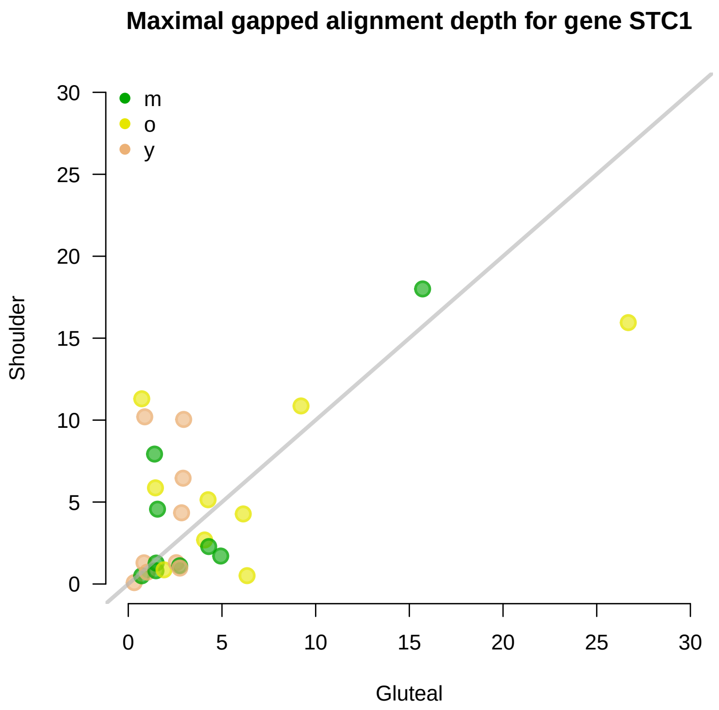

3.15 KIAA1324L

Parameter	Value
gene_name	KIAA1324L
gene_id	ENSG00000164659
maxald	1751
old	up
seqid	7
strand	-
start	86876906
end	87059699
descr	KIAA1324-like

Table 16: Gene identifcation

Gene expression estimates for Age

Influence of UV exposition on gene expression

Figure 85: Gene expression estimates based on CPM (SummarizeOveraps)

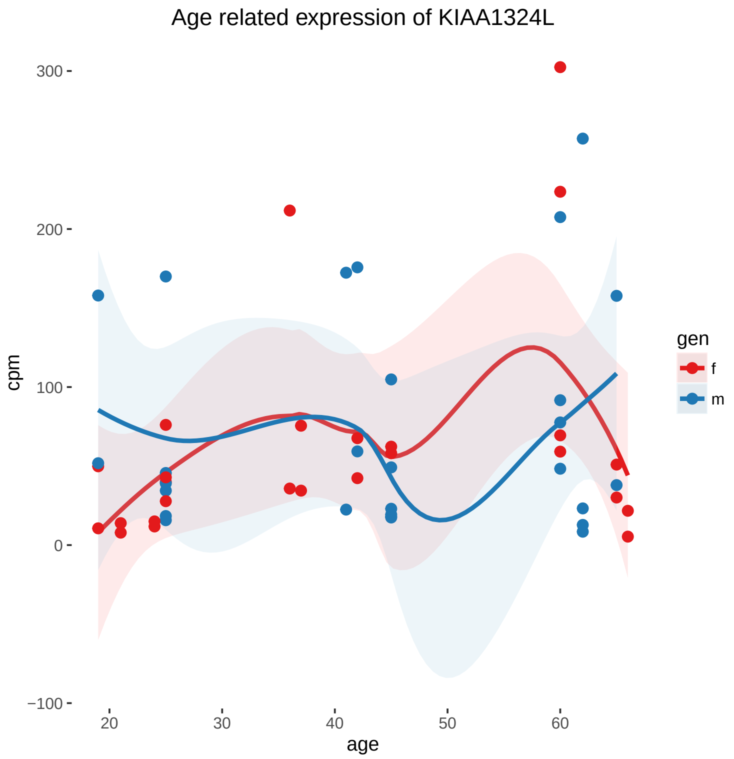

Figure 86: edgeR QLF test based CPM estimates

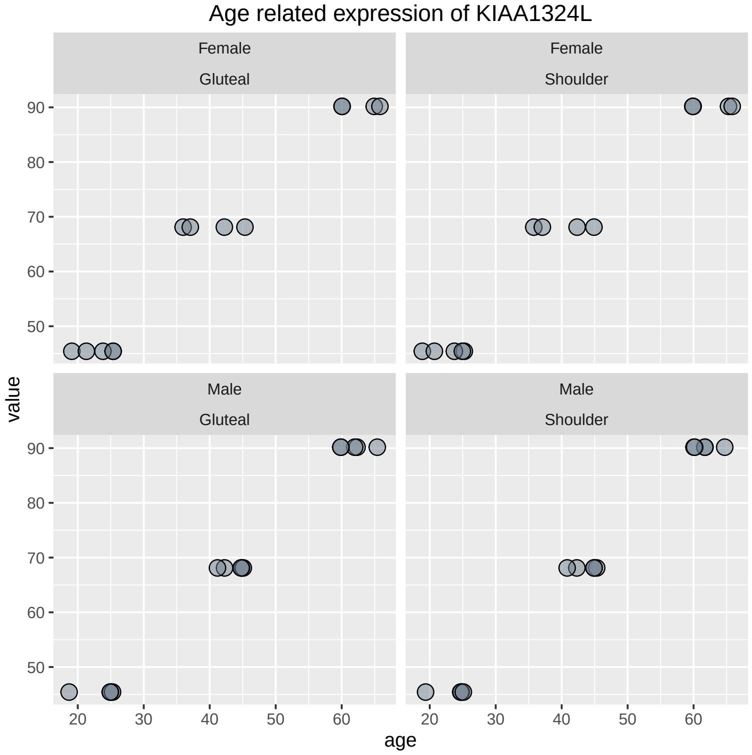

Figure 87: ReadExpSet based genewise CPM estimates

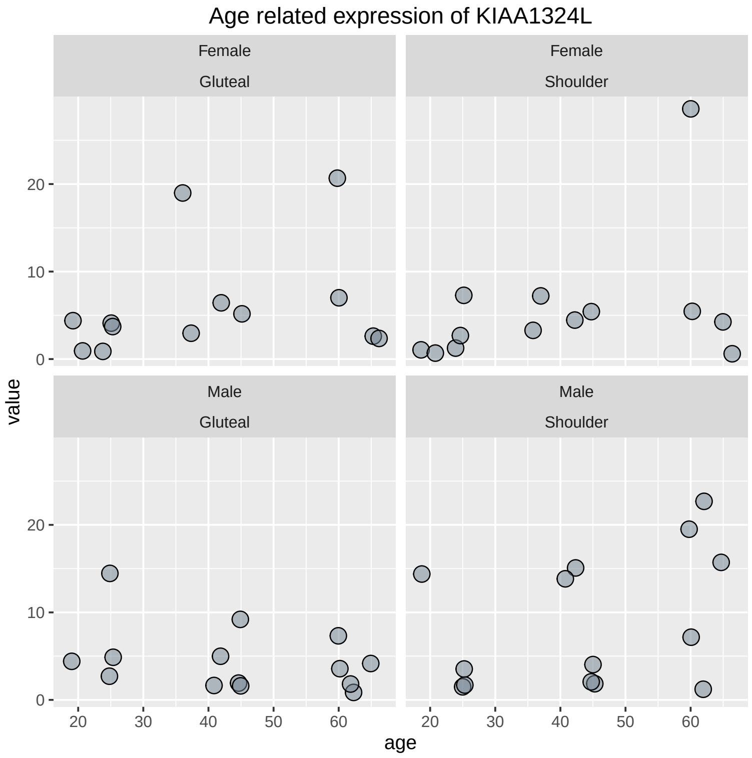

Figure 88: Loess regression for exon align depth

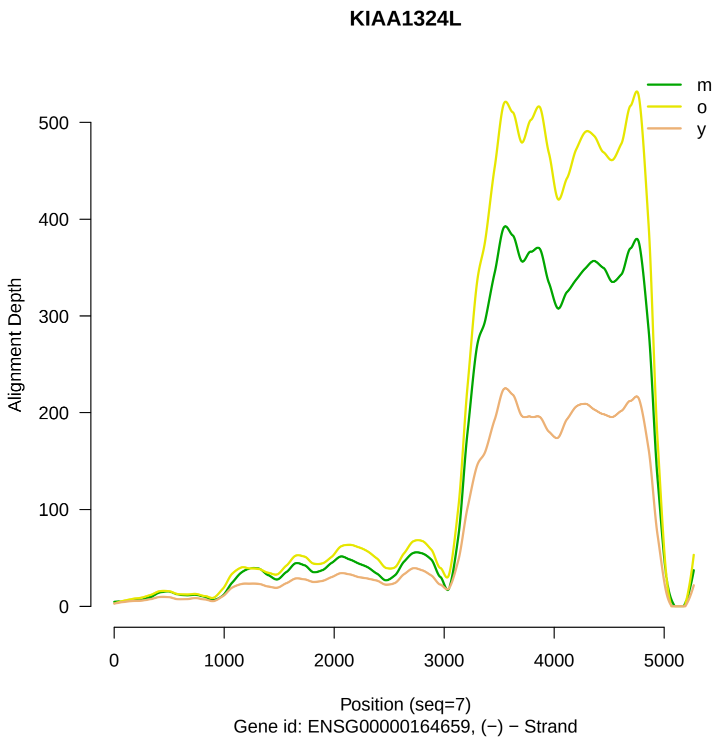

Figure 89: edgeR QLF test based CPM estimates

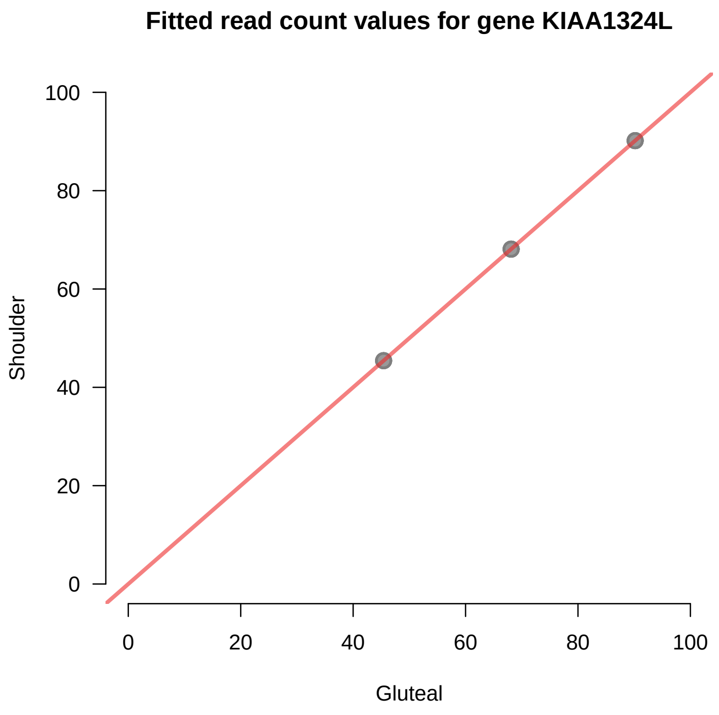

Figure 90: ReadExpSet based genewise CPM estimates

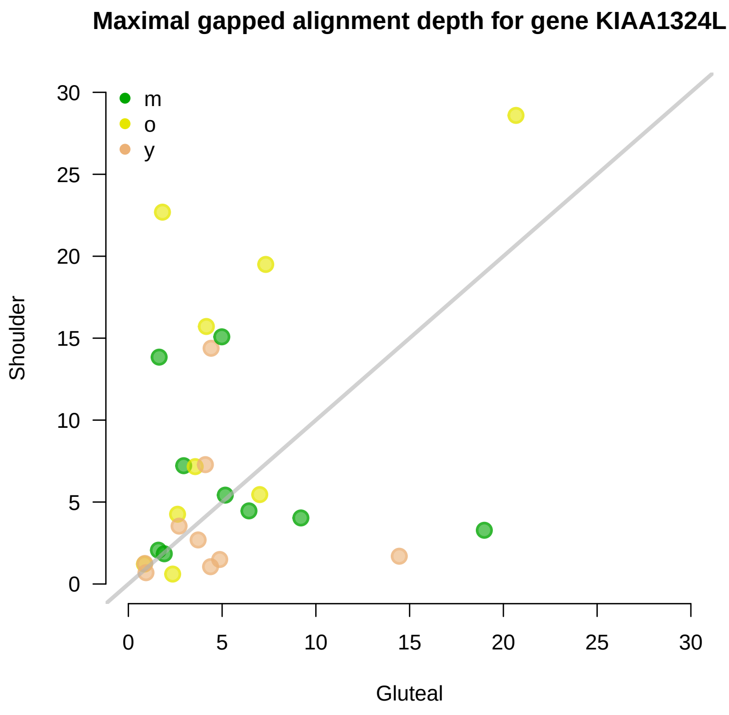

3.16 TRNP1

Parameter	Value
gene_name	TRNP1
gene_id	ENSG00000253368
maxald	3613
old	down
seqid	1
strand	+
start	26993707
end	27000898
descr	TMF1-regulated nuclear protein 1

Table 17: Gene identifcation

Gene expression estimates for Age

Influence of UV exposition on gene expression

Figure 91: Gene expression estimates based on CPM (SummarizeOveraps)

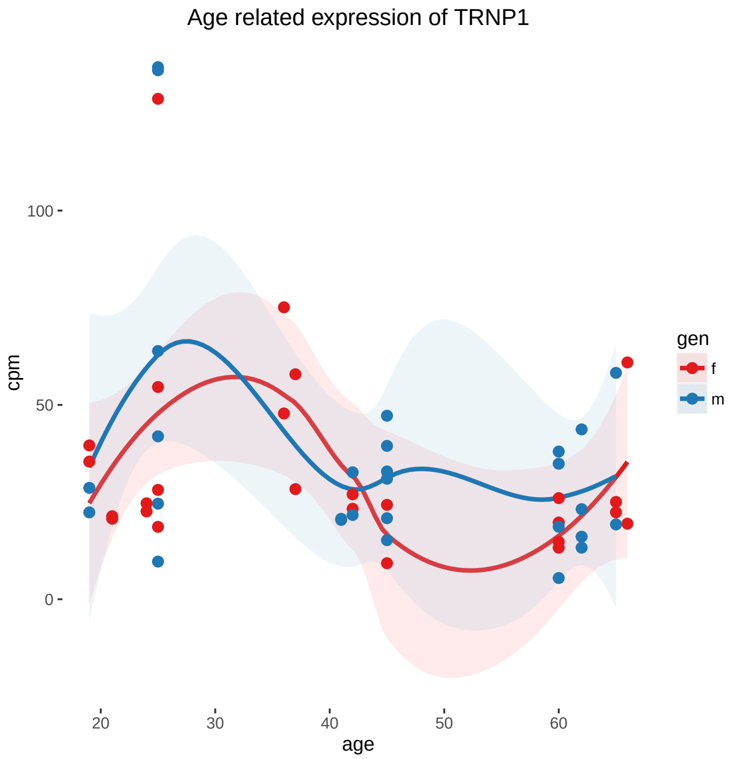

Figure 92: edgeR QLF test based CPM estimates

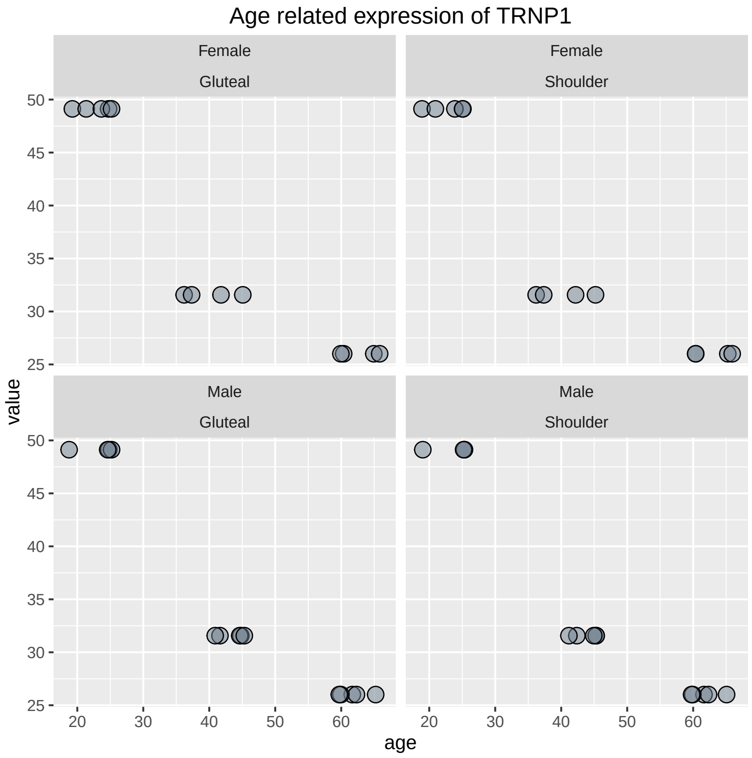

Figure 93: ReadExpSet based genewise CPM estimates

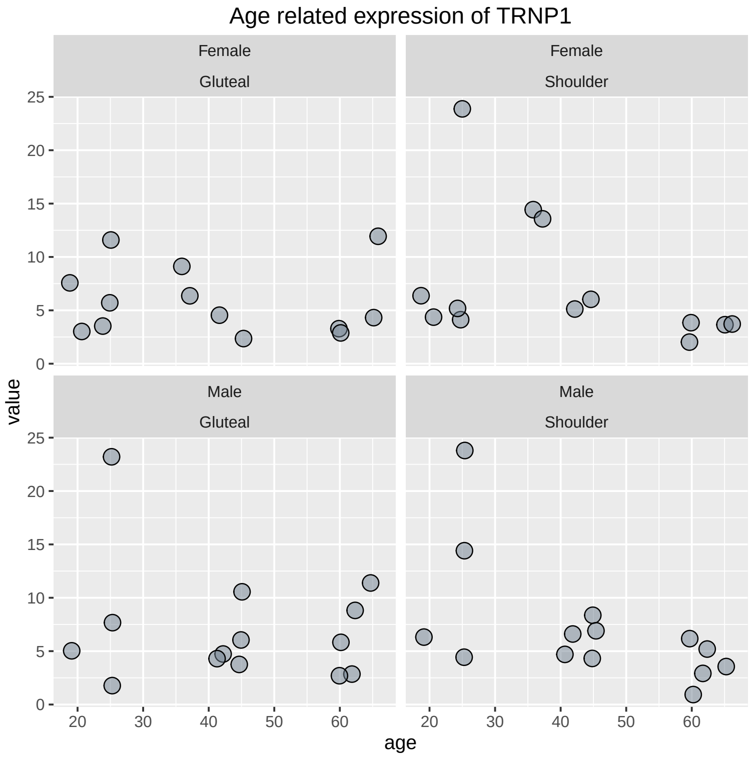

Figure 94: Loess regression for exon align depth

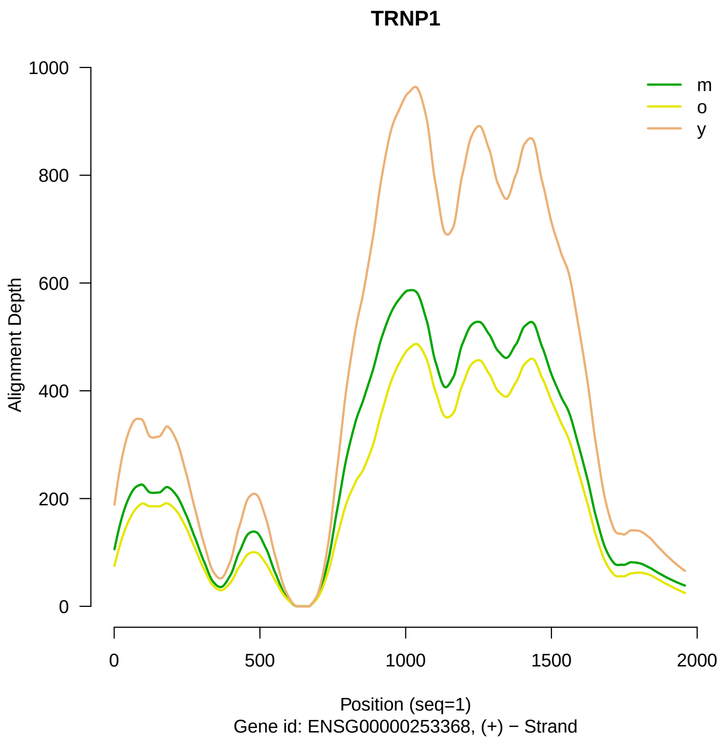

Figure 95: edgeR QLF test based CPM estimates

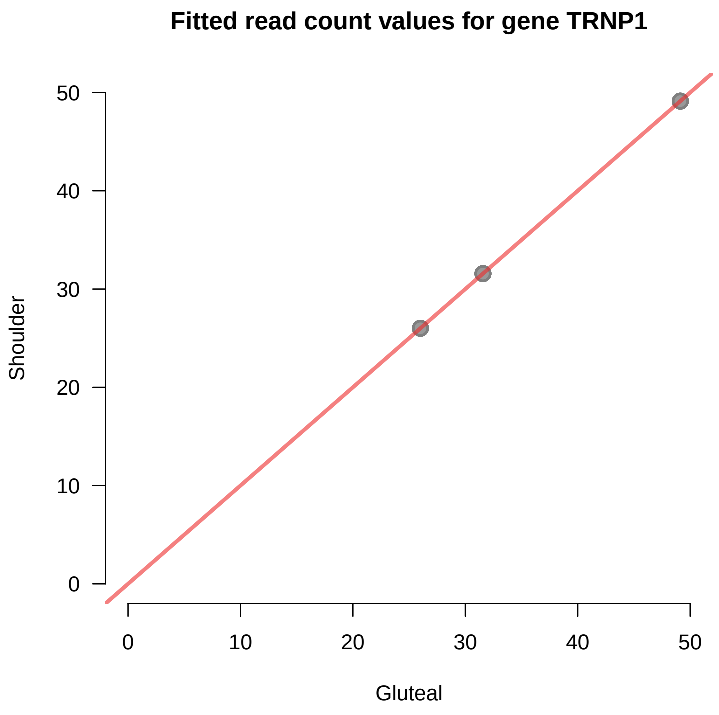

Figure 96: ReadExpSet based genewise CPM estimates

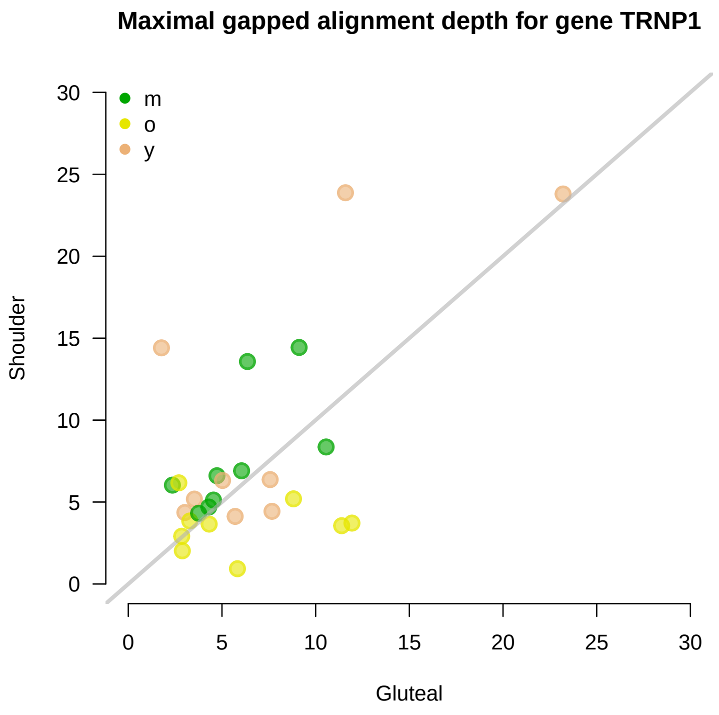

3.17 HSPB7

Parameter	Value
gene_name	HSPB7
gene_id	ENSG00000173641
maxald	14954
old	down
seqid	1
strand	-
start	16014028
end	16019594
descr	heat shock 27kDa protein family, member 7 (cardiovascular)

Table 18: Gene identifcation

Gene expression estimates for Age

Influence of UV exposition on gene expression

Figure 97: Gene expression estimates based on CPM (SummarizeOveraps)

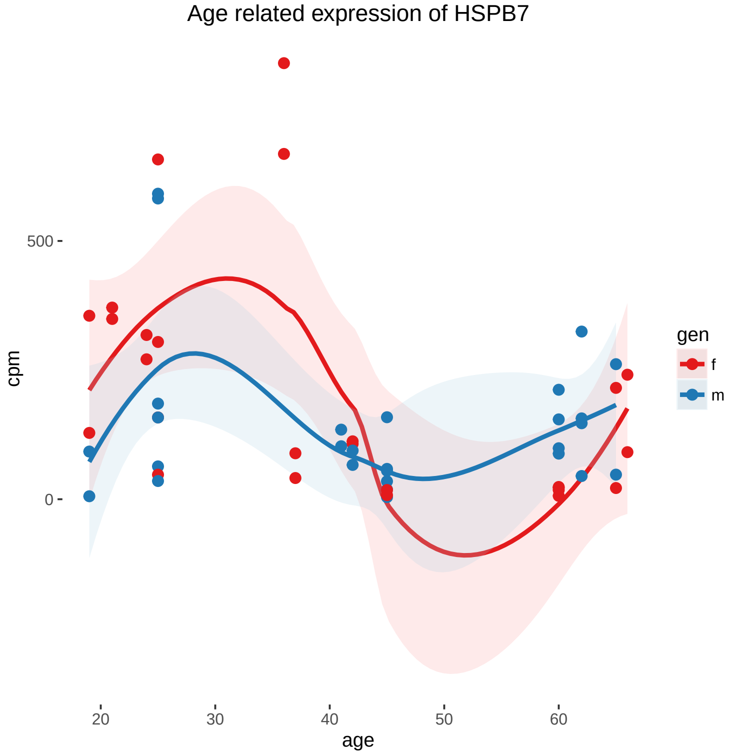

Figure 98: edgeR QLF test based CPM estimates

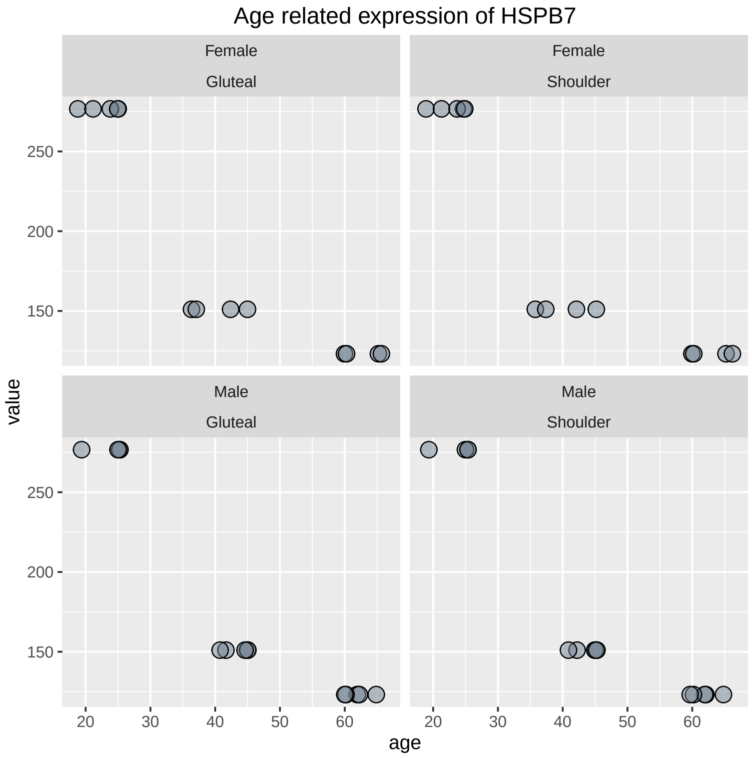

Figure 99: ReadExpSet based genewise CPM estimates

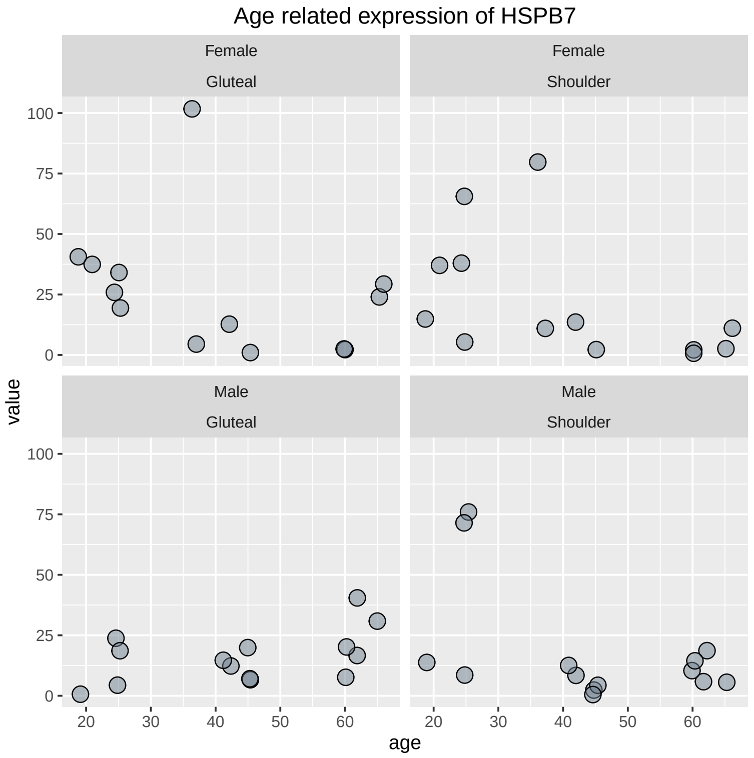

Figure 100: Loess regression for exon align depth

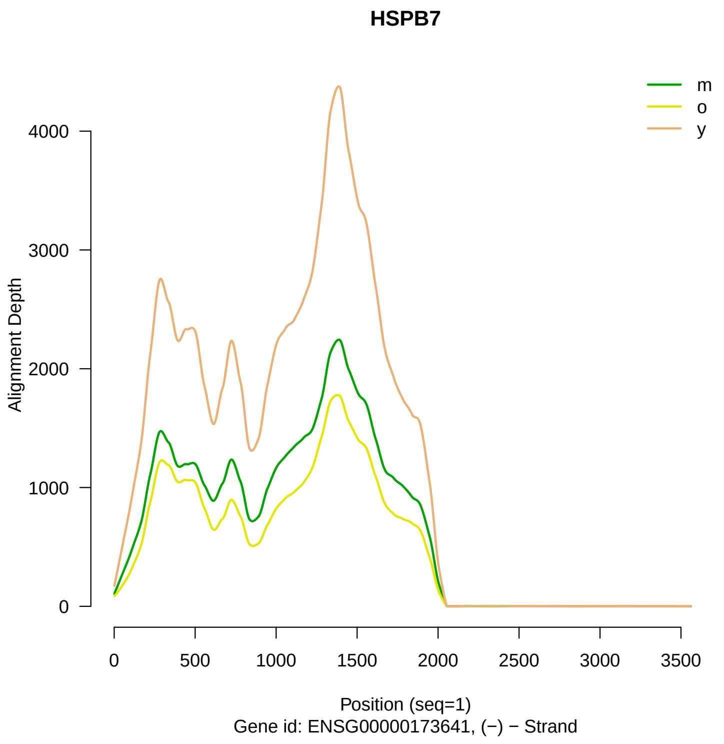

Figure 101: edgeR QLF test based CPM estimates

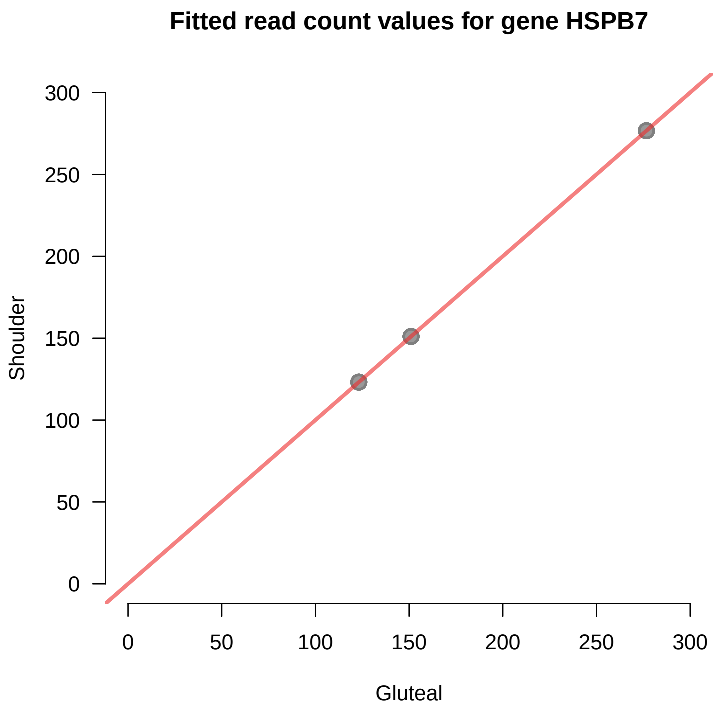

Figure 102: ReadExpSet based genewise CPM estimates

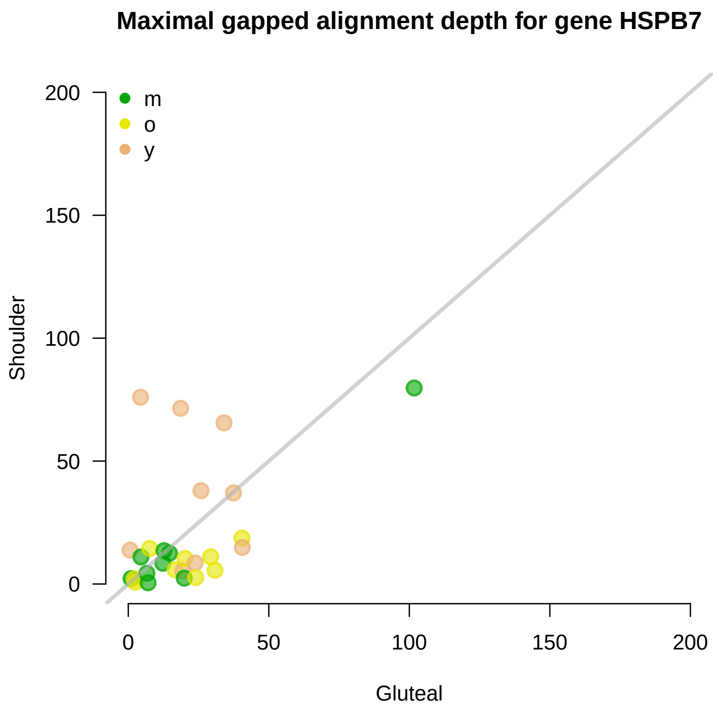

3.18 PRRX2

Parameter	Value
gene_name	PRRX2
gene_id	ENSG00000167157
maxald	6616
old	down
seqid	9
strand	+
start	129665641
end	129722674
descr	paired related homeobox 2

Table 19: Gene identifcation

Gene expression estimates for Age

Influence of UV exposition on gene expression

Figure 103: Gene expression estimates based on CPM (SummarizeOveraps)

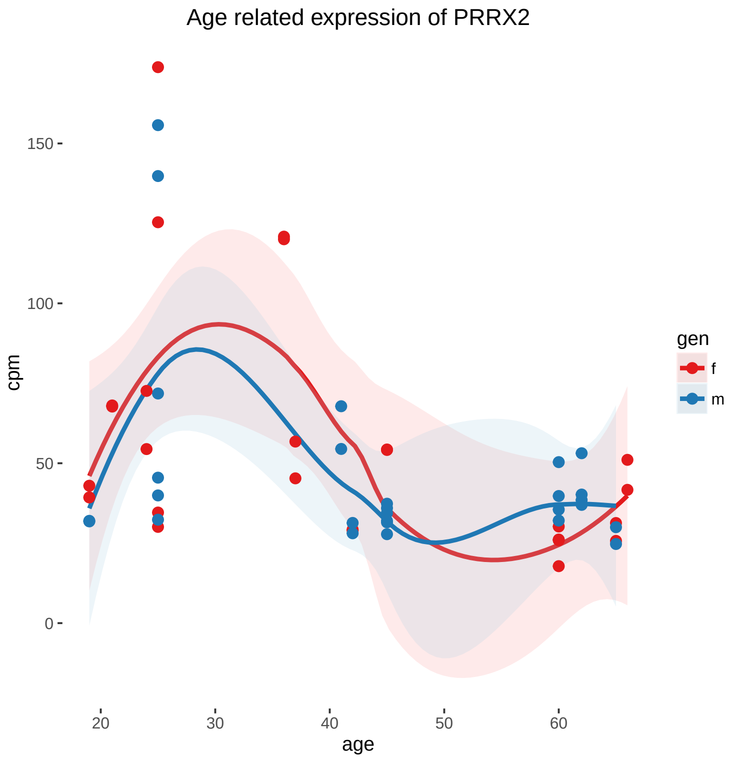

Figure 104: edgeR QLF test based CPM estimates

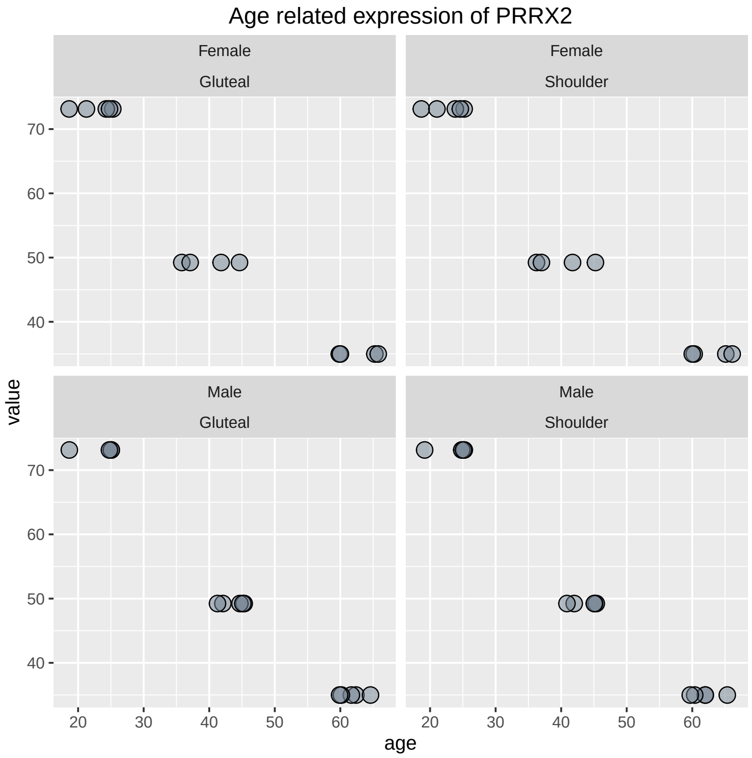

Figure 105: ReadExpSet based genewise CPM estimates

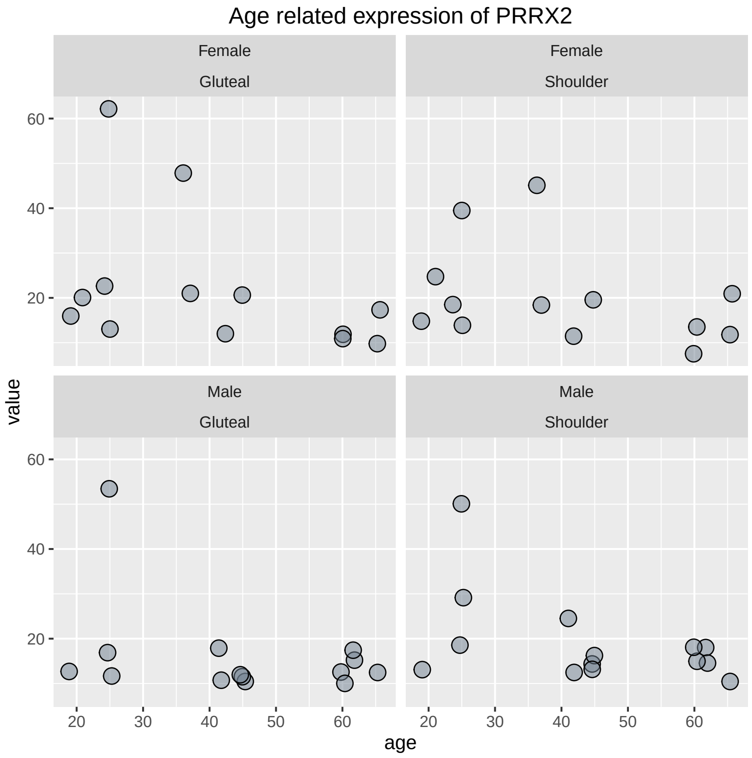

Figure 106: Loess regression for exon align depth

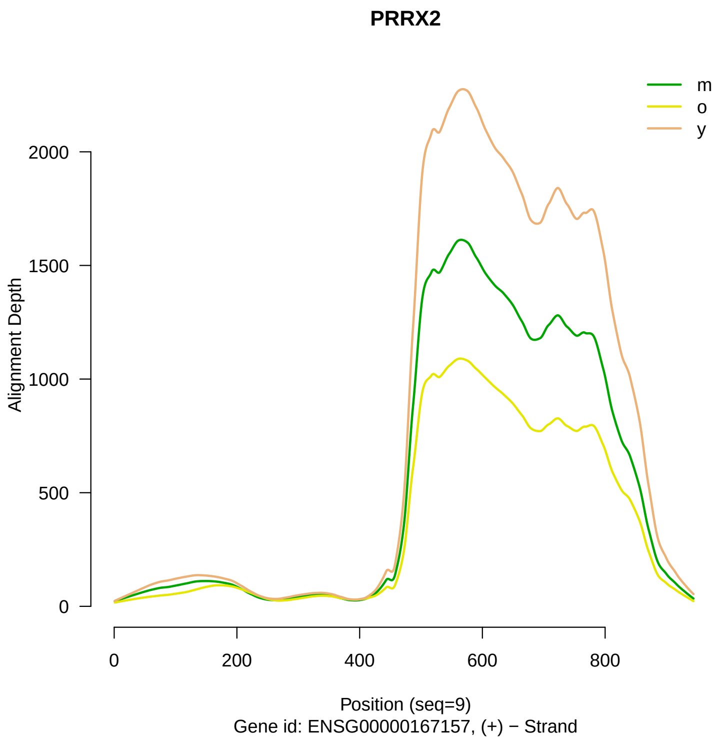

Figure 107: edgeR QLF test based CPM estimates

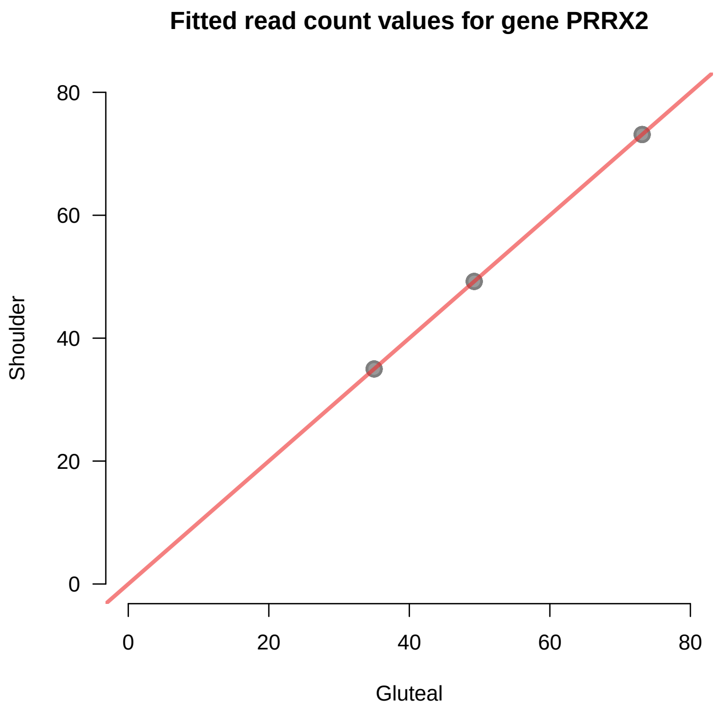

Figure 108: ReadExpSet based genewise CPM estimates

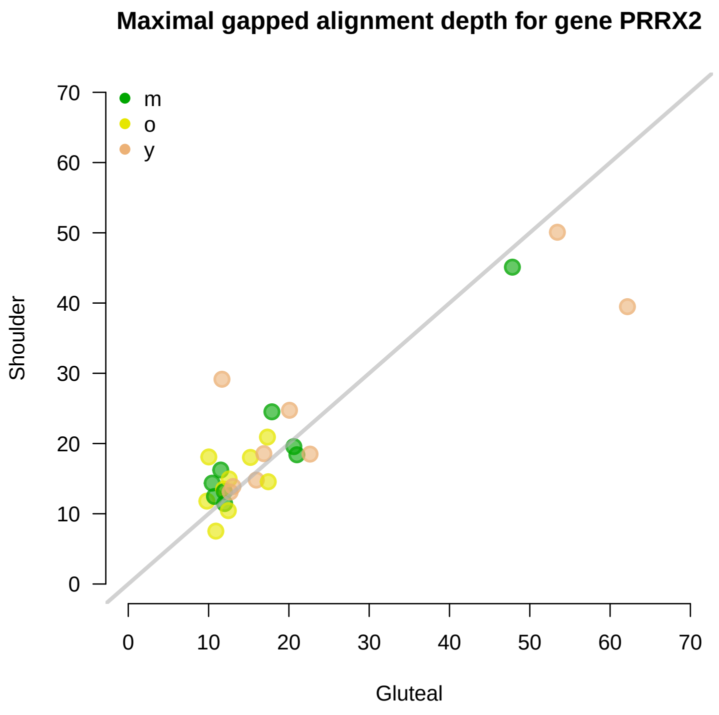

3.19 SMAD7

Parameter	Value
gene_name	SMAD7
gene_id	ENSG00000101665
maxald	3768
old	down
seqid	18
strand	-
start	48919853
end	48950711
descr	SMAD family member 7

Table 20: Gene identifcation

Gene expression estimates for Age

Influence of UV exposition on gene expression

Figure 109: Gene expression estimates based on CPM (SummarizeOveraps)

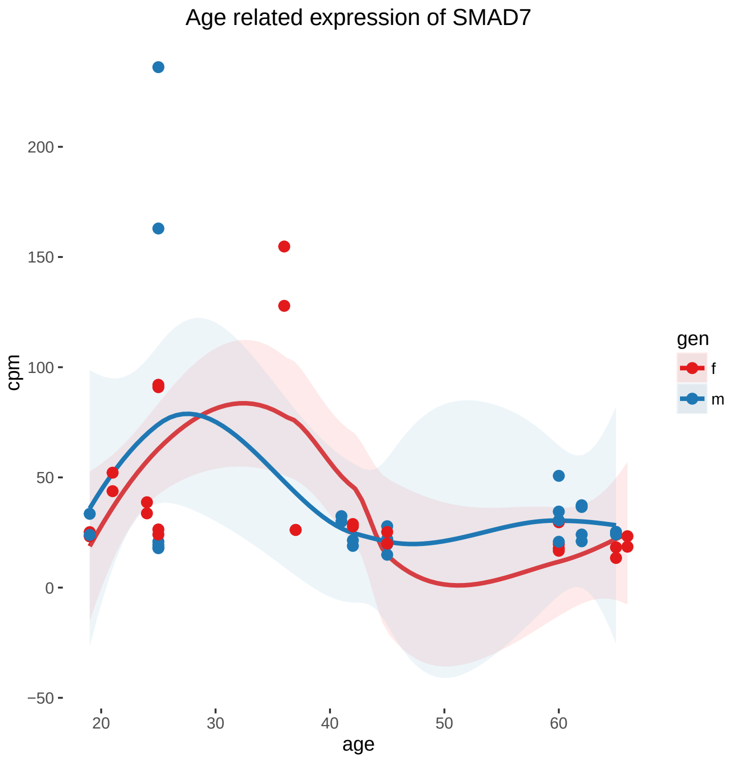

Figure 110: edgeR QLF test based CPM estimates

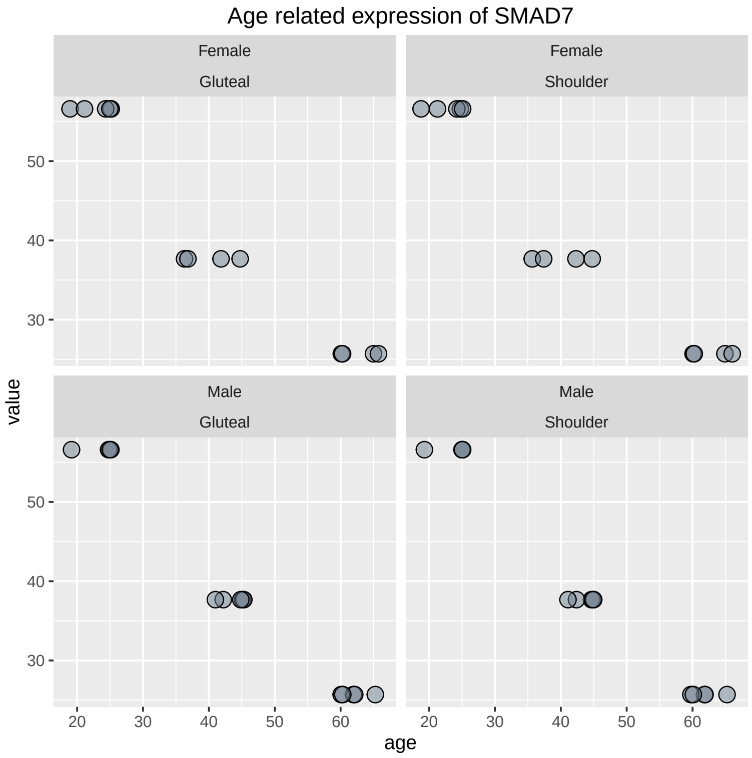

Figure 111: ReadExpSet based genewise CPM estimates

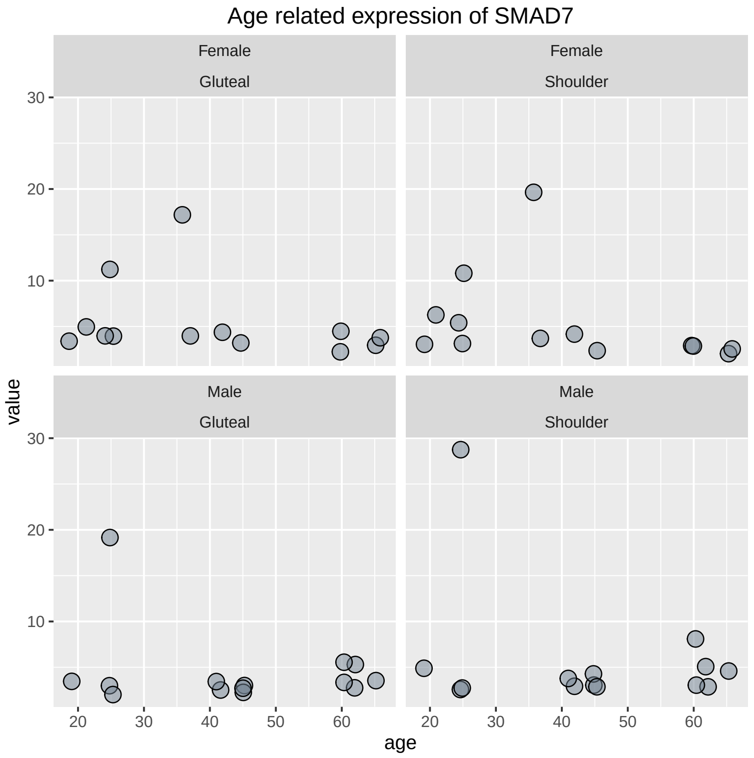

Figure 112: Loess regression for exon align depth

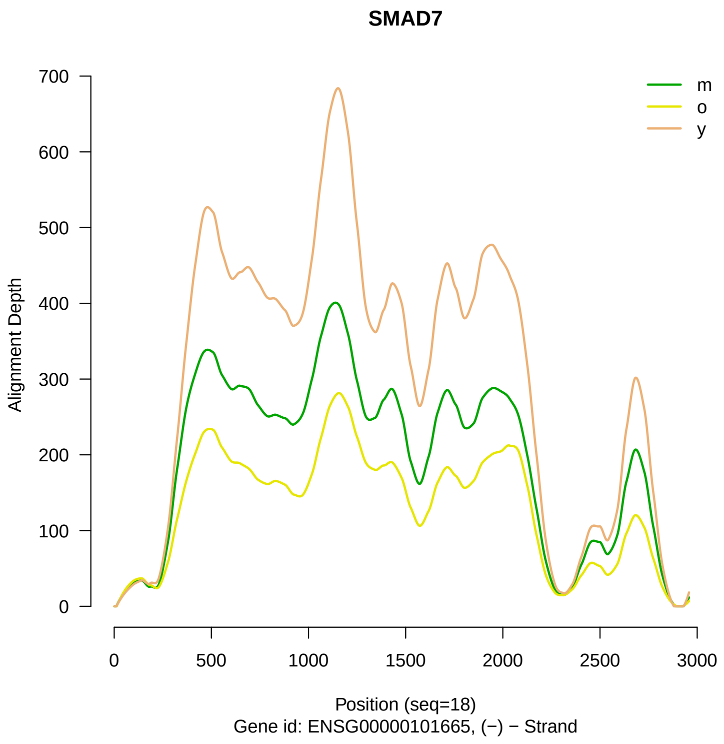

Figure 113: edgeR QLF test based CPM estimates

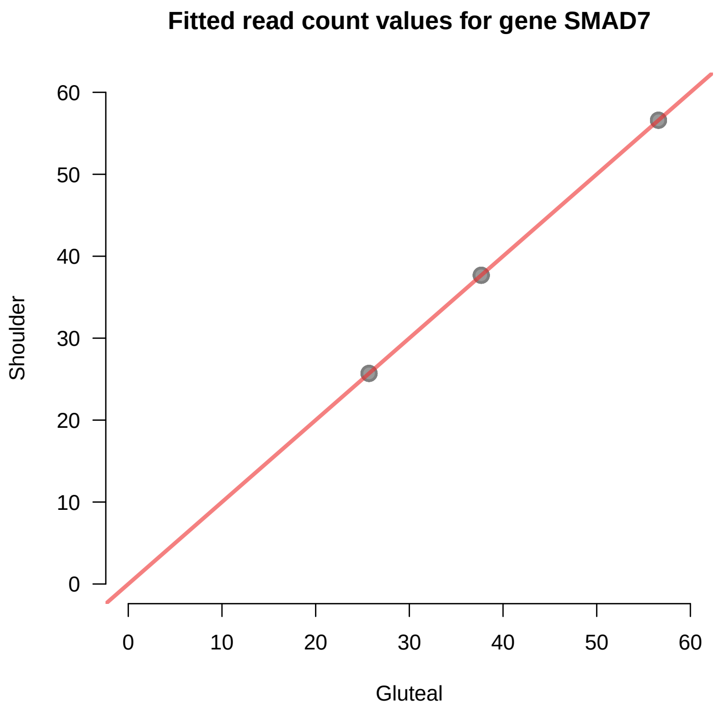

Figure 114: ReadExpSet based genewise CPM estimates

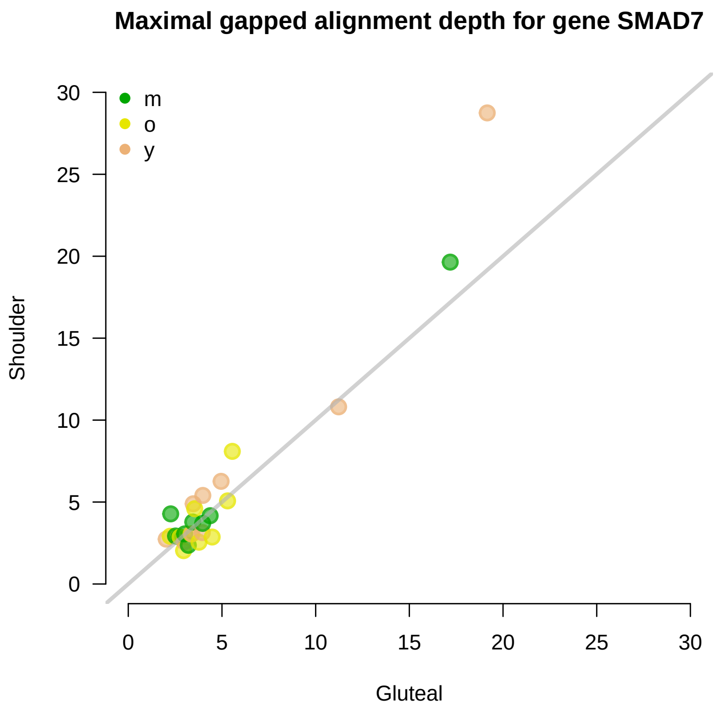

3.20 FAM83G

Parameter	Value
gene_name	FAM83G
gene_id	ENSG00000188522
maxald	523
old	down
seqid	17
strand	-
start	18968789
end	19004804
descr	family with sequence similarity 83, member G

Table 21: Gene identifcation

Gene expression estimates for Age

Influence of UV exposition on gene expression

Figure 115: Gene expression estimates based on CPM (SummarizeOveraps)

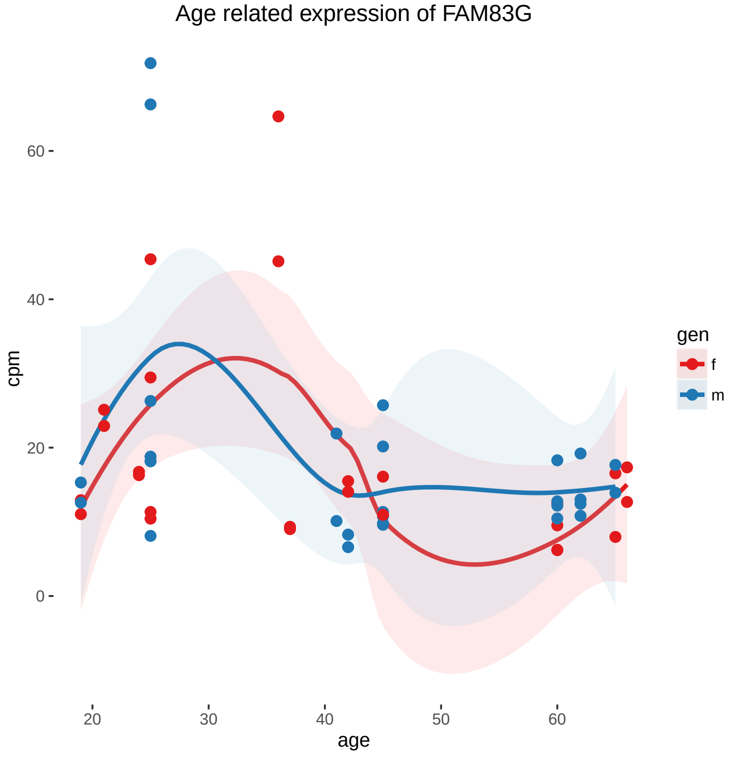

Figure 116: edgeR QLF test based CPM estimates

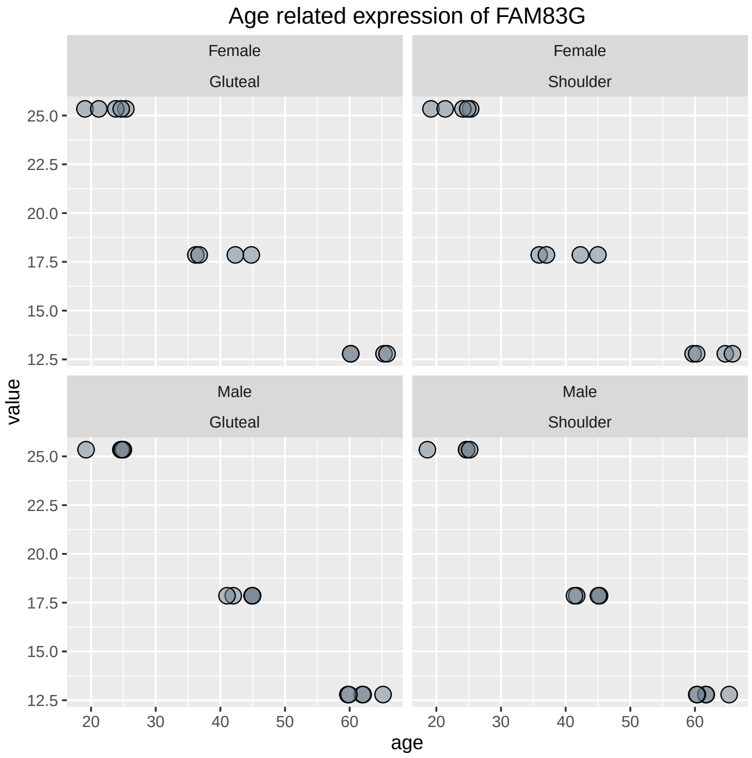

Figure 117: ReadExpSet based genewise CPM estimates

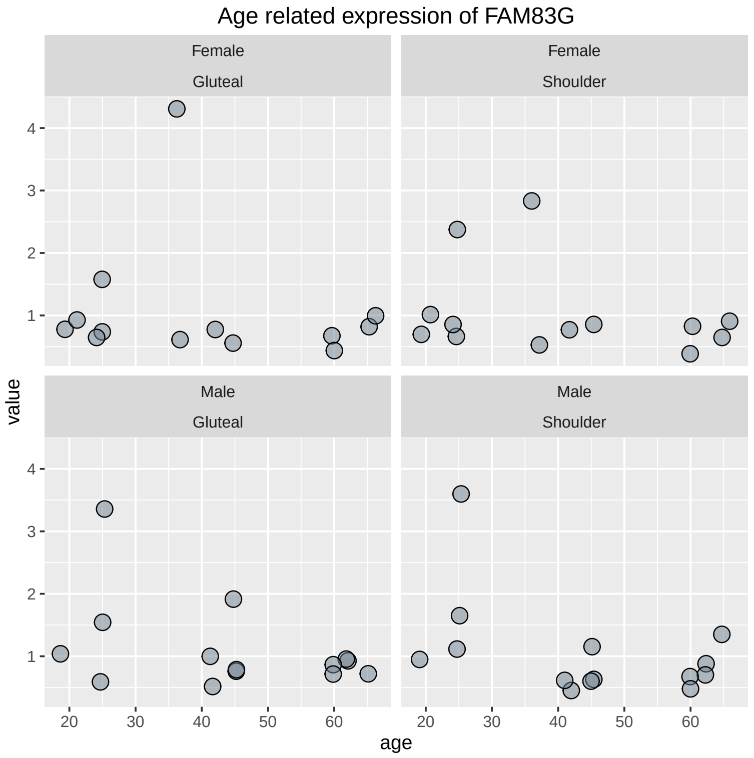

Figure 118: Loess regression for exon align depth

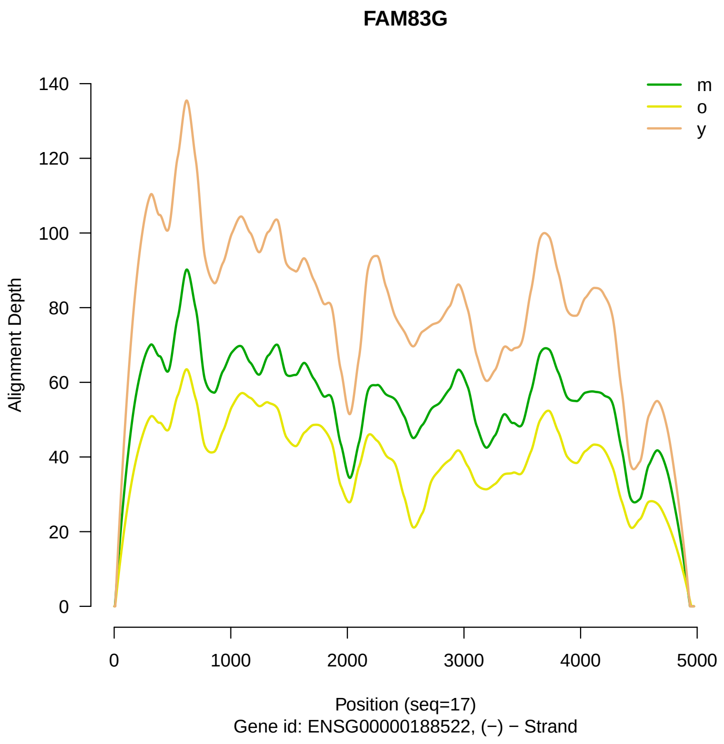

Figure 119: edgeR QLF test based CPM estimates

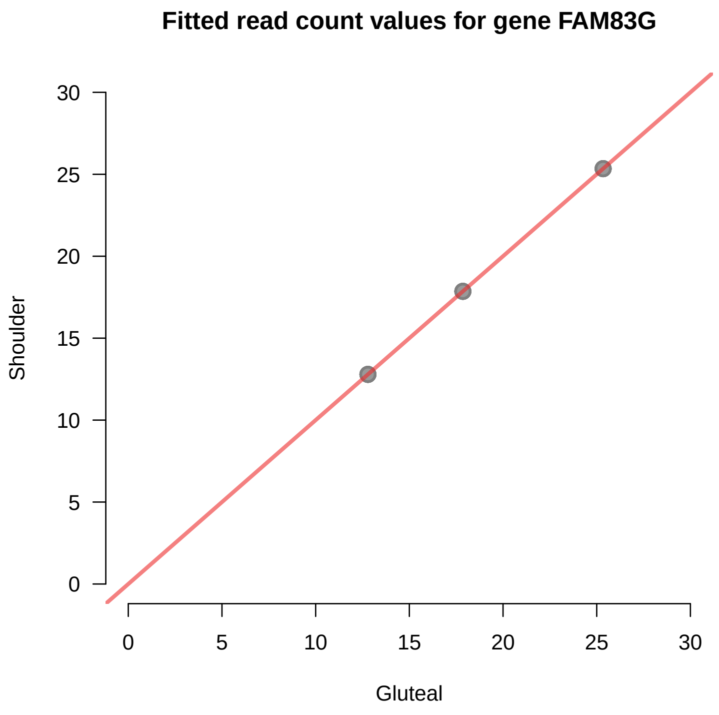

Figure 120: ReadExpSet based genewise CPM estimates

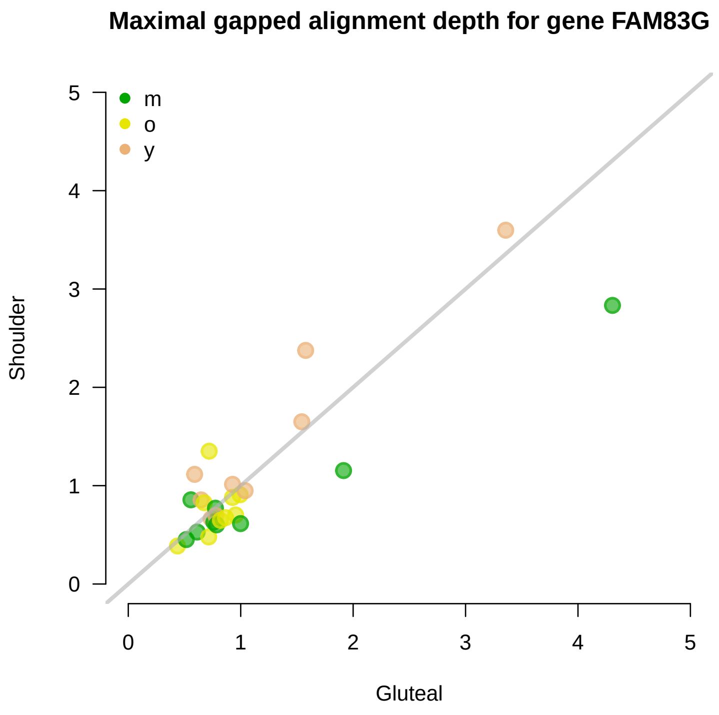

3.21 DDR1

Parameter	Value
gene_name	DDR1
gene_id	ENSG00000204580
maxald	606
old	down
seqid	6
strand	+
start	30876421
end	30900156
descr	discoidin domain receptor tyrosine kinase 1

Table 22: Gene identifcation

Gene expression estimates for Age

Influence of UV exposition on gene expression

Figure 121: Gene expression estimates based on CPM (SummarizeOveraps)

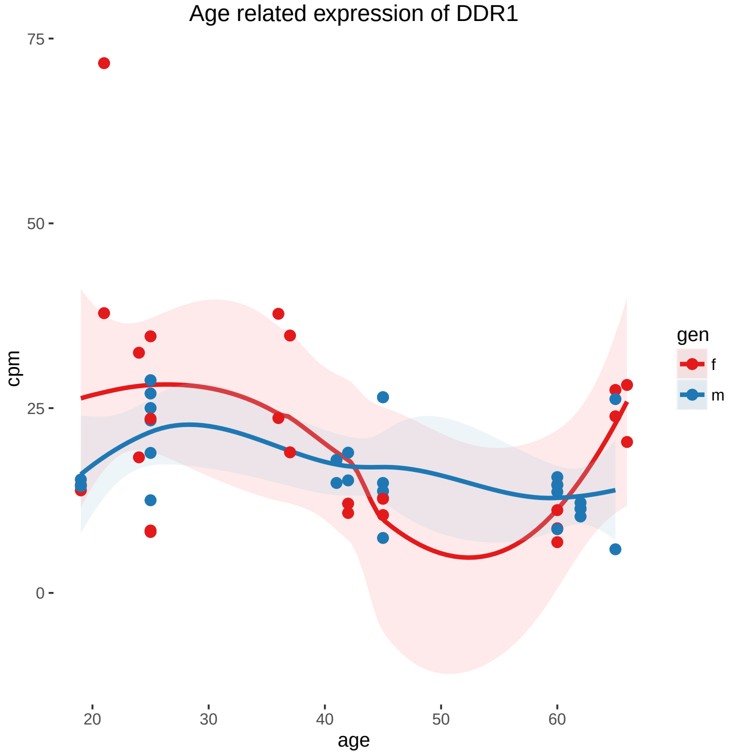

Figure 122: edgeR QLF test based CPM estimates

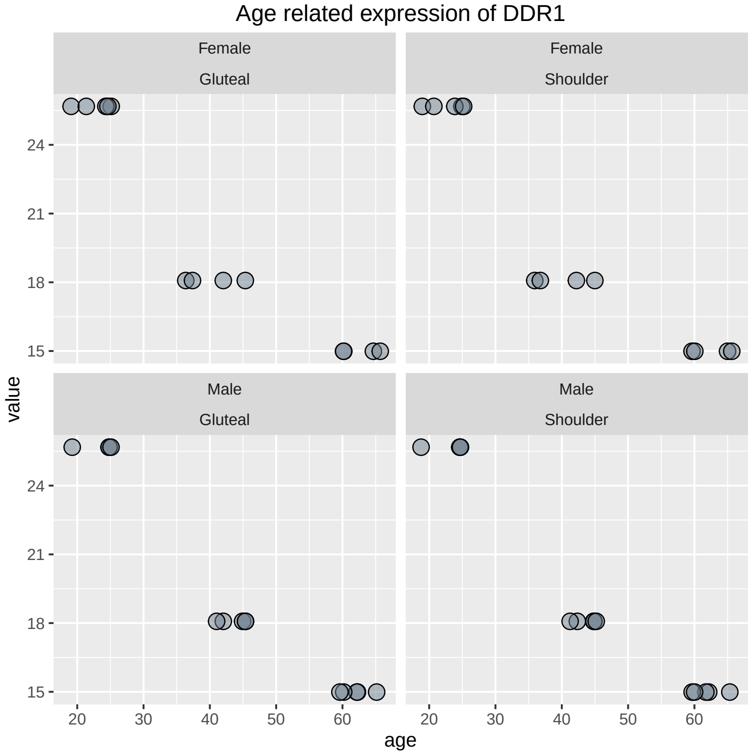

Figure 123: ReadExpSet based genewise CPM estimates

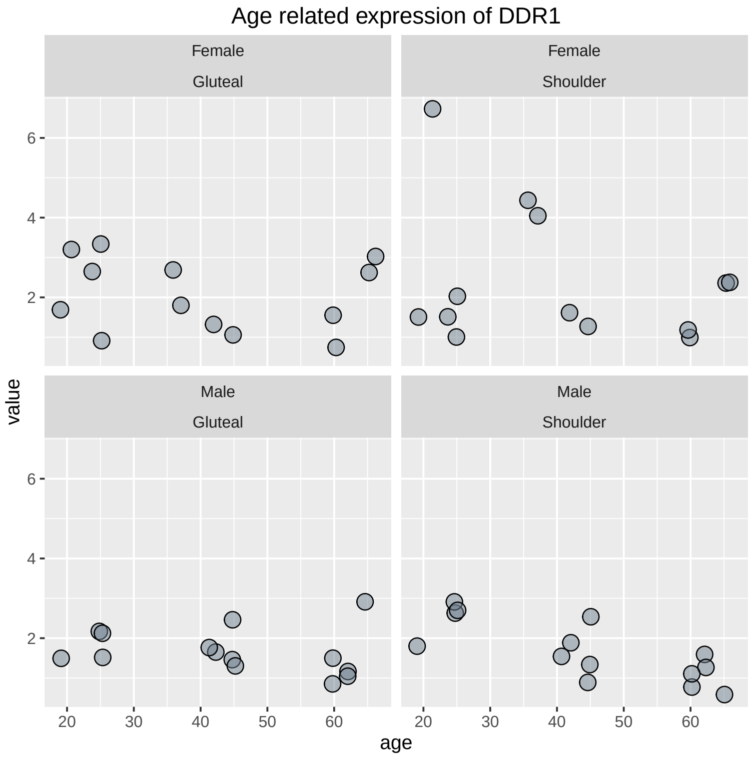

Figure 124: Loess regression for exon align depth

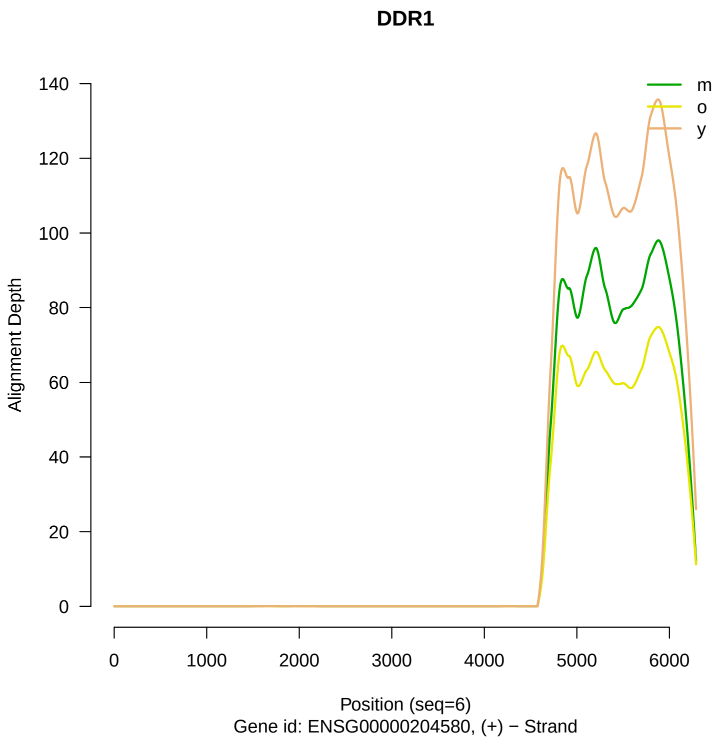

Figure 125: edgeR QLF test based CPM estimates

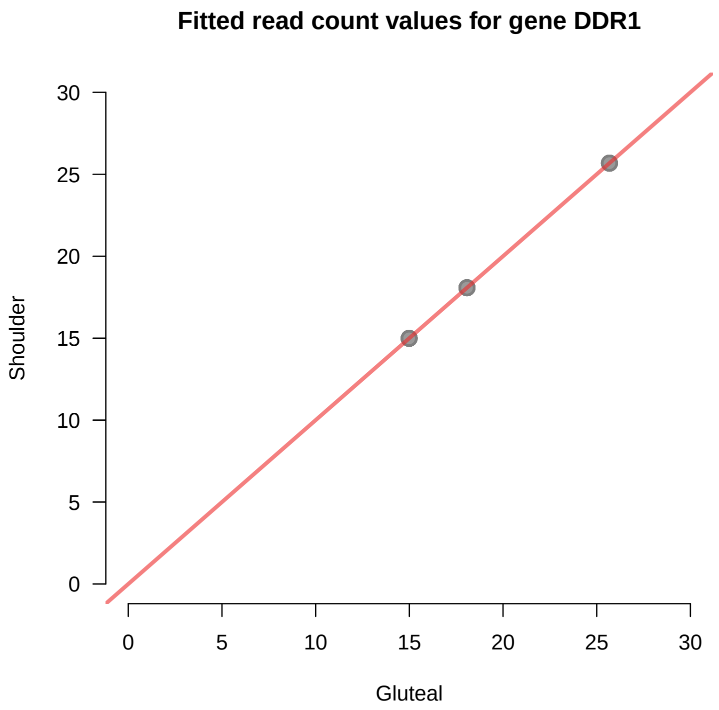

Figure 126: ReadExpSet based genewise CPM estimates

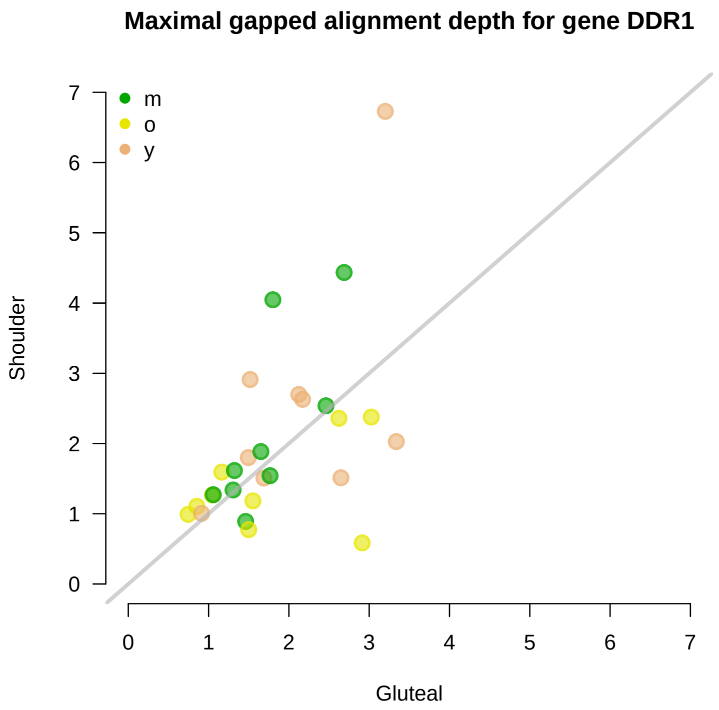

3.22 PPP1R3C

Parameter	Value
gene_name	PPP1R3C
gene_id	ENSG00000119938
maxald	1889
old	down
seqid	10
strand	-
start	91628442
end	91633054
descr	protein phosphatase 1, regulatory subunit 3C

Table 23: Gene identifcation

Gene expression estimates for Age

Influence of UV exposition on gene expression

Figure 127: Gene expression estimates based on CPM (SummarizeOveraps)

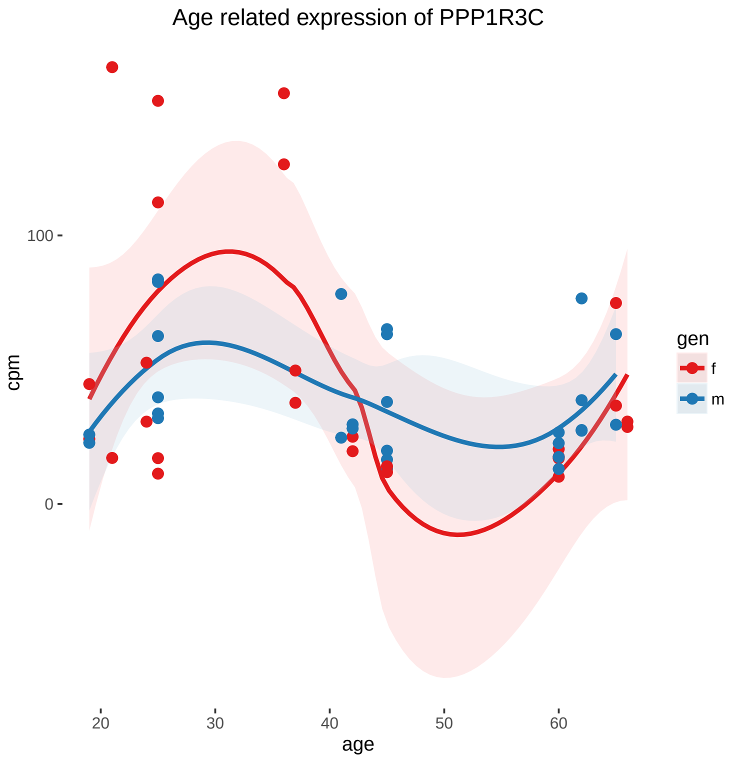

Figure 128: edgeR QLF test based CPM estimates

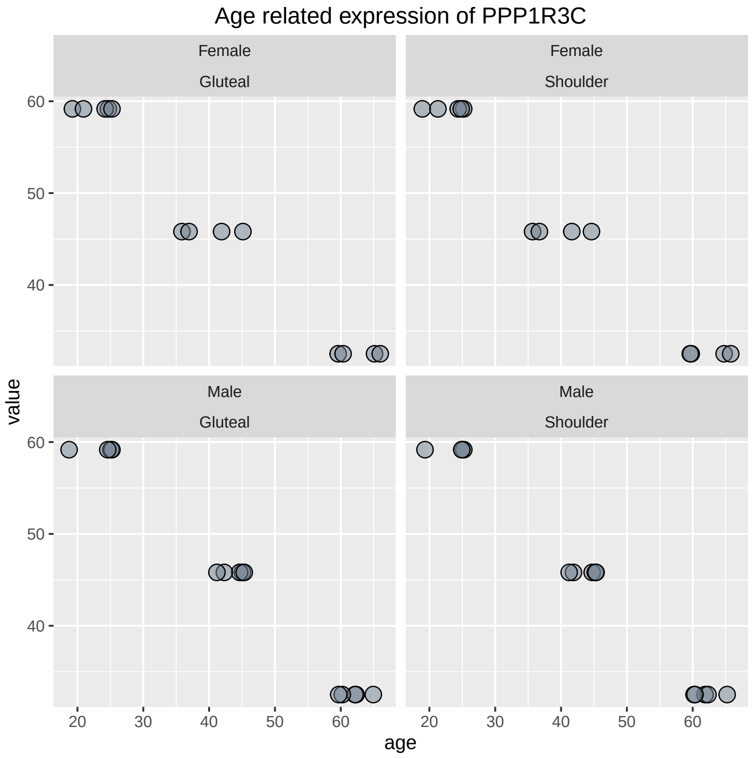

Figure 129: ReadExpSet based genewise CPM estimates

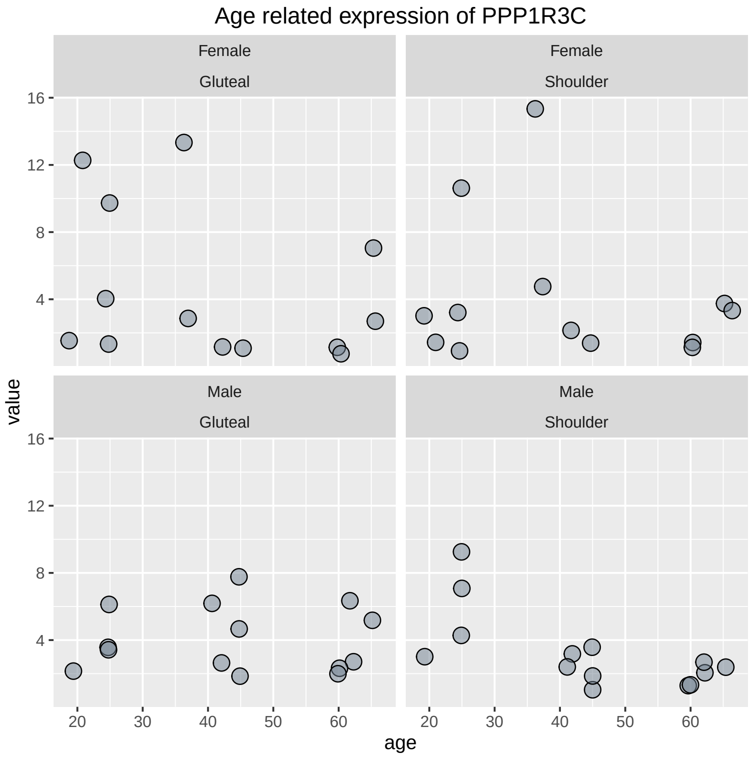

Figure 130: Loess regression for exon align depth

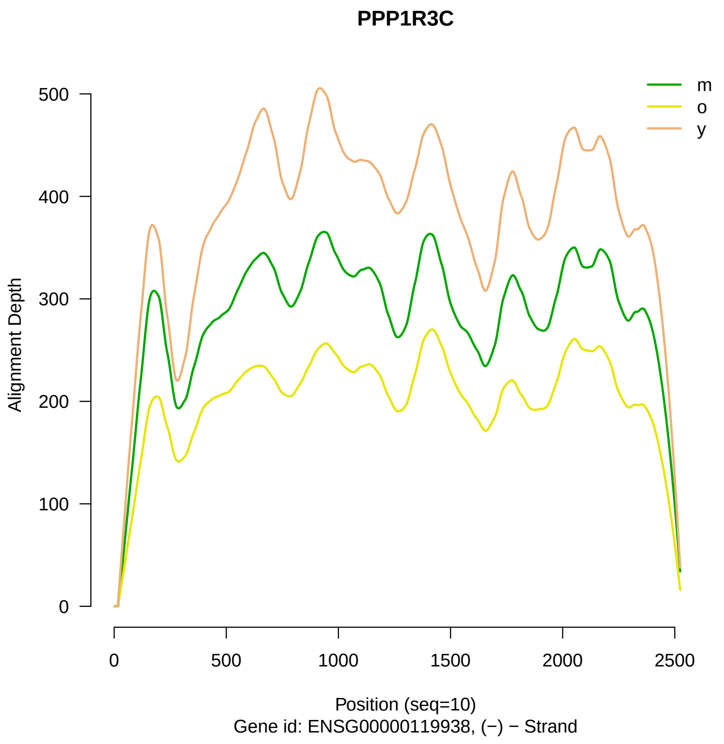

Figure 131: edgeR QLF test based CPM estimates

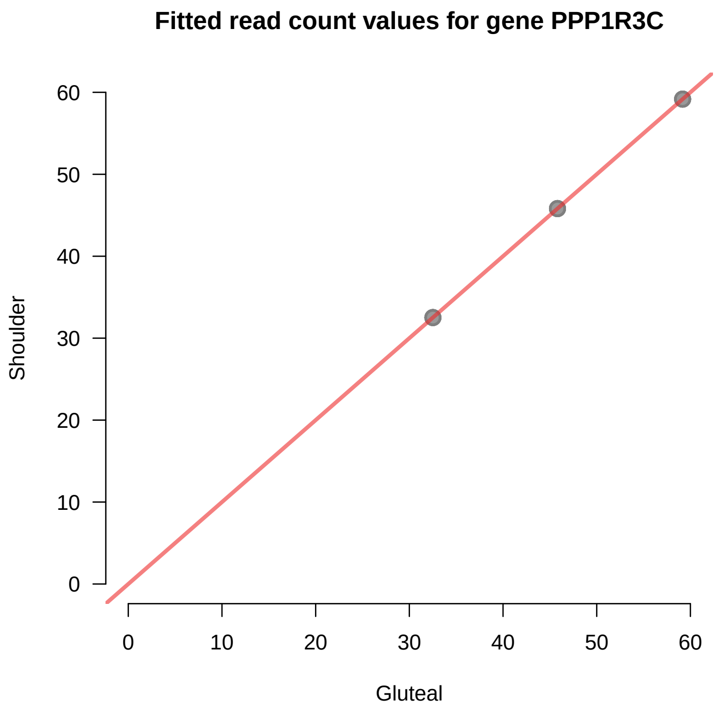

Figure 132: ReadExpSet based genewise CPM estimates

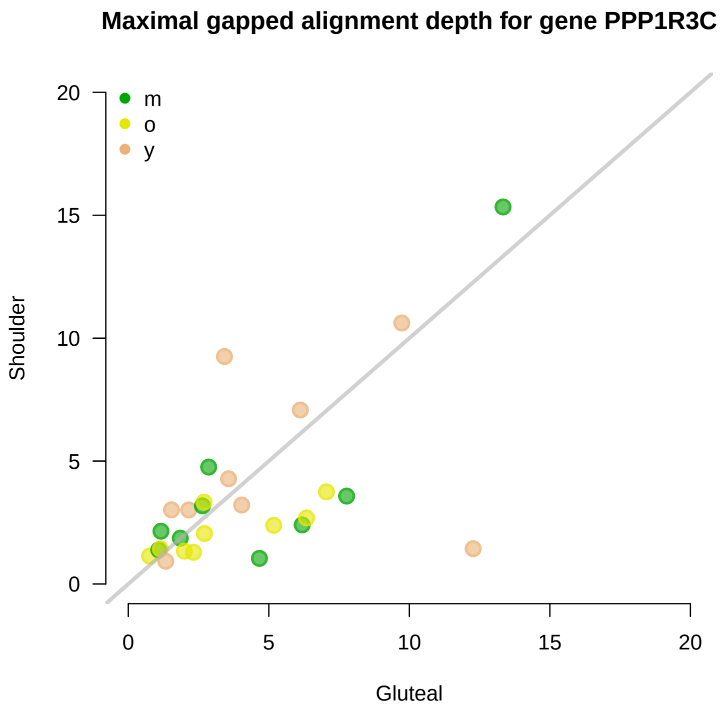

3.23 EVA1A

Parameter	Value
gene_name	EVA1A
gene_id	ENSG00000115363
maxald	862
old	down
seqid	2
strand	-
start	75469302
end	75569722
descr	eva-1 homolog A (C. elegans)

Table 24: Gene identifcation

Gene expression estimates for Age

Influence of UV exposition on gene expression

Figure 133: Gene expression estimates based on CPM (SummarizeOveraps)

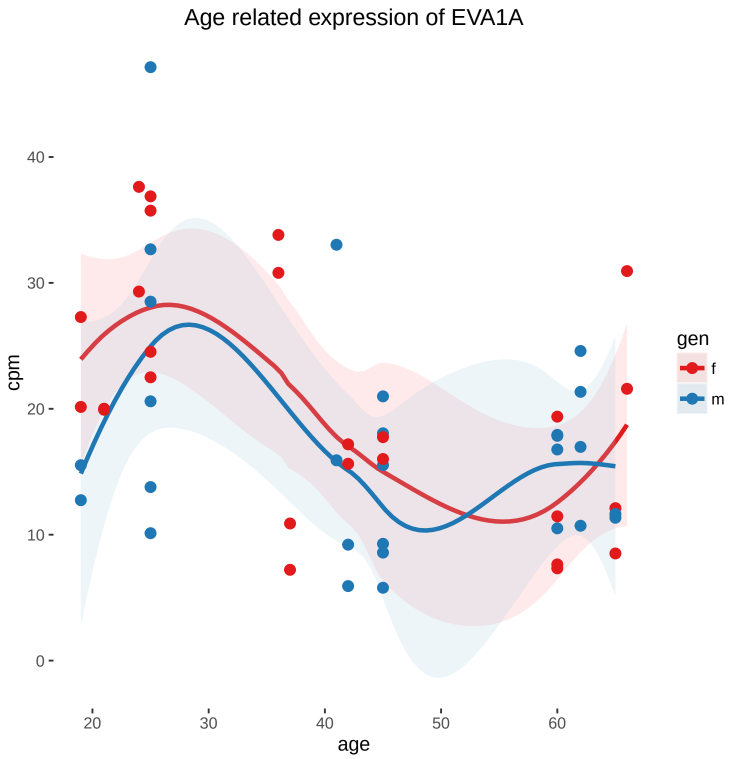

Figure 134: edgeR QLF test based CPM estimates

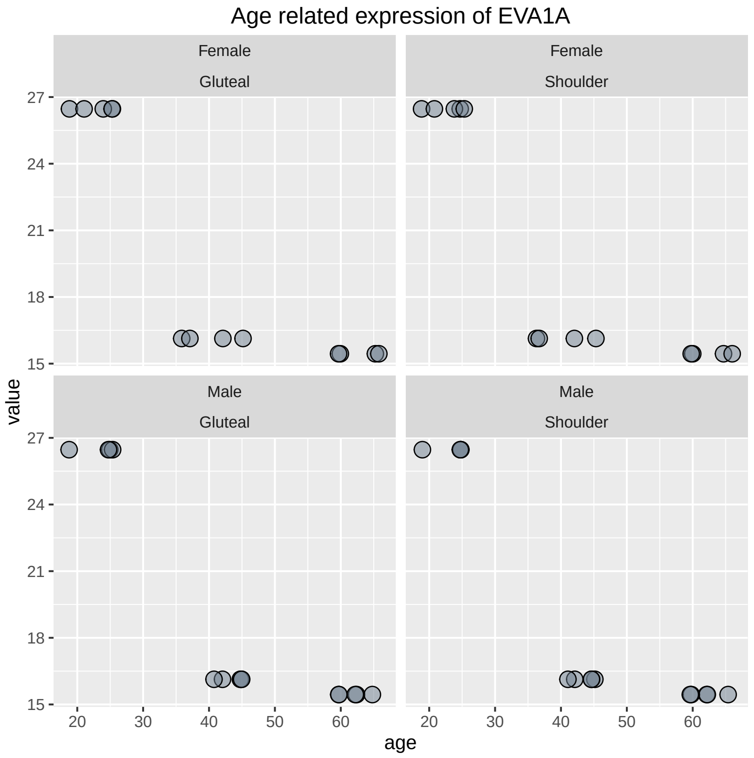

Figure 135: ReadExpSet based genewise CPM estimates

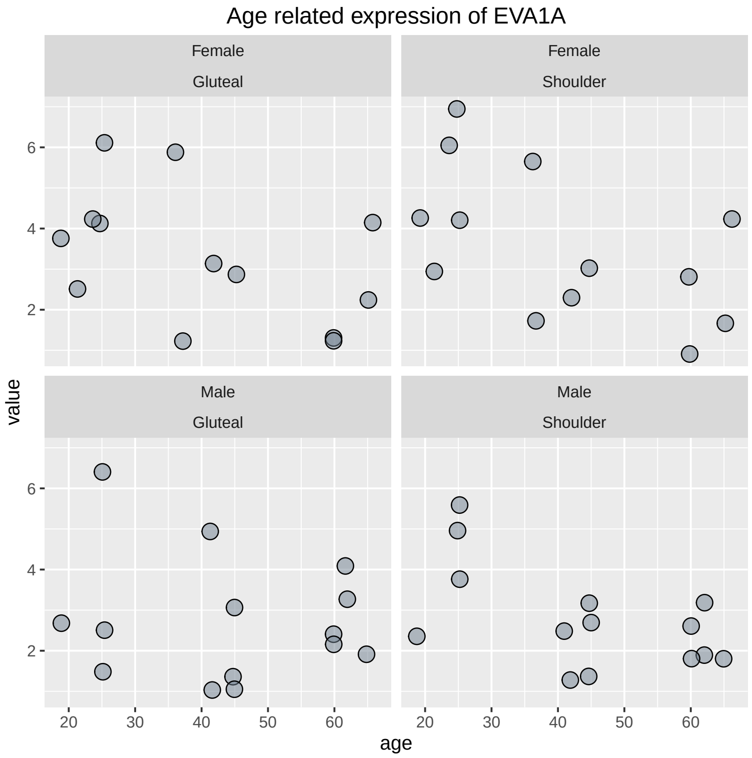

Figure 136: Loess regression for exon align depth

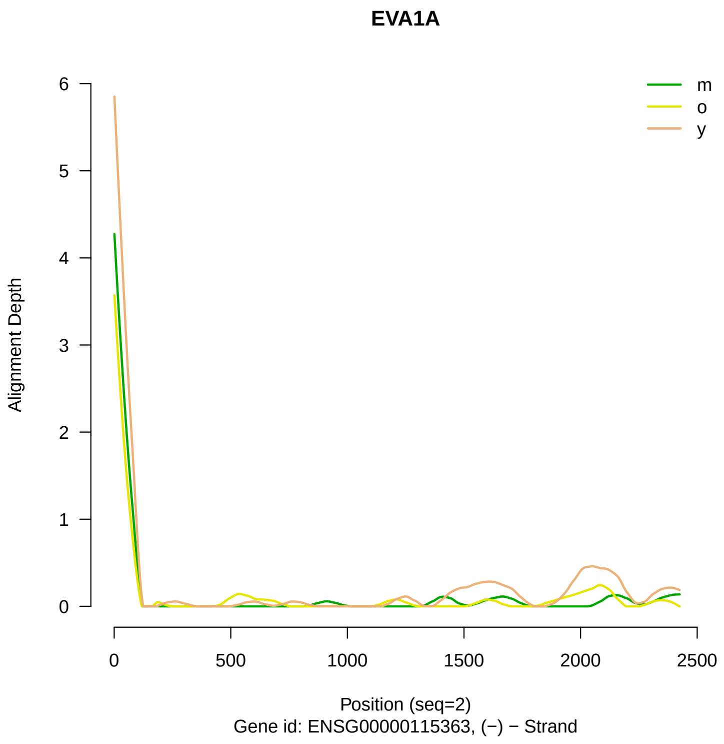

Figure 137: edgeR QLF test based CPM estimates

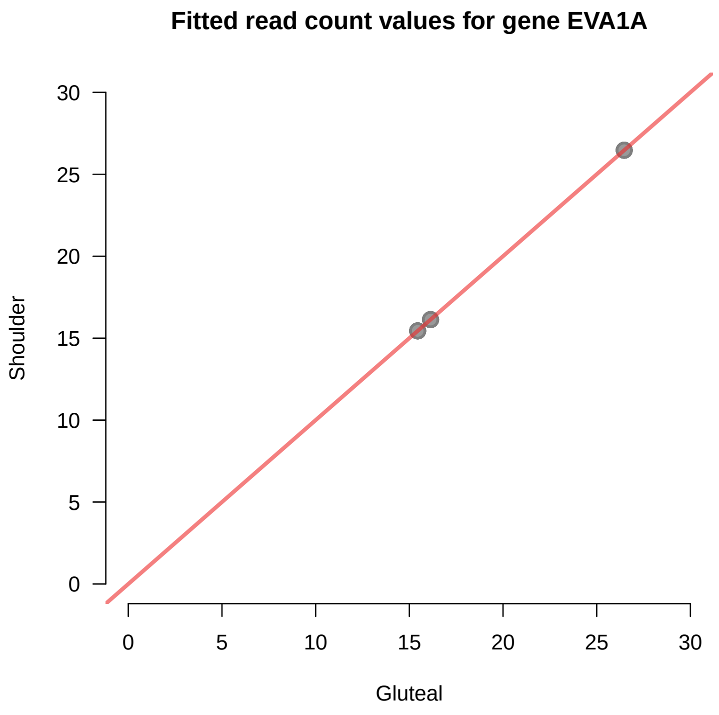

Figure 138: ReadExpSet based genewise CPM estimates

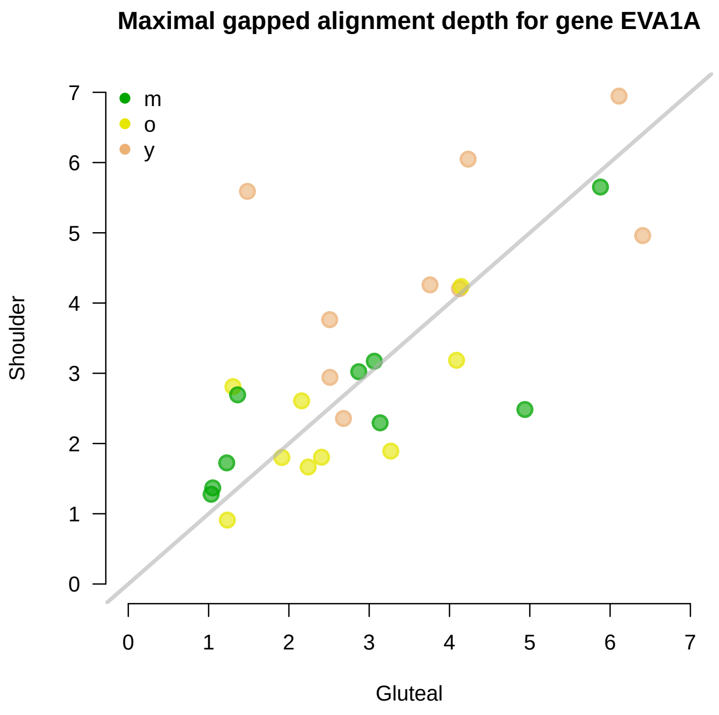

3.24 CRISPLD2

Parameter	Value
gene_name	CRISPLD2
gene_id	ENSG00000103196
maxald	2516
old	down
seqid	16
strand	+
start	84819984
end	84920768
descr	cysteine-rich secretory protein LCCL domain containing 2

Table 25: Gene identifcation

Gene expression estimates for Age

Influence of UV exposition on gene expression

Figure 139: Gene expression estimates based on CPM (SummarizeOveraps)

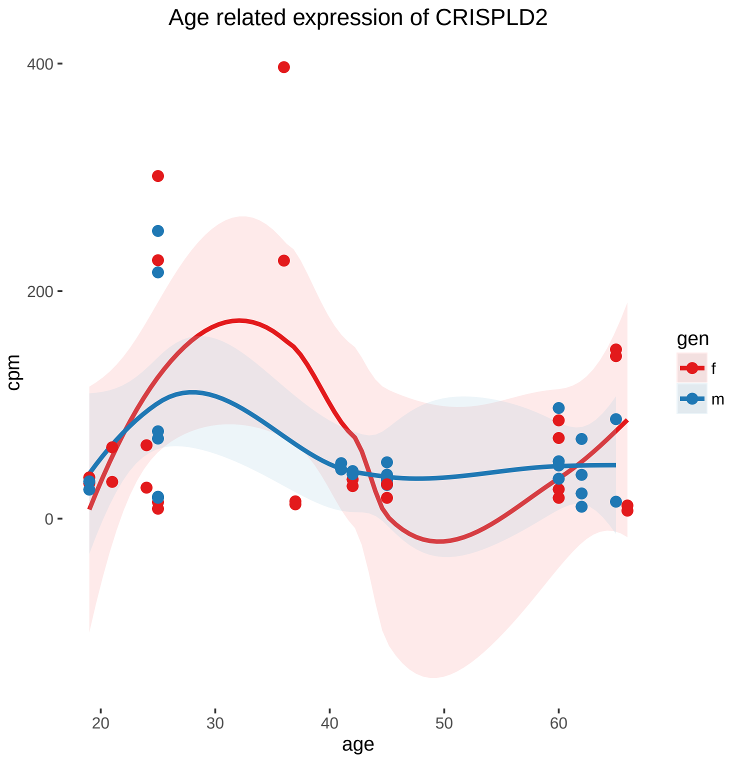

Figure 140: edgeR QLF test based CPM estimates

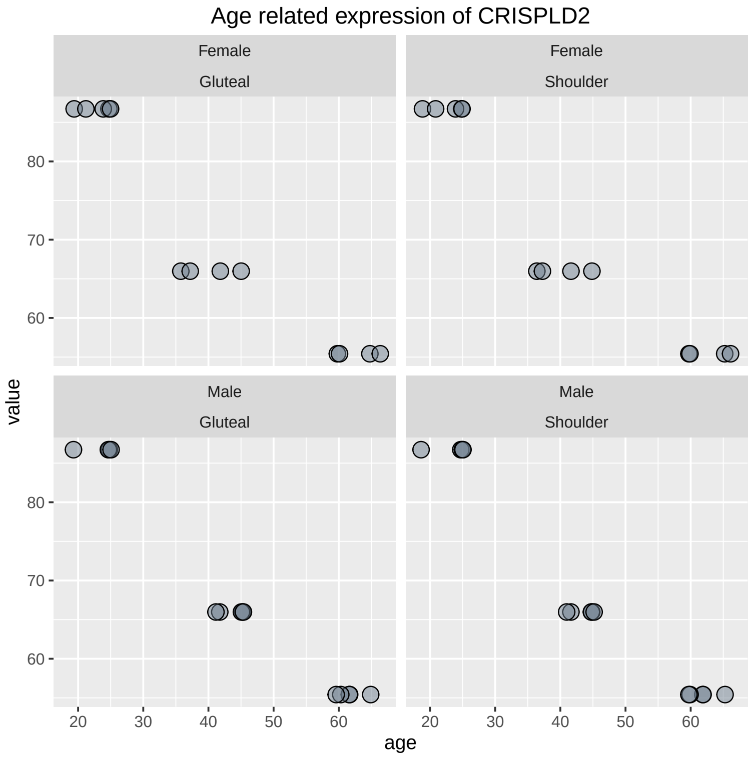

Figure 141: ReadExpSet based genewise CPM estimates

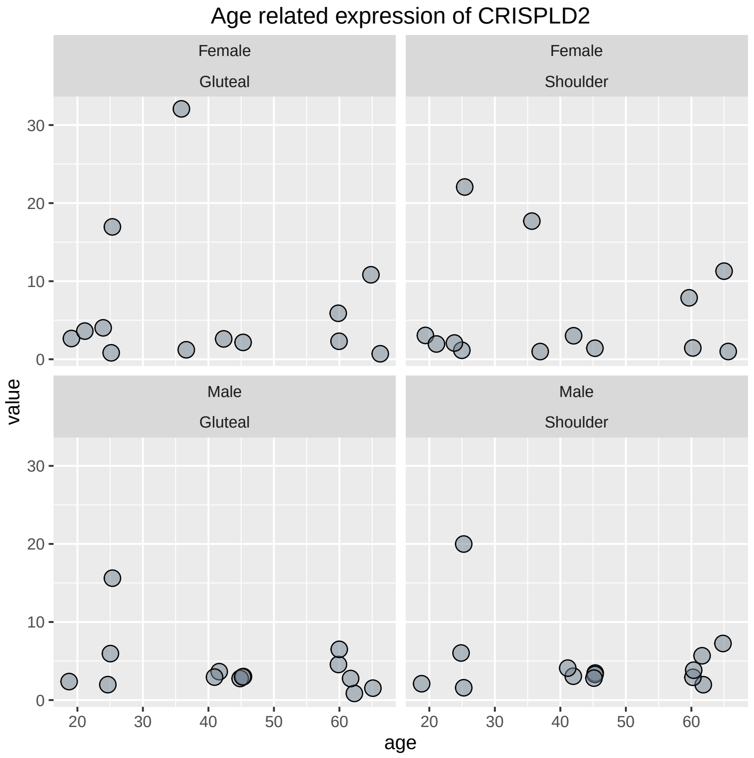

Figure 142: Loess regression for exon align depth

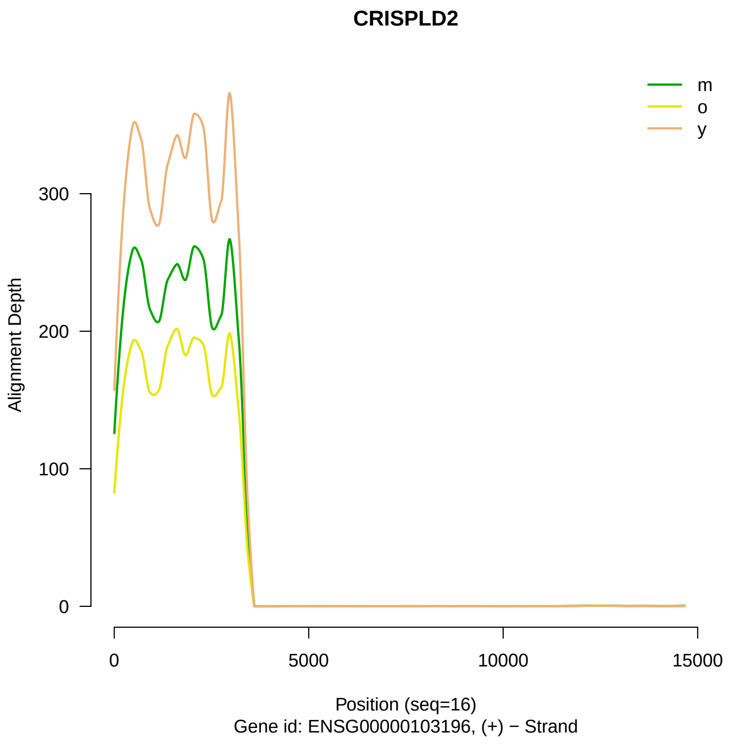

Figure 143: edgeR QLF test based CPM estimates

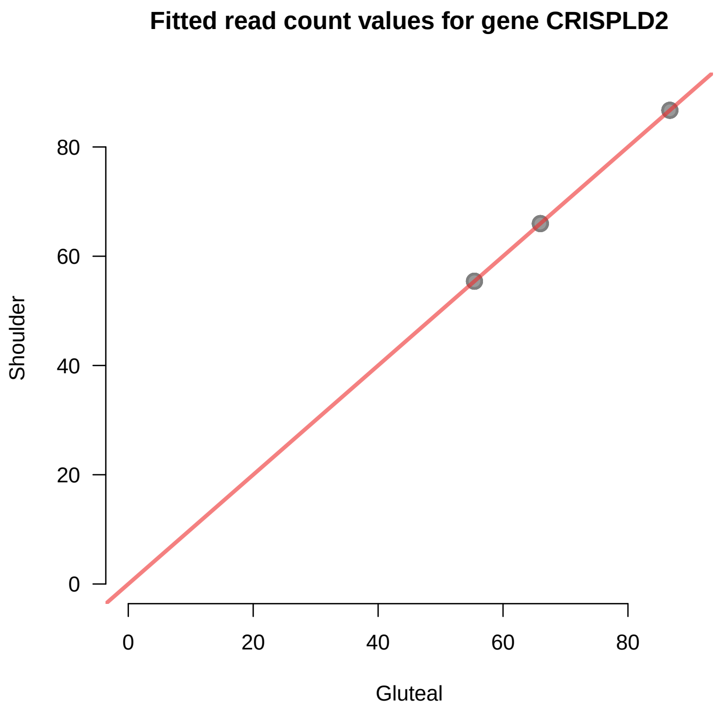

Figure 144: ReadExpSet based genewise CPM estimates

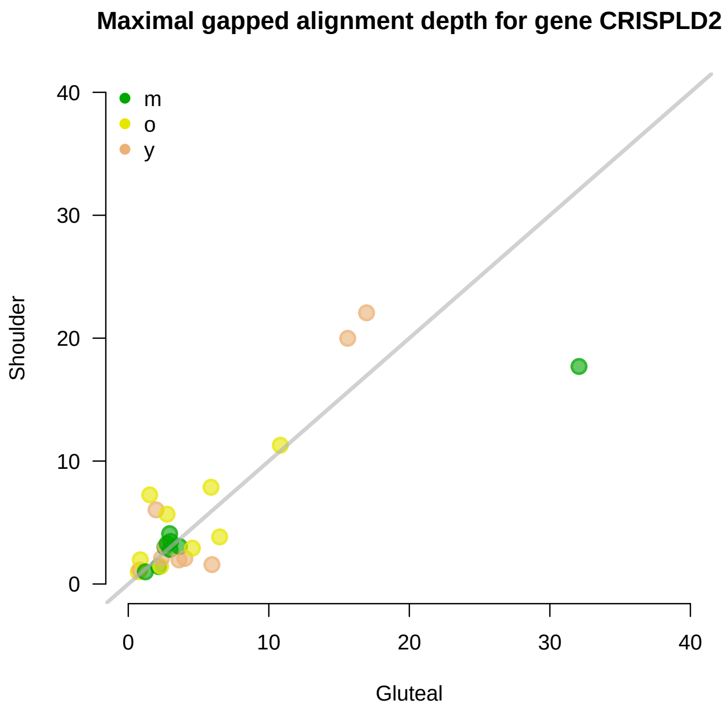

3.25 RP11-309L24.6

Parameter	Value
gene_name	RP11-309L24.6
gene_id	ENSG00000224163
maxAld	6288
old	down
seqid	7
strand	-
start	128912732
end	128914063
descr	

Table 26: Gene identifcation

Gene expression estimates for Age

Influence of UV exposition on gene expression

Figure 145: Gene expression estimates based on CPM (SummarizeOveraps)

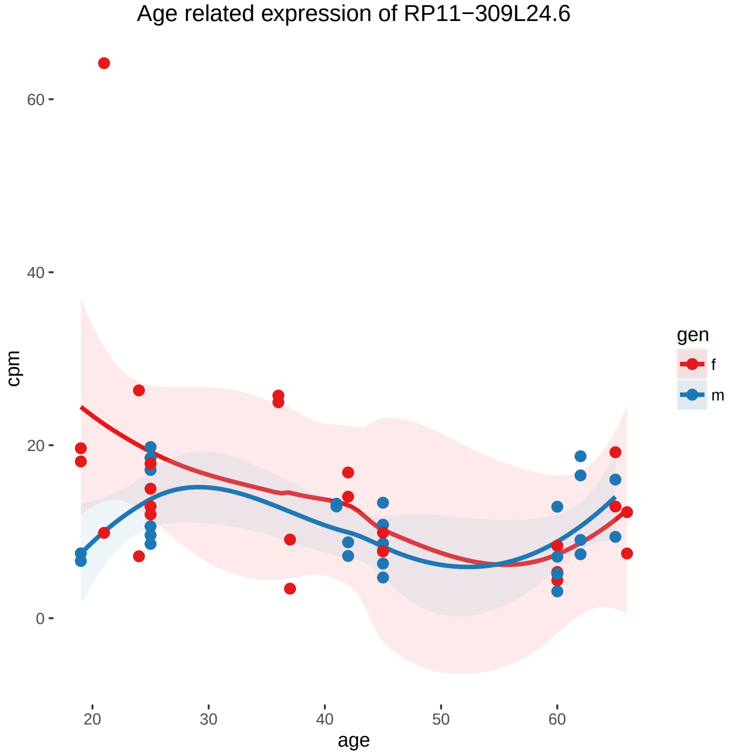

Figure 146: edgeR QLF test based CPM estimates

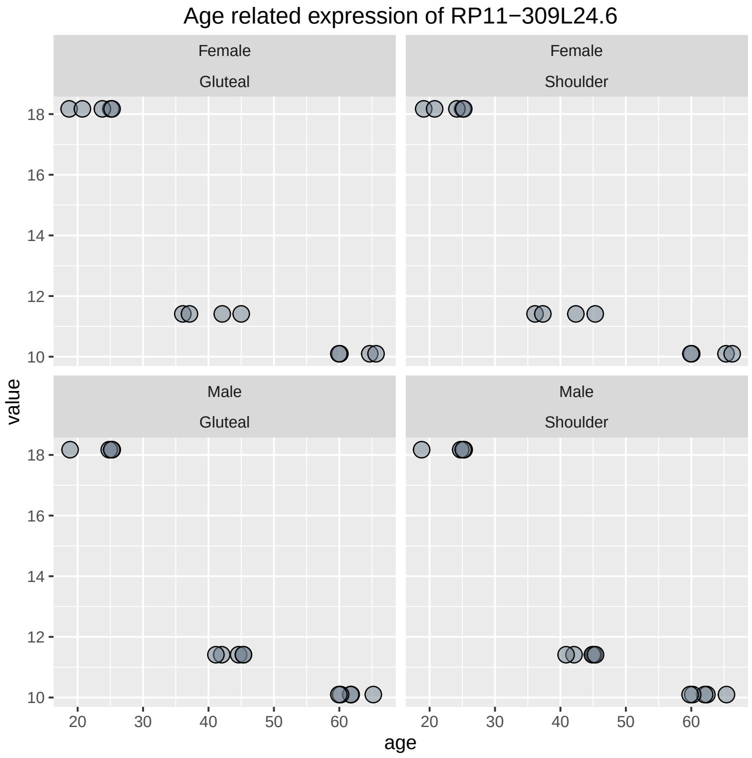

Figure 147: ReadExpSet based genewise CPM estimates

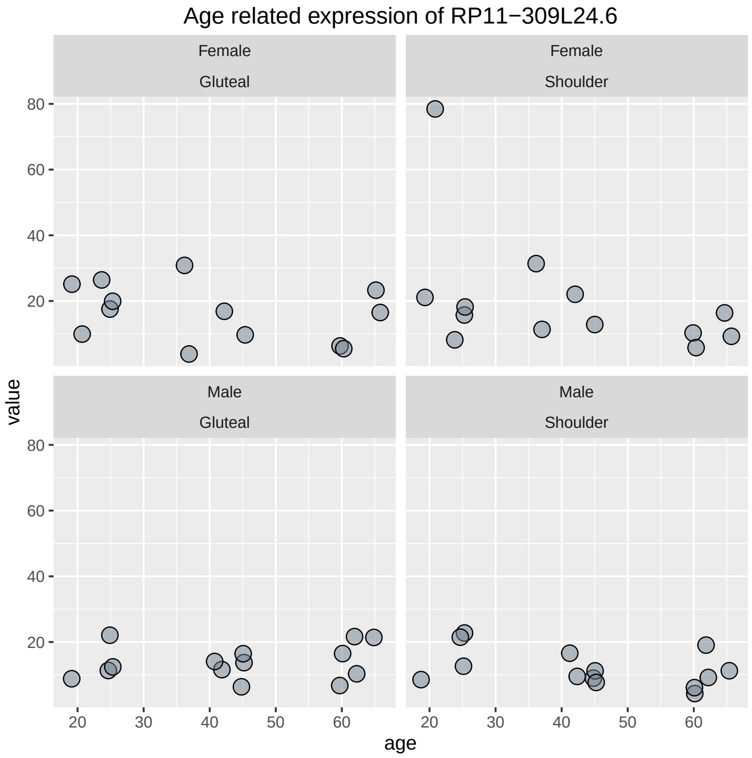

Figure 148: Loess regression for exon align depth

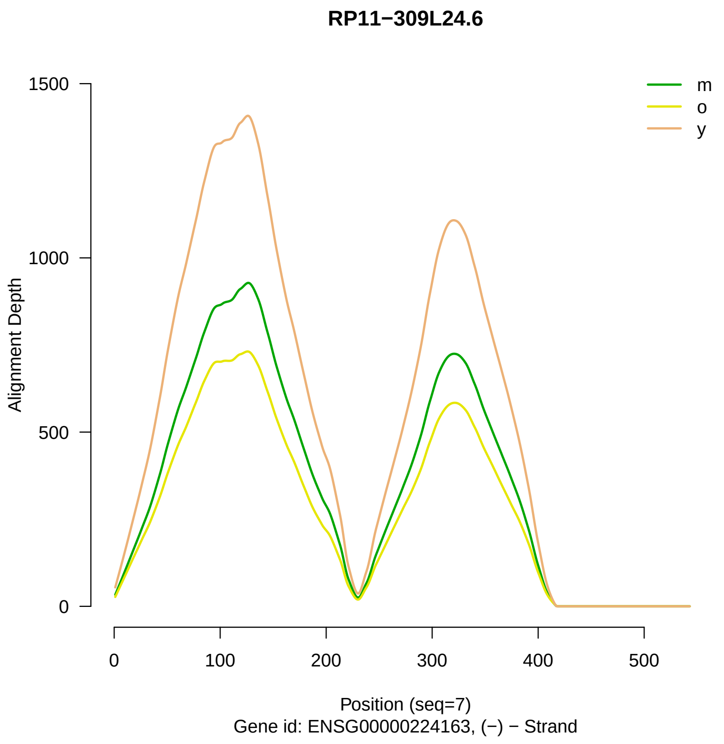

Figure 149: edgeR QLF test based CPM estimates

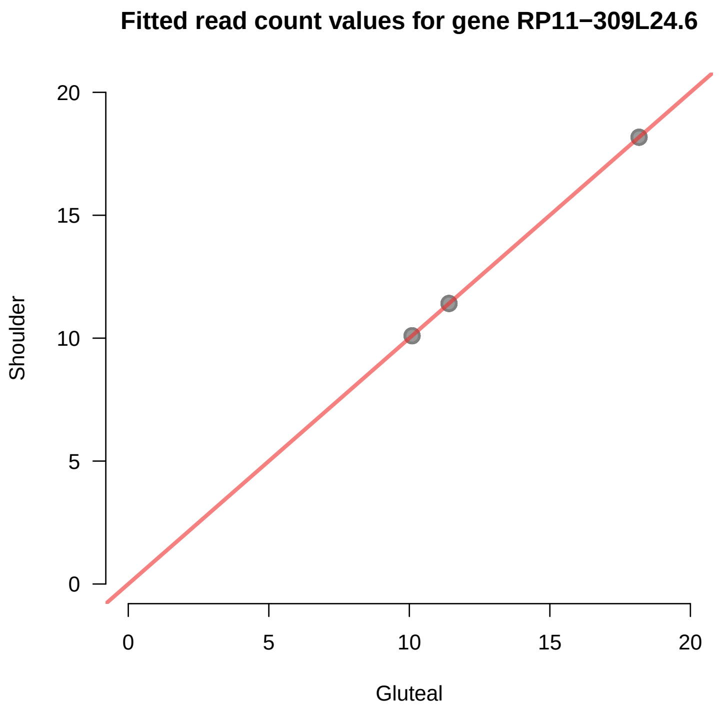

Figure 150: ReadExpSet based genewise CPM estimates

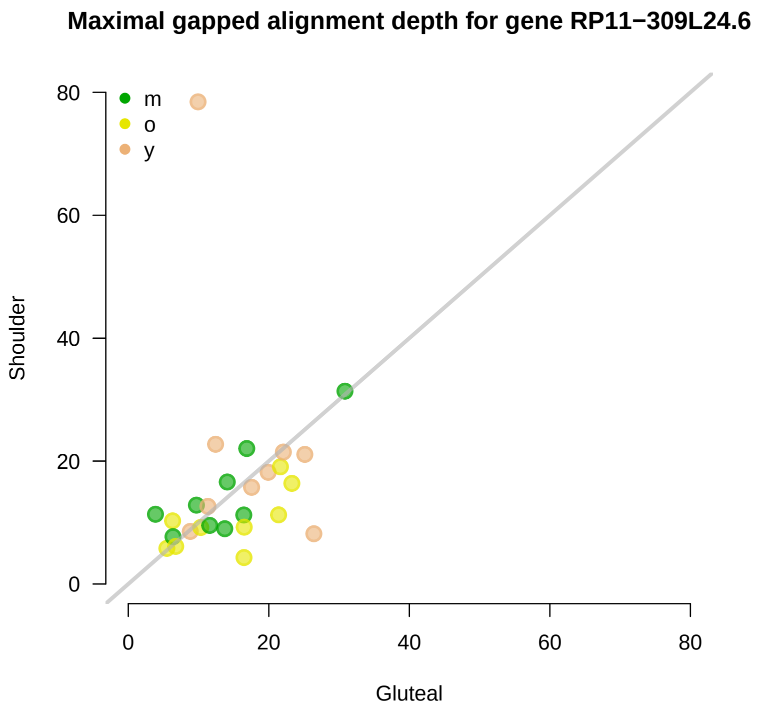

3.26 ZNF385D

Parameter	Value
gene_name	ZNF385D
gene_id	ENSG00000151789
maxald	529
old	up
seqid	3
strand	-
start	21415071
end	22373321
descr	zinc finger protein 385D

Table 27: Gene identifcation

Gene expression estimates for Age

Influence of UV exposition on gene expression

Figure 151: Gene expression estimates based on CPM (SummarizeOveraps)

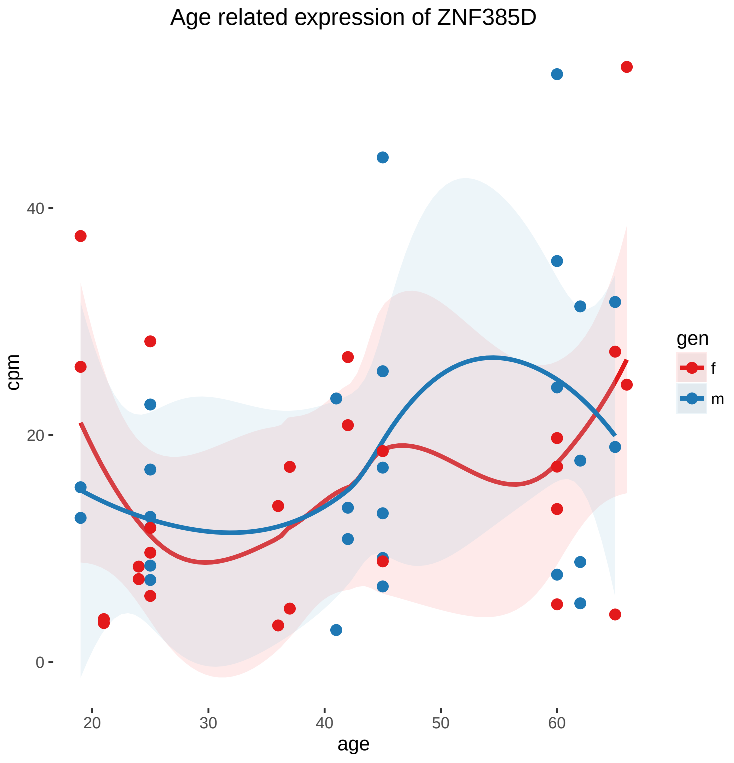

Figure 152: edgeR QLF test based CPM estimates

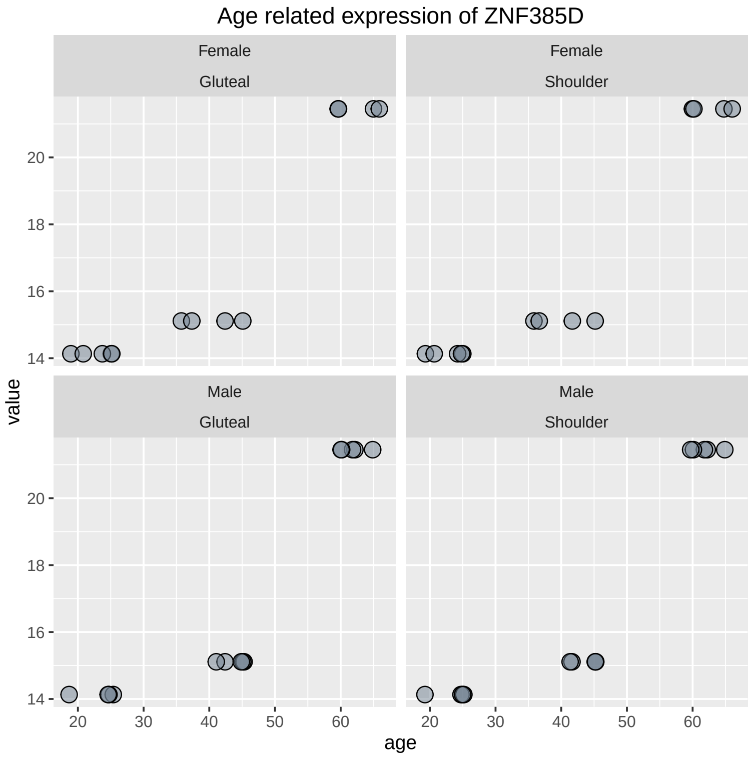

Figure 153: ReadExpSet based genewise CPM estimates

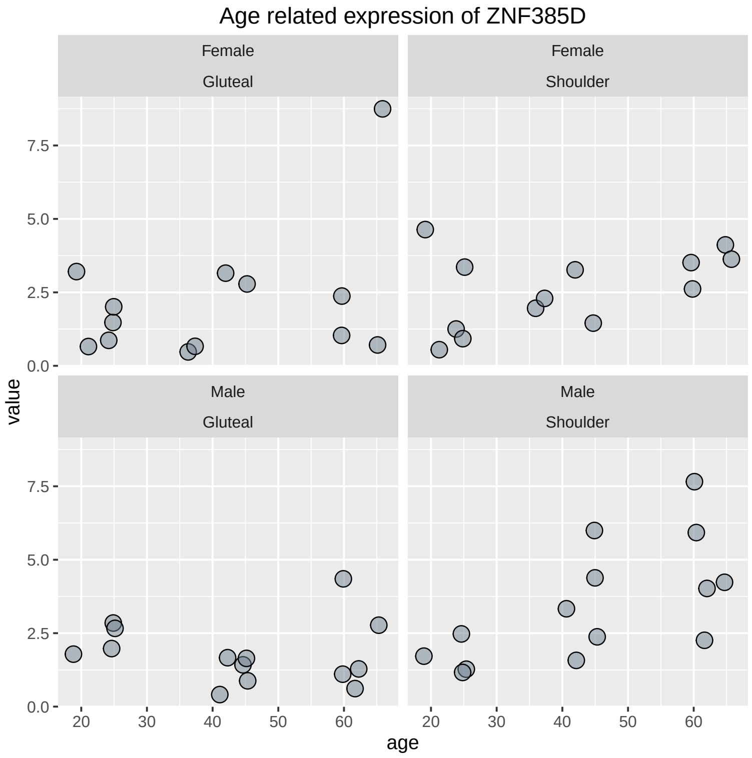

Figure 154: Loess regression for exon align depth

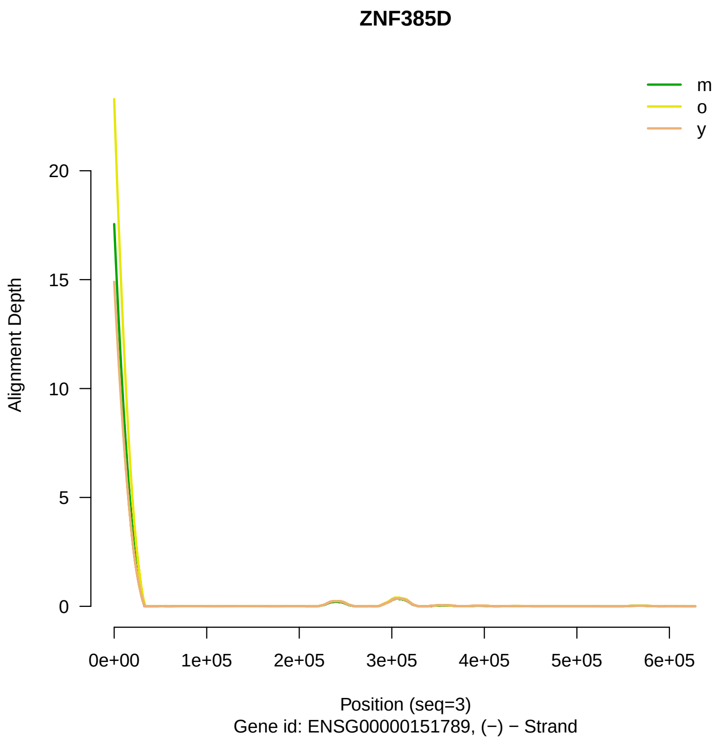

Figure 155: edgeR QLF test based CPM estimates

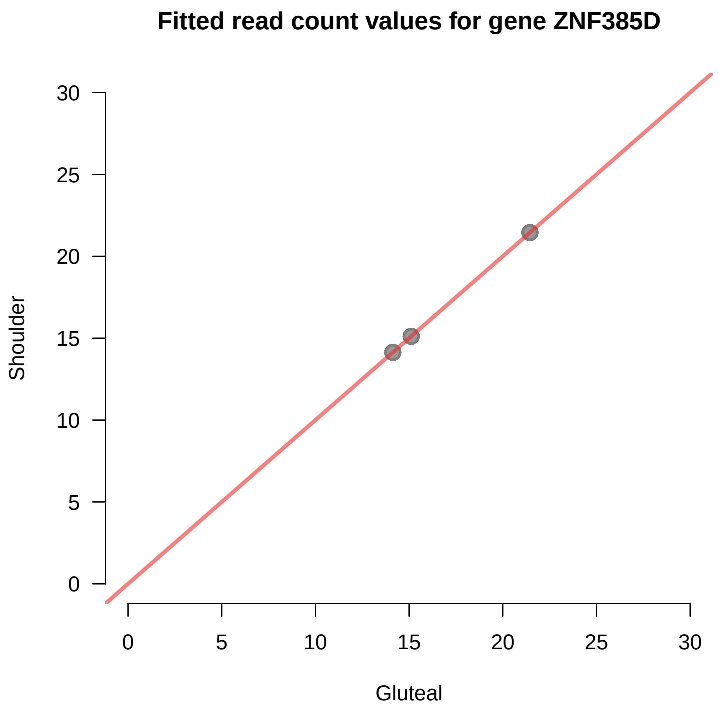

Figure 156: ReadExpSet based genewise CPM estimates

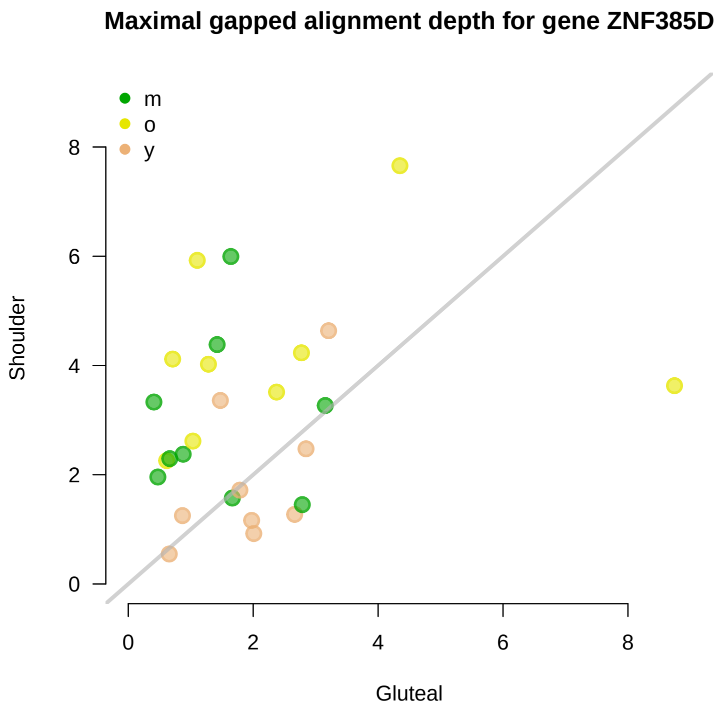

3.27 FGFRL1

Parameter	Value
gene_name	FGFRL1
gene_id	ENSG00000127418
maxald	1174
old	down
seqid	4
strand	+
start	1009936
end	1026897
descr	fibroblast growth factor receptor-like 1

Table 28: Gene identifcation

Gene expression estimates for Age

Influence of UV exposition on gene expression

Figure 157: Gene expression estimates based on CPM (SummarizeOverlaps)

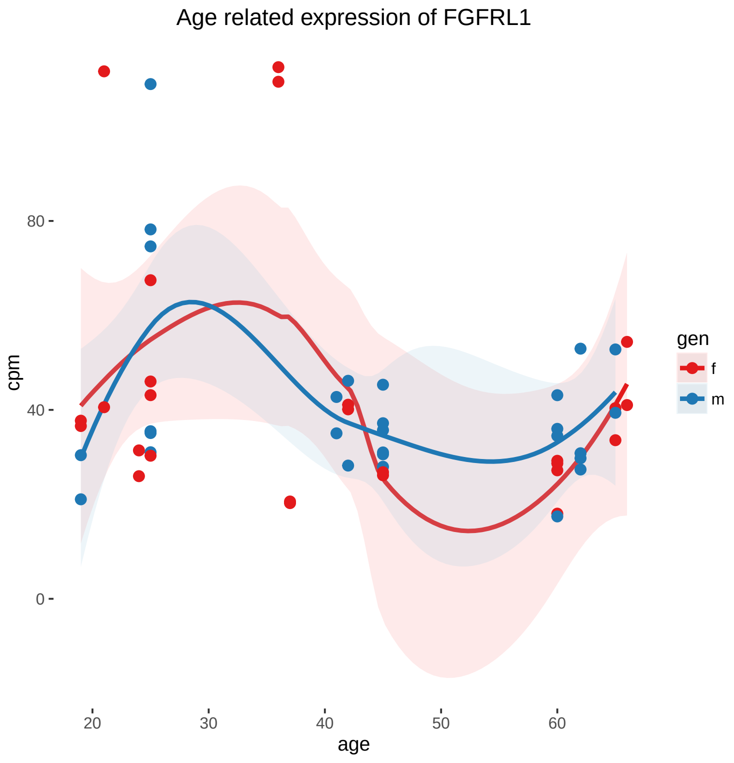

Figure 158: edgeR QLF test based CPM estimates

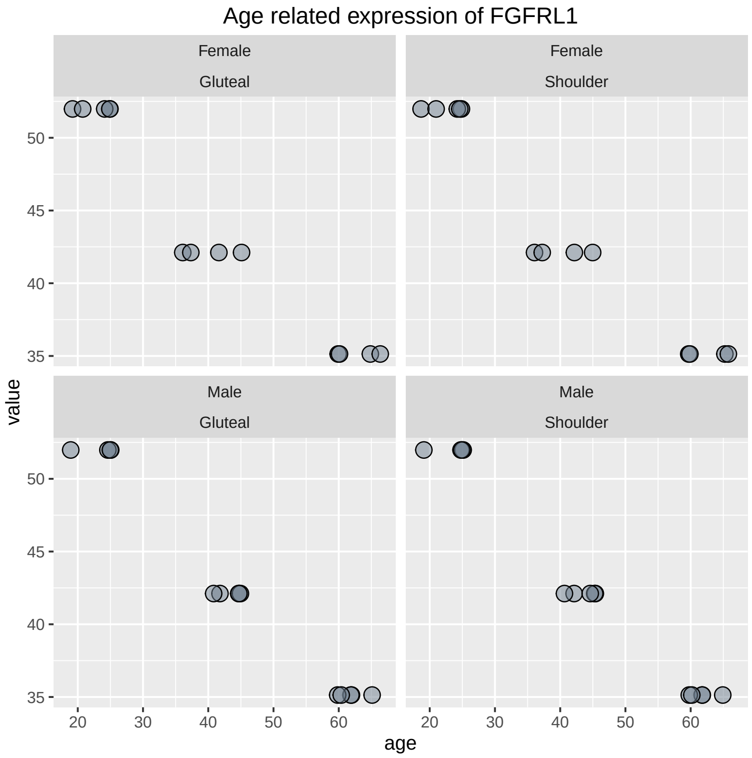

Figure 159: ReadExpSet based genewise CPM estimates

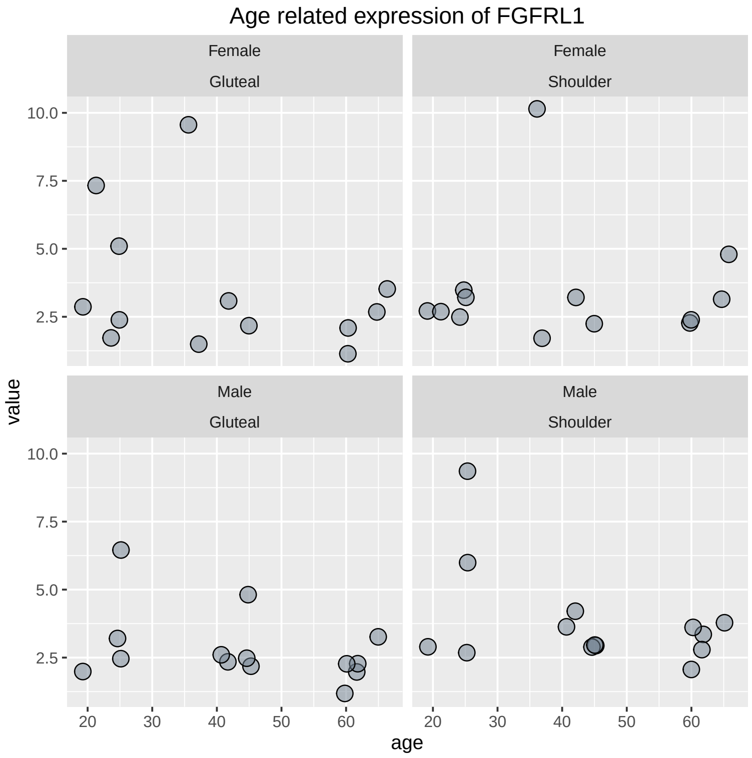

Figure 160: Loess regression for exon align depth

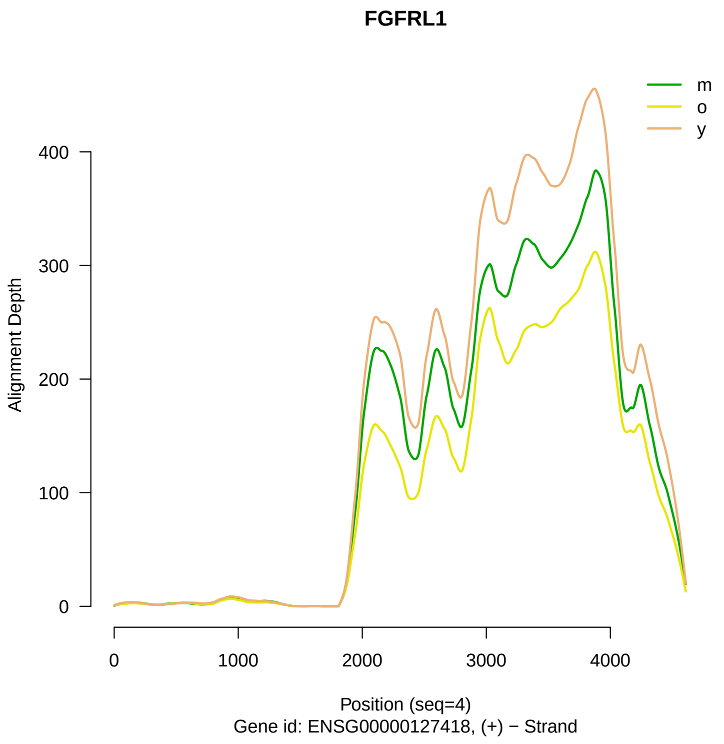

Figure 161: edgeR QLF test based CPM estimates

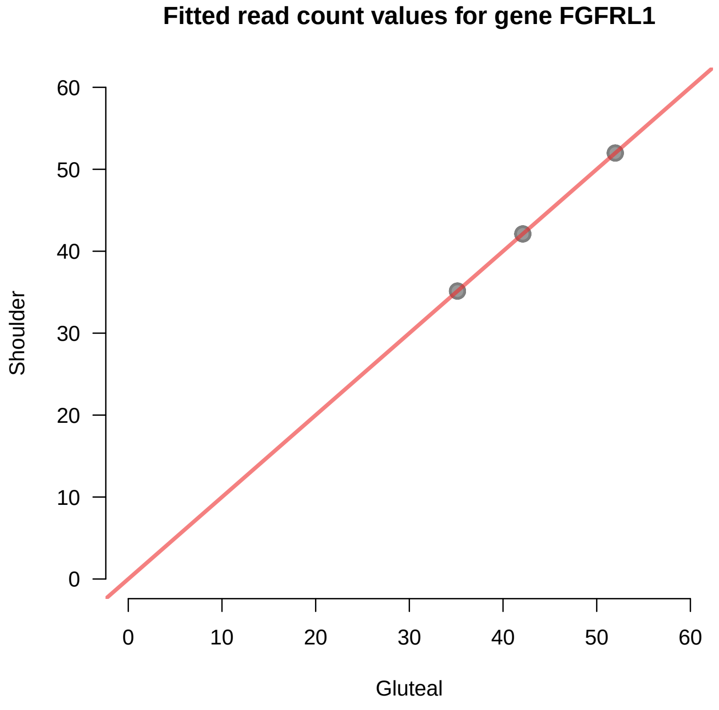

Figure 162: ReadExpSet based genewise CPM estimates

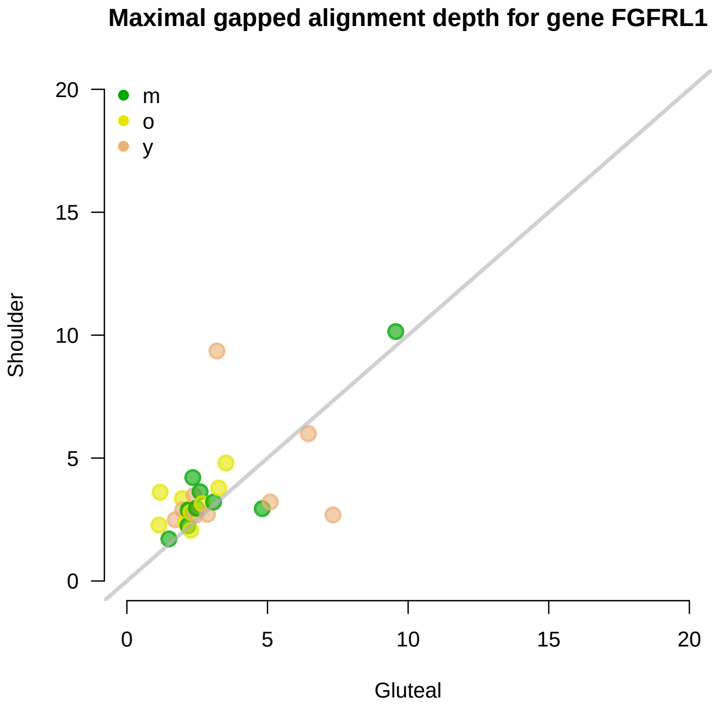

3.28 CKB

Parameter	Value
gene_name	CKB
gene_id	ENSG00000166165
maxald	2224
old	down
seqid	14
strand	-
start	103519659
end	103523111
descr	creatine kinase, brain

Table 29: Gene identifcation

Gene expression estimates for Age

Influence of UV exposition on gene expression

Figure 163: Gene expression estimates based on CPM (SummarizeOveraps)

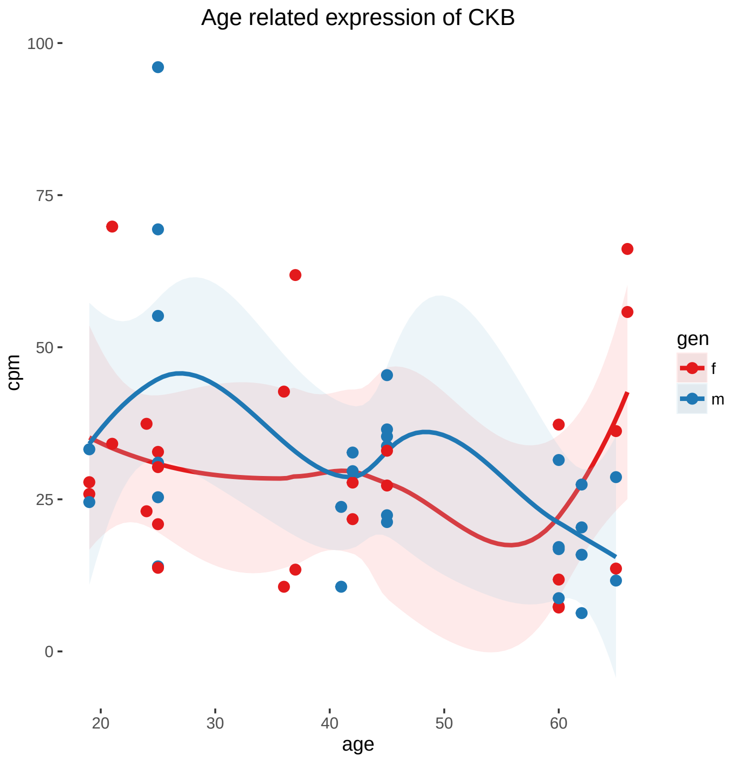

Figure 164: edgeR QLF test based CPM estimates

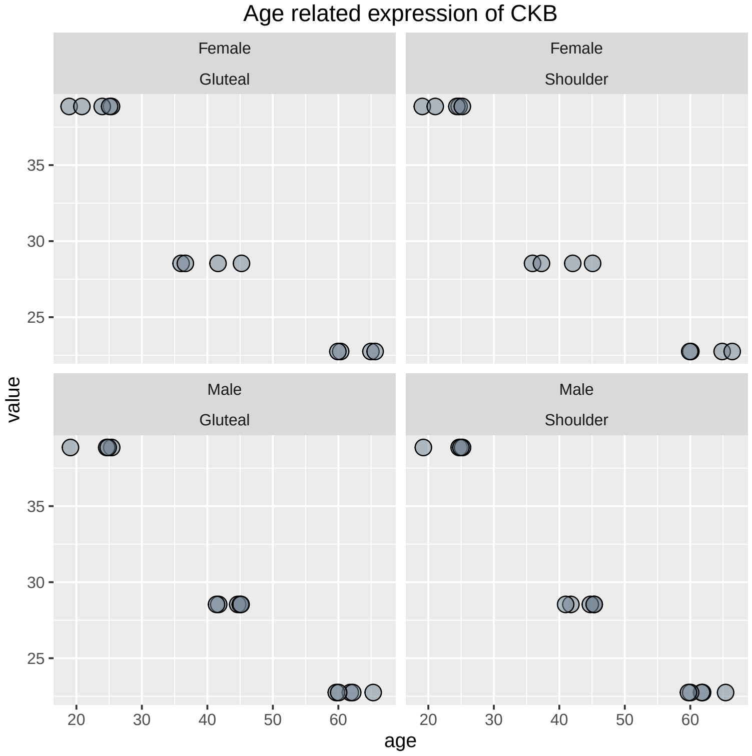

Figure 165: ReadExpSet based genewise CPM estimates

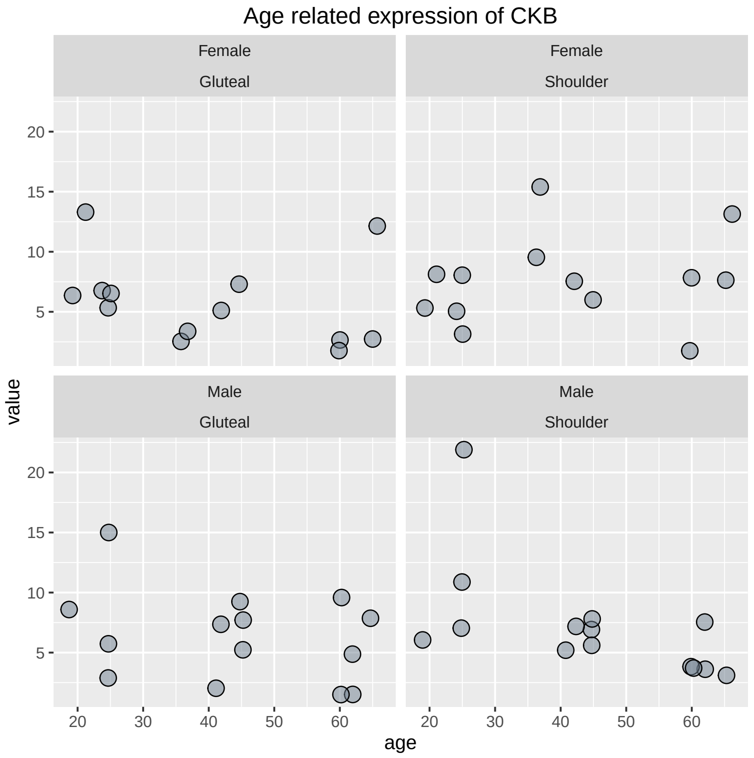

Figure 166: Loess regression for exon align depth

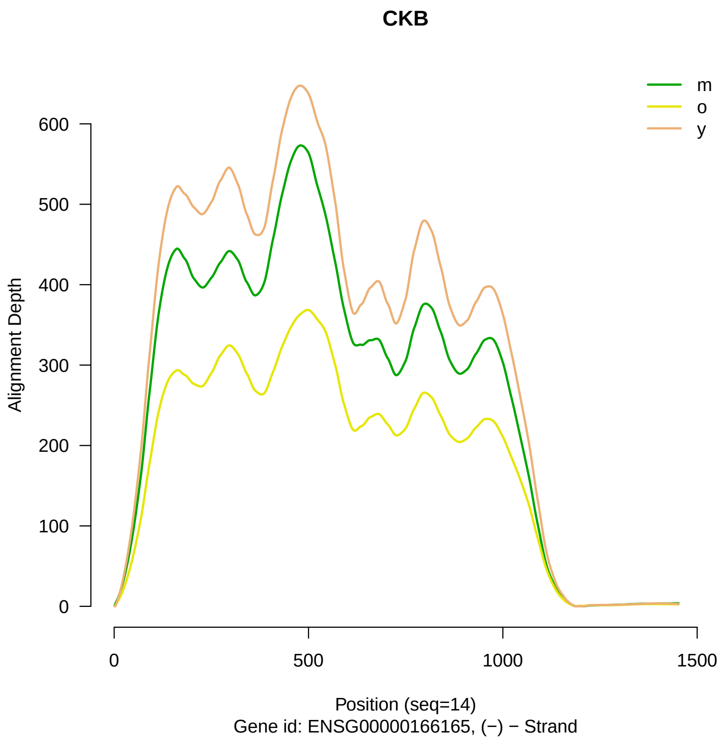

Figure 167: edgeR QLF test based CPM estimates

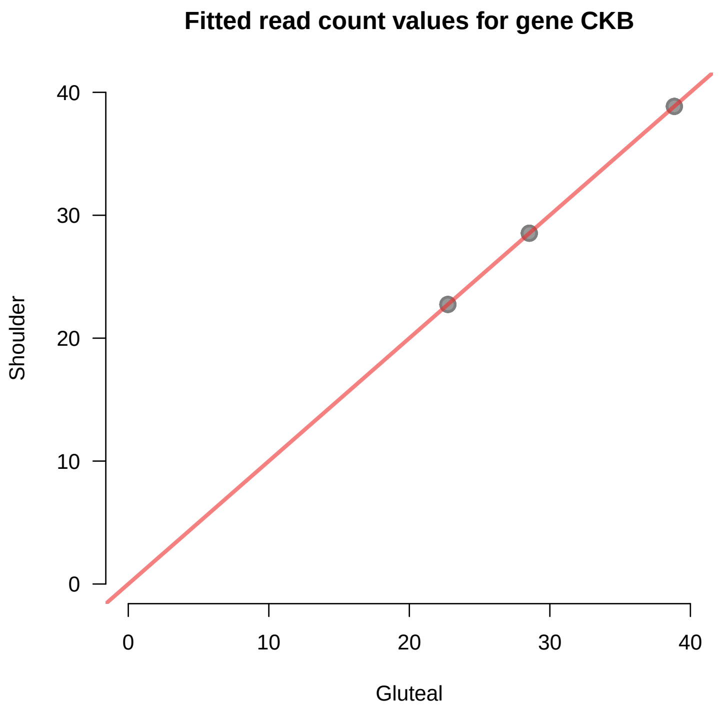

Figure 168: ReadExpSet based genewise CPM estimates

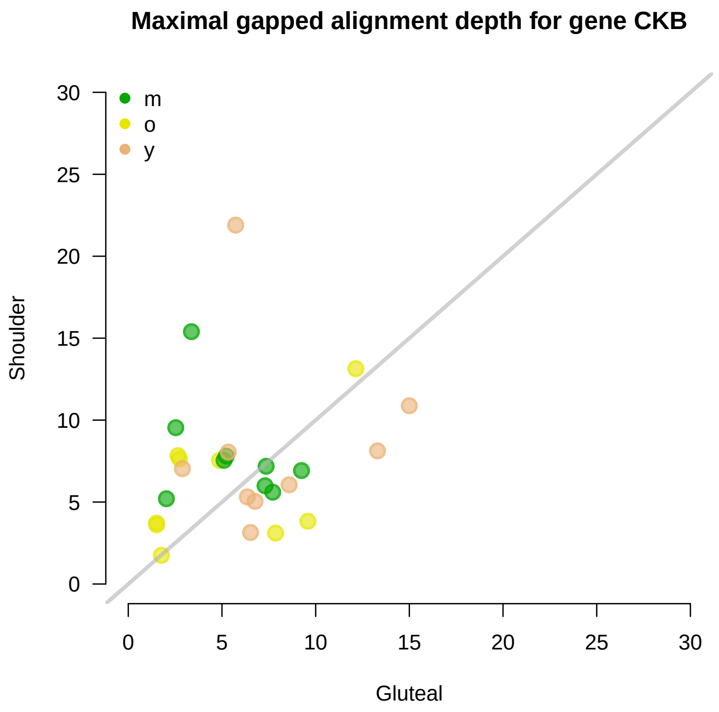

3.29 FILIP1L

Parameter	Value
gene_name	FILIP1L
gene_id	ENSG00000168386
maxald	2028
old	down
seqid	3
strand	-
start	99830141
end	100114513
descr	filamin A interacting protein 1-like

Table 30: Gene identifcation

Gene expression estimates for Age

Influence of UV exposition on gene expression

Figure 169: Gene expression estimates based on CPM (SummarizeOveraps)

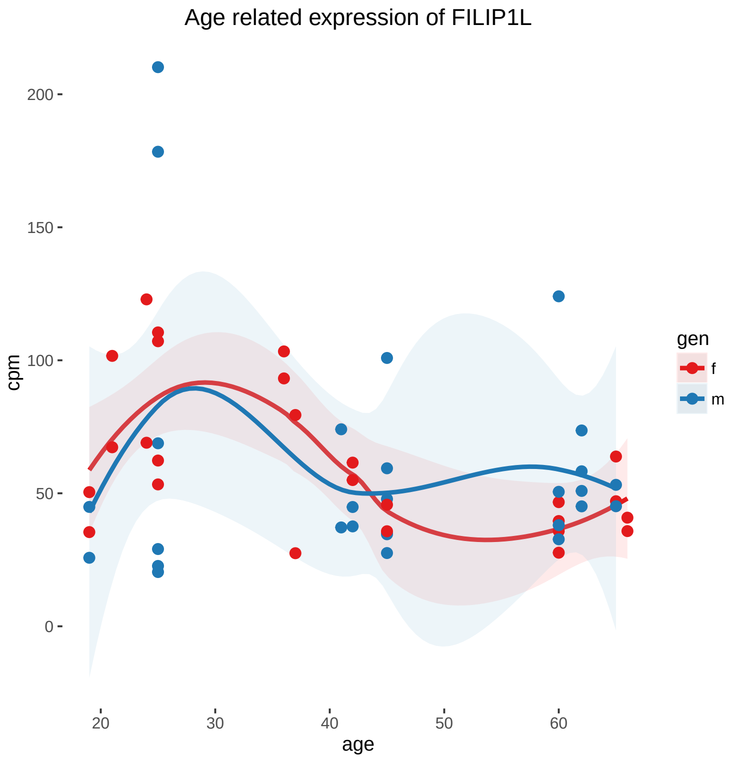

Figure 170: edgeR QLF test based CPM estimates

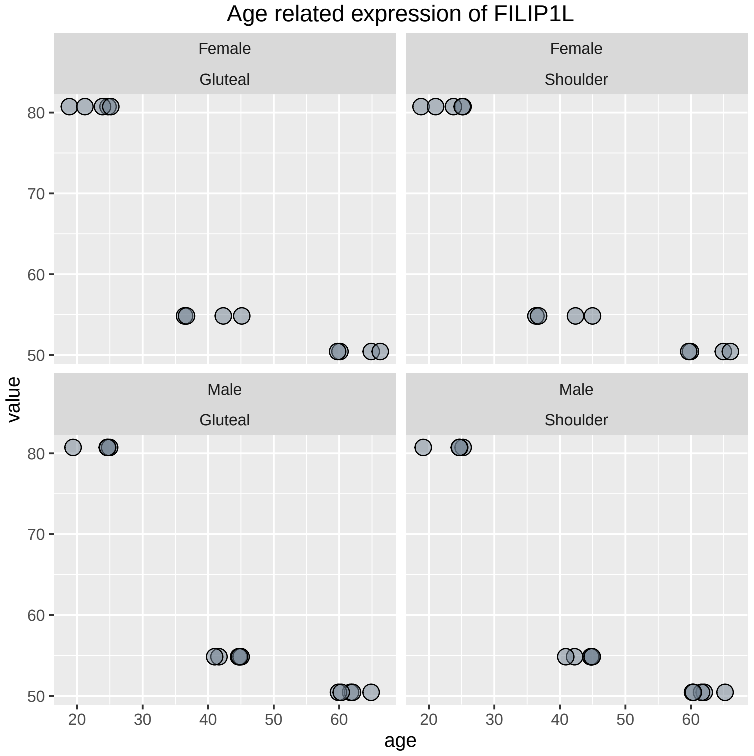

Figure 171: ReadExpSet based genewise CPM estimates

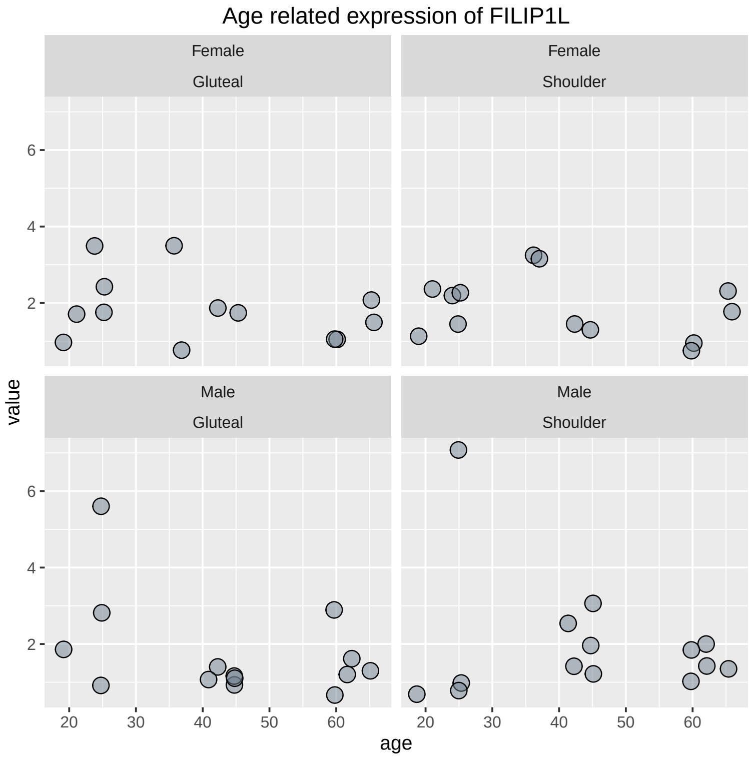

Figure 172: Loess regression for exon align depth

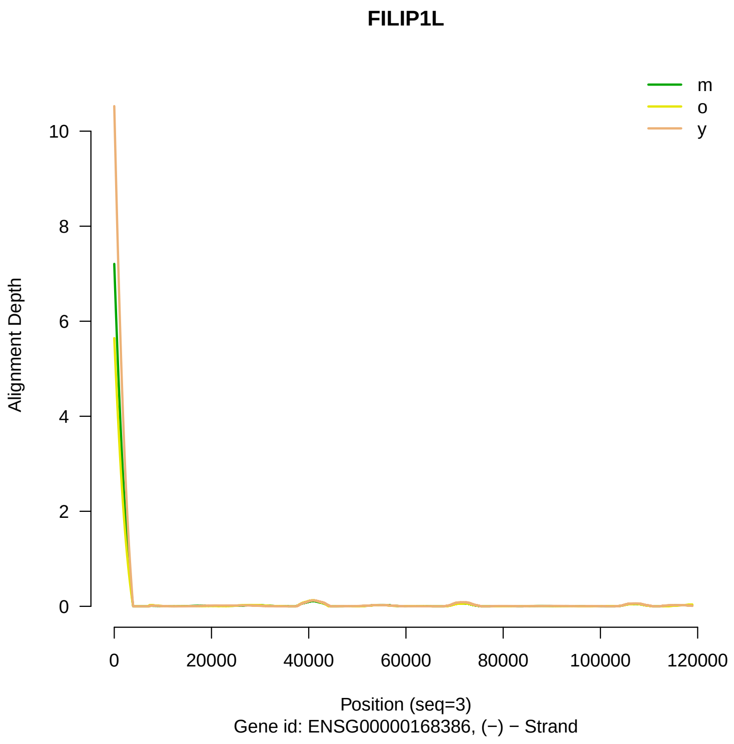

Figure 173: edgeR QLF test based CPM estimates

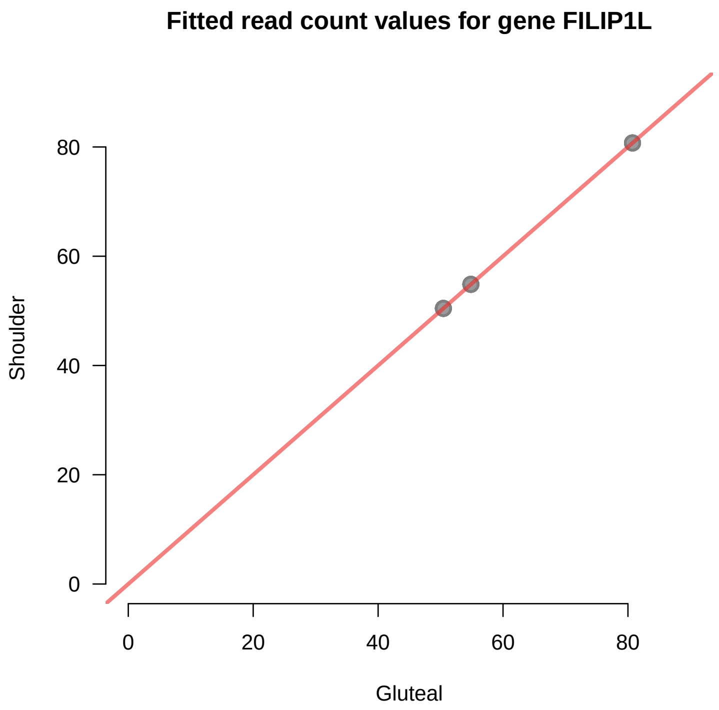

Figure 174: ReadExpSet based genewise CPM estimates

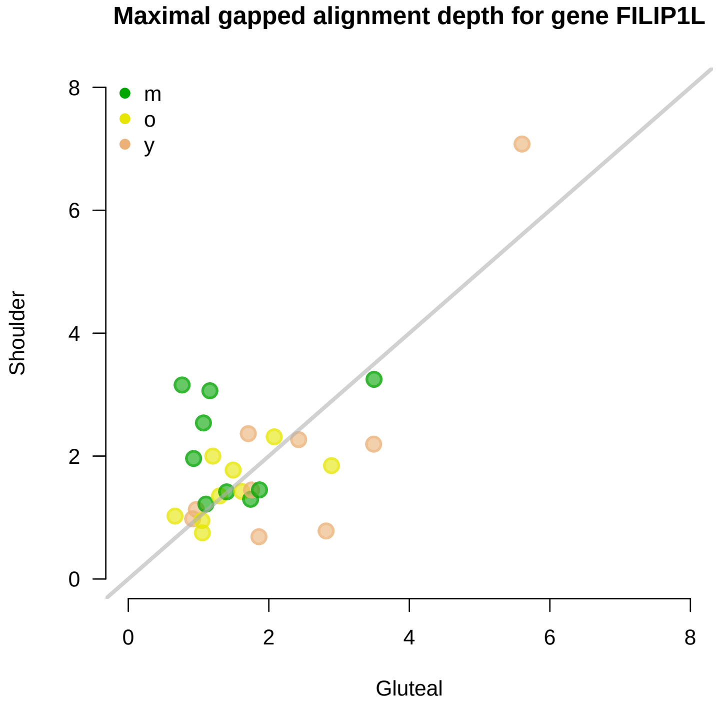

3.30 GJA1

Parameter	Value
gene_name	GJA1
gene_id	ENSG00000152661
maxald	12333
old	up
seqid	6
strand	+
start	121435692
end	121449727
descr	gap junction protein, alpha 1, 43kDa

Table 31: Gene identifcation

Gene expression estimates for Age

Influence of UV exposition on gene expression

Figure 175: Gene expression estimates based on CPM (SummarizeOveraps)

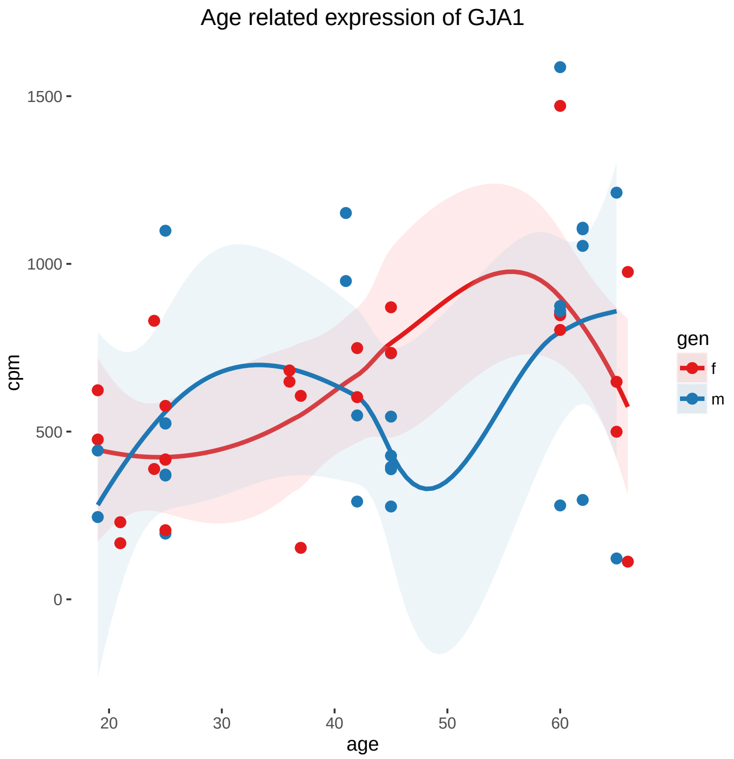

Figure 176: edgeR QLF test based CPM estimates

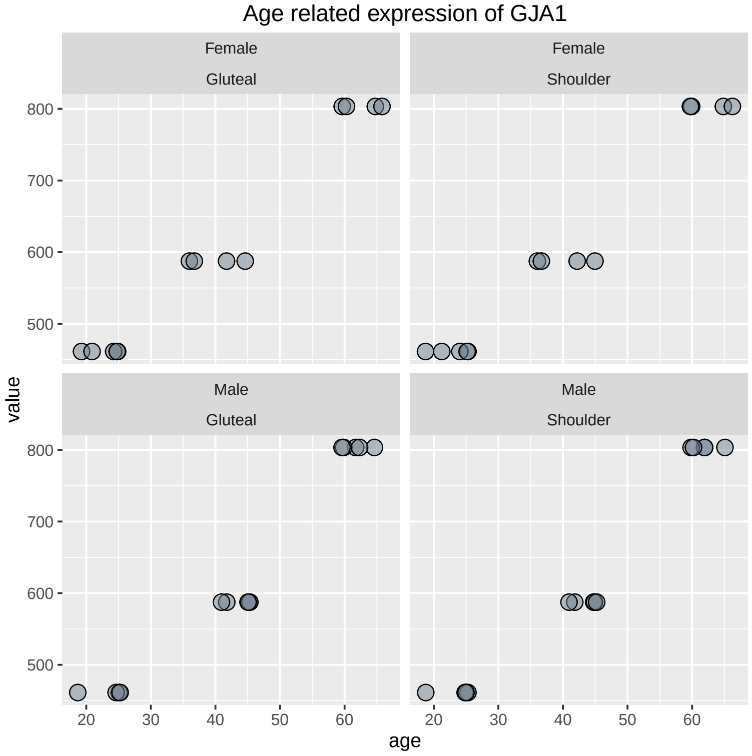

Figure 177: ReadExpSet based genewise CPM estimates

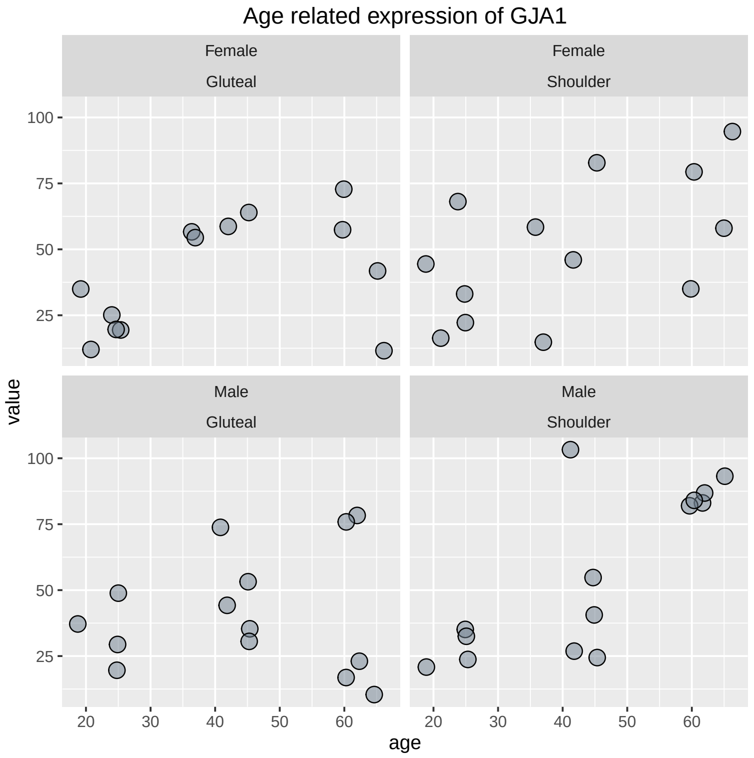

Figure 178: Loess regression for exon align depth

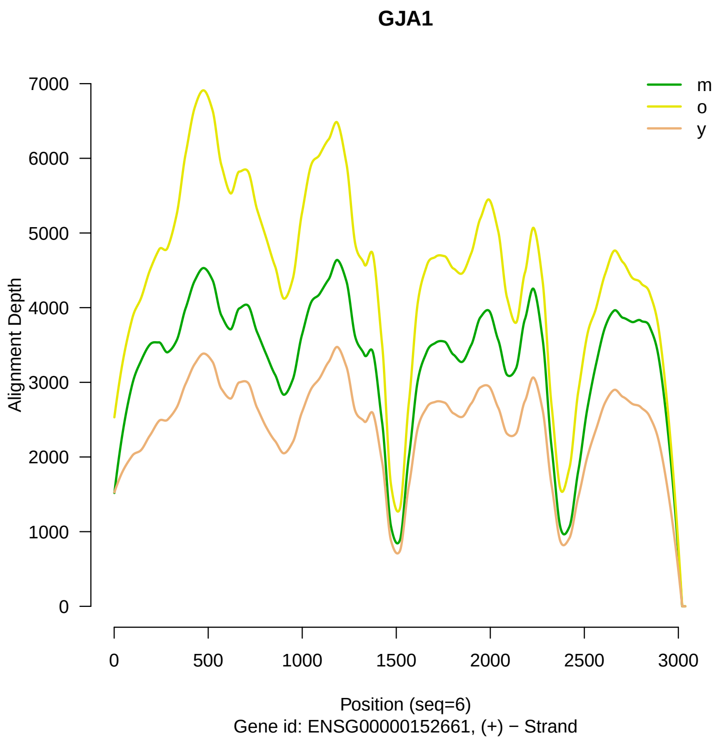

Figure 179: edgeR QLF test based CPM estimates

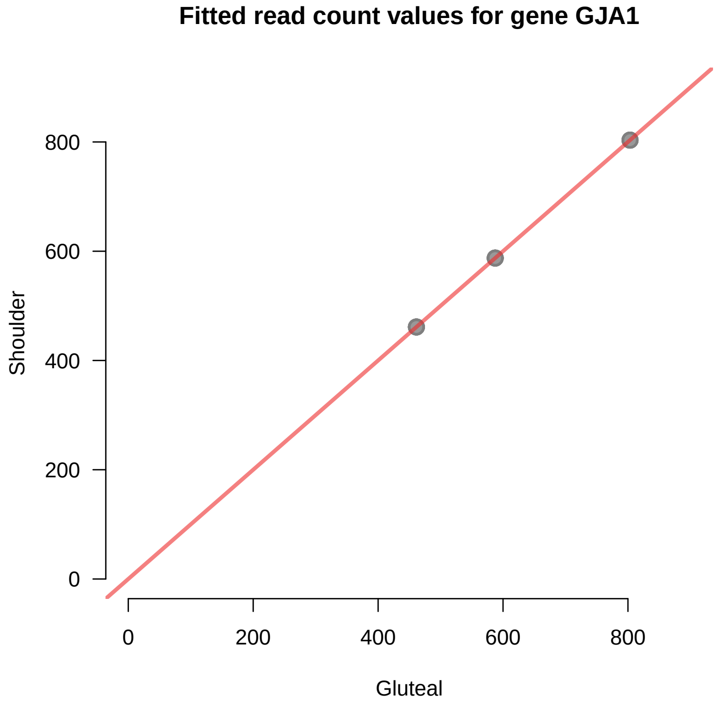

Figure 180: ReadExpSet based genewise CPM estimates

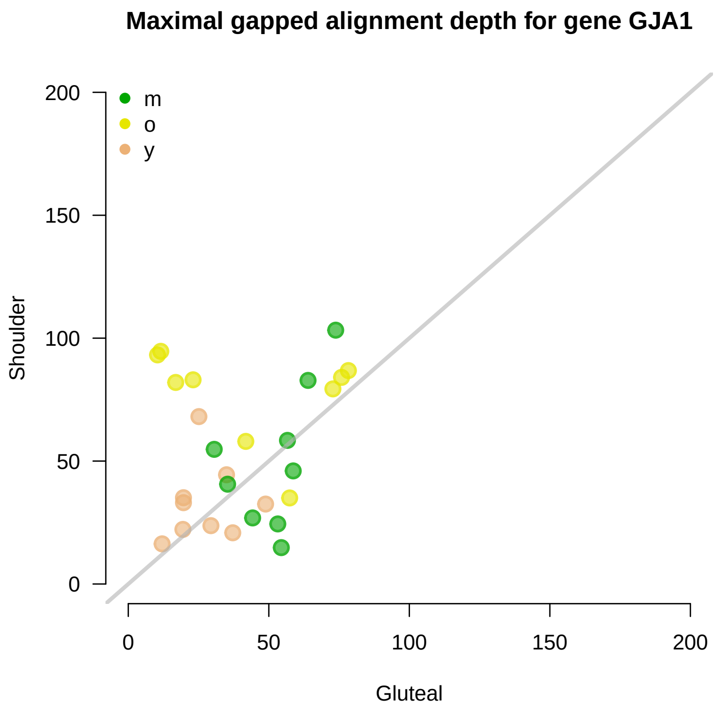

3.31 ENC1

Parameter	Value
gene_name	ENC1
gene_id	ENSG00000171617
maxald	1200
old	down
seqid	5
strand	-
start	74627406
end	74641424
descr	ectodermal-neural cortex 1 (with BTB domain)

Table 32: Gene identifcation

Gene expression estimates for Age

Influence of UV exposition on gene expression

Figure 181: Gene expression estimates based on CPM (SummarizeOveraps)

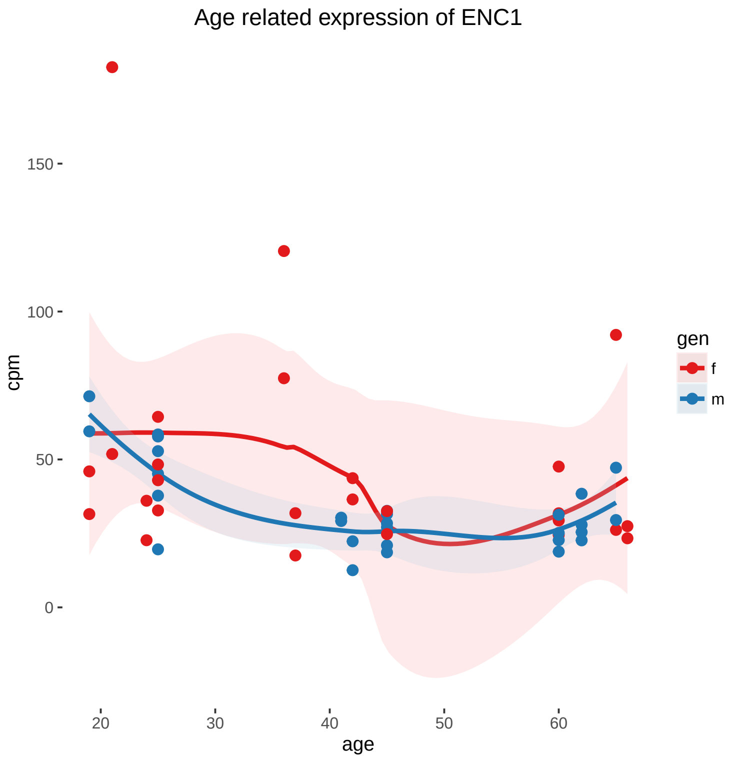

Figure 182: edgeR QLF test based CPM estimates

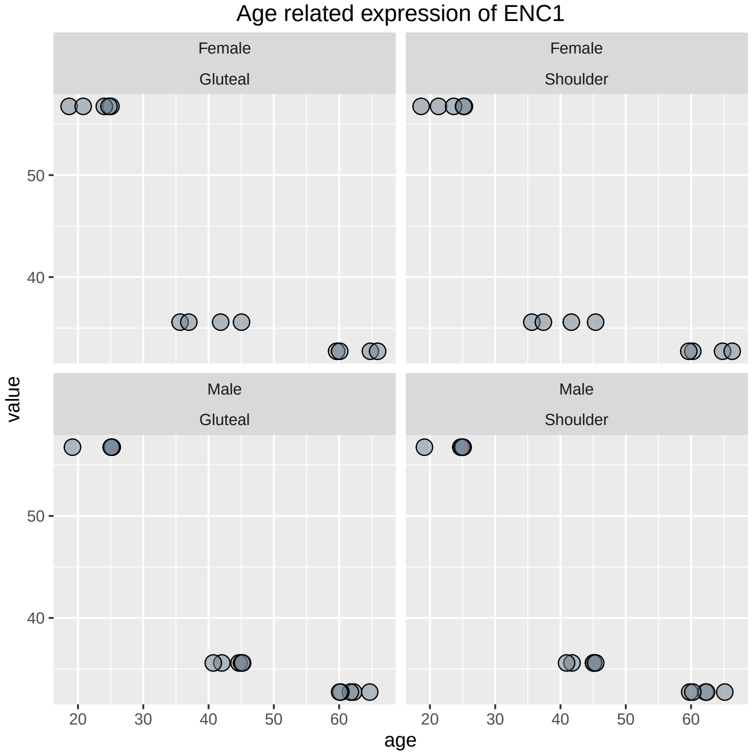

Figure 183: ReadExpSet based genewise CPM estimates

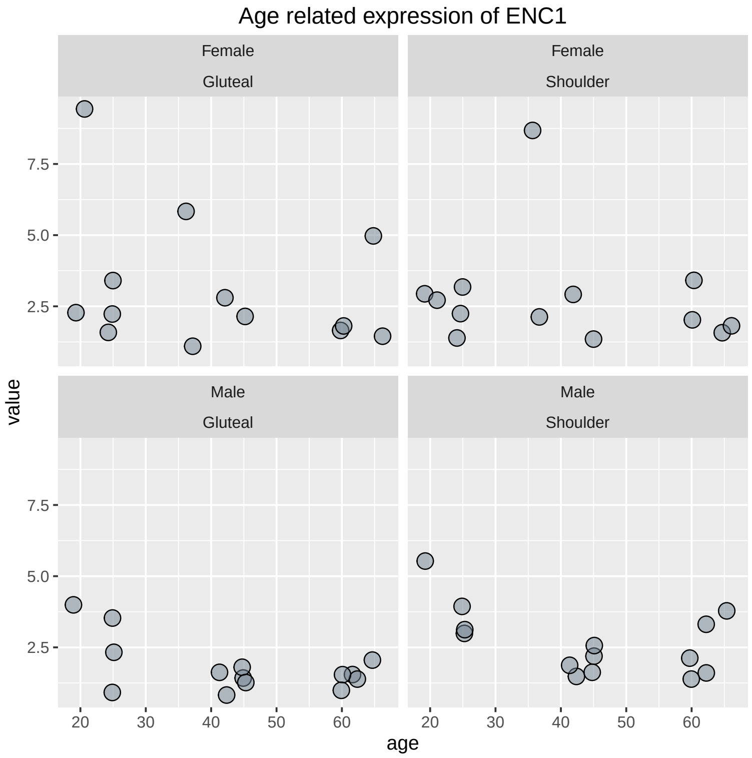

Figure 184: Loess regression for exon align depth

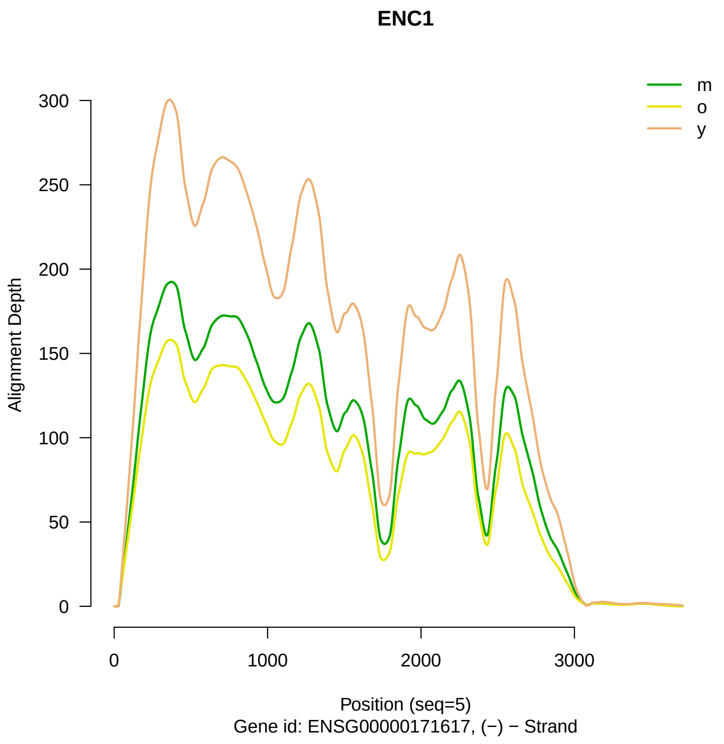

Figure 185: edgeR QLF test based CPM estimates

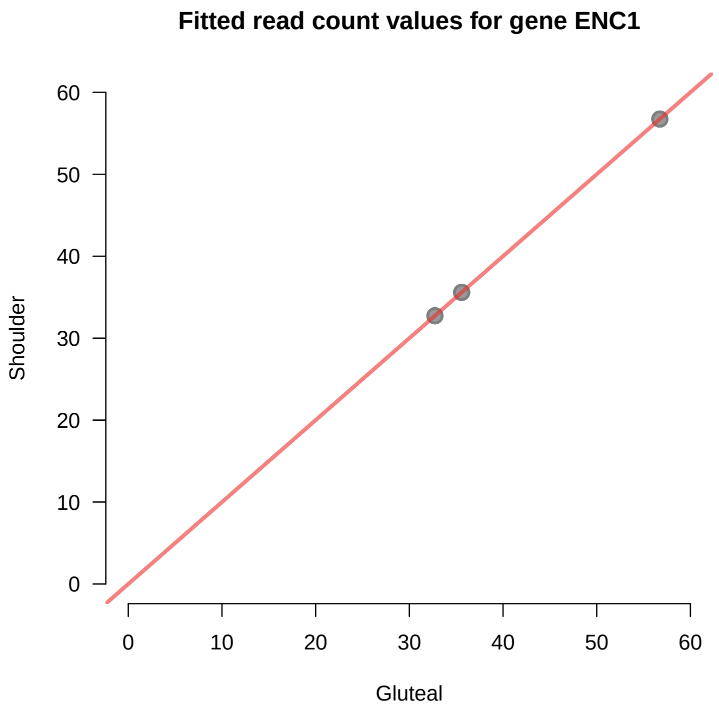

Figure 186: ReadExpSet based genewise CPM estimates

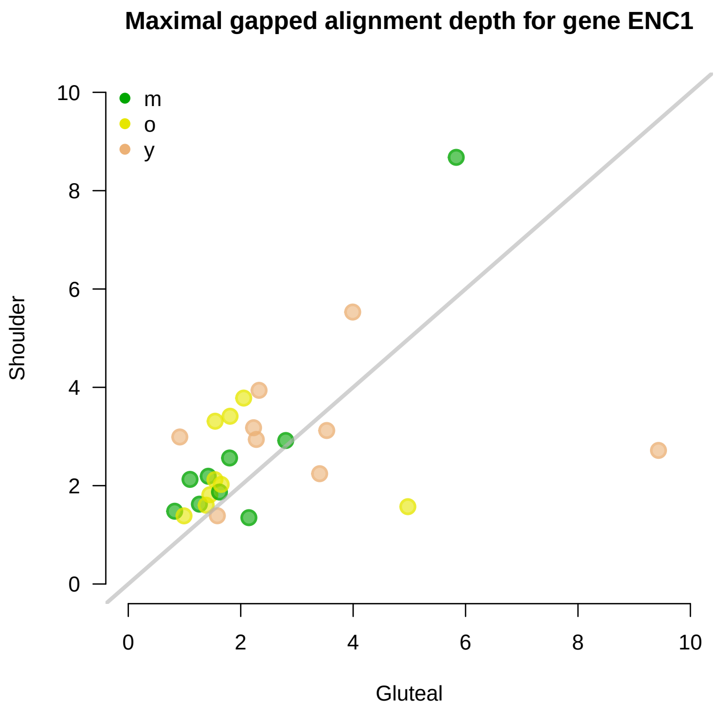

3.32 SH2D4A

Parameter	Value
gene_name	SH2D4A
gene_id	ENSG00000104611
maxald	5544
old	down
seqid	8
strand	+
start	19313617
end	19396218
descr	SH2 domain containing 4A

Table 33: Gene identifcation

Gene expression estimates for Age

Influence of UV exposition on gene expression

Figure 187: Gene expression estimates based on CPM (SummarizeOveraps)

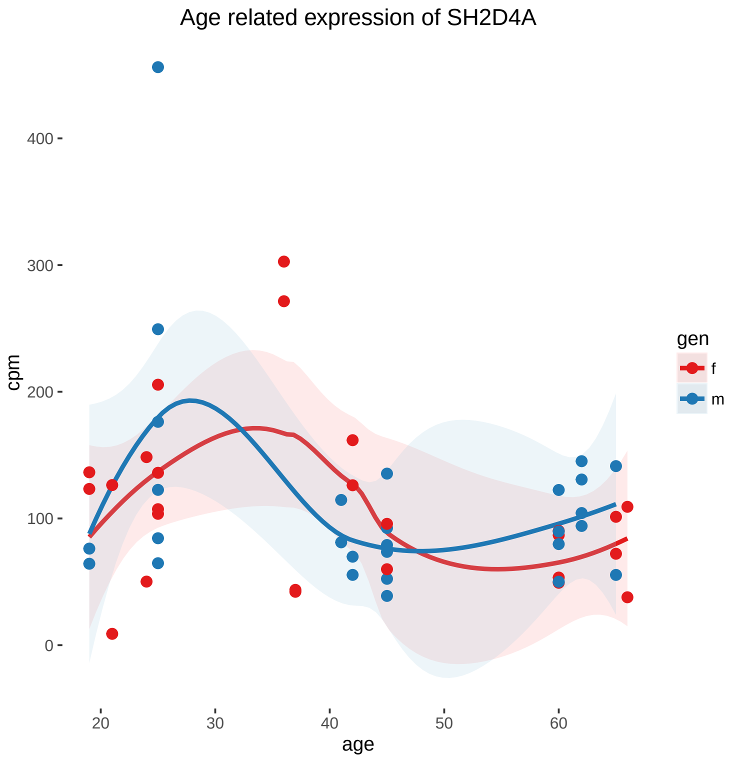

Figure 188: edgeR QLF test based CPM estimates

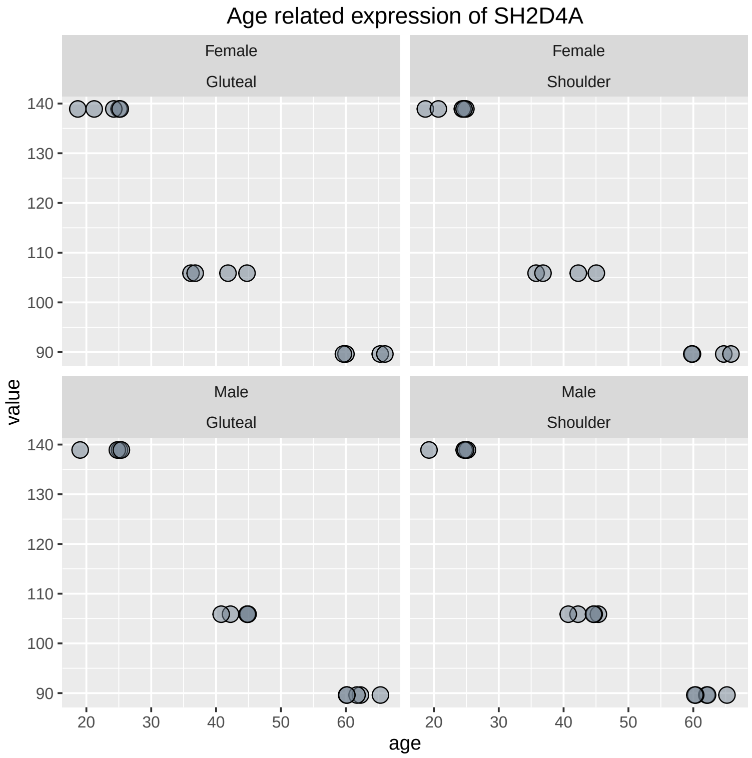

Figure 189: ReadExpSet based genewise CPM estimates

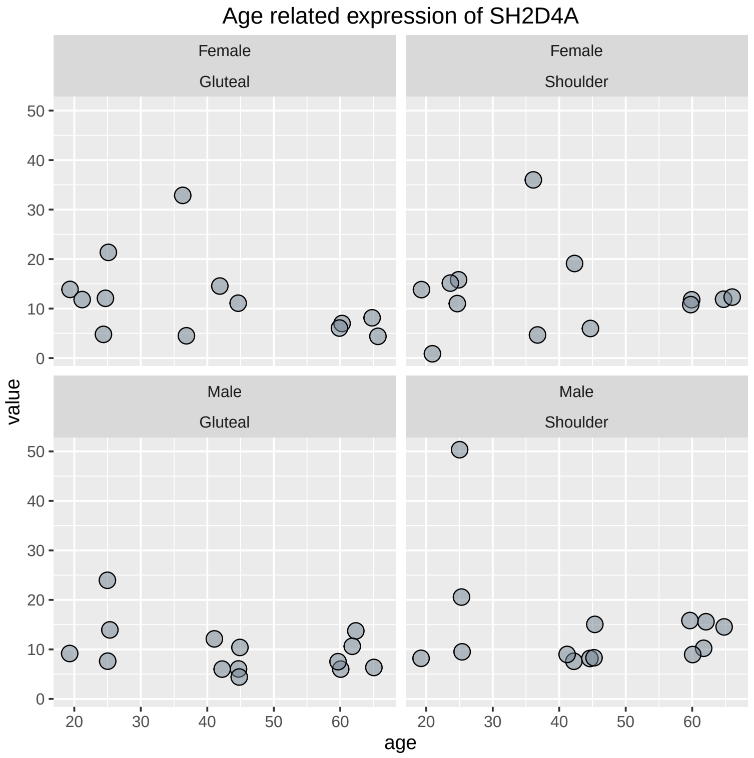

Figure 190: Loess regression for exon align depth

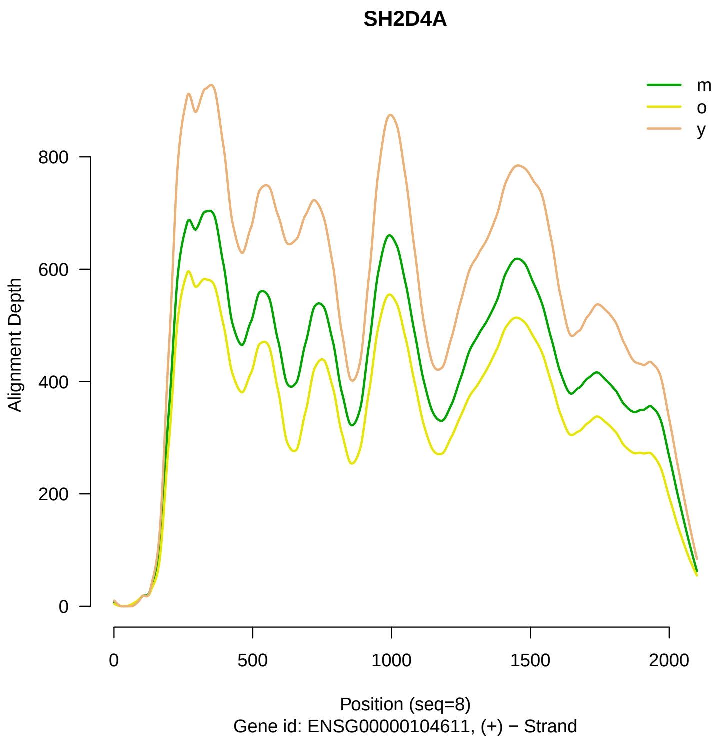

Figure 191: edgeR QLF test based CPM estimates

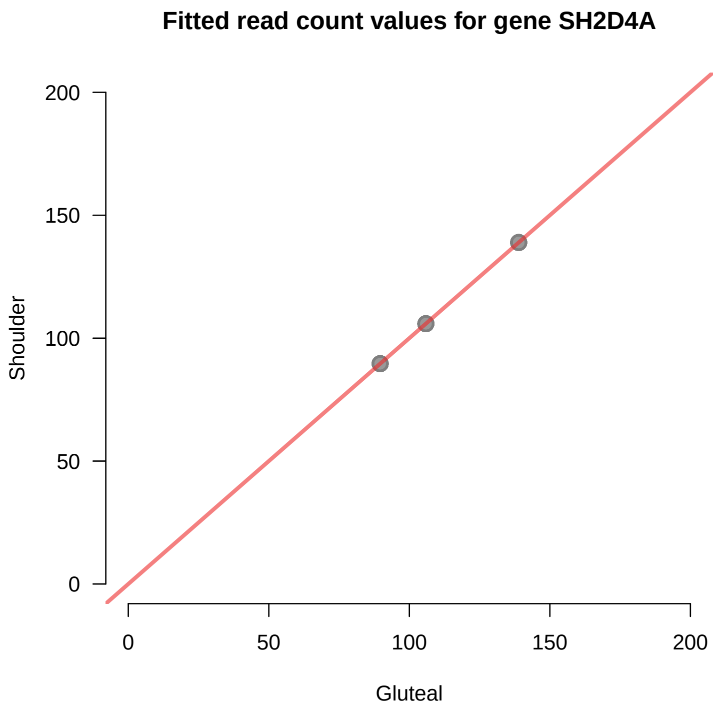

Figure 192: ReadExpSet based genewise CPM estimates

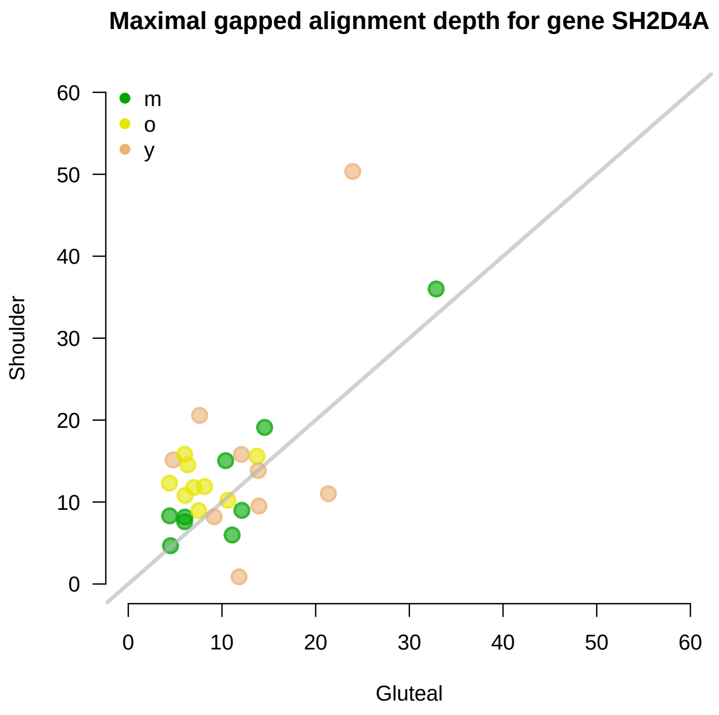

3.33 ARHGAP23P1

Parameter	Value
gene_name	ARHGAP23P1
gene_id	ENSG00000260781
maxald	763
old	down
seqid	16
strand	-
start	33907419
end	33937167
descr	Rho GTPase activating protein 23 pseudogene 1

Table 34: Gene identifcation

Gene expression estimates for Age

Influence of UV exposition on gene expression

Figure 193: Gene expression estimates based on CPM (SummarizeOveraps)

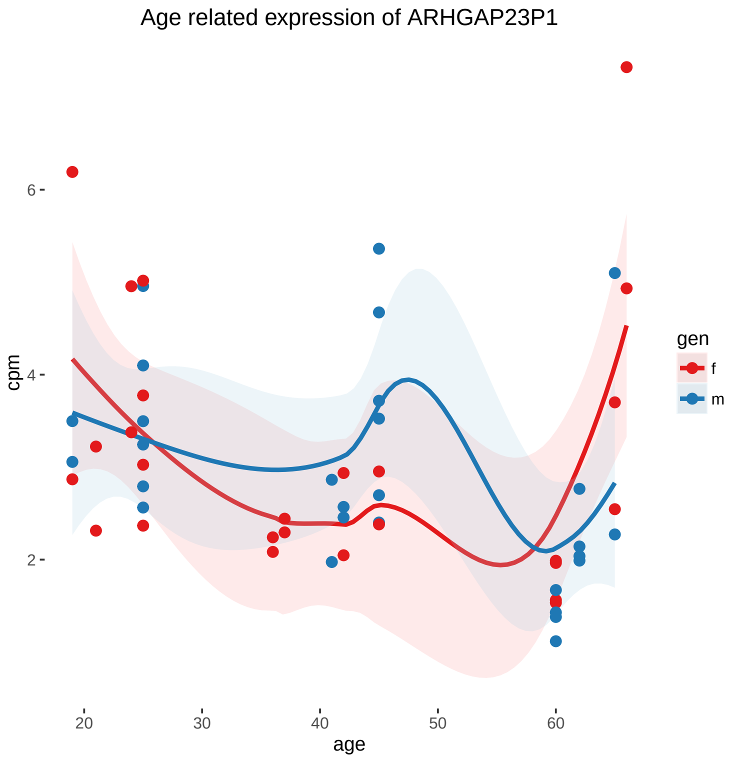

Figure 194: edgeR QLF test based CPM estimates

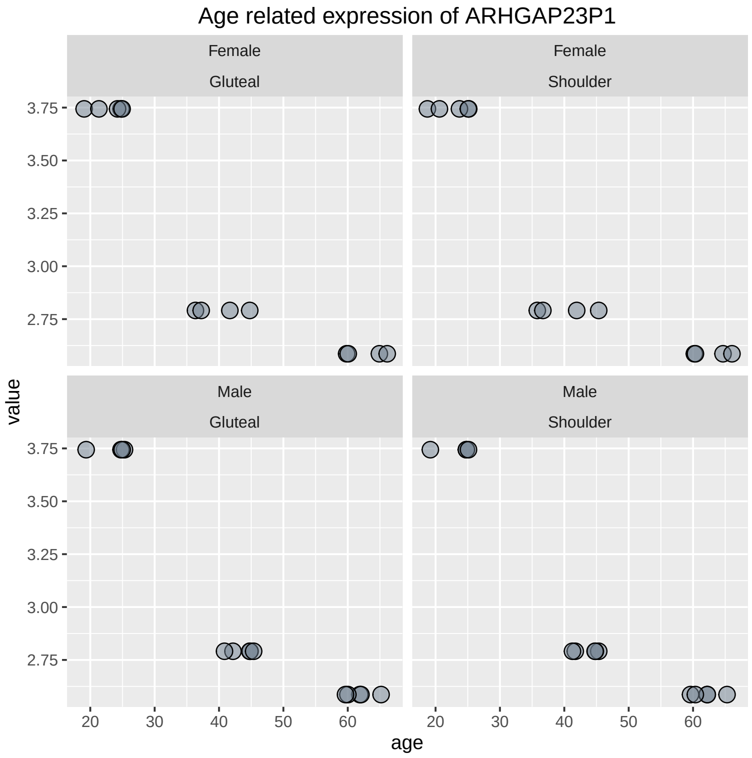

Figure 195: ReadExpSet based genewise CPM estimates

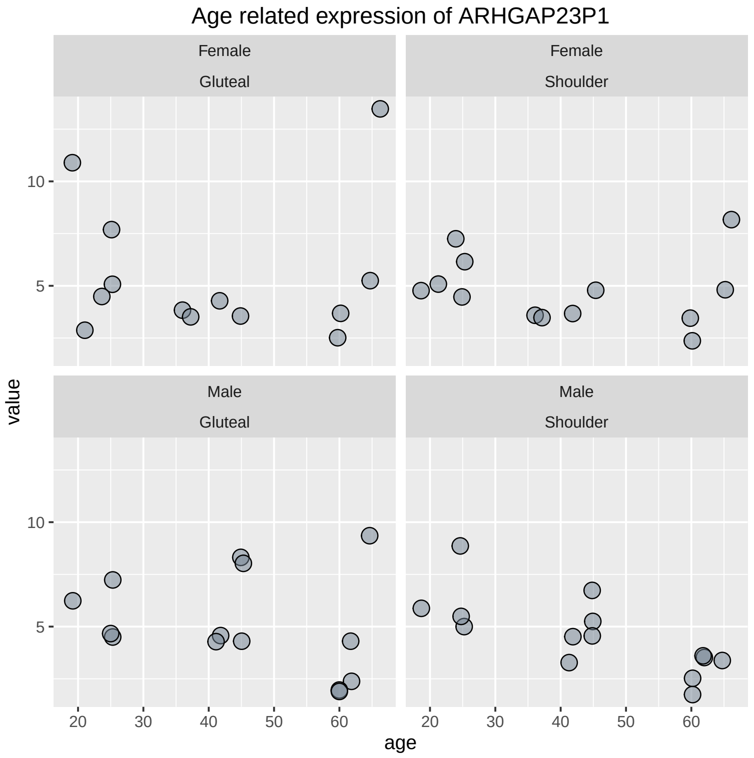

Figure 196: Loess regression for exon align depth

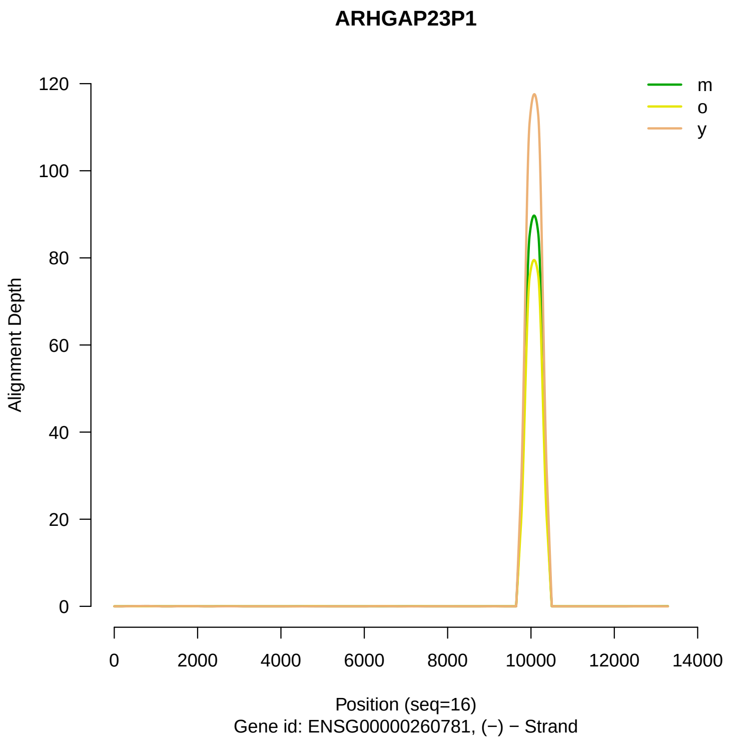

Figure 197: edgeR QLF test based CPM estimates

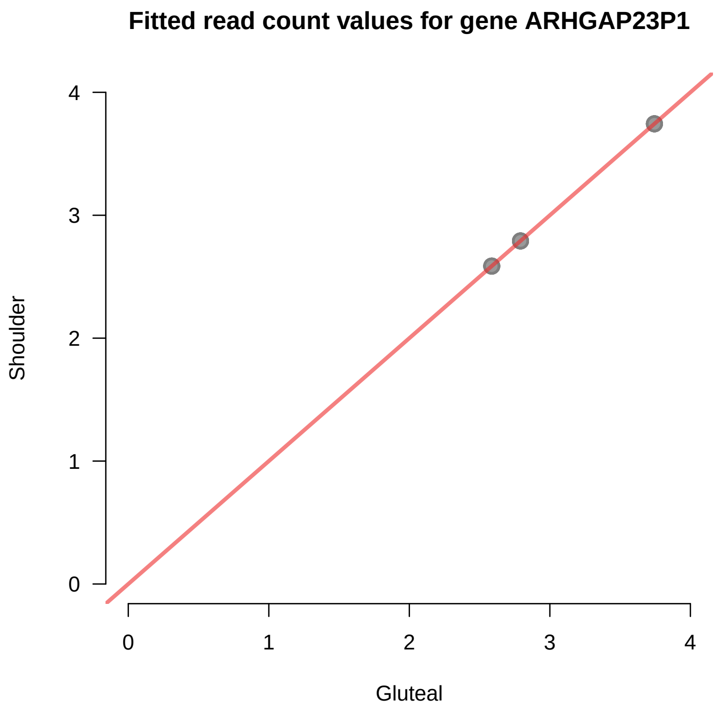

Figure 198: ReadExpSet based genewise CPM estimates

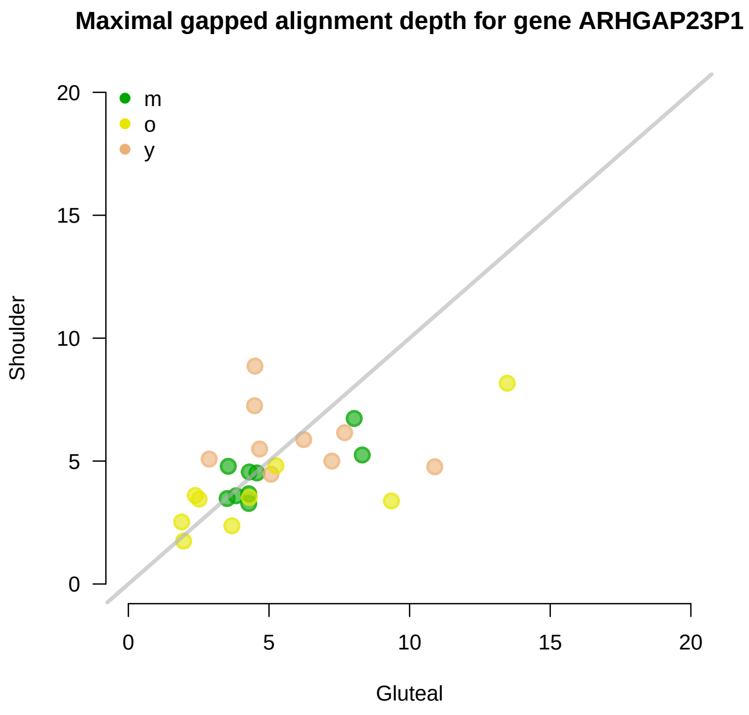

3.34 SERTAD1

Parameter	Value
gene_name	SERTAD1
gene_id	ENSG00000197019
maxald	2018
old	down
seqid	19
strand	-
start	40421592
end	40426025
descr	SERTA domain containing 1

Table 35: Gene identifcation

Gene expression estimates for Age

Influence of UV exposition on gene expression

Figure 199: Gene expression estimates based on CPM (SummarizeOveraps)

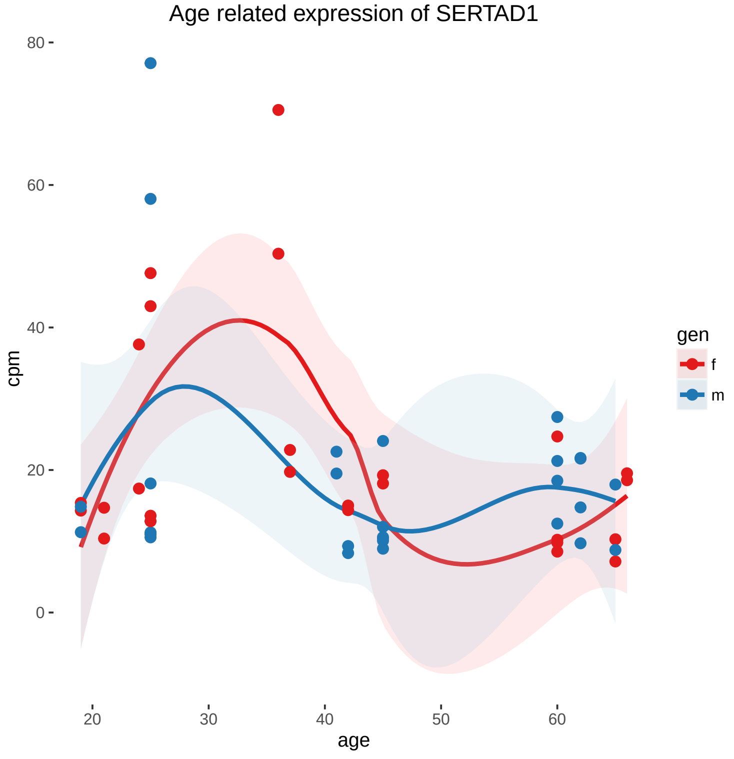

Figure 200: edgeR QLF test based CPM estimates

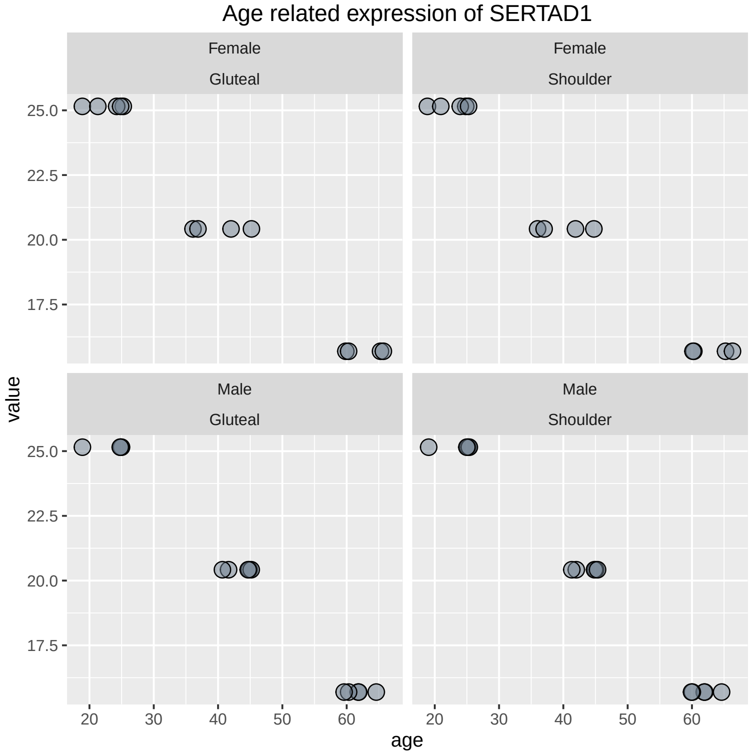

Figure 201: ReadExpSet based genewise CPM estimates

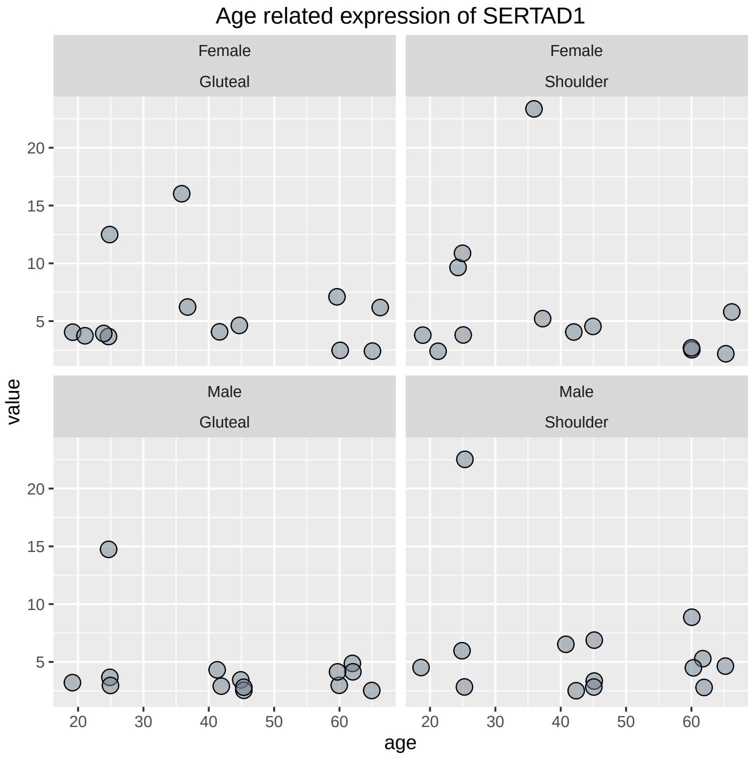

Figure 202: Loess regression for exon align depth

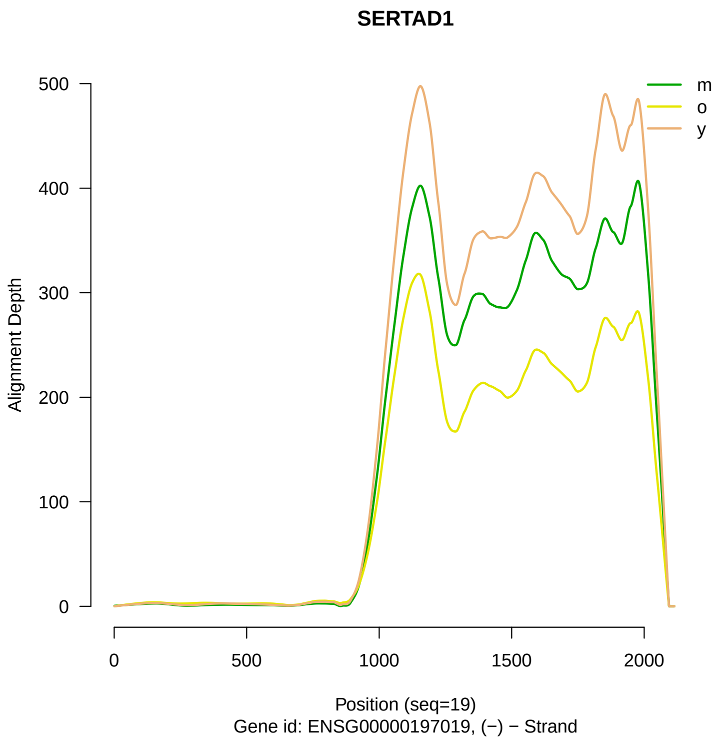

Figure 203: edgeR QLF test based CPM estimates

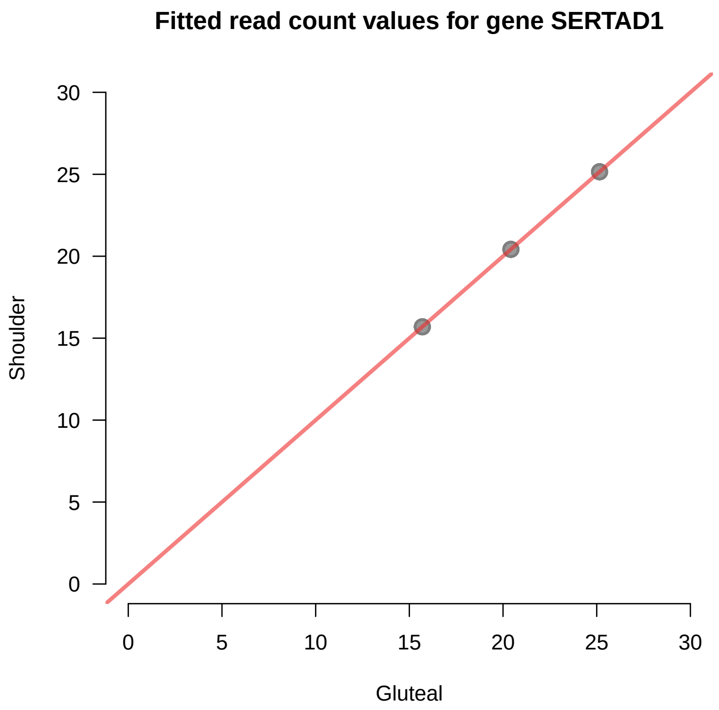

Figure 204: ReadExpSet based genewise CPM estimates

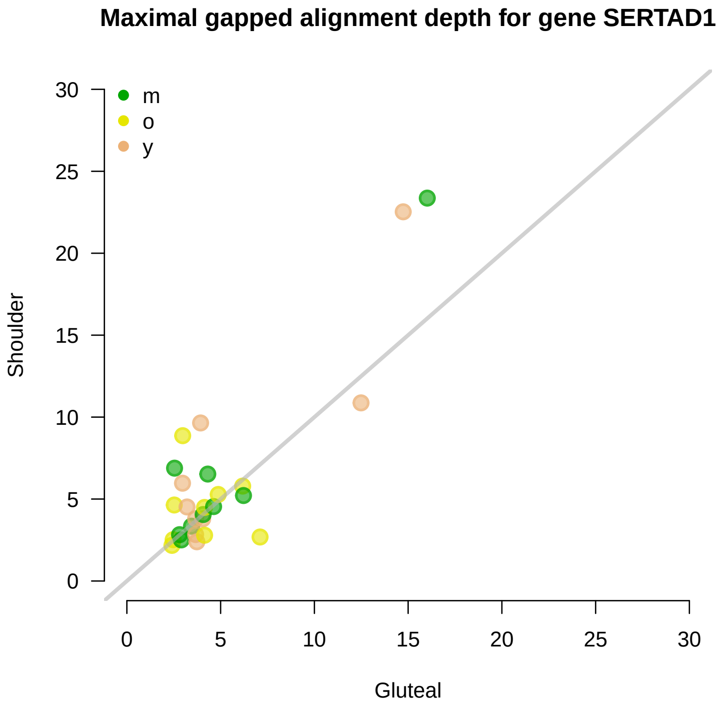

3.35 FGF13

Parameter	Value
gene_name	FGF13
gene_id	ENSG00000129682
maxald	345
old	up
seqid	X
strand	-
start	138614731
end	139222777
descr	fibroblast growth factor 13

Table 36: Gene identifcation

Gene expression estimates for Age

Influence of UV exposition on gene expression

Figure 205: Gene expression estimates based on CPM (SummarizeOveraps)

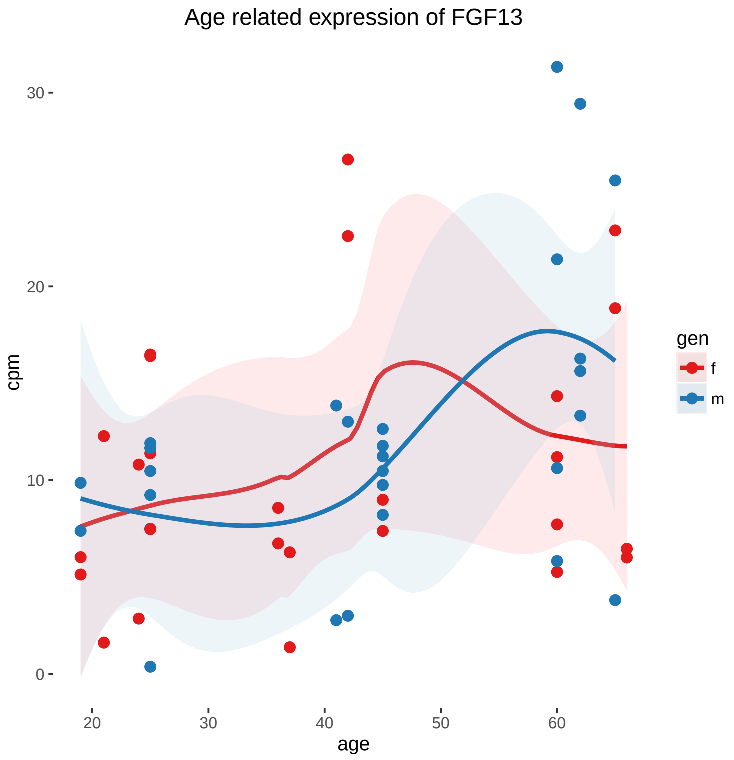

Figure 206: edgeR QLF test based CPM estimates

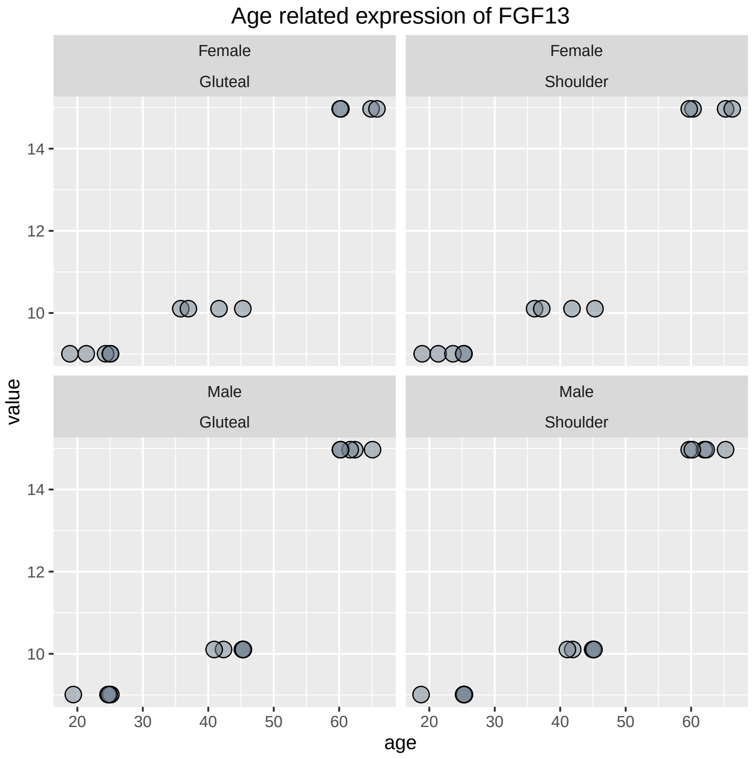

Figure 207: ReadExpSet based genewise CPM estimates

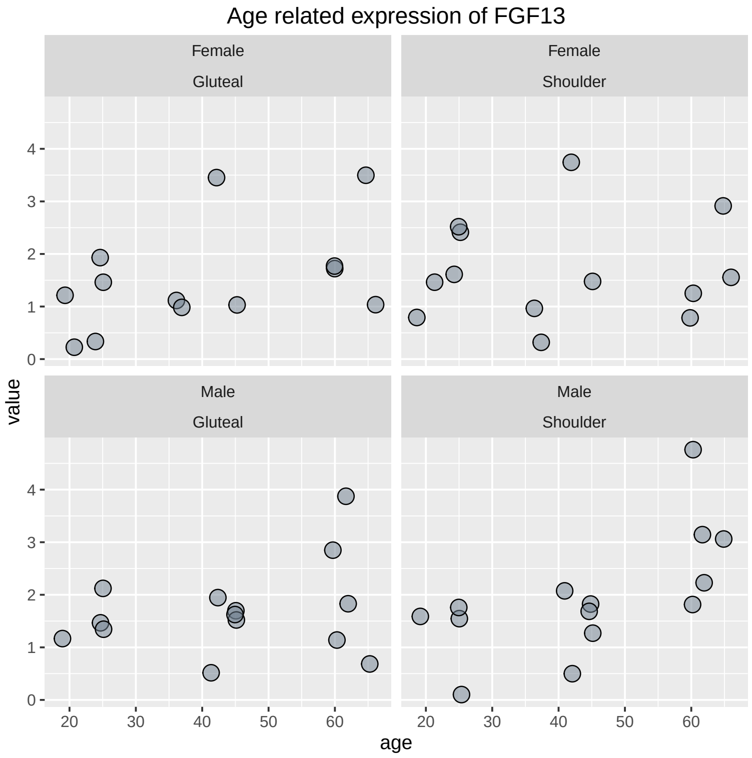

Figure 208: Loess regression for exon align depth

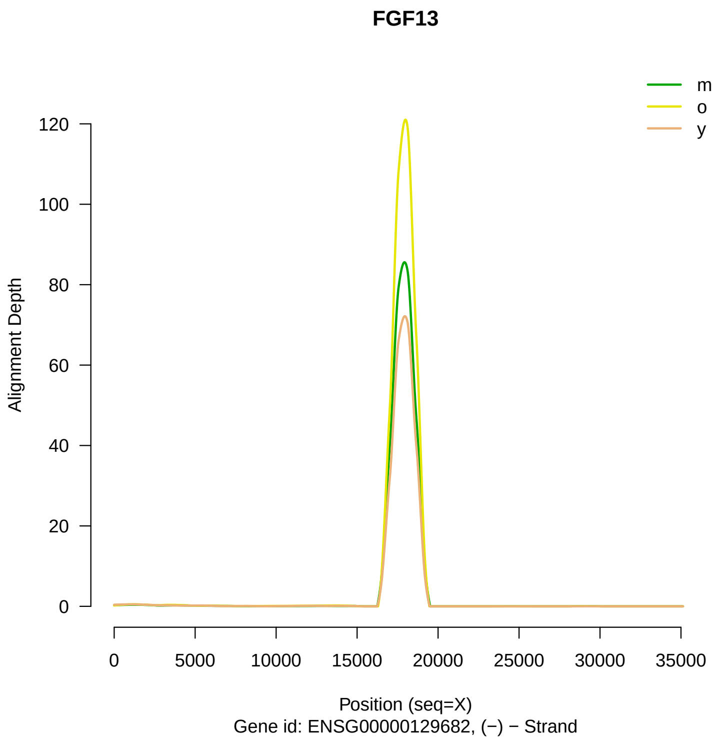

Figure 209: edgeR QLF test based CPM estimates

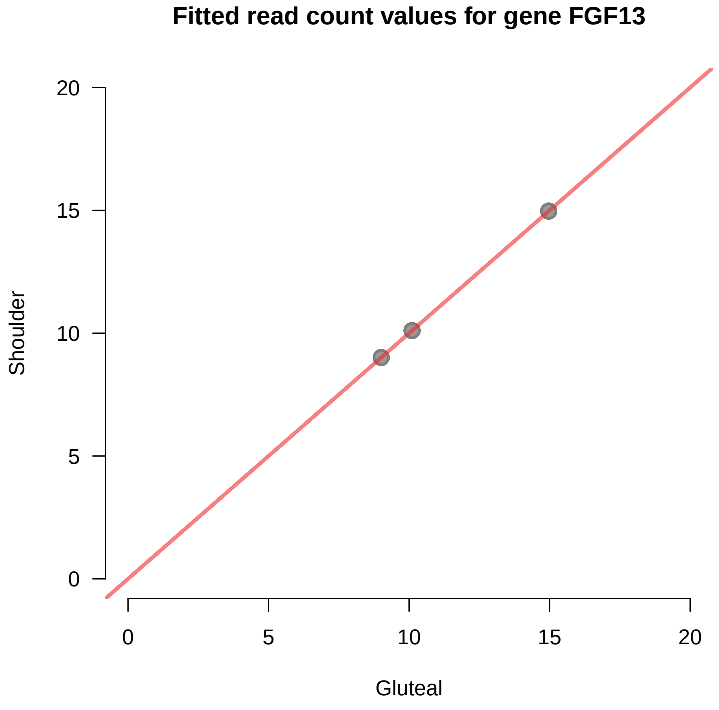

Figure 210: ReadExpSet based genewise CPM estimates

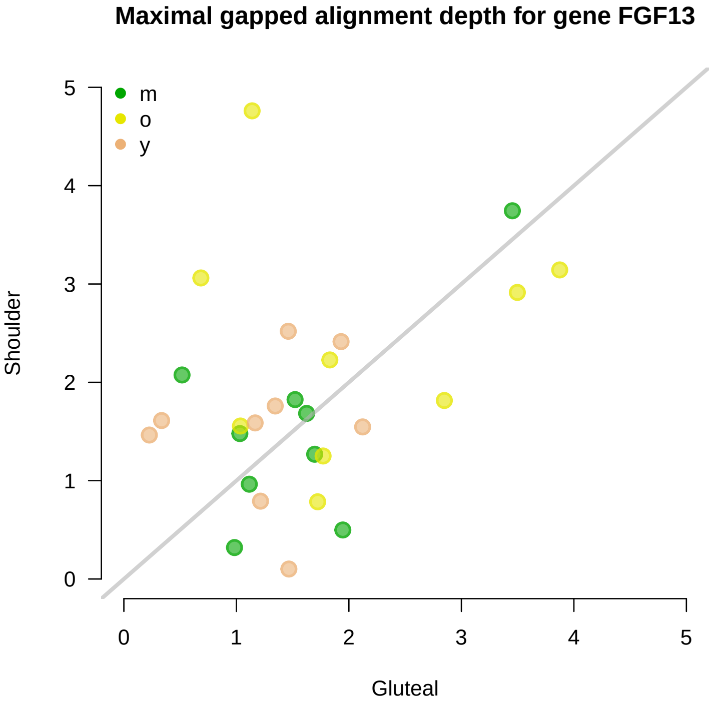

3.36 EHD1

Parameter	Value
gene_name	EHD1
gene_id	ENSG00000110047
maxald	2871
old	down
seqid	11
strand	-
start	64851642
end	64888296
descr	EH-domain containing 1

Table 37: Gene identifcation

Gene expression estimates for Age

Influence of UV exposition on gene expression

Figure 211: Gene expression estimates based on CPM (SummarizeOveraps)

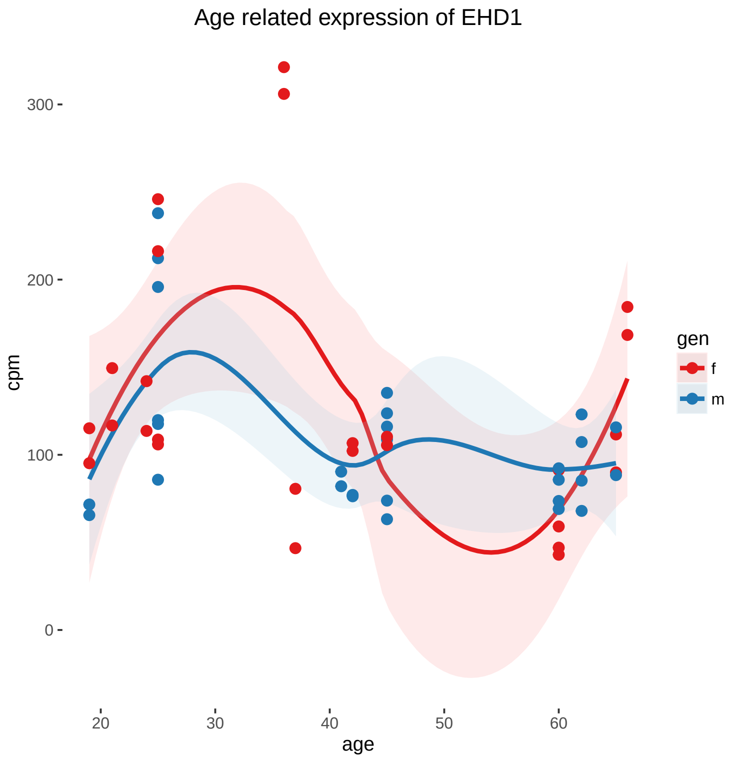

Figure 212: edgeR QLF test based CPM estimates

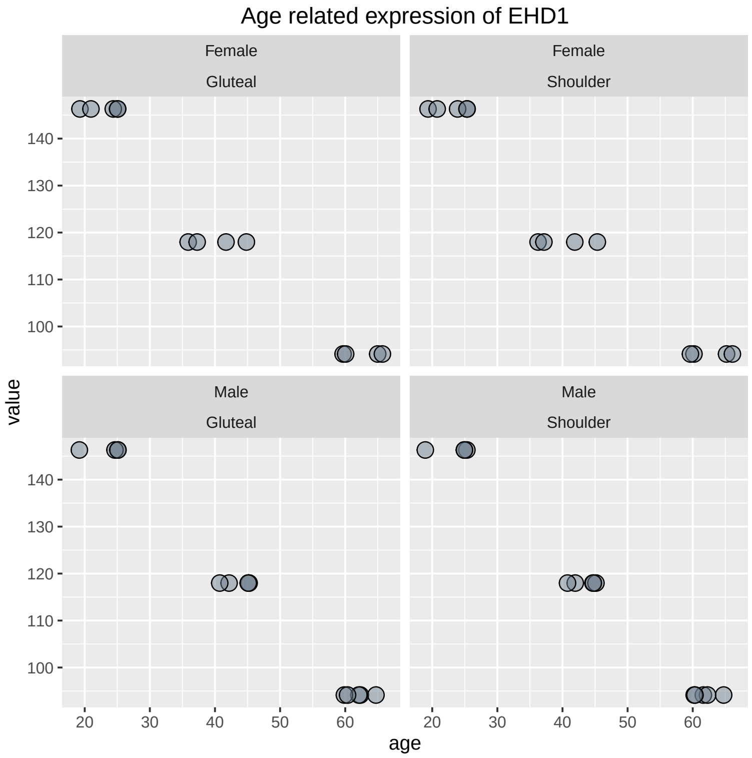

Figure 213: ReadExpSet based genewise CPM estimates

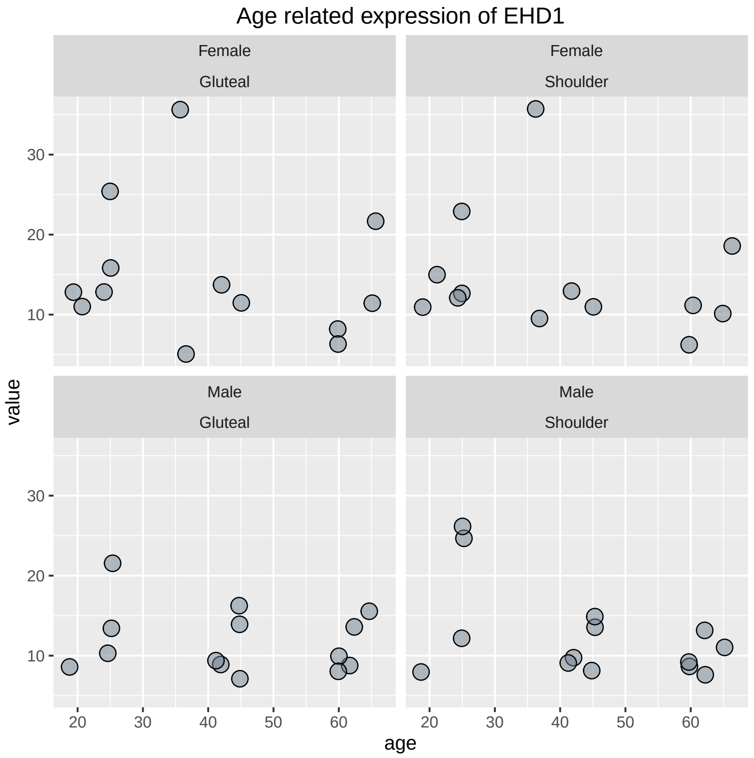

Figure 214: Loess regression for exon align depth

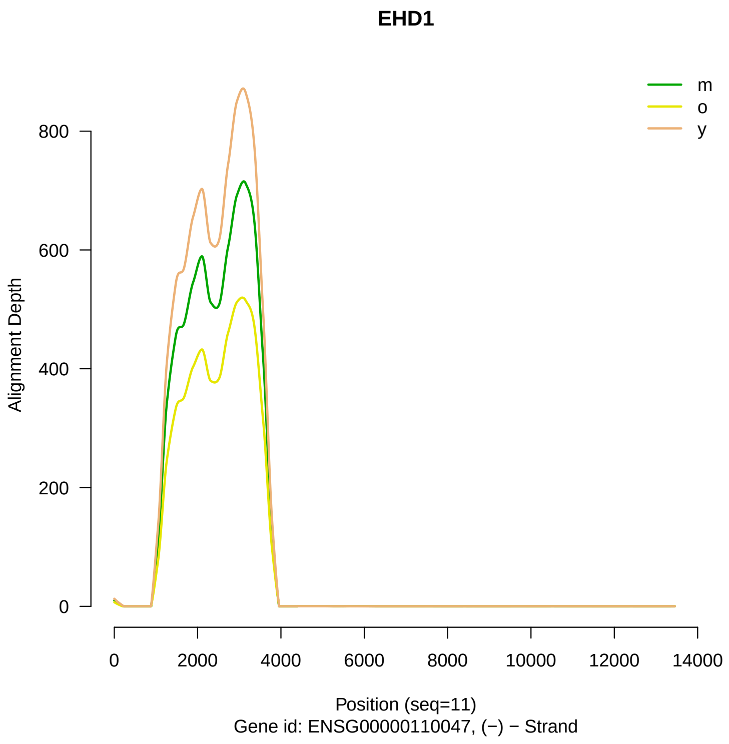

Figure 215: edgeR QLF test based CPM estimates

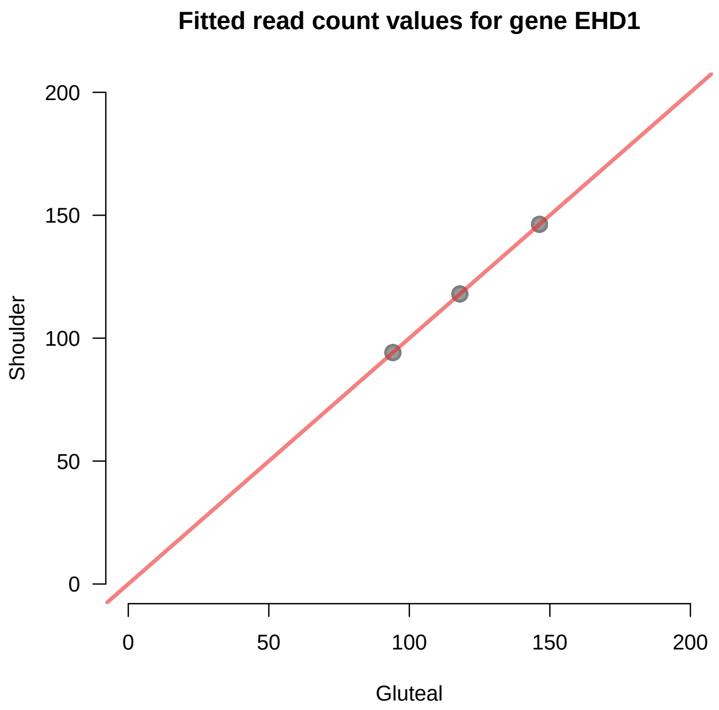

Figure 216: ReadExpSet based genewise CPM estimates

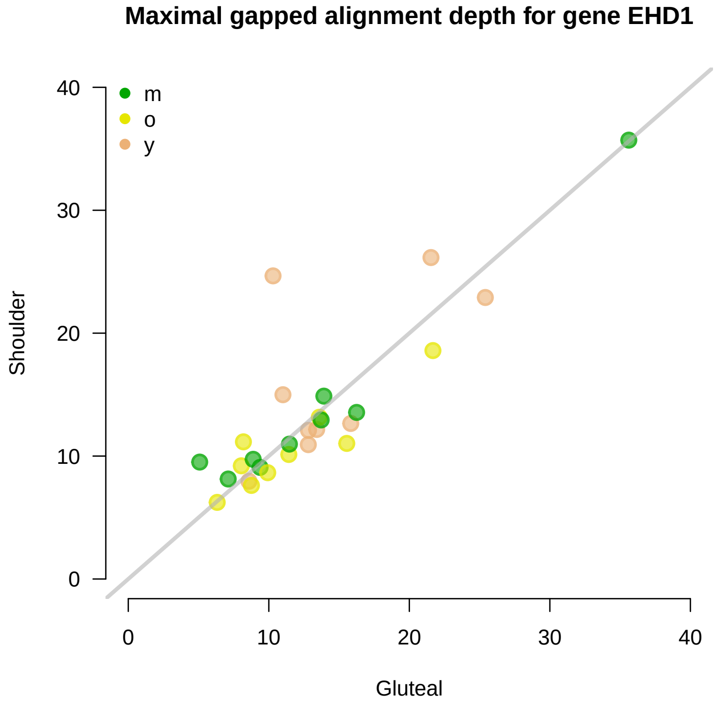

3.37 USP41

Parameter	Value
gene_name	USP41
gene_id	ENSG00000161133
maxald	155
old	up
seqid	22
strand	-
start	20350578
end	20390758
descr	ubiquitin specific peptidase 41

Table 38: Gene identifcation

Gene expression estimates for Age

Influence of UV exposition on gene expression

Figure 217: Gene expression estimates based on CPM (SummarizeOveraps)

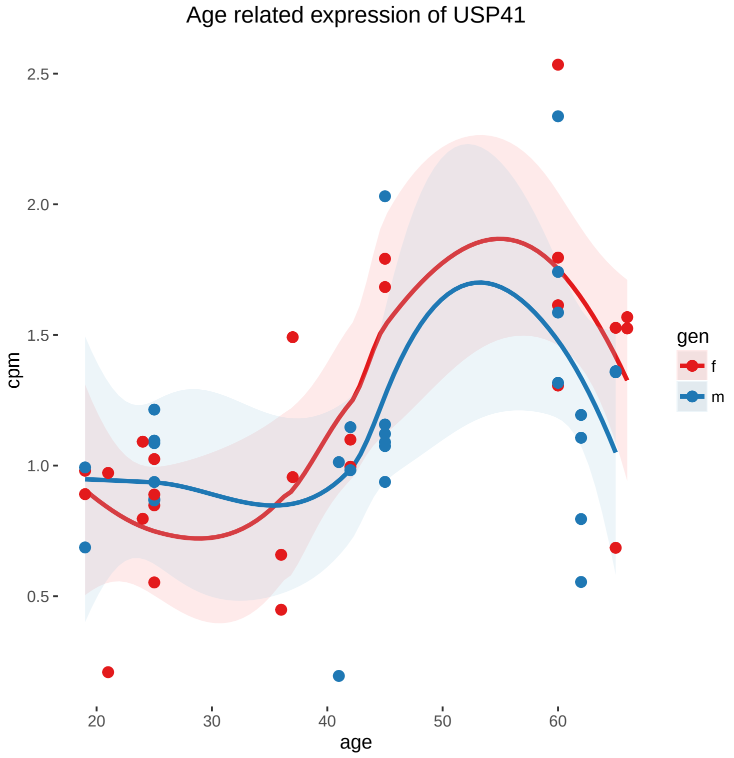

Figure 218: edgeR QLF test based CPM estimates

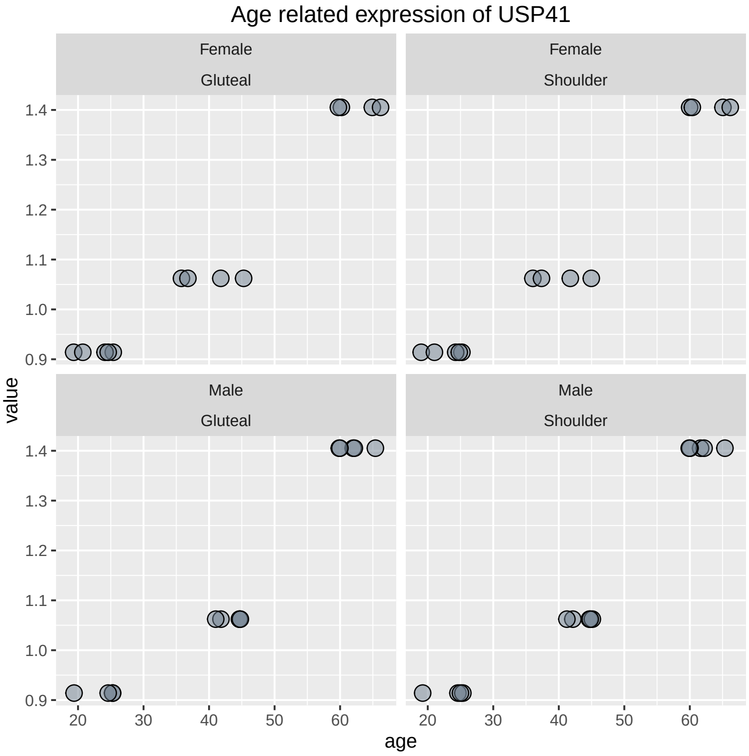

Figure 219: ReadExpSet based genewise CPM estimates

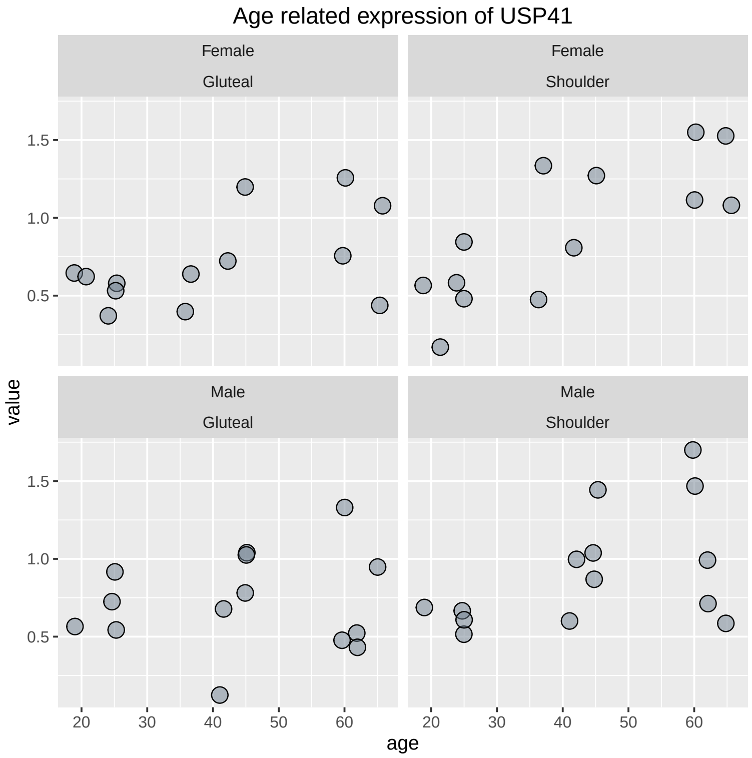

Figure 220: Loess regression for exon align depth

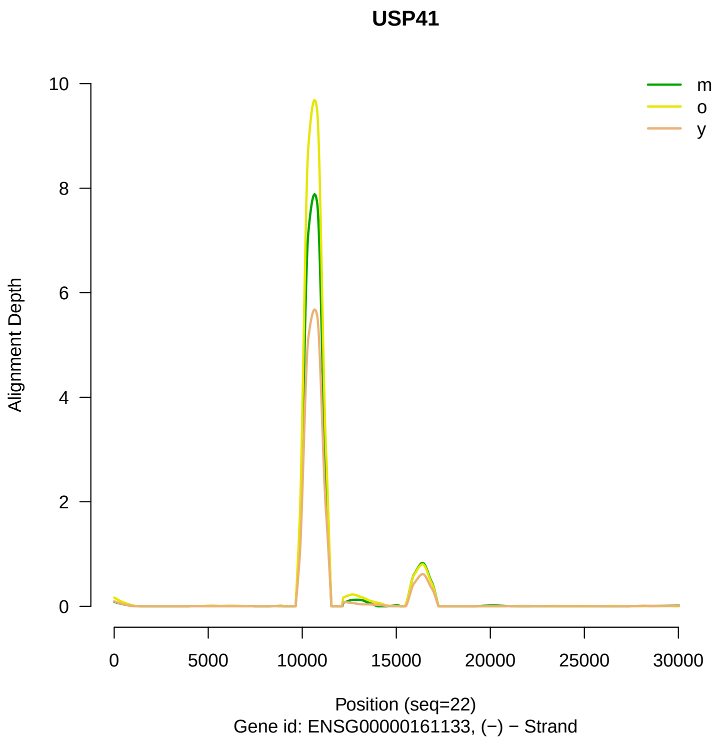

Figure 221: edgeR QLF test based CPM estimates

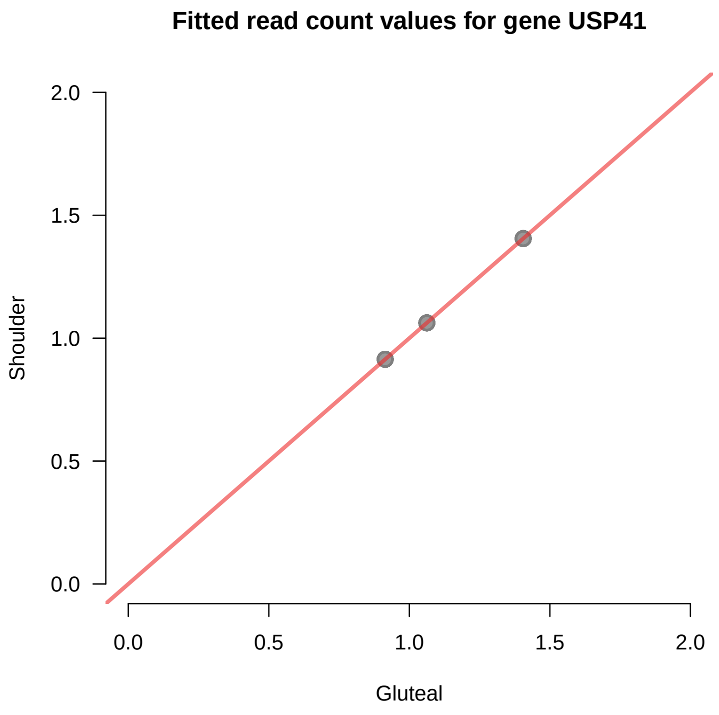

Figure 222: ReadExpSet based genewise CPM estimates

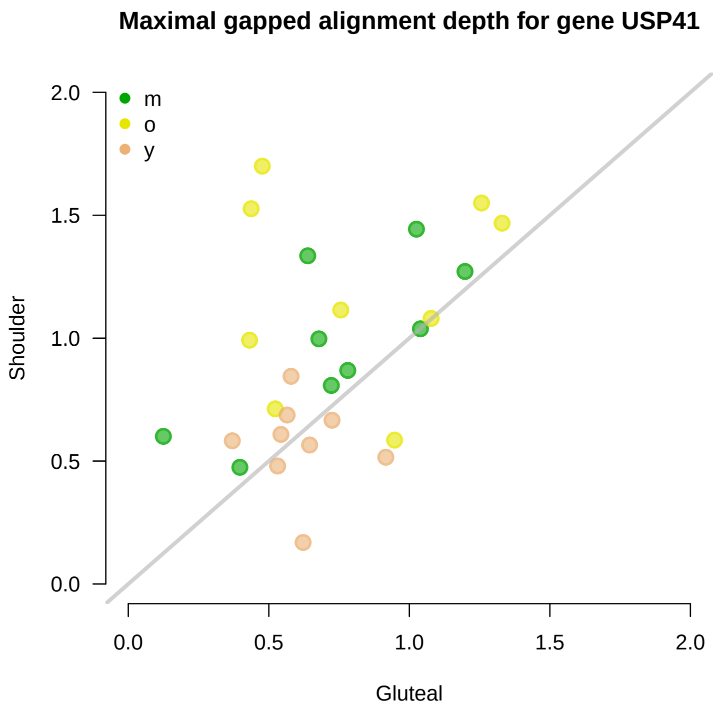

3.38 ACSS3

Parameter	Value
gene_name	ACSS3
gene_id	ENSG00000111058
maxald	201
old	down
seqid	12
strand	+
start	80936414
end	81261205
descr	acyl-CoA synthetase short-chain family member 3

Table 39: Gene identifcation

Gene expression estimates for Age

Influence of UV exposition on gene expression

Figure 223: Gene expression estimates based on CPM (SummarizeOveraps)

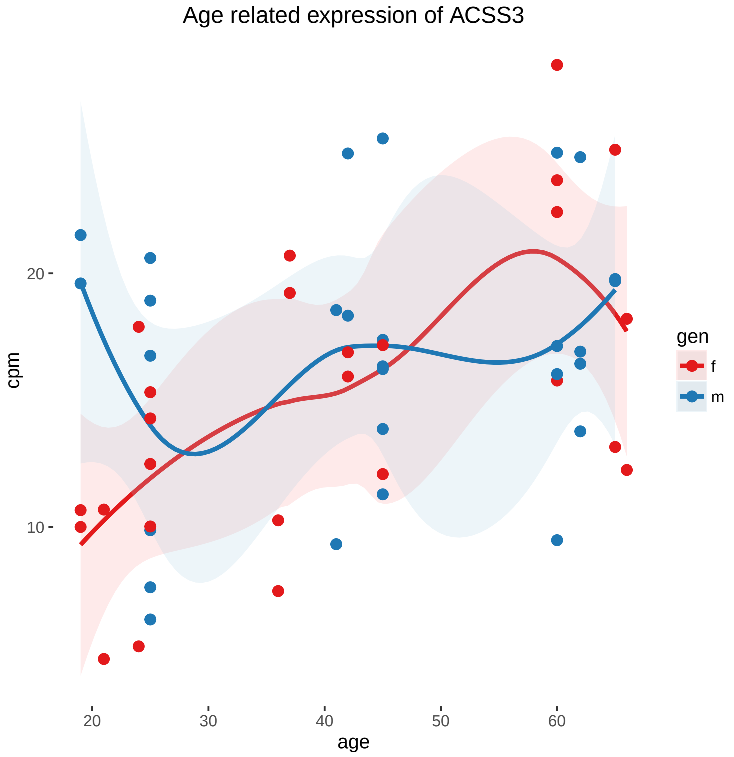

Figure 224: edgeR QLF test based CPM estimates

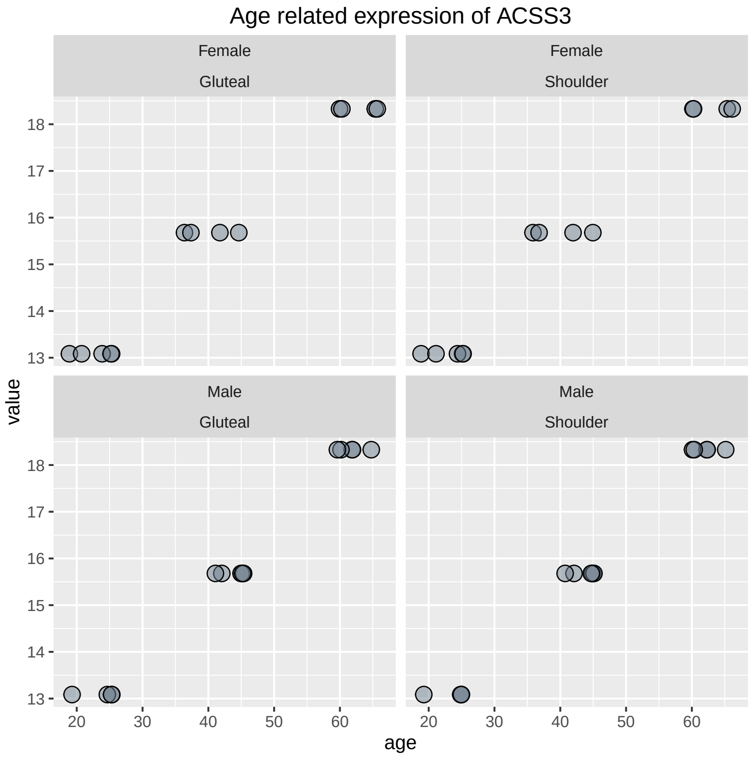

Figure 225: ReadExpSet based genewise CPM estimates

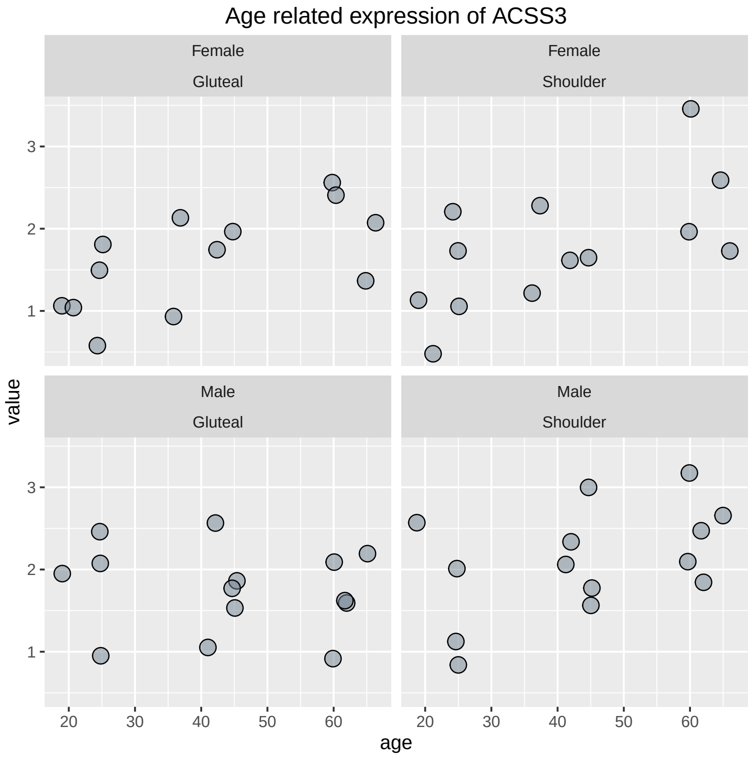

Figure 226: Loess regression for exon align depth

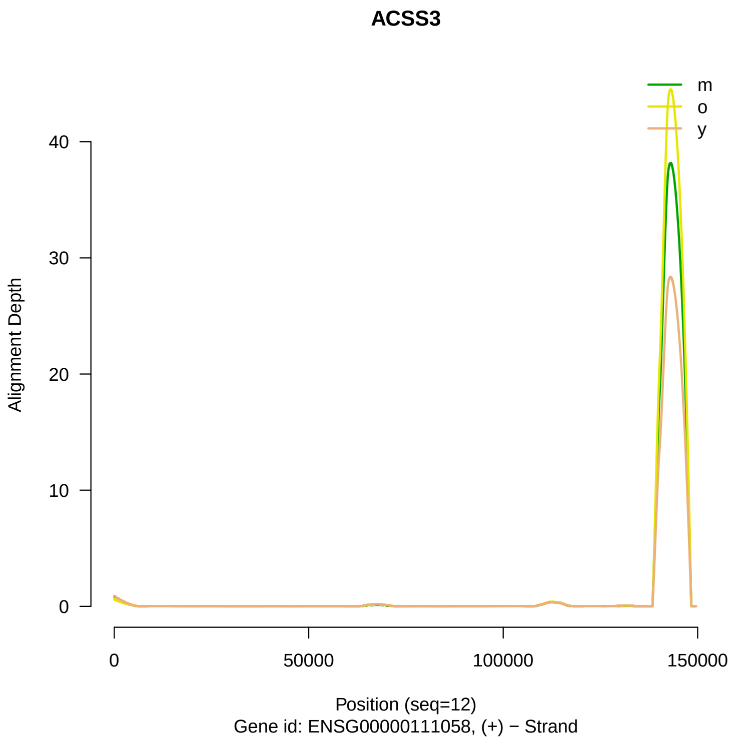

Figure 227: edgeR QLF test based CPM estimates

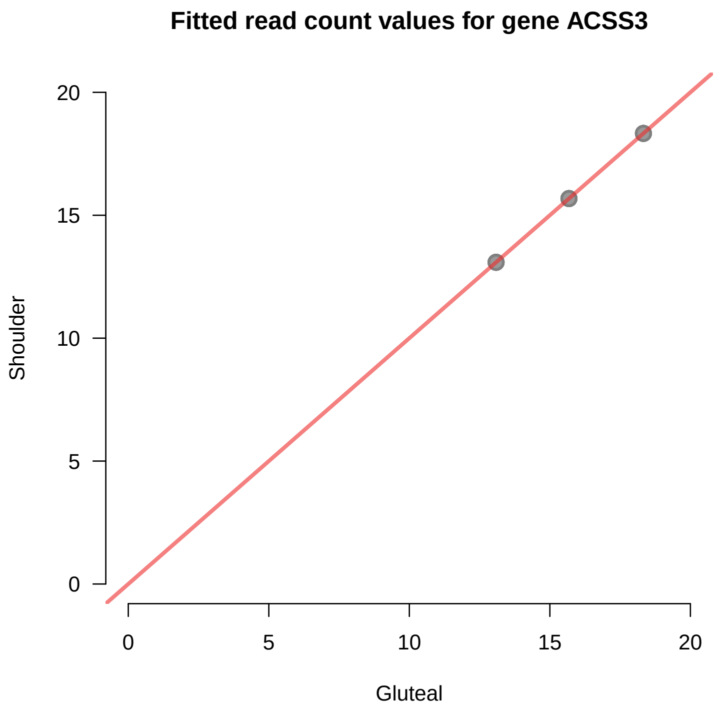

Figure 228: ReadExpSet based genewise CPM estimates

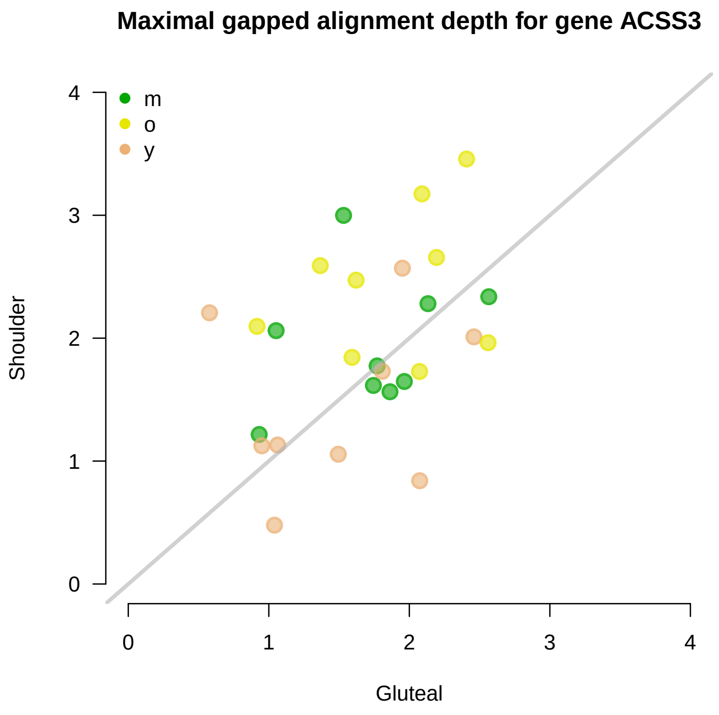

3.39 BACE2

Parameter	Value
gene_name	BACE2
gene_id	ENSG00000182240
maxald	2384
old	up
seqid	21
strand	+
start	41167801
end	41282518
descr	beta-site APP-cleaving enzyme 2

Table 40: Gene identifcation

Gene expression estimates for Age

Influence of UV exposition on gene expression

Figure 229: Gene expression estimates based on CPM (SummarizeOveraps)

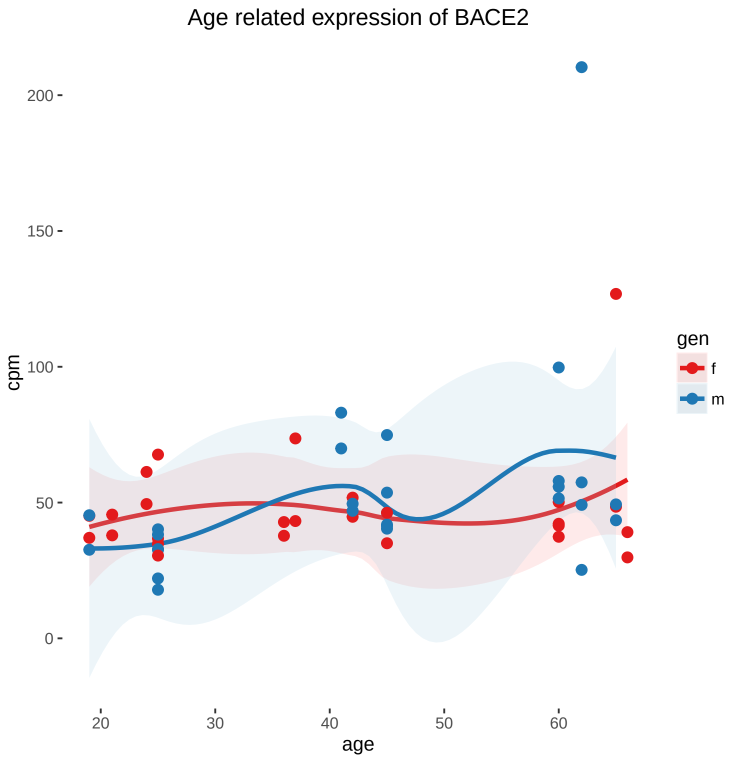

Figure 230: edgeR QLF test based CPM estimates

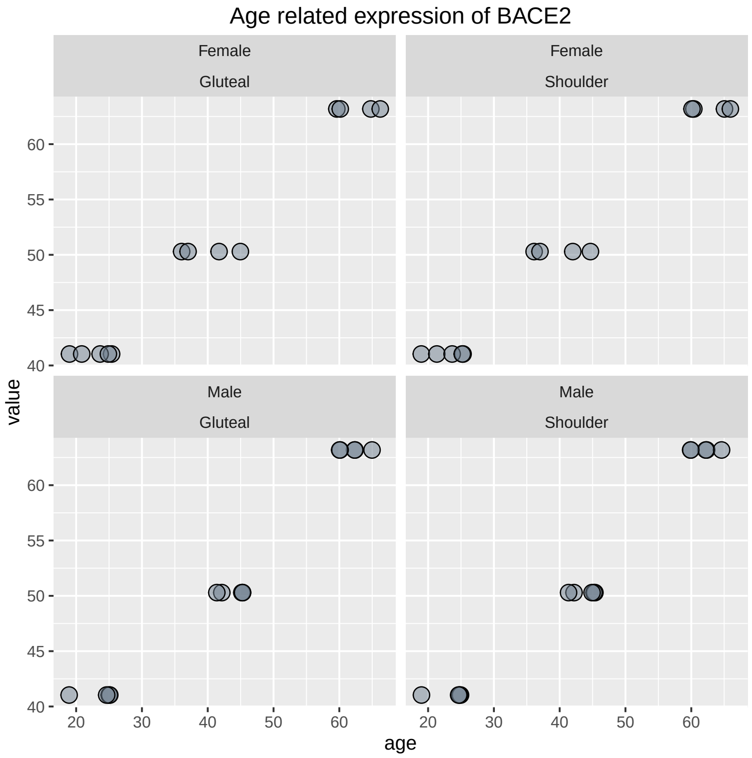

Figure 231: ReadExpSet based genewise CPM estimates

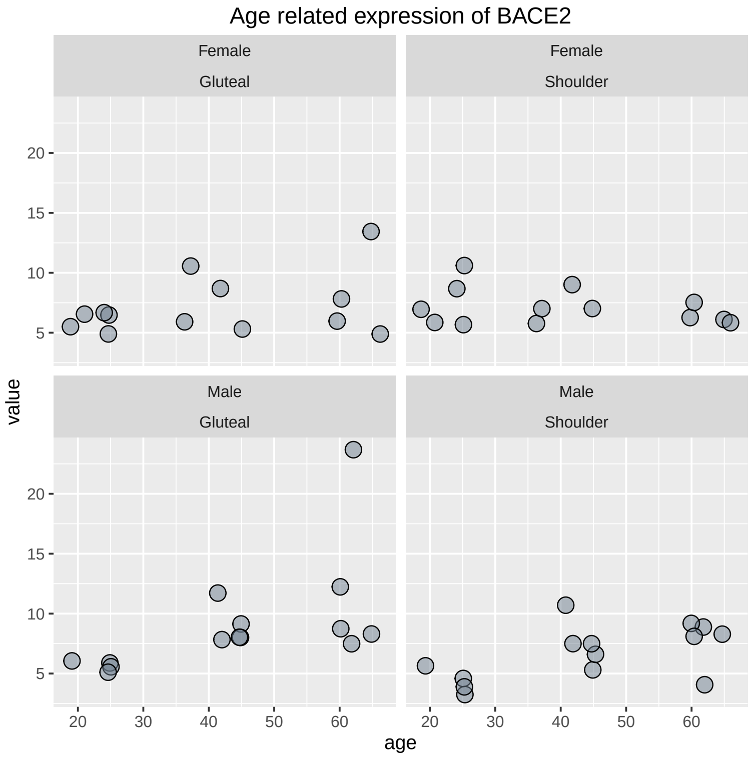

Figure 232: Loess regression for exon align depth

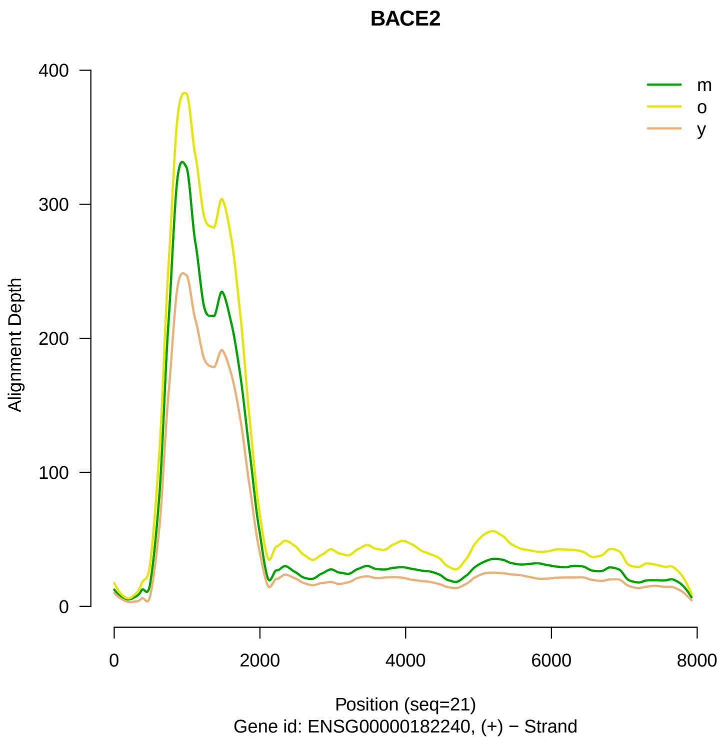

Figure 233: edgeR QLF test based CPM estimates

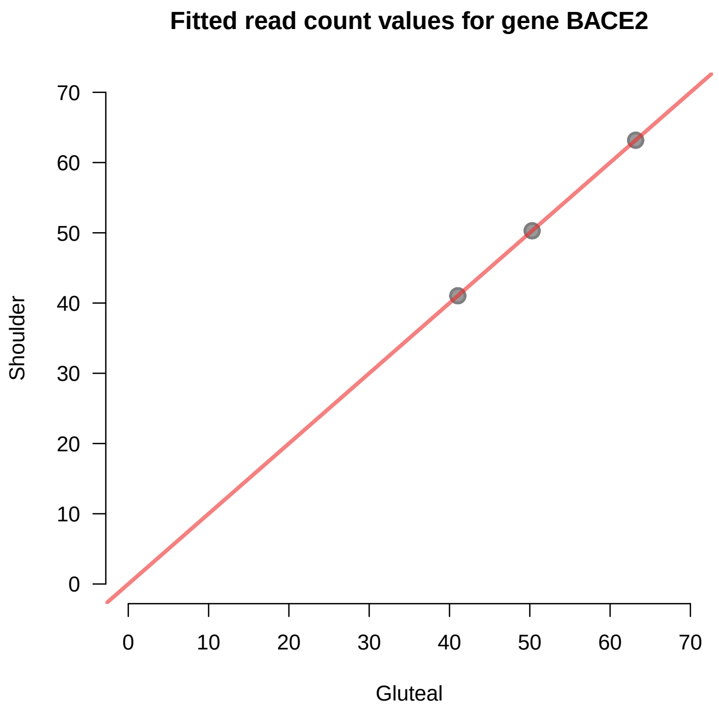

Figure 234: ReadExpSet based genewise CPM estimates

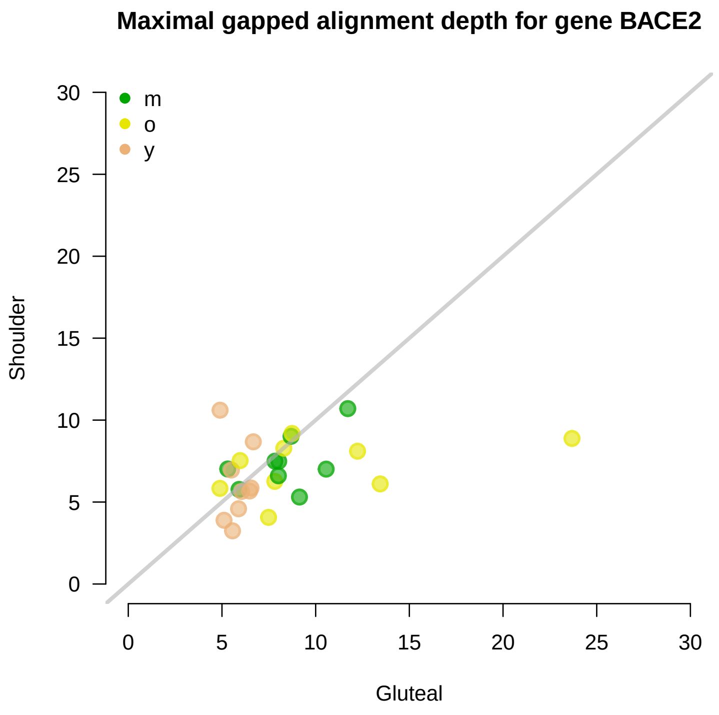

3.40 ADGRL4

Parameter	Value
gene_name	ADGRL4
gene_id	ENSG00000162618
maxald	524
old	up
seqid	1
strand	-
start	78889764
end	79006718
descr	adhesion G protein-coupled receptor L4

Table 41: Gene identifcation

Gene expression estimates for Age

Influence of UV exposition on gene expression

Figure 235: Gene expression estimates based on CPM (SummarizeOveraps)

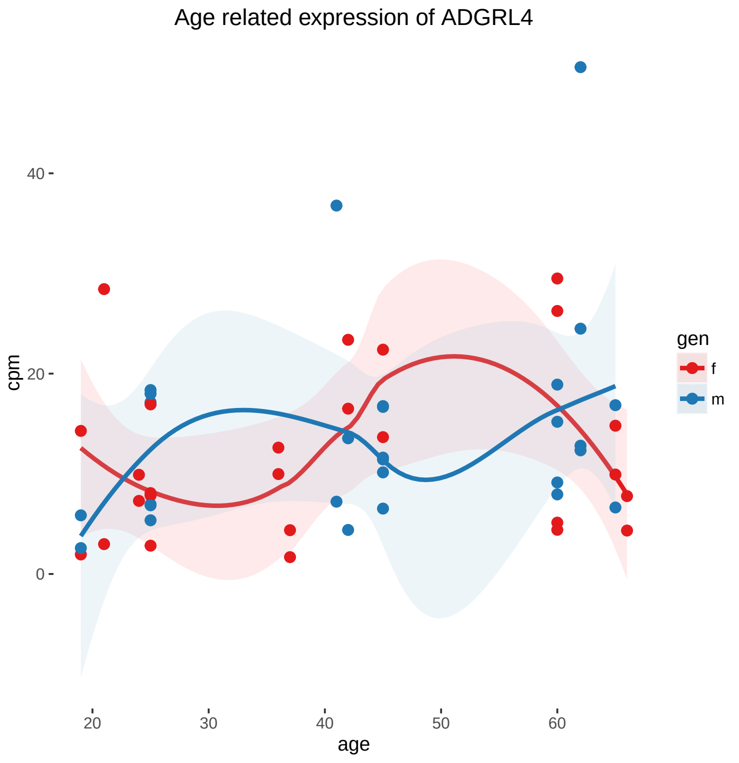

Figure 236: edgeR QLF test based CPM estimates

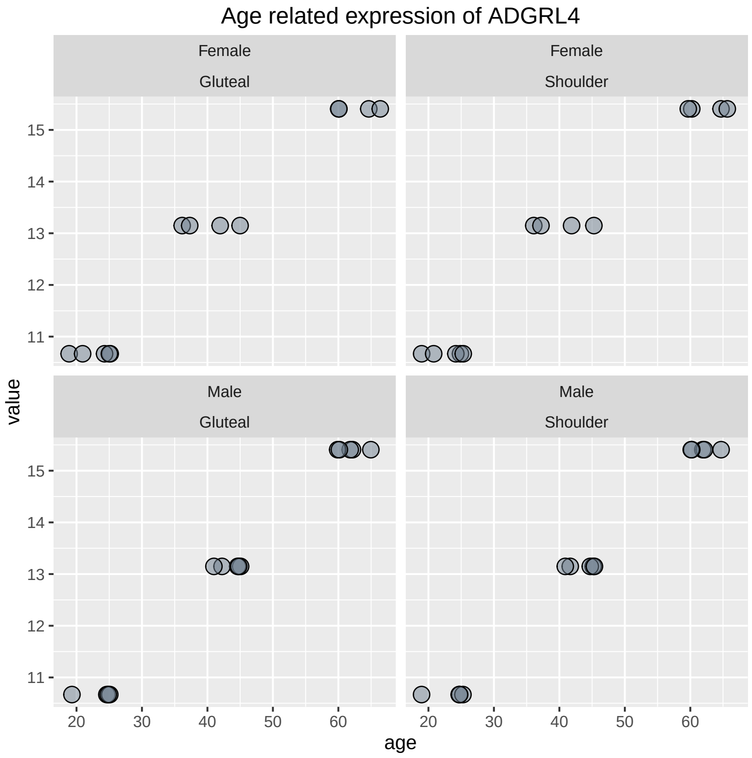

Figure 237: ReadExpSet based genewise CPM estimates

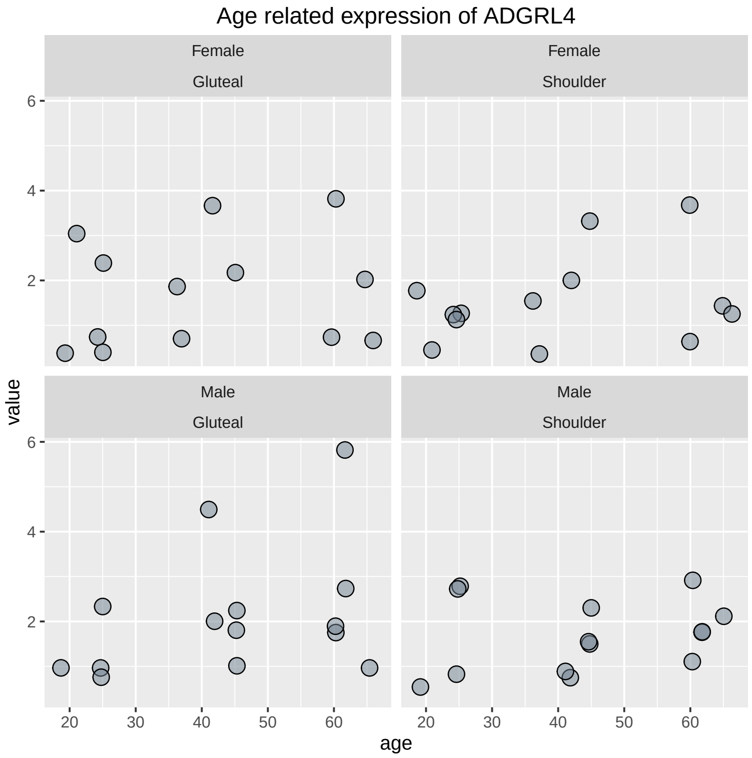

Figure 238: Loess regression for exon align depth

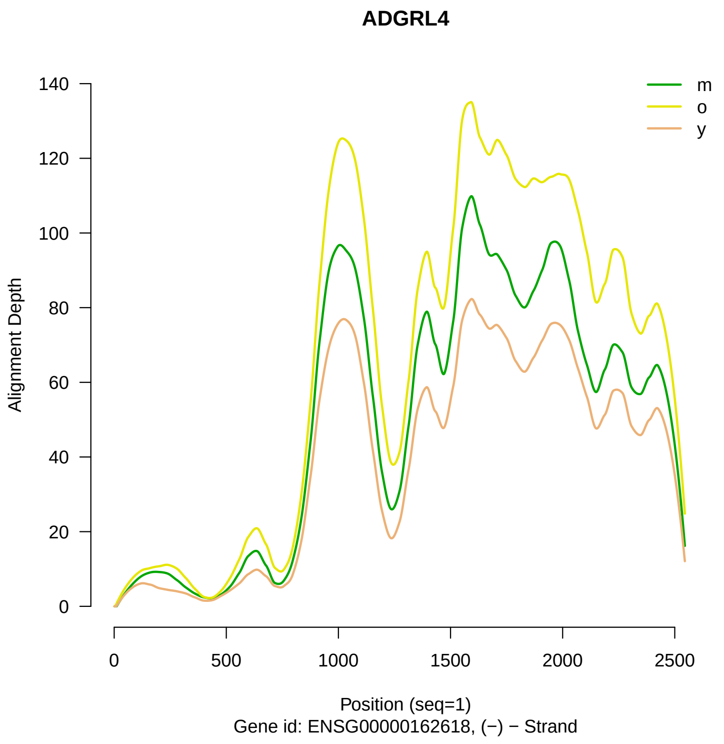

Figure 239: edgeR QLF test based CPM estimates

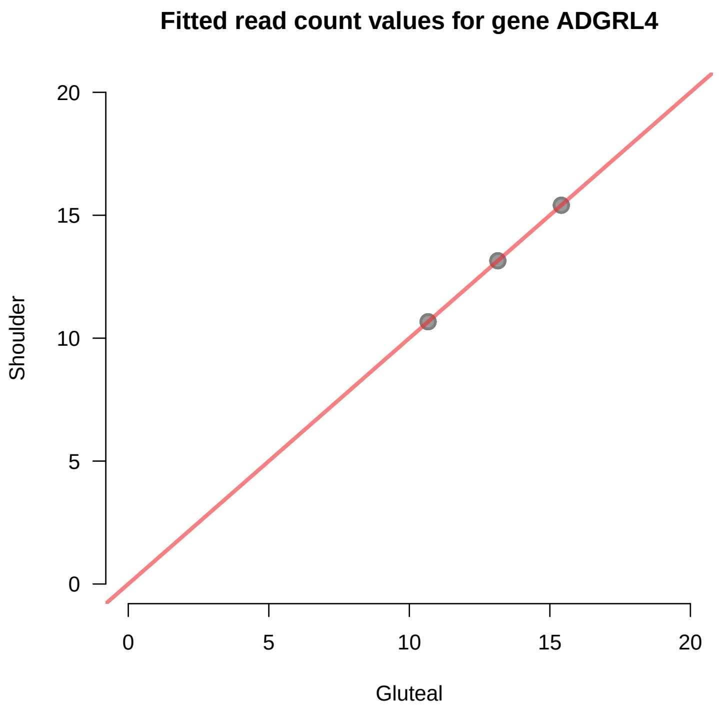

Figure 240: ReadExpSet based genewise CPM estimates

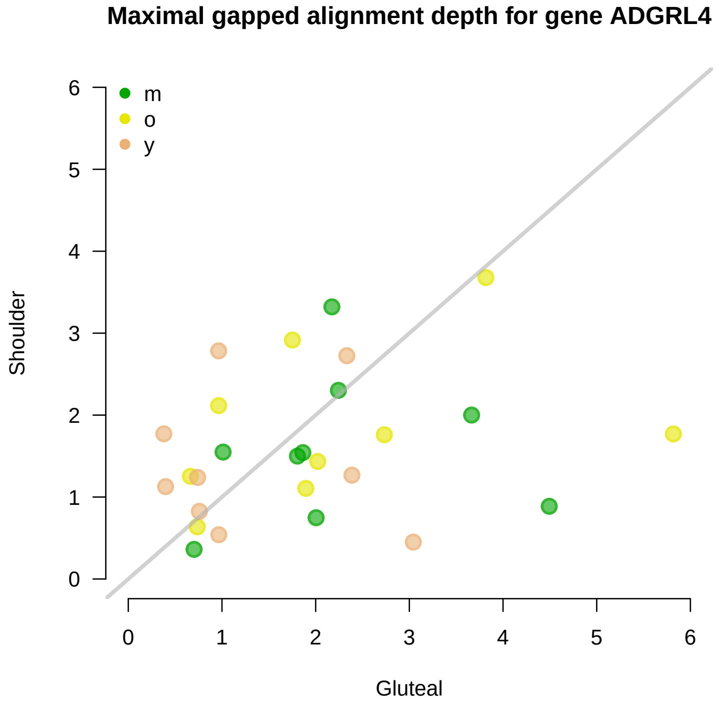

3.41 ROBO1

Parameter	Value
gene_name	ROBO1
gene_id	ENSG00000169855
maxald	826
old	up
seqid	3
strand	-
start	78597240
end	79767815
descr	roundabout guidance receptor 1

Table 42: Gene identifcation

Gene expression estimates for Age

Influence of UV exposition on gene expression

Figure 241: Gene expression estimates based on CPM (SummarizeOveraps)

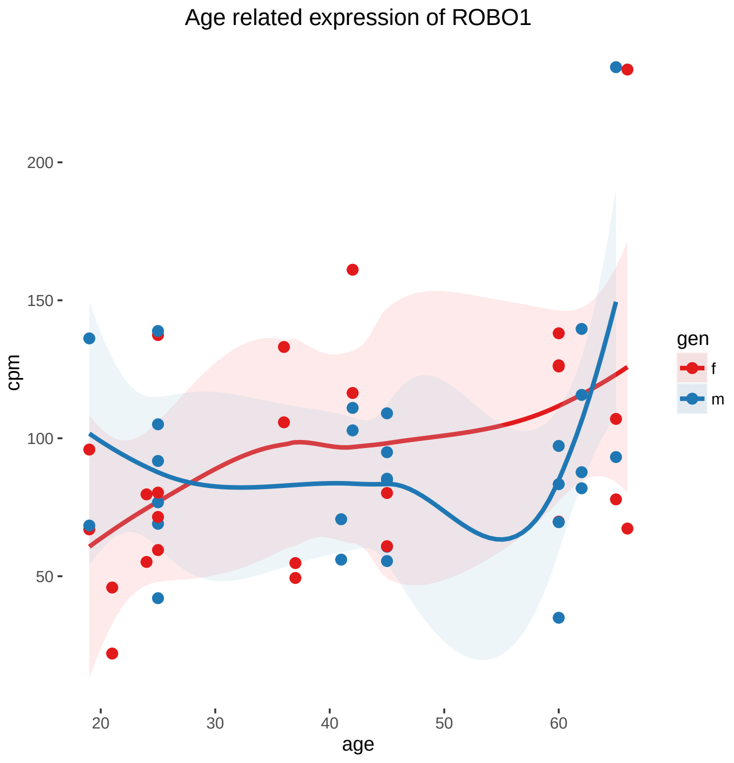

Figure 242: edgeR QLF test based CPM estimates

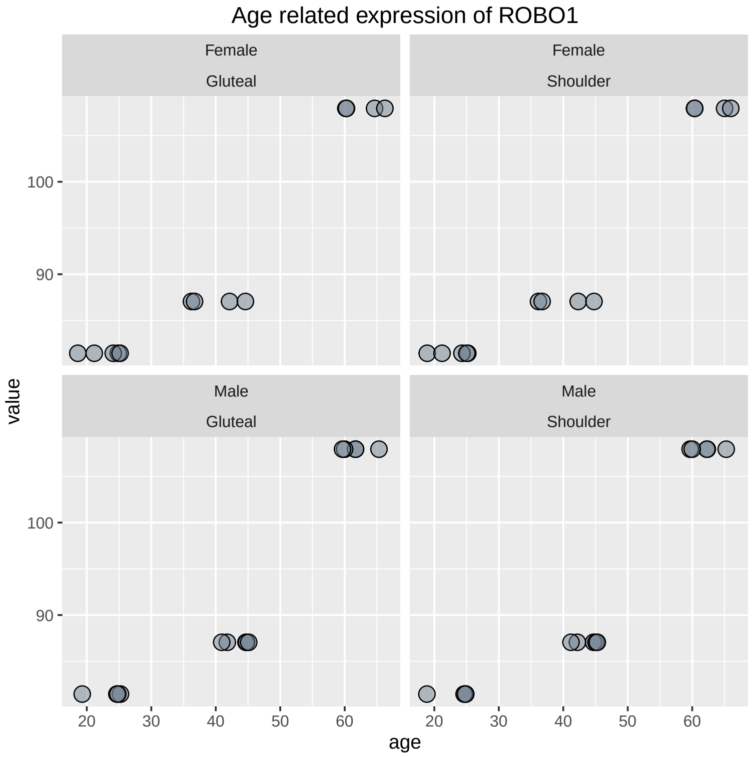

Figure 243: ReadExpSet based genewise CPM estimates

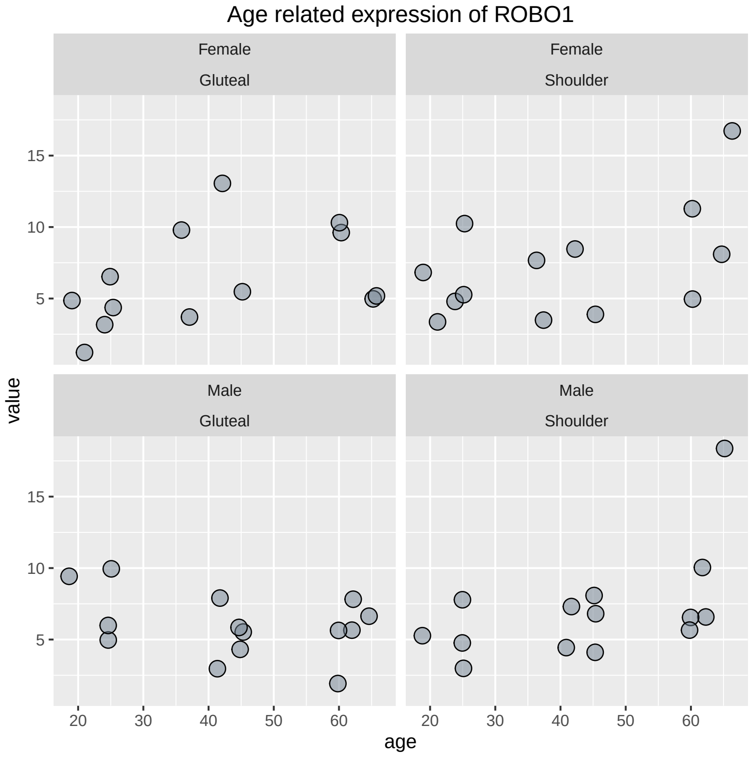

Figure 244: Loess regression for exon align depth

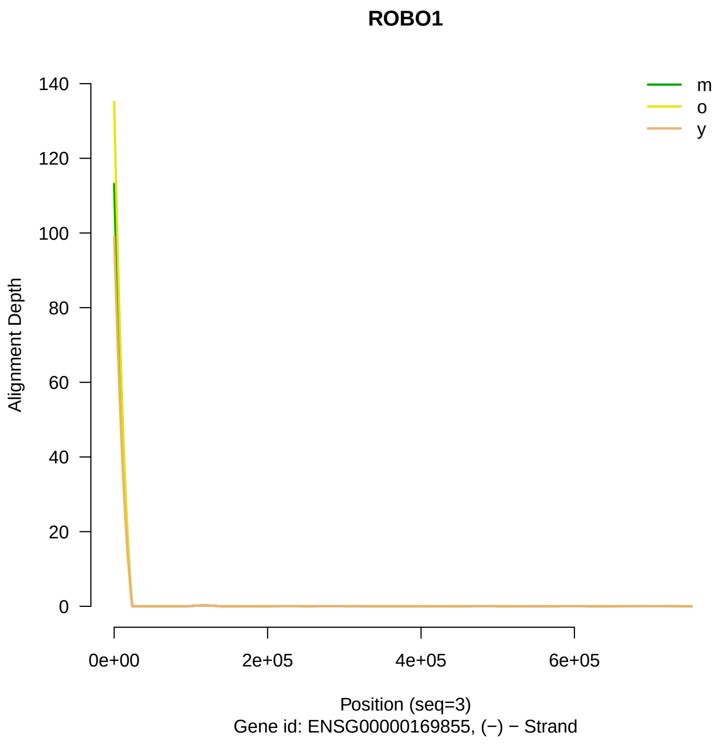

Figure 245: edgeR QLF test based CPM estimates

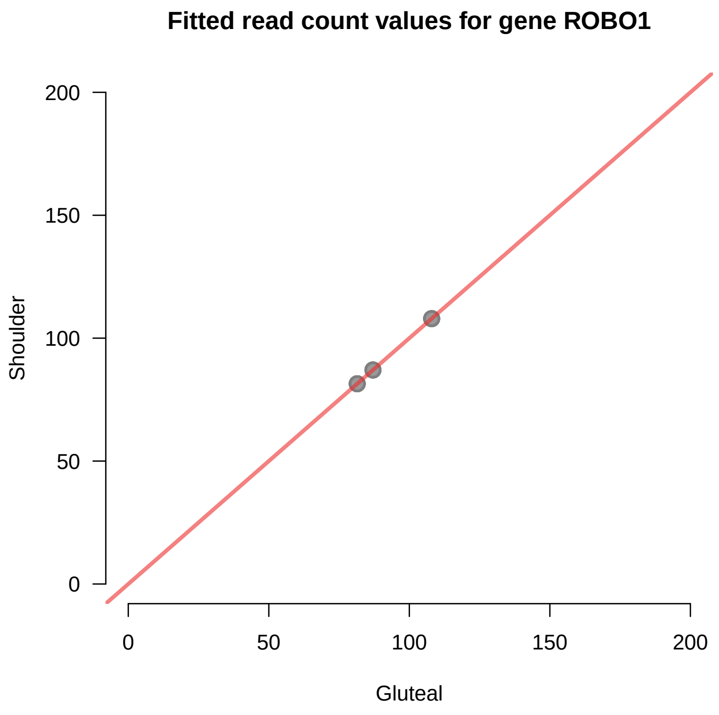

Figure 246: ReadExpSet based genewise CPM estimates

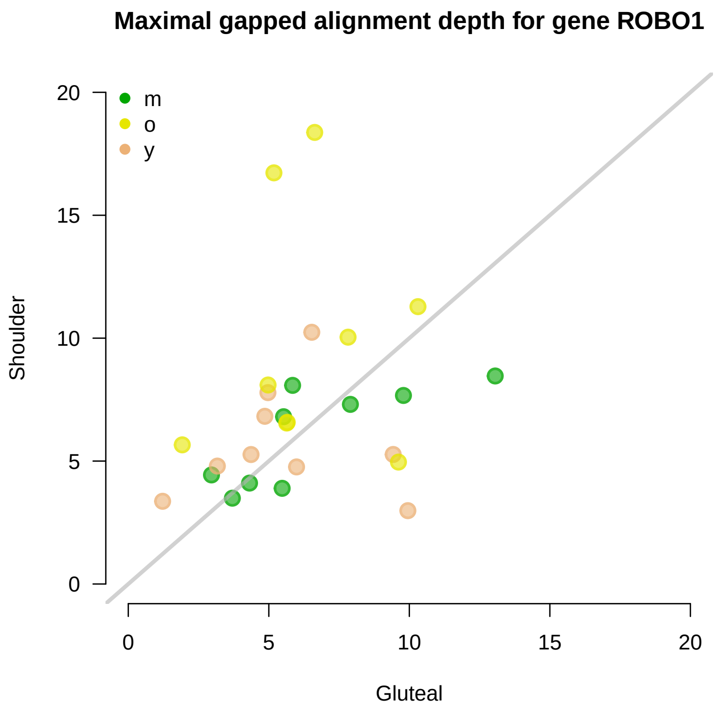

3.42 KCNC4

Parameter	Value
gene_name	KCNC4
gene_id	ENSG00000116396
maxald	187
old	down
seqid	1
strand	+
start	110211343
end	110283100
descr	potassium channel, voltage gated Shaw related subfamily C, member 4

Table 43: Gene identifcation

Gene expression estimates for Age

Figure 247: Gene expression estimates based on CPM (SummarizeOveraps)

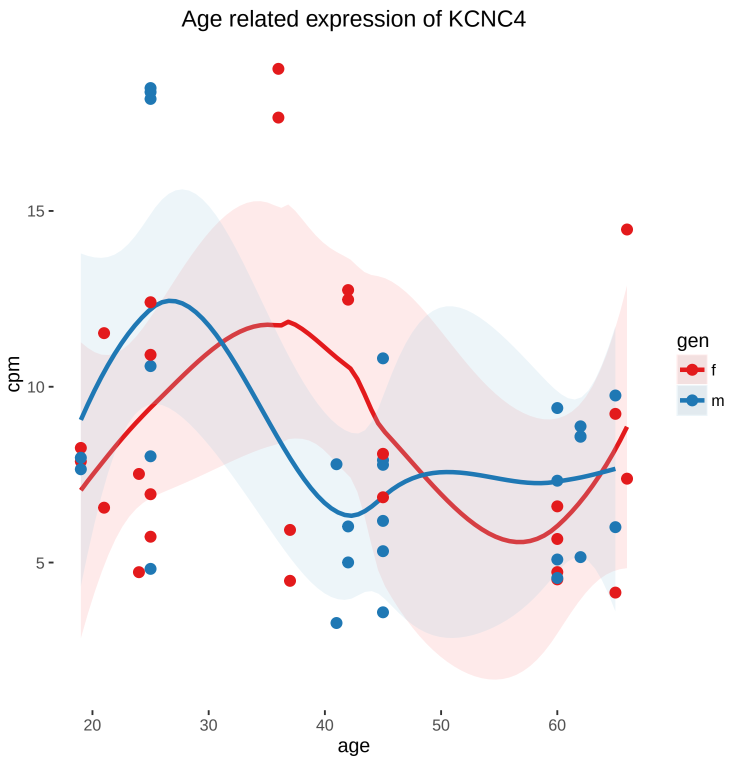

Influence of UV exposition on gene expression

Figure 248: edgeR QLF test based CPM estimates

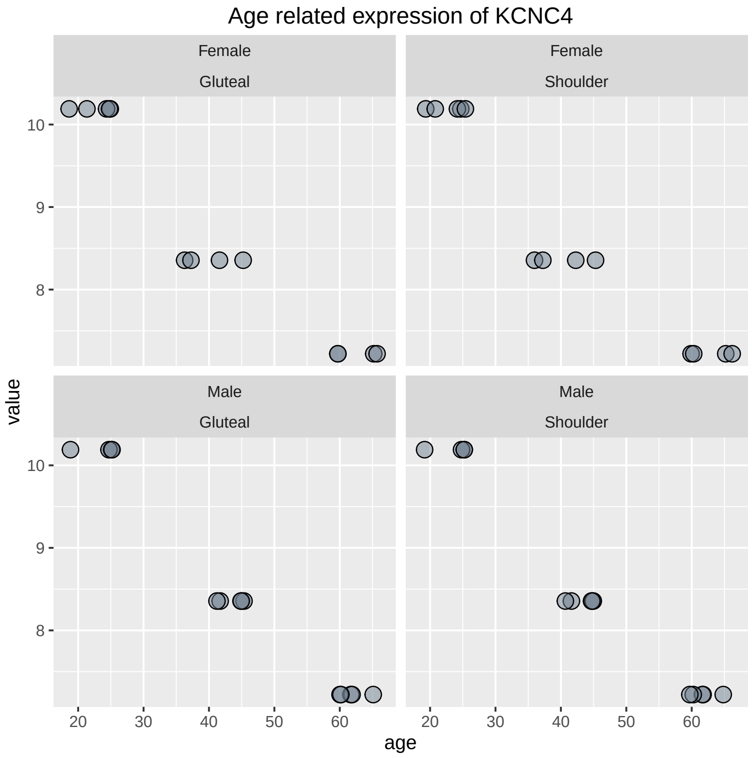

Figure 249: ReadExpSet based genewise CPM estimates

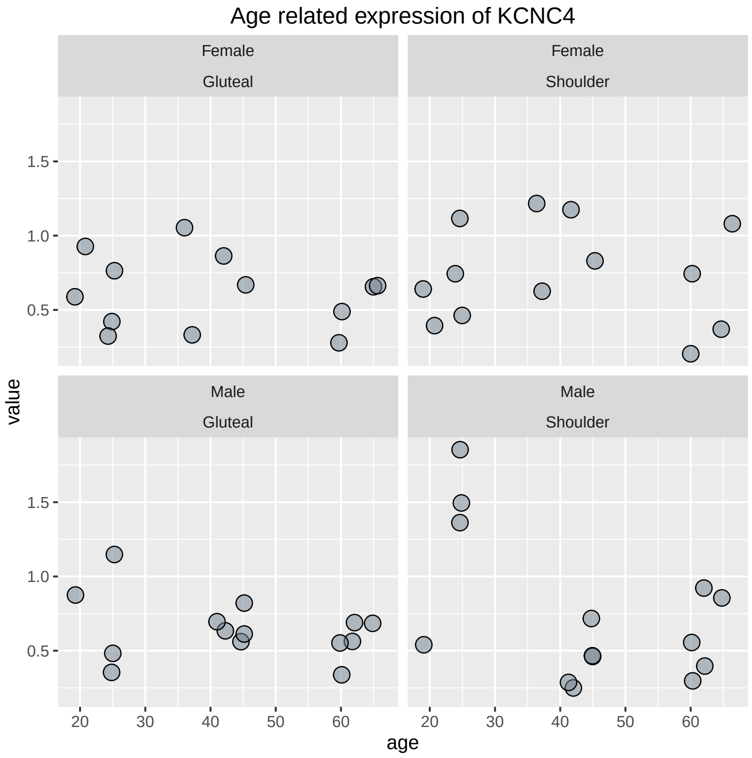

Figure 250: Loess regression for exon align depth

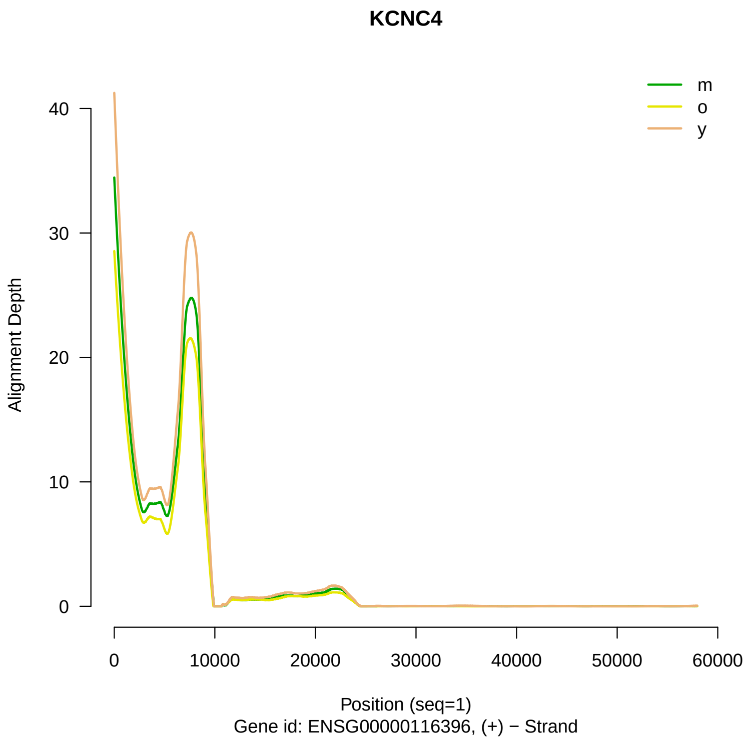

Figure 251: edgeR QLF test based CPM estimates

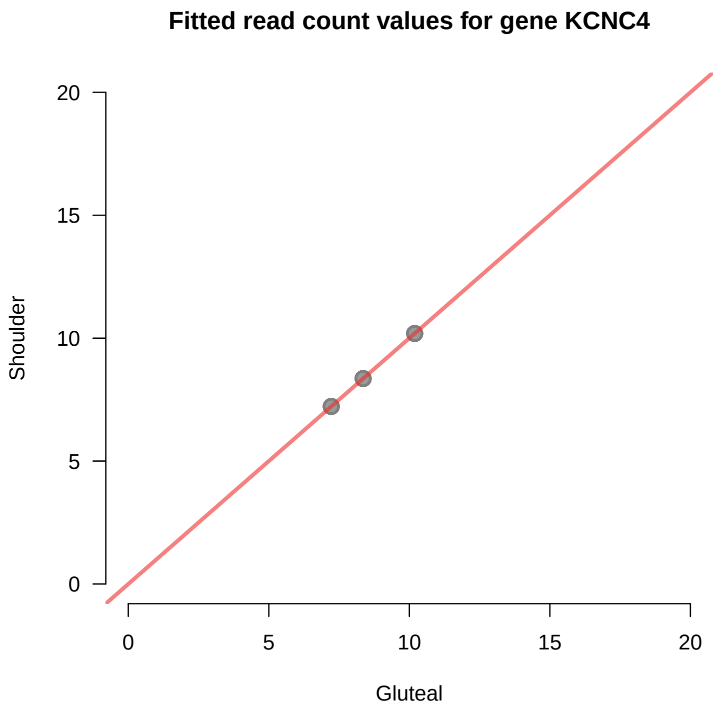

Figure 252: ReadExpSet based genewise CPM estimates

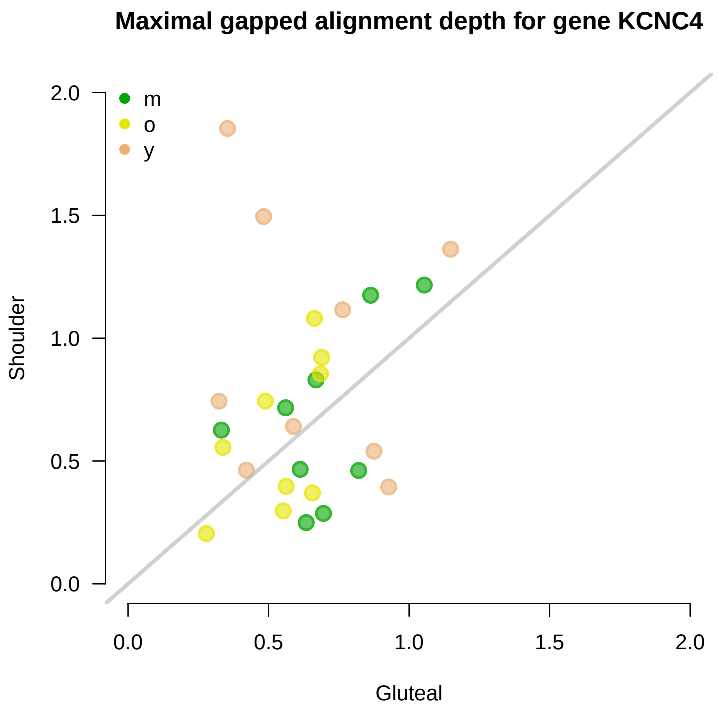

Supplement: S3 File — PDF file with gene expression data for the 42 age-related DE genes (derived from edgeR QLF test and ReadExpSet). (pdf) [file pone.0175657.s003.pdf]
