# Supplementary material for: Age, gender and UV-exposition related effects on gene expression in in vivo aged short term cultivated human dermal fibroblasts
Source: PLoS One. 2017 May 5;12(5):e0175657. doi: 10.1371/journal.pone.0175657 (PMC5419556; doi:10.1371/journal.pone.0175657)

Age related differential expression. Supplemental Material for: Age related Gene Expression Differences on Human Dermal Fibroblasts

Wolfgang Kaisers, CBiBs HHU Düsseldorf

June 16, 2016

Contents

1 Differential expression analysis **1**
1.1 Sample groups 1

2 Complete gene table **1**

3 Gene list **2**
3.1 ATOH8 2
3.2 PODXL 3
3.3 SNAI1 4
3.4 ID3 5
3.5 SPHK1 6
3.6 ID1 7
3.7 ERRFI1 8
3.8 PENK 9
3.9 SEPT5 10
3.10 CPZ 11
3.11 PRPS1 12
3.12 MEG3 13
3.13 CNN1 14
3.14 STC1 15
3.15 KIAA1324L 16
3.16 TRNP1 17
3.17 HSPB7 18
3.18 PRRX2 19
3.19 SMAD7 20
3.20 FAM83G 21
3.21 DDR1 22
3.22 PPP1R3C 23

3.23 EVA1A . . . 24
3.24 CRISPLD2 . . . 25
3.25 RP11-309L24.6 . . . 26
3.26 ZNF385D . . . 27
3.27 FGFRL1 . . . 28
3.28 CKB . . . 29
3.29 FILIP1L . . . 30
3.30 GJA1 . . . 31
3.31 ENC1 . . . 32
3.32 SH2D4A . . . 33
3.33 ARHGAP23P1 . . . 34
3.34 SERTAD1 . . . 35
3.35 FGF13 . . . 36
3.36 EHD1 . . . 37
3.37 USP41 . . . 38
3.38 ACSS3 . . . 39
3.39 BACE2 . . . 40
3.40 ADGRL4 . . . 41
3.41 ROBO1 . . . 42
3.42 KCNC4 . . . 43

1 Differential expression analysis

1.1 Sample groups

Sample groups for differential expression analysis varied by donor age. The three disinguished groups are:

- Young (18-25 years)
- Middle (35-49 years)
- Old (60-67 years)

2 Complete gene table

gene_name	gene_id	maxald	old	seqid	strand	ID
ATOH8	ENSG00000168874	4308	down	2	+	1
PODXL	ENSG00000128567	3927	down	7	-	2
SNAI1	ENSG00000124216	4575	down	20	+	3
ID3	ENSG00000117318	19530	down	1	-	4
SPHK1	ENSG00000176170	6585	down	17	+	5
ID1	ENSG00000125968	14913	down	20	+	6

Continued on next page

gene_name	gene_id	maxald	old	seqid	strand	ID
ERRFI1	ENSG00000116285	1776	down	1	-	7
PENK	ENSG00000181195	2554	up	8	-	8
SEPT5	ENSG00000184702	1468	down	22	+	9
CPZ	ENSG00000109625	2585	up	4	+	10
PRPS1	ENSG00000147224	5550	down	X	+	11
MEG3	ENSG00000214548	3031	down	14	+	12
CNN1	ENSG00000130176	16224	down	19	+	13
STC1	ENSG00000159167	1877	up	8	-	14
KIAA1324L	ENSG00000164659	1751	up	7	-	15
TRNP1	ENSG00000253368	3613	down	1	+	16
HSPB7	ENSG00000173641	14954	down	1	-	17
PRRX2	ENSG00000167157	6616	down	9	+	18
SMAD7	ENSG00000101665	3768	down	18	-	19
FAM83G	ENSG00000188522	523	down	17	-	20
DDR1	ENSG00000204580	606	down	6	+	21
PPP1R3C	ENSG00000119938	1889	down	10	-	22
EVA1A	ENSG00000115363	862	down	2	-	23
CRISPLD2	ENSG00000103196	2516	down	16	+	24
RP11-309L24.6	ENSG00000224163	6288	down	7	-	25
ZNF385D	ENSG00000151789	529	up	3	-	26
FGFRL1	ENSG00000127418	1174	down	4	+	27
CKB	ENSG00000166165	2224	down	14	-	28
FILIP1L	ENSG00000168386	2028	down	3	-	29
GJA1	ENSG00000152661	12333	up	6	+	30
ENC1	ENSG00000171617	1200	down	5	-	31
SH2D4A	ENSG00000104611	5544	down	8	+	32
ARHGAP23P1	ENSG00000260781	763	down	16	-	33
SERTAD1	ENSG00000197019	2018	down	19	-	34
FGF13	ENSG00000129682	345	up	X	-	35
EHD1	ENSG00000110047	2871	down	11	-	36
USP41	ENSG00000161133	155	up	22	-	37
ACSS3	ENSG00000111058	201	down	12	+	38
BACE2	ENSG00000182240	2384	up	21	+	39
ADGRL4	ENSG00000162618	524	up	1	-	40
ROBO1	ENSG00000169855	826	up	3	-	41
KCNC4	ENSG00000116396	187	down	1	+	42

Table 1: Age differential expressed genes

3 Gene list

3.1 ATOH8

Figure 1: Genewise CPM estimates from SOV

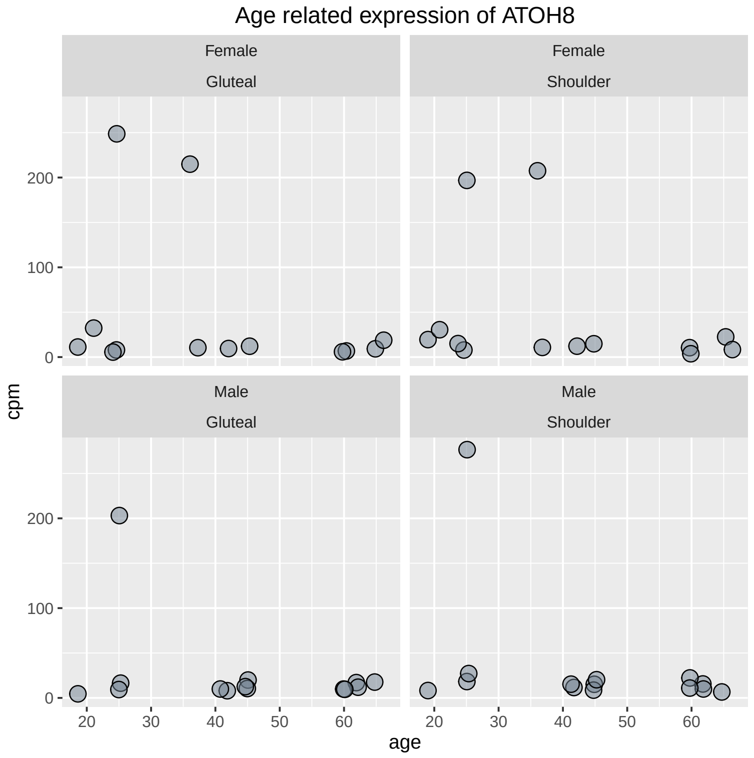

3.2 PODXL

Figure 2: Genewise CPM estimates from SOV

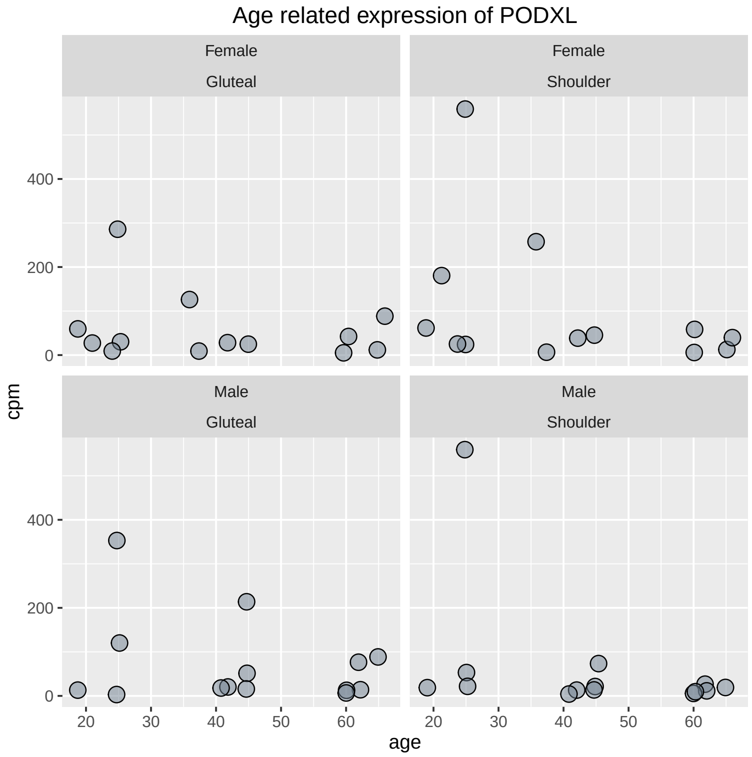

3.3 SNAI1

Figure 3: Genewise CPM estimates from SOV

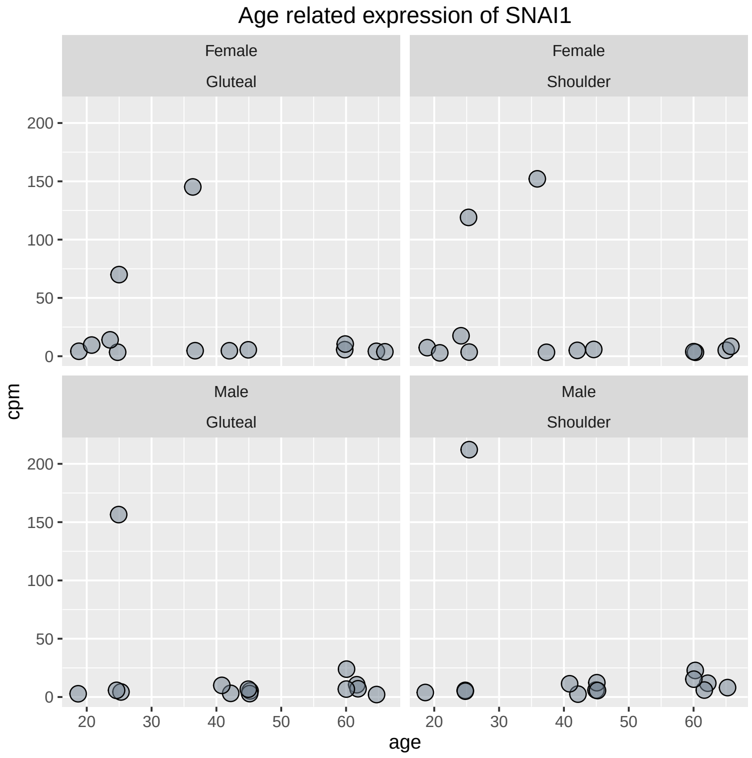

3.4 ID3

Figure 4: Genewise CPM estimates from SOV

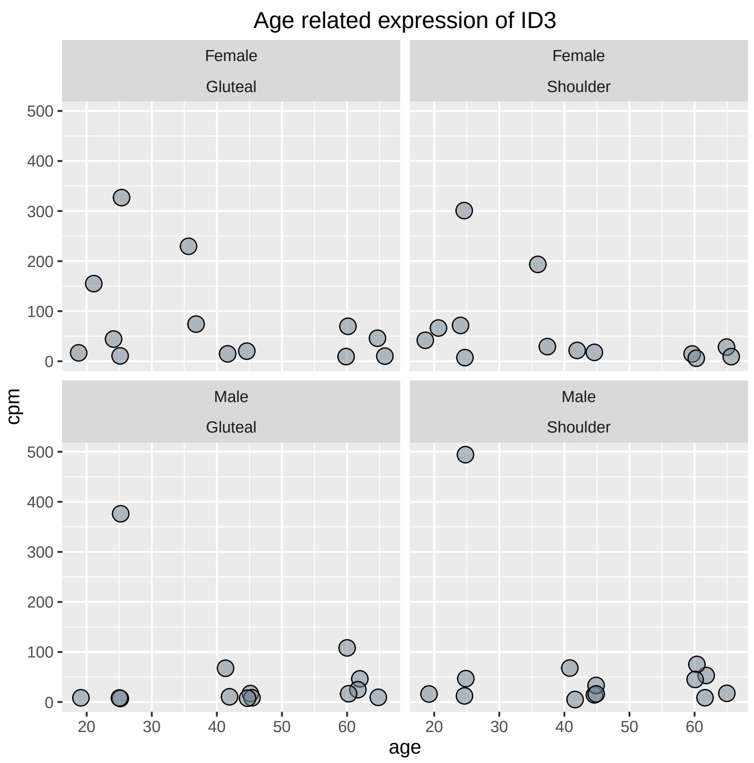

3.5 SPHK1

Figure 5: Genewise CPM estimates from SOV

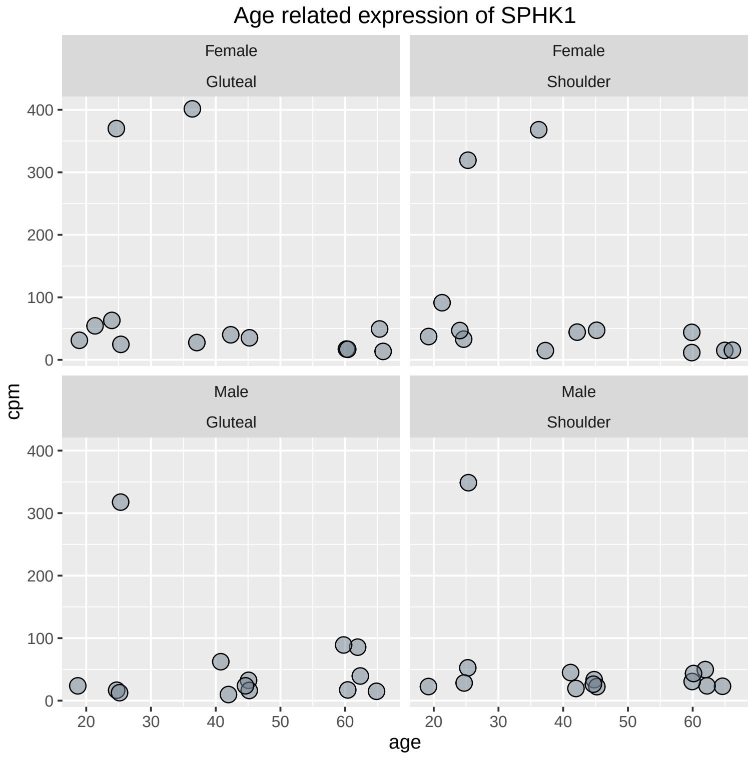

3.6 ID1

Figure 6: Genewise CPM estimates from SOV

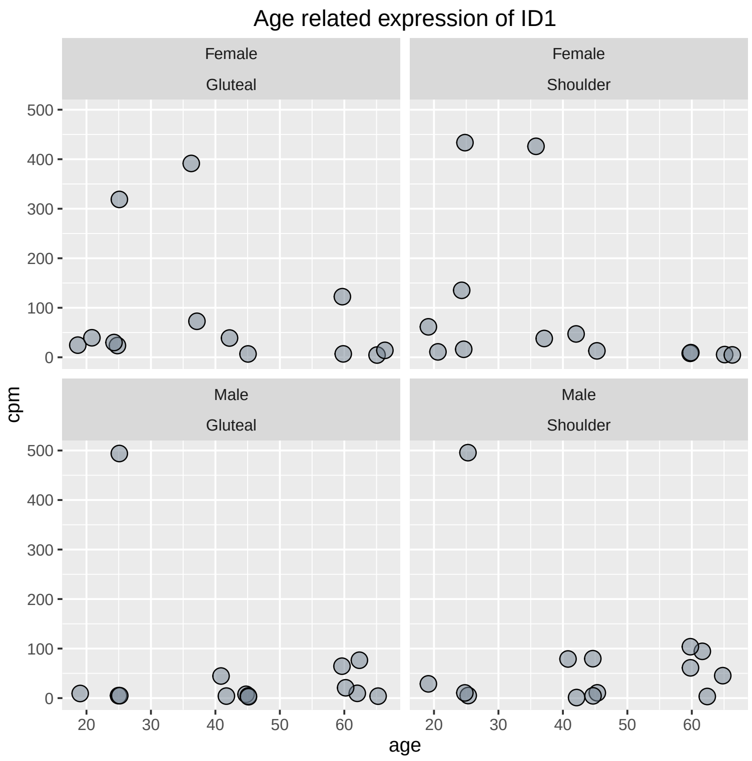

3.7 ERRFI1

Figure 7: Genewise CPM estimates from SOV

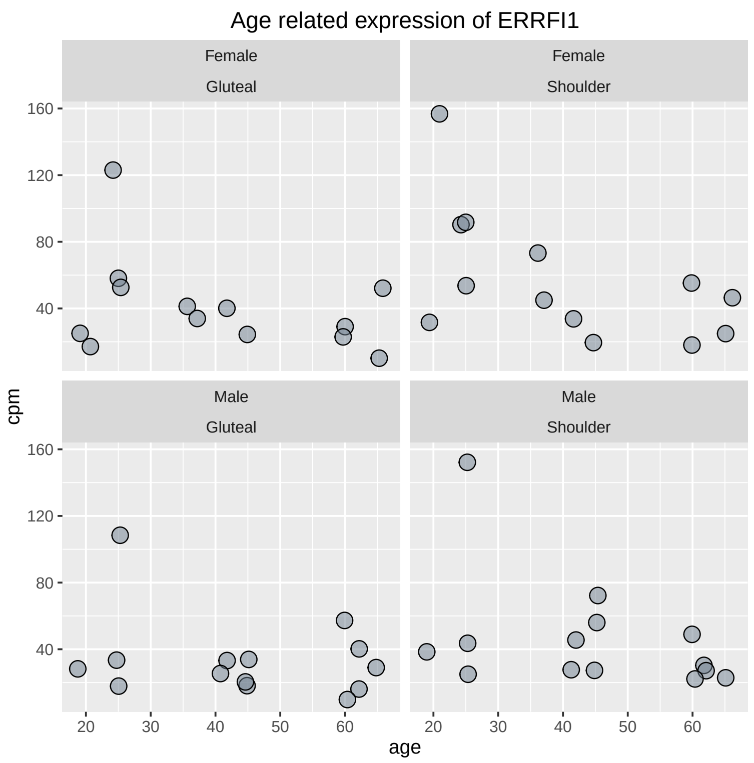

3.8 PENK

Figure 8: Genewise CPM estimates from SOV

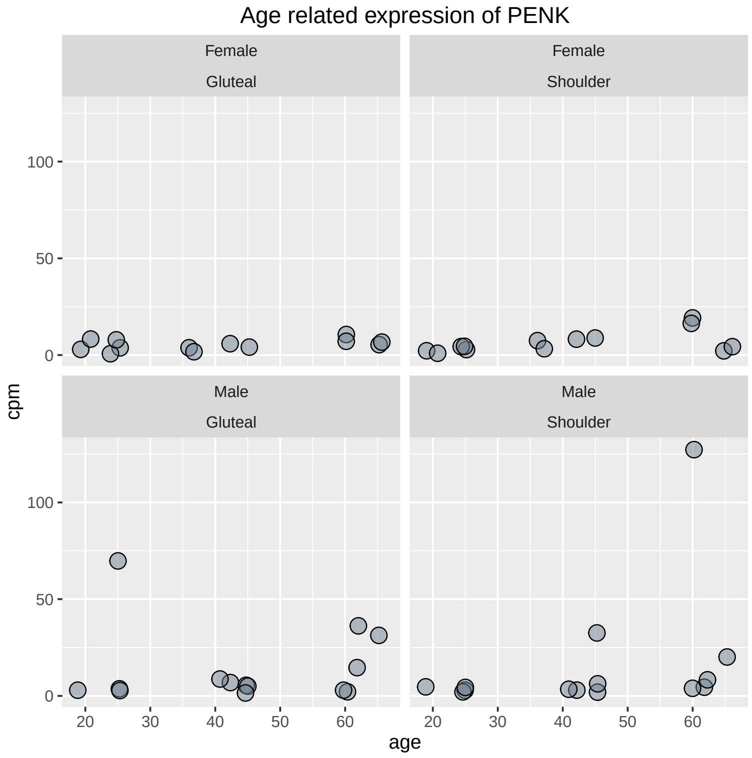

3.9 SEPT5

Figure 9: Genewise CPM estimates from SOV

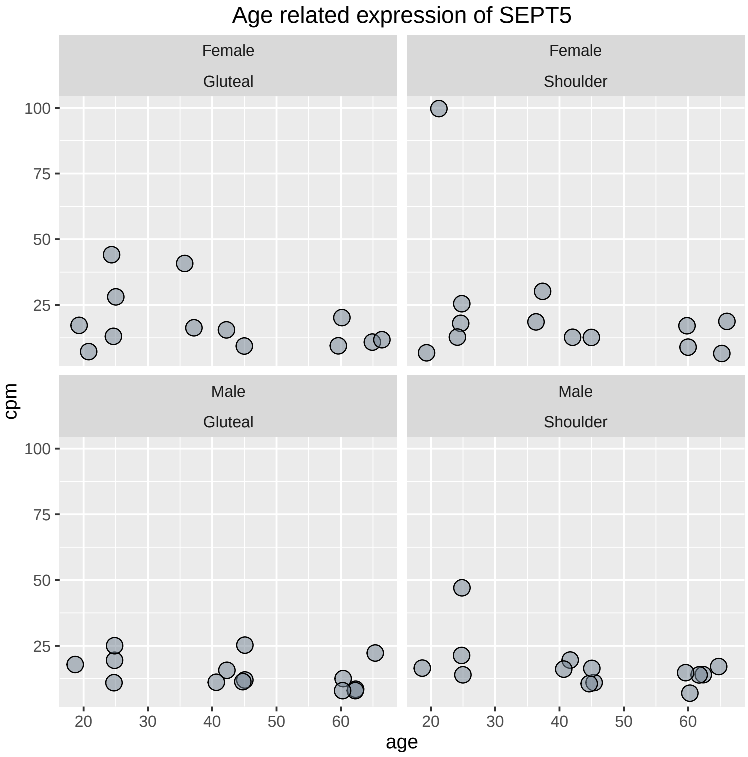

3.10 CPZ

Figure 10: Genewise CPM estimates from SOV

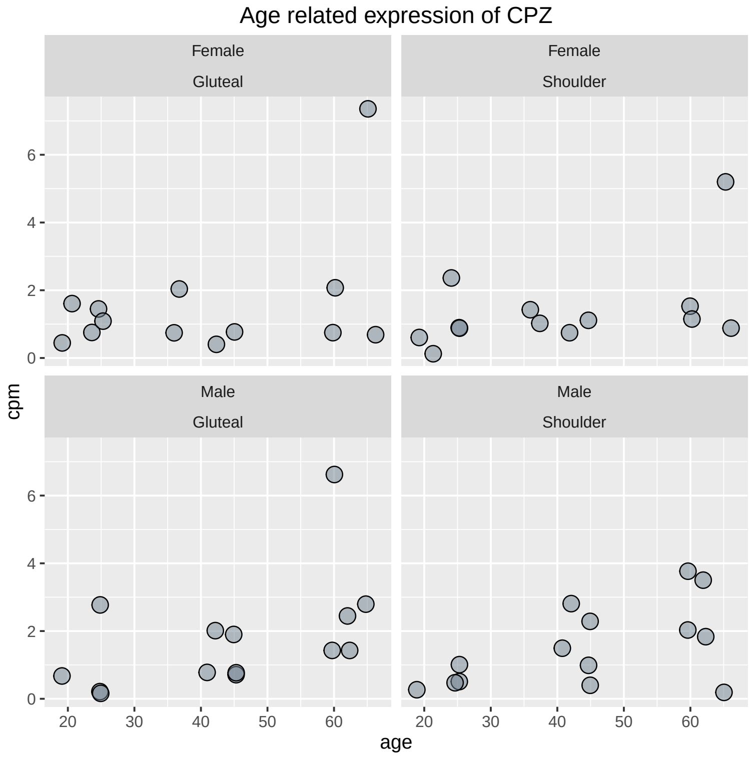

3.11 PRPS1

Figure 11: Genewise CPM estimates from SOV

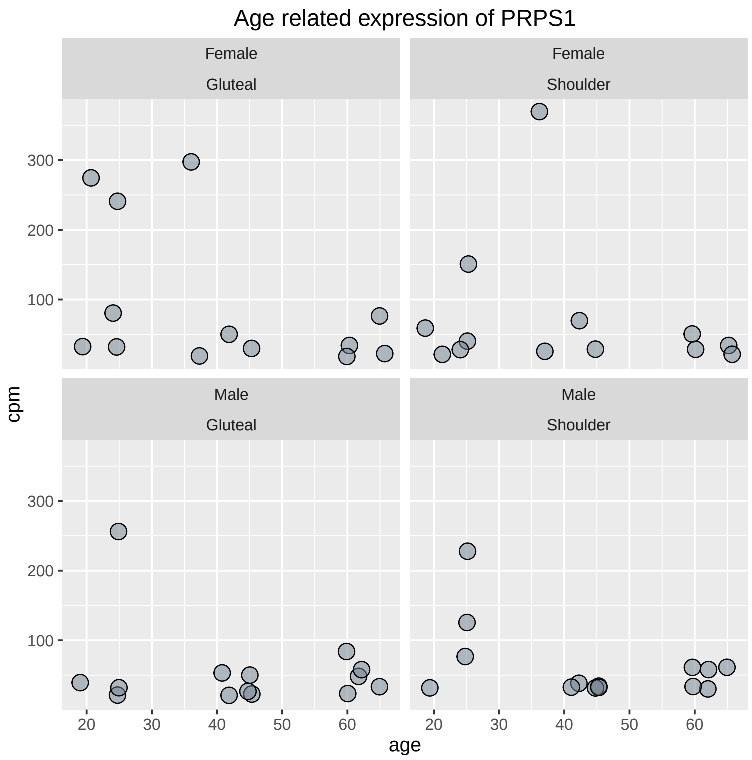

3.12 MEG3

Figure 12: Genewise CPM estimates from SOV

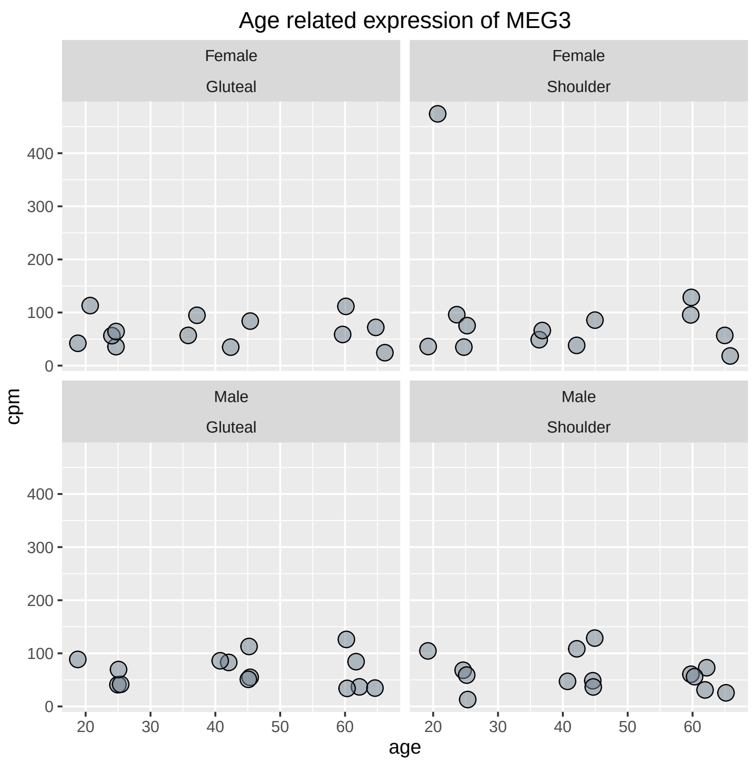

3.13 CNN1

Figure 13: Genewise CPM estimates from SOV

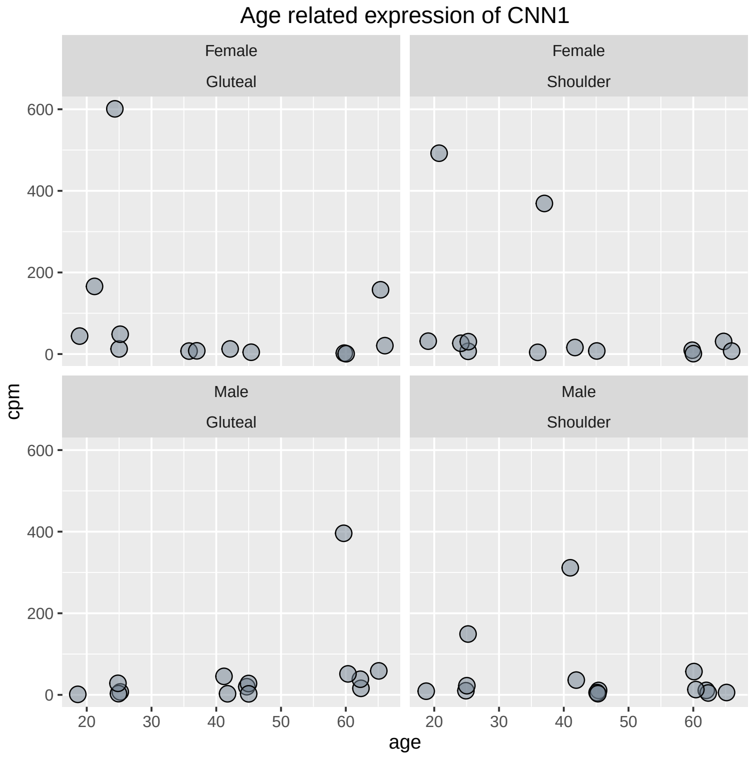

3.14 STC1

Figure 14: Genewise CPM estimates from SOV

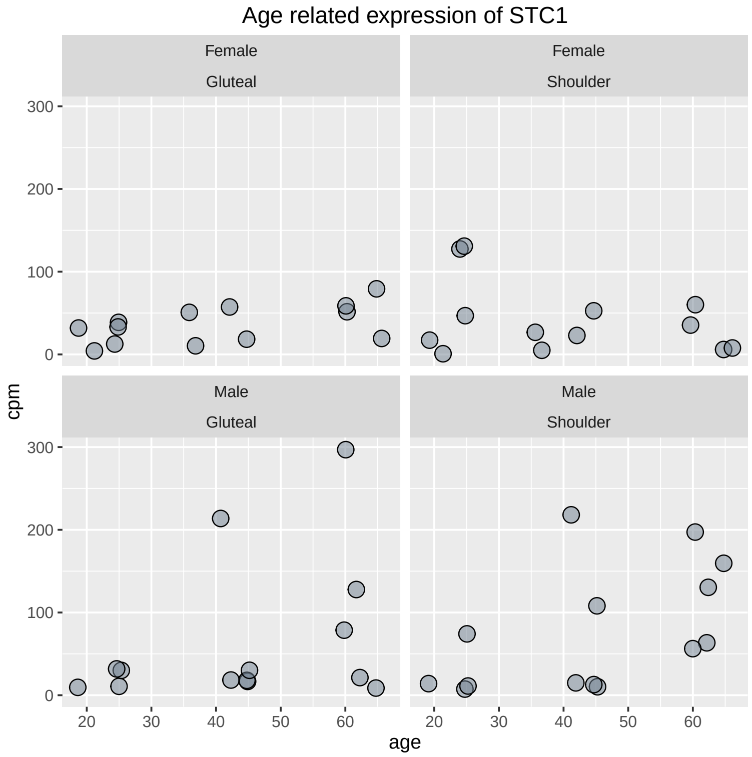

3.15 KIAA1324L

Figure 15: Genewise CPM estimates from SOV

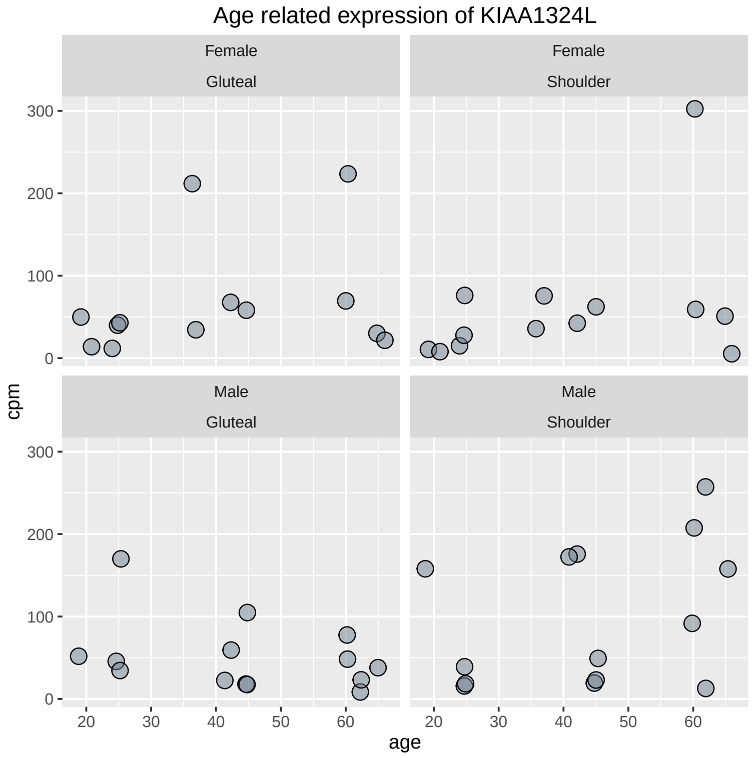

3.16 TRNP1

Figure 16: Genewise CPM estimates from SOV

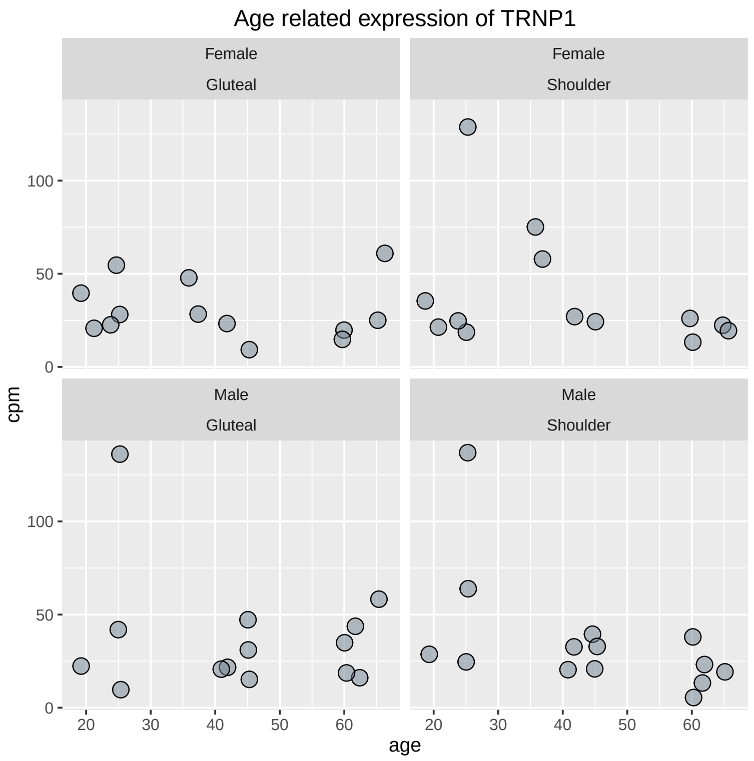

3.17 HSPB7

Figure 17: Genewise CPM estimates from SOV

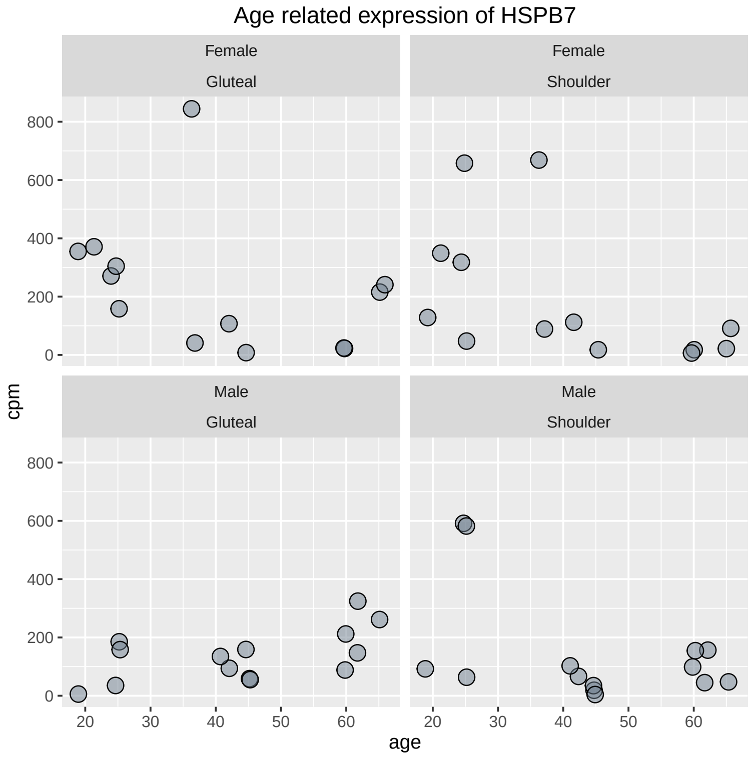

3.18 PRRX2

Figure 18: Genewise CPM estimates from SOV

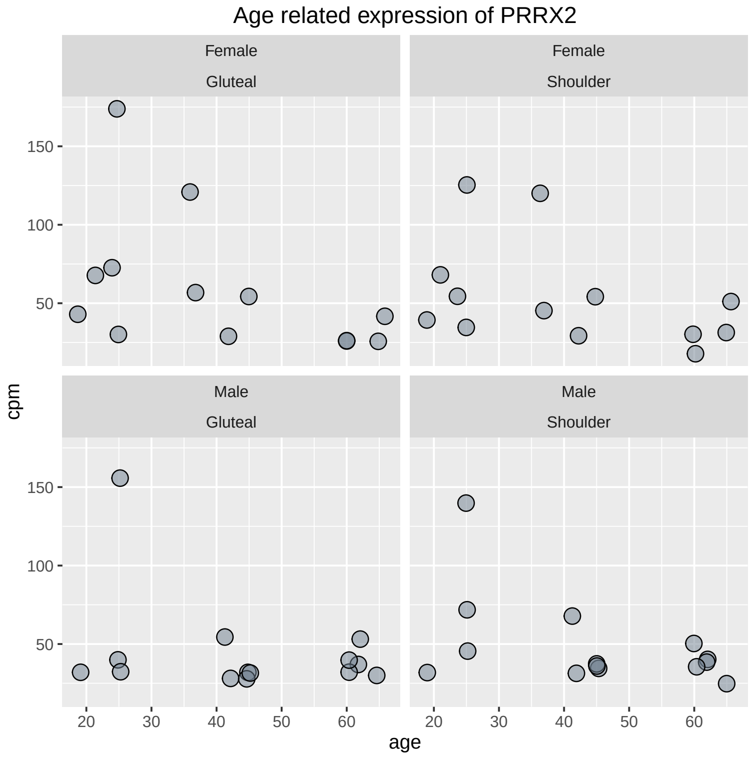

3.19 SMAD7

Figure 19: Genewise CPM estimates from SOV

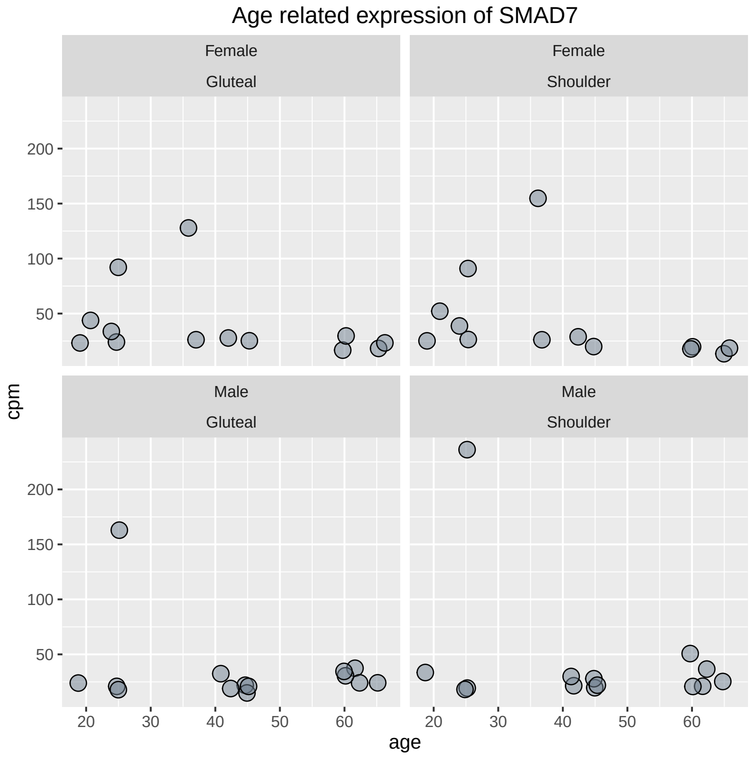

3.20 FAM83G

Figure 20: Genewise CPM estimates from SOV

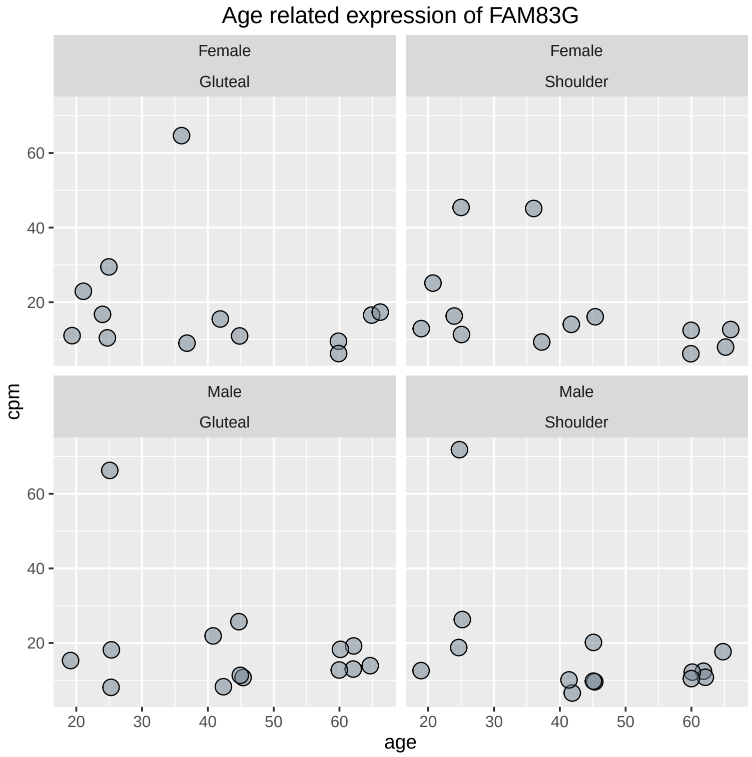

3.21 DDR1

Figure 21: Genewise CPM estimates from SOV

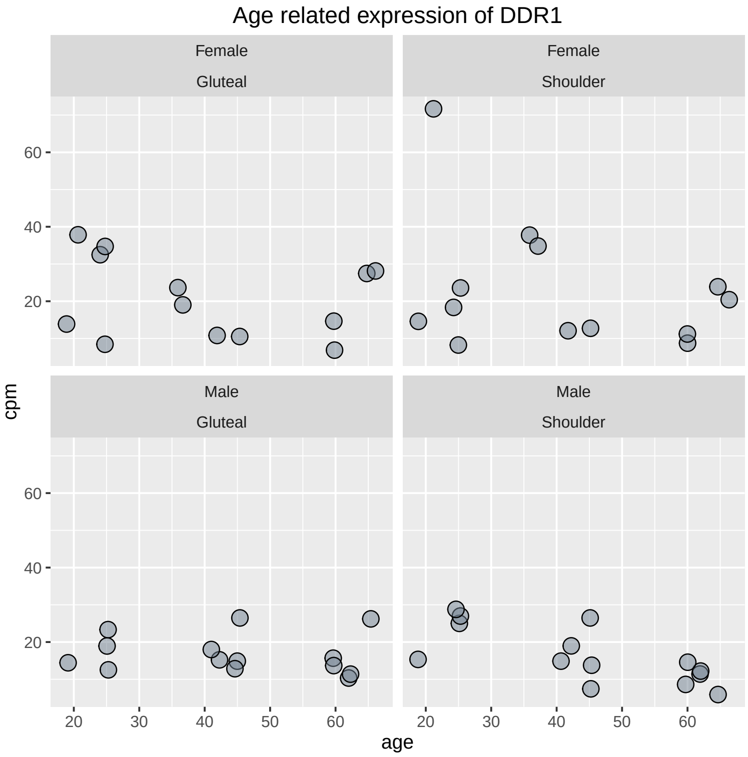

3.22 PPP1R3C

Figure 22: Genewise CPM estimates from SOV

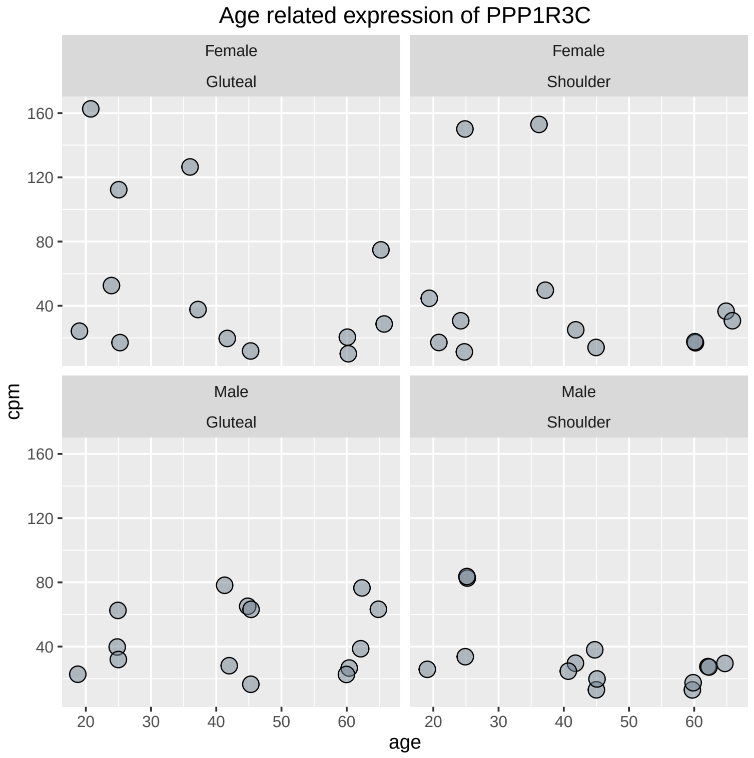

3.23 EVA1A

Figure 23: Genewise CPM estimates from SOV

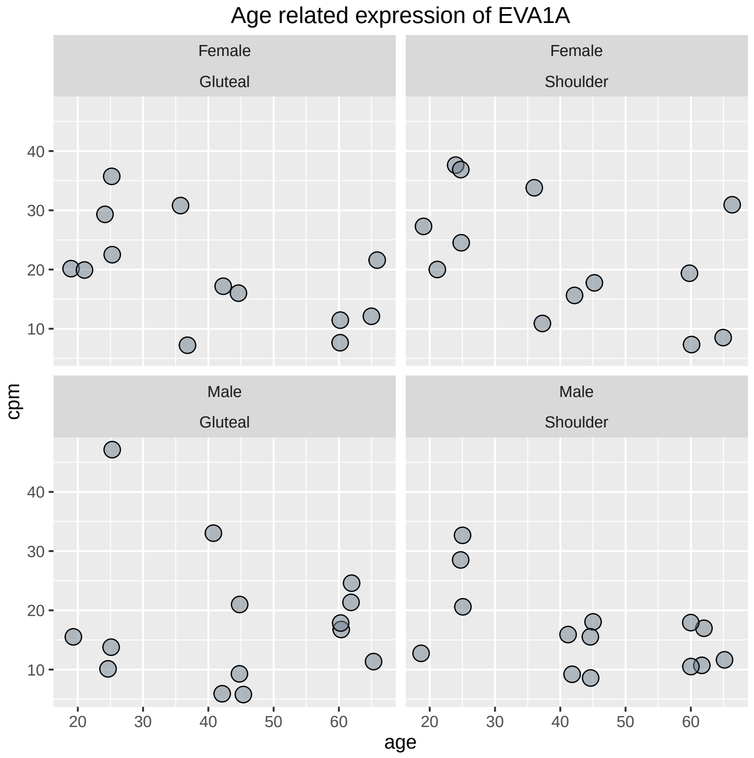

3.24 CRISPLD2

Figure 24: Genewise CPM estimates from SOV

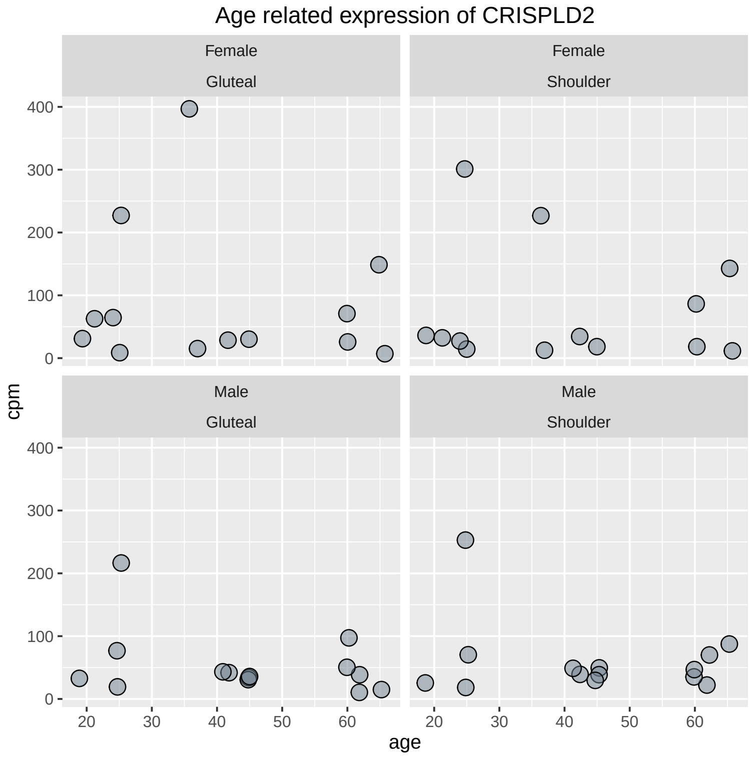

3.25 RP11-309L24.6

Figure 25: Genewise CPM estimates from SOV

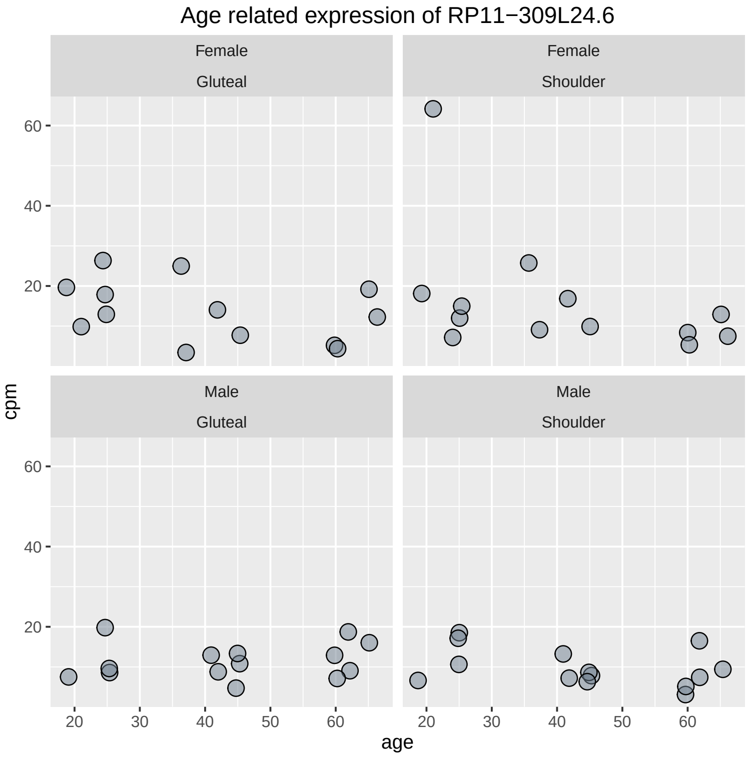

3.26 ZNF385D

Figure 26: Genewise CPM estimates from SOV

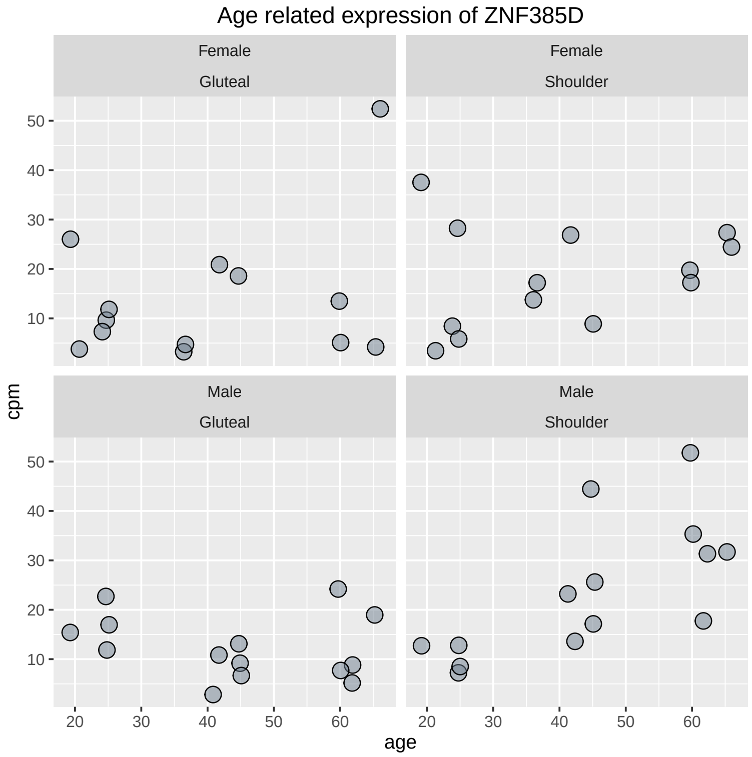

3.27 FGFRL1

Figure 27: Genewise CPM estimates from SOV

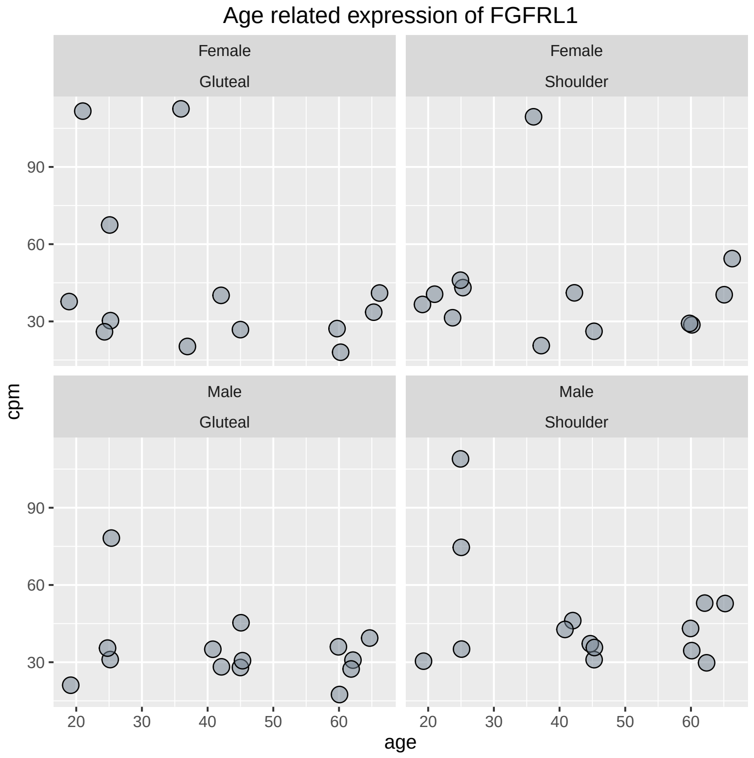

3.28 CKB

Figure 28: Genewise CPM estimates from SOV

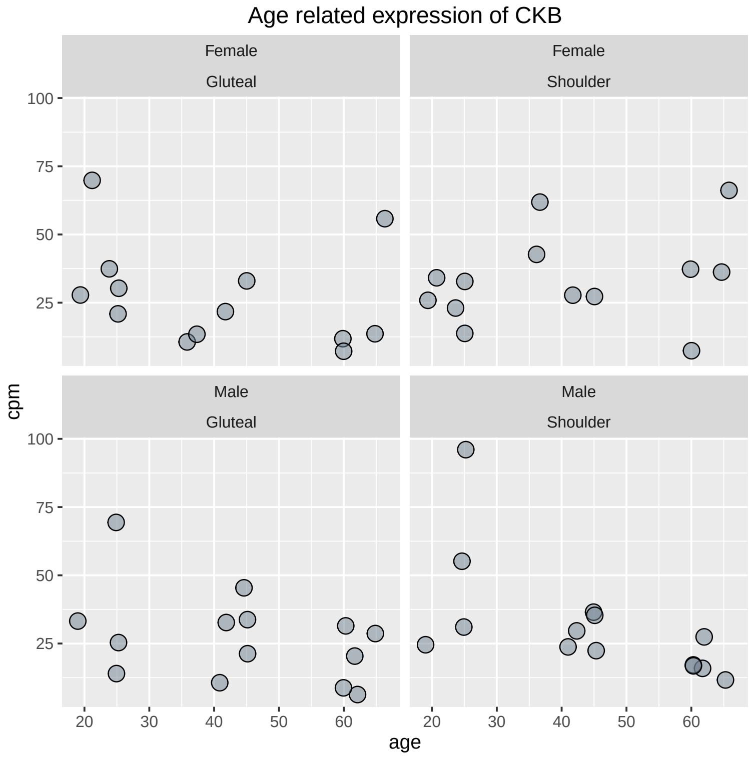

3.29 FILIP1L

Figure 29: Genewise CPM estimates from SOV

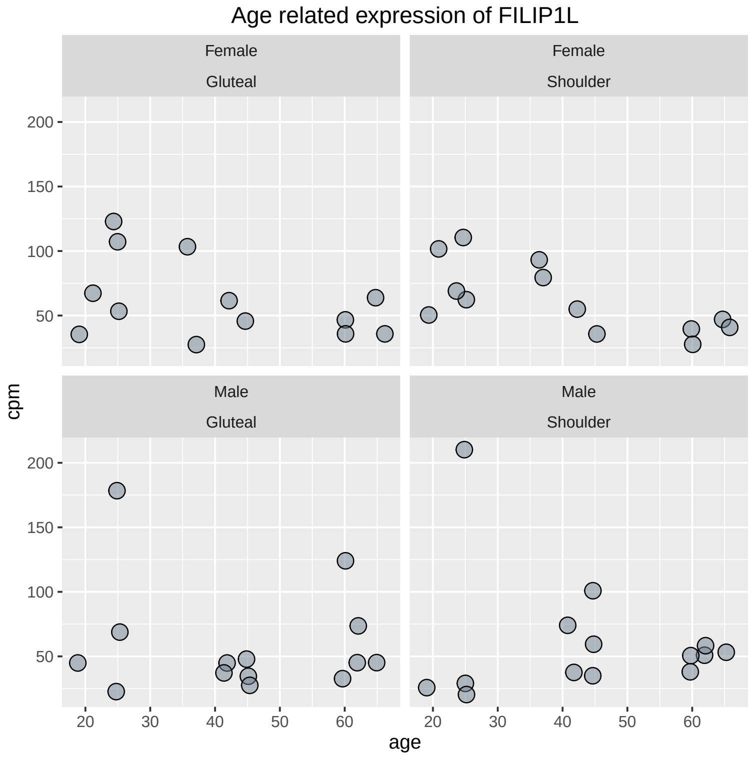

3.30 GJA1

Figure 30: Genewise CPM estimates from SOV

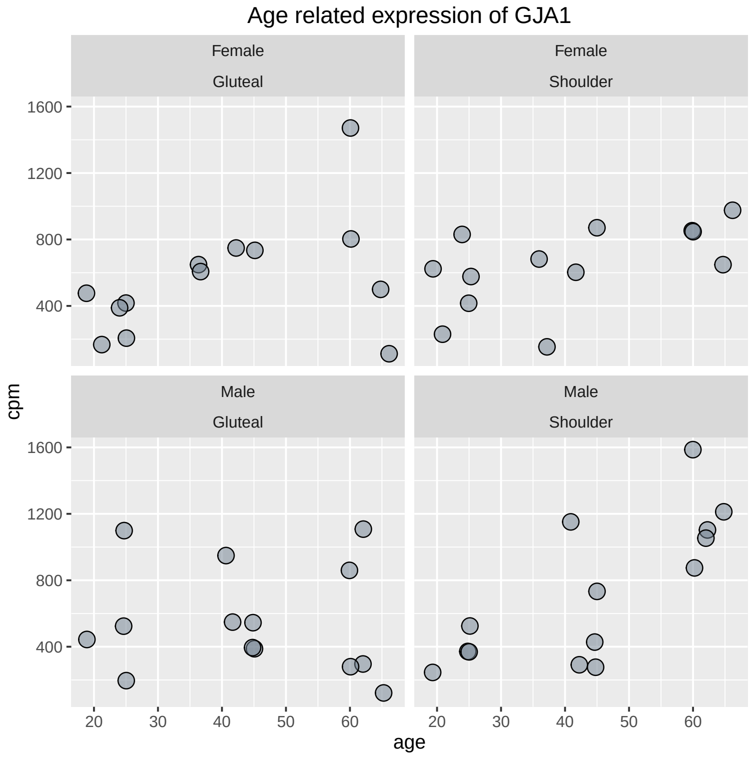

3.31 ENC1

Figure 31: Genewise CPM estimates from SOV

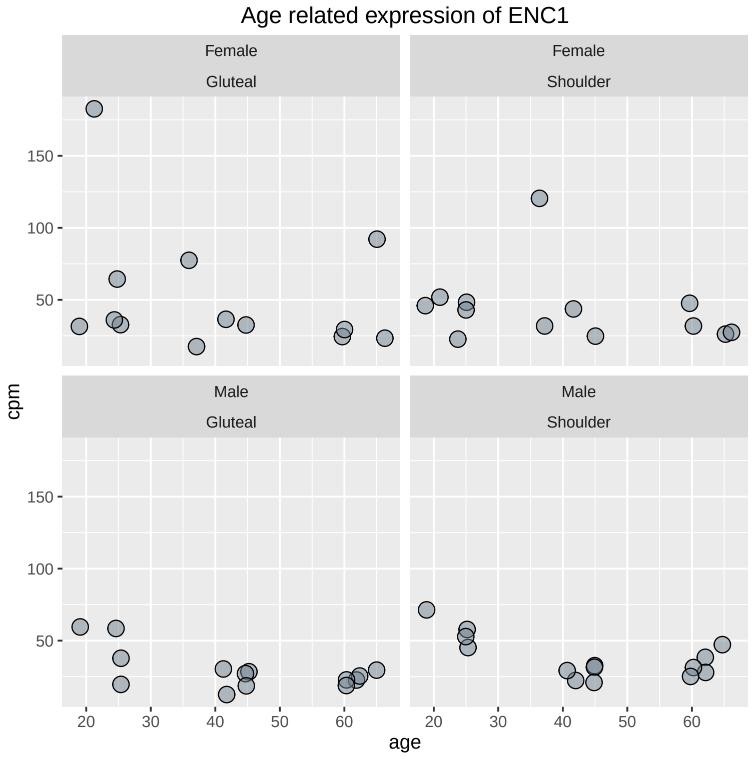

3.32 SH2D4A

Figure 32: Genewise CPM estimates from SOV

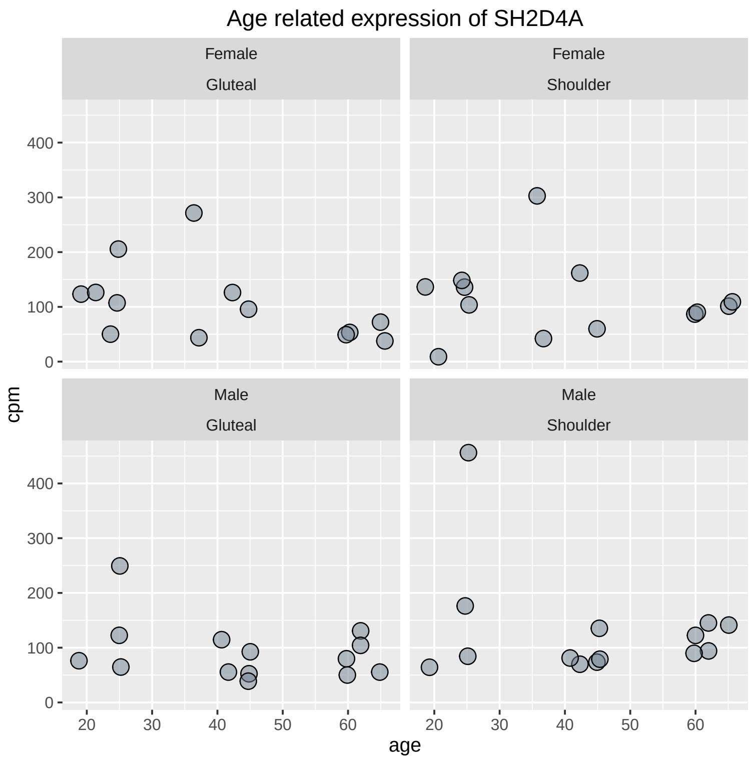

3.33 ARHGAP23P1

Figure 33: Genewise CPM estimates from SOV

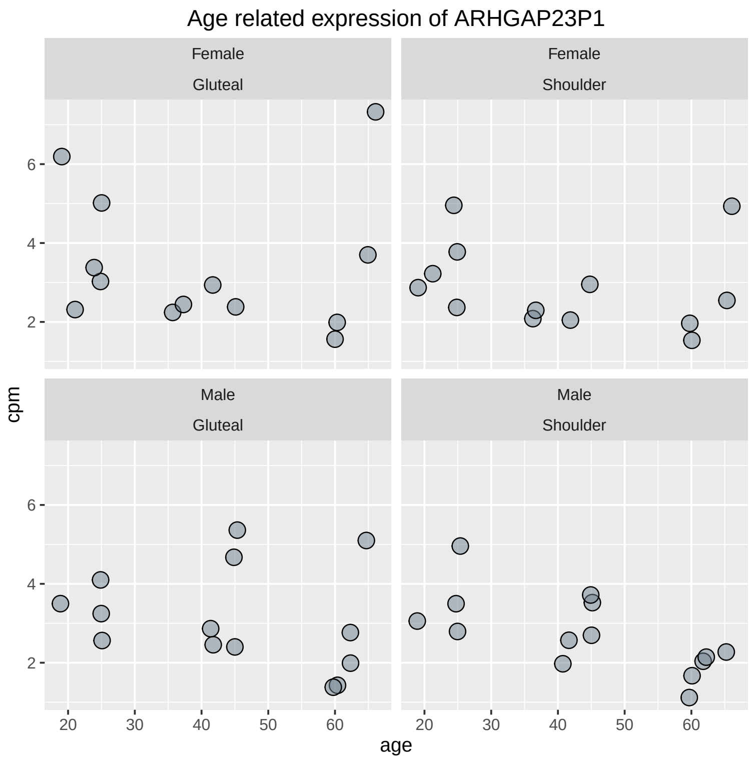

3.34 SERTAD1

Figure 34: Genewise CPM estimates from SOV

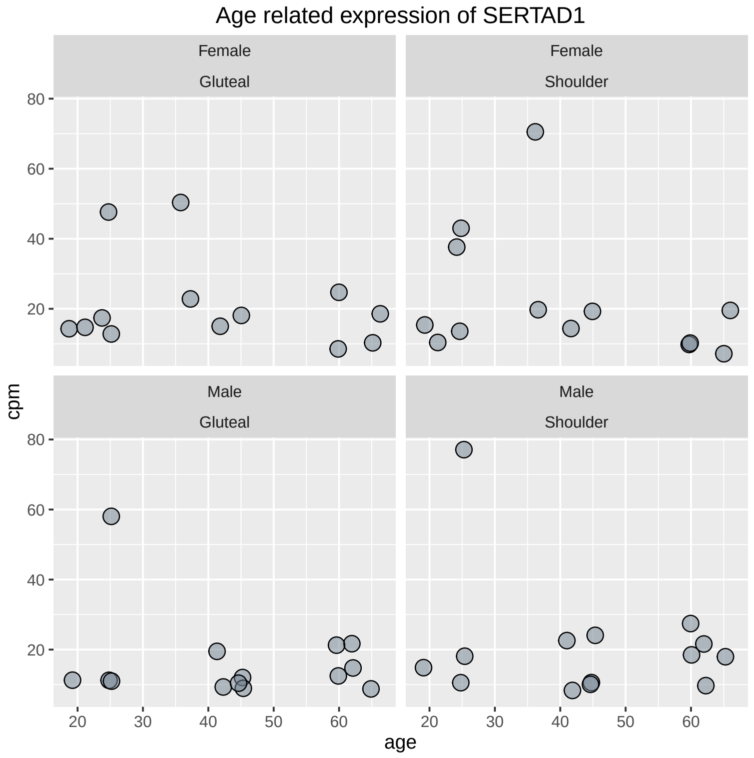

3.35 FGF13

Figure 35: Genewise CPM estimates from SOV

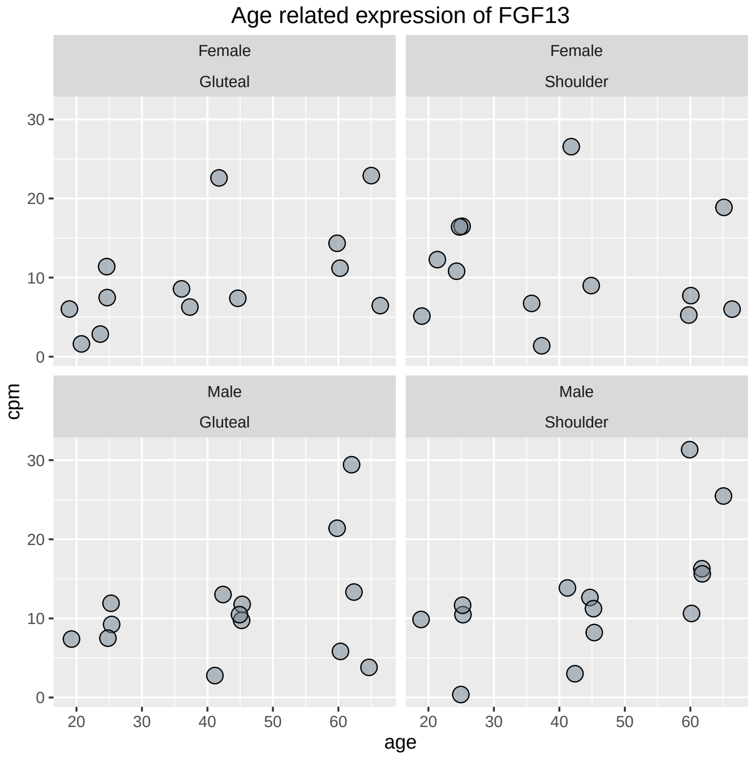

3.36 EHD1

Figure 36: Genewise CPM estimates from SOV

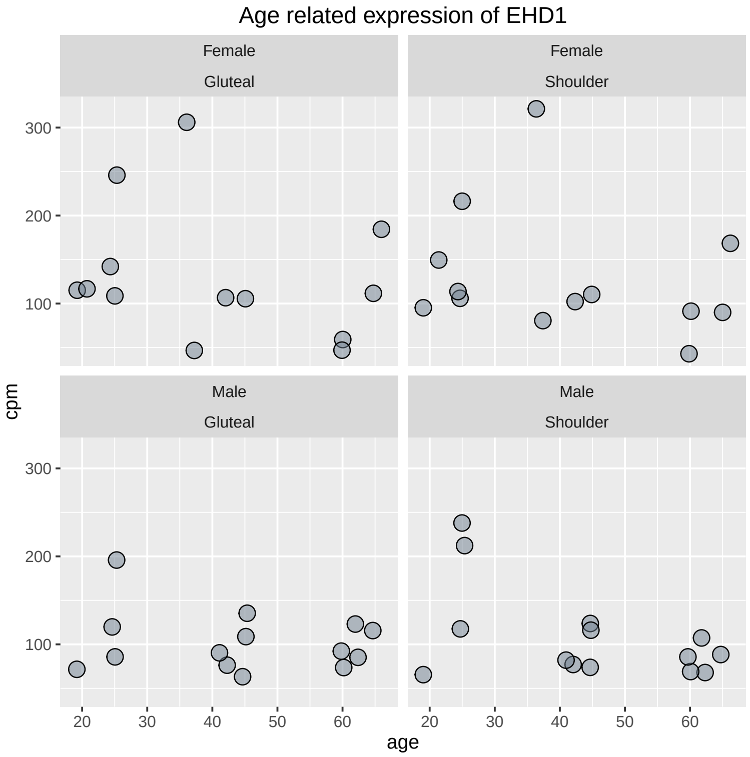

3.37 USP41

Figure 37: Genewise CPM estimates from SOV

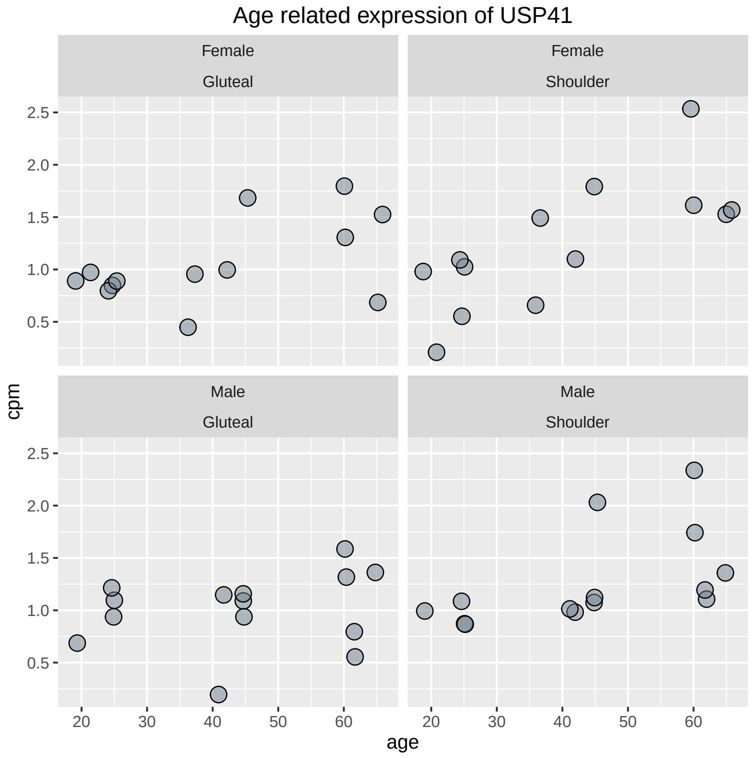

3.38 ACSS3

Figure 38: Genewise CPM estimates from SOV

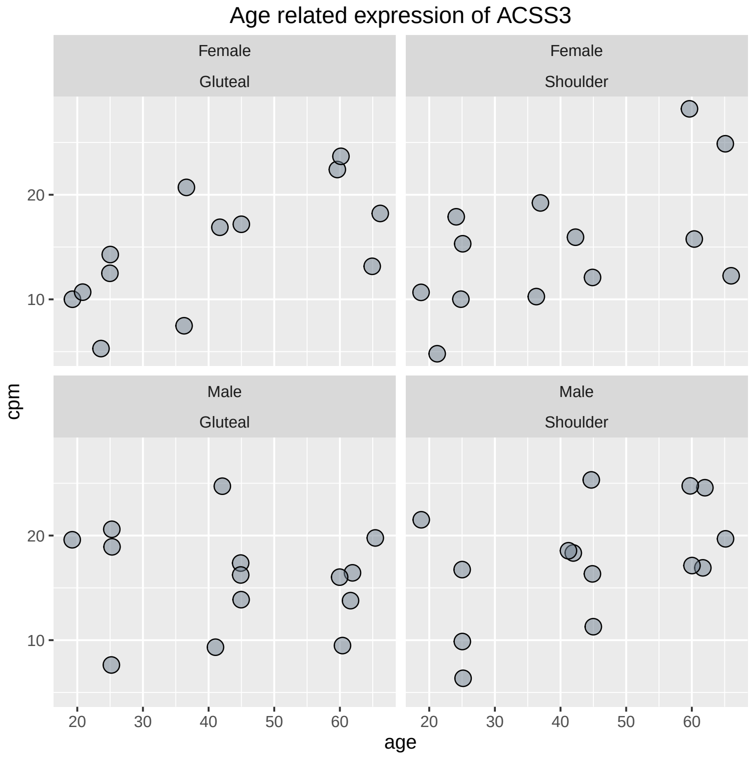

3.39 BACE2

Figure 39: Genewise CPM estimates from SOV

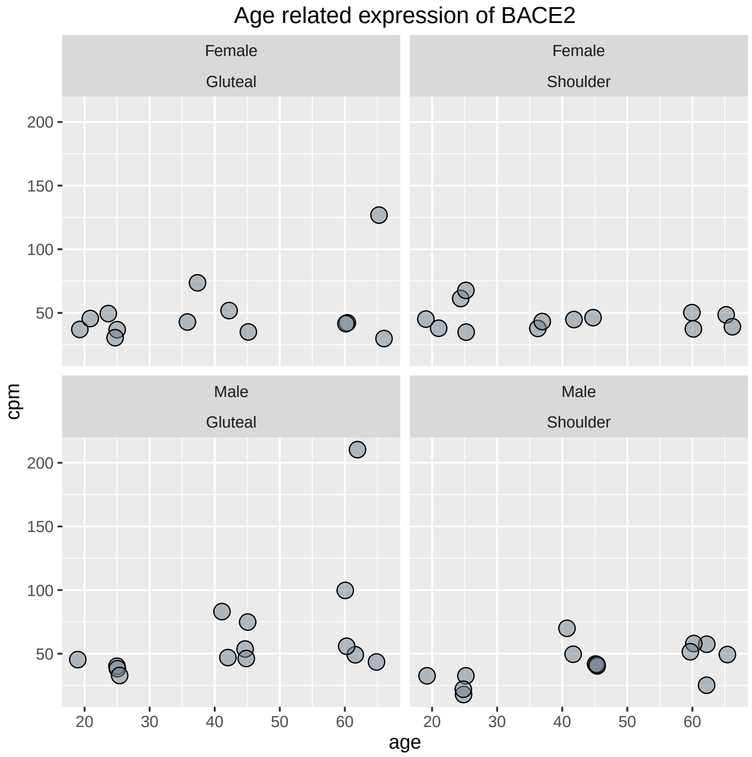

3.40 ADGRL4

Figure 40: Genewise CPM estimates from SOV

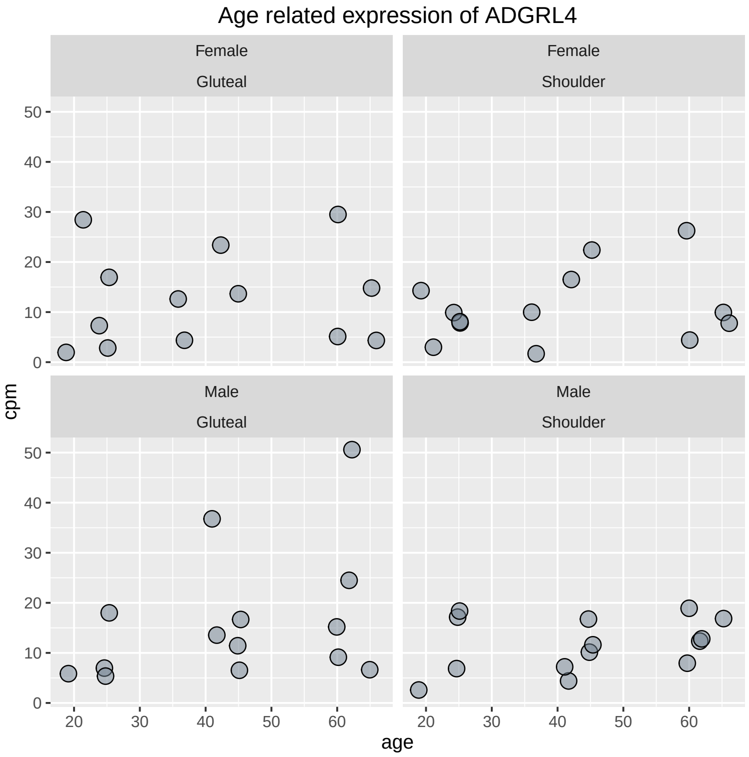

3.41 ROBO1

Figure 41: Genewise CPM estimates from SOV

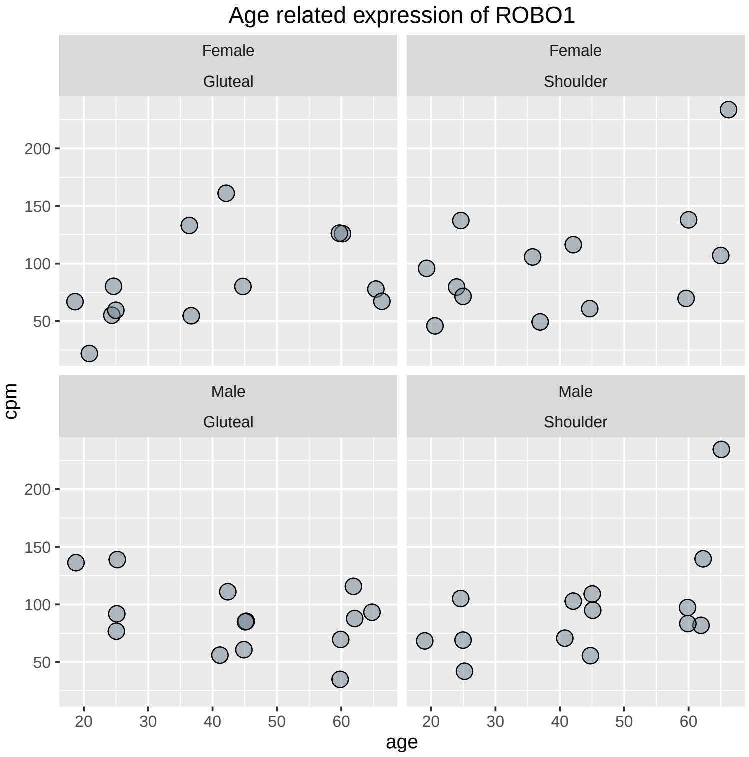

3.42 KCNC4

Figure 42: Genewise CPM estimates from SOV

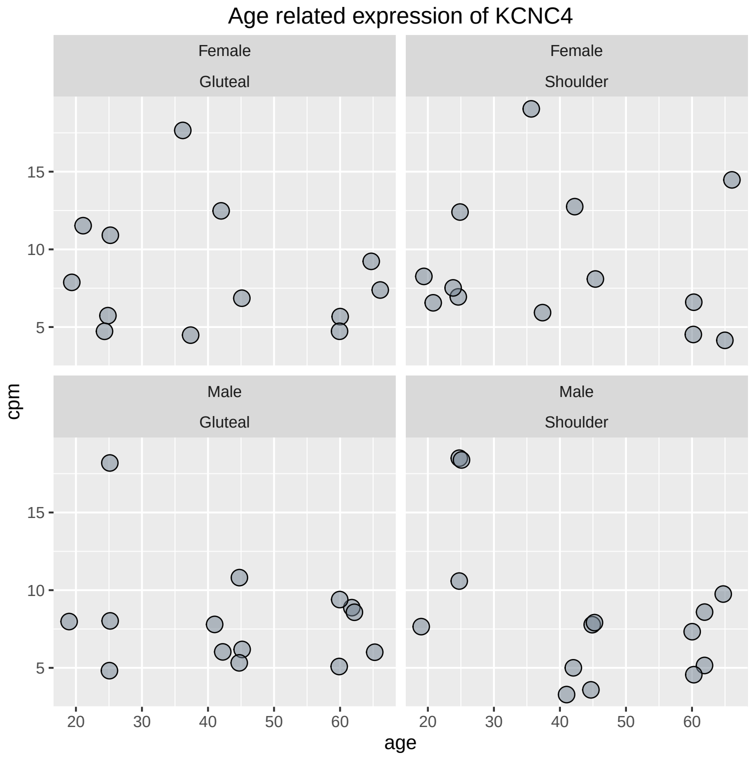

Supplement: S4 File — PDF file with summarizeOverlaps derived CPM data for the 42 age-related DE genes. (pdf) [file pone.0175657.s004.pdf]
